# Intraspecific variation of recombination rate in maize

**DOI:** 10.1186/gb-2013-14-9-r103

**Published:** 2013-09-19

**Authors:** Eva Bauer, Matthieu Falque, Hildrun Walter, Cyril Bauland, Christian Camisan, Laura Campo, Nina Meyer, Nicolas Ranc, Renaud Rincent, Wolfgang Schipprack, Thomas Altmann, Pascal Flament, Albrecht E Melchinger, Monica Menz, Jesús Moreno-González, Milena Ouzunova, Pedro Revilla, Alain Charcosset, Olivier C Martin, Chris-Carolin Schön

**Affiliations:** 1 Plant Breeding, Technische Universität München, 85354 Freising, Germany; 2 INRA, UMR de Génétique Végétale/Université Paris-Sud - CNRS, 91190 Gif-sur-Yvette, France; 3 Limagrain Europe, 63720 Chappes, France; 4 Centro Investigacións Agrarias Mabegondo (CIAM), 15080 La Coruña, Spain; 5 KWS SAAT AG, 37574 Einbeck, Germany; 6 Syngenta SAS, 31790 Saint-Sauveur, France; 7 BIOGEMMA, Genetics and Genomics in Cereals, 63720 Chappes, France; 8 Plant Breeding, Universität Hohenheim, 70599 Stuttgart, Germany; 9 Molecular Genetics, Leibniz Institute of Plant Genetics and Crop Plant Research (IPK), 06466 Gatersleben, Germany; 10 Misión Biológica de Galicia (CSIC), 36080 Pontevedra, Spain

## Abstract

**Background:**

In sexually reproducing organisms, meiotic crossovers ensure the proper segregation of chromosomes and contribute to genetic diversity by shuffling allelic combinations. Such genetic reassortment is exploited in breeding to combine favorable alleles, and in genetic research to identify genetic factors underlying traits of interest via linkage or association-based approaches. Crossover numbers and distributions along chromosomes vary between species, but little is known about their intraspecies variation.

**Results:**

Here, we report on the variation of recombination rates between 22 European maize inbred lines that belong to the Dent and Flint gene pools. We genotype 23 doubled-haploid populations derived from crosses between these lines with a 50 k-SNP array and construct high-density genetic maps, showing good correspondence with the maize B73 genome sequence assembly. By aligning each genetic map to the B73 sequence, we obtain the recombination rates along chromosomes specific to each population. We identify significant differences in recombination rates at the genome-wide, chromosome, and intrachromosomal levels between populations, as well as significant variation for genome-wide recombination rates among maize lines. Crossover interference analysis using a two-pathway modeling framework reveals a negative association between recombination rate and interference strength.

**Conclusions:**

To our knowledge, the present work provides the most comprehensive study on intraspecific variation of recombination rates and crossover interference strength in eukaryotes. Differences found in recombination rates will allow for selection of high or low recombining lines in crossing programs. Our methodology should pave the way for precise identification of genes controlling recombination rates in maize and other organisms.

## Background

In sexually reproducing organisms, crossovers (COs) stabilize the pairing of homologous chromosomes during meiosis and ensure their correct segregation. By reciprocal exchange of parental genetic material, the COs also lead to new allelic combinations and thus play an important role in creating genetic diversity. Meiotic recombination occurs during prophase I of meiosis, when DNA double-strand breaks (DSBs) catalyzed by the topo-isomerase-related enzyme SPO11 [1] are repaired via reciprocal exchange of genetic material between homologous chromosomes. The final number of recombination events depends on (1) the number of DSBs, and (2) the proportion of DSBs that are repaired as COs. The remaining DSBs may be repaired via other pathways leading to small conversions called non-crossovers, or may even be repaired using the sister chromatid instead of the homologous chromosome [2]. In organisms such as mice, humans, or plants, the number of DSBs is at least 10 times higher than the number of COs [3-5]. The number of COs can vary under the control of genetic factors [5] that affect recombination rate, but there is also some homeostasis of the CO number that modulates the CO/DSB ratio [6]. CO interference is a phenomenon observed in almost all organisms, by which two successive COs on a chromosome are rarely very close to each other [7,8]. The standard picture considers that close to a DSB that is repaired as a CO, other DSBs are preferentially repaired as non-crossovers [9-11], so interference may play a role in the regulation of CO numbers and distributions. Two distinct pathways of CO formation have been found to coexist in most species investigated, including *Saccharomyces cerevisiae*[12,13], *Solanum lycopersicum*[14], *Arabidopsis thaliana*[15], and *Mus musculus*[16]. In plants, the majority of COs are formed via pathway 1, which depends on genes of the *ZMM* family (hereafter referred to as P1), and which are subject to interference [11,14,17]. The remaining COs are formed via pathway 2 (hereafter referred to as P2), which depends on the *Mus81* gene, and in which there is little or no interference [18].

The meiotic recombination rate is known to vary among species [19,20]. In mammals, some intraspecific variation of local recombination rate has been revealed by sperm typing and has been explained by *cis* and *trans* genetic factors. In particular, the allelic diversity in the zinc finger domain of PRDM9, a DNA-binding protein, has been shown to influence recombination hot-spot activity [21,22]. In plants no PRDM9 homologs have been identified so far and little is known about the variation of the genome-wide recombination rates (GWRRs) within species. Most data come from chiasmata counts or linkage mapping experiments [20,23]. However, to our knowledge, there are hardly any cases in plants where many crosses involving distantly related parents have been used to compare recombination rates and infer the patterns of recombination along chromosomes based on high-density linkage maps. The same holds for CO interference: even though it plays an important role in determining recombination rate, intraspecific variation of interference has rarely been investigated in plants. Understanding the landscape of recombination within a species is of intrinsic interest, and it is also important in the context of genome-based prediction [24] and genome-wide association studies [25], since recombination determines the extent of linkage disequilibrium in populations under study. The extent of linkage disequilibrium has an impact on the linkage phase between predictive markers and quantitative trait loci (QTL) and on the marker densities required to find significant associations between markers and traits of interest.

Maize (*Zea mays* L.) has been the subject of genetic studies for more than a hundred years [26]. It is a diploid species (n = 10) with ancient polyploid origin [27]. The first genetic linkage map in maize based on DNA markers was constructed using restriction fragment length polymorphism markers [28]. Since then, many more genetic maps have been published based on restriction fragment length polymorphism, amplified fragment length polymorphism, or simple sequence repeat markers [29,30]. With increasing amounts of maize sequence information, single nucleotide polymorphism (SNP) markers were developed and used for diversity analysis and genetic mapping in maize [31,32]. Recently, the Illumina^®^ MaizeSNP50 genotyping array comprising around 50,000 SNPs was developed and the first high-density genetic maps of maize were published using two intermated recombinant inbred line populations [33]. These maps comprised up to 21,000 SNP markers and were compared with the maize genome reference sequence generated from the US inbred line B73, which is the largest and most complex plant genome sequenced so far [34]. The B73 AGP v2 assembly covers 2.07 Gb of the approximately 2.5 Gb maize genome.

For studying intraspecific variation of recombination rate in maize, we created two half-sib panels, one for Dent and one for Flint inbred lines. Dent and Flint are two maize gene pools that are important for hybrid breeding in Europe [35]. The two half-sib panels comprised a total of 24 full-sib families. The Dent panel comprises 10 crosses of a central Dent inbred line (F353) with diverse Dent founder inbreds of the European Dent gene pool that were developed in Europe and North America. In the Flint panel a central Flint inbred line (UH007) was crossed to 11 genetically diverse Flint founder inbreds of the European Flint breeding material. The Flint founder lines originate from early maize introductions to Spain as well as from so-called Northern Flints from France and Germany. In addition, two reciprocal crosses between the two central Dent and Flint lines were analyzed and in each panel one cross with the US Dent line B73 was included as a connection to the US nested association mapping (NAM) population [32].

Here, we report on the analysis of the variation of recombination rate in 23 populations of maize using SNPs from the MaizeSNP50 BeadChip [33]. All populations consist of doubled haploid (DH) lines obtained by *in vivo* haploid induction [36]. DH lines produced with this method reflect female meiosis. Unlike F_2_ or RILs, DH lines have the great advantage that the genetic information of each gamete is directly observed. Our objectives were to analyze intraspecific variation for (1) GWRR and chromosome-wide recombination rate, (2) recombination landscape along chromosomes, and (3) CO interference, using high-density genetic linkage maps. We found significant differences of GWRR between individual populations as well as between Dent × Dent versus Flint × Flint populations, but not between the Dent and Flint gene pools in general. GWRR was not correlated with the genetic structure of the lines as inferred from admixture analysis. We analyzed the recombination landscapes in all populations and found significant differences between individual populations and between pools. Finally, we characterized quantitatively the interfering and non-interfering CO formation pathways and found a negative correlation over all chromosomes between interference intensity of pathway P1 and GWRR.

## Results

We constructed two half-sib panels comprising a total of 24 maize full-sib families of DH lines for the analysis of intraspecific variation of recombination rates. The full-sib families within the two panels represent the diversity of important founder lines of the European Dent and Flint germplasm, respectively (Table 1; Additional file 1). In the Dent panel (prefix CFD), all full-sib families have the same common parental line F353 which was crossed to diverse Dent founder lines. The common parent of the Flint full-sib families (prefix CFF) is line UH007, which was crossed to diverse Flint founder lines. In all crosses, the haploid inducer line was used as male parent when crossed with the F_1_ plants for *in vivo* haploid induction, so our maps reflect only female meioses. Details on the diversity analysis of the parental lines are given in Additional file 2.

**Table 1:** Summary data of the genetic maps

**Population name**	**Parents**	**Cross type**^**a**^	**Remarks**	**Number of DHs**	**Total genetic map length (cM)**	**Genome-wide recombination rate (cM/Mbp)**	**Number of markers in framework map**	**Total number of markers**	**Largest gap (cM)**
CFD01	F353 × UH007	D × F	Reciprocal cross of central lines	86	1,536.6	0.748	701	14,112	9.0
CFD02	F353 × B73	D × D	Link to US NAM	73	1,319.6	0.642	474	14,955	9.2
CFD03	F353 × D06	D × D		103	1,347.3	0.656	756	13,195	10.9
CFD04	F353 × D09	D × D		105	1,432.9	0.697	833	12,336	9.8
CFD05	F353 × EC169	D × D		77	1,179.7	0.574	470	13,993	10.5
CFD06	F353 × F252	D × D		105	1,441.2	0.701	780	11,900	12.7
CFD07	F353 × F618	D × D		108	1,334.5	0.649	854	13,466	10.5
CFD09	F353 × Mo17	D × D		63	1,287.4	0.627	289	15,955	10.9
CFD10	F353 × UH250	D × D		99	1,288.7	0.627	763	13,583	10.0
CFD11	F353 × UH304	D × D		86	1,537.7	0.748	503	8,887	17.0
CFD12	F353 × W117	D × D		100	1,360.8	0.662	817	13,829	10.2
CFF01	UH007 × F353	F × D	Reciprocal cross of central lines	99	1,507.0	0.733	903	13,697	8.9
CFF02	UH007 × B73	F × D	Link to US NAM	120	1,666.6	0.811	1,215	16,765	6.4
CFF03	UH007 × D152	F × F		112	1,893.1	0.921	848	8,988	32.0
CFF04	UH007 × EC49A	F × F		53	1,535.6	0.747	279	12,385	10.5
CFF05	UH007 × EP44	F × F	Population too small for mapping	34	NA	NA	NA	NA	NA
CFF06	UH007 × EZ5	F × F		50	1,785.0	0.869	303	13,791	12.9
CFF07	UH007 × F03802	F × F		129	1,630.3	0.793	870	9,190	29.9
CFF08	UH007 × F2	F × F		77	1,554.9	0.757	495	10,045	15.5
CFF09	UH007 × F283	F × F		134	1,442.1	0.702	938	10,193	25.0
CFF10	UH007 × F64	F × F		108	1,437.3	0.699	892	13,502	8.3
CFF12	UH007 × UH006	F × F		114	1,654.6	0.805	683	7,274	15.6
CFF13	UH007 × UH009	F × F		117	1,768.6	0.861	563	6,379	53.7
CFF15	UH007 × DK105	F × F		115	1,751.6	0.852	832	9,200	28.4
DentAll^b^				919	1,358.9	0.661	NA	NA	17.0
FlintAll^c^				1009	1,643.4	0.800	NA	NA	53.7
All^d^				2233	1,517.4	0.738	NA	NA	53.7

^a^D, Dent; F, Flint. ^b^Pooled Dent populations. ^c^Pooled Flint populations (without CFF05). ^d^All populations pooled (without CFF05). cM, centiMorgan; NA, not available.

### High-density genetic map construction in maize full-sib families

As a first step in the analysis of recombination, individual genetic maps were constructed for 23 out of the 24 populations and each of the 10 chromosomes. Map statistics are given in Table 1 with more details in Additional file 3, and the complete list of marker positions in all maps with the raw segregation data is given in Additional file 4. In total, 39,439 SNPs of the MaizeSNP50 array could be mapped in at least one population. Over all populations, around 33,884 COs were observed in 2,233 female meioses and, on average, 11,988 SNPs were mapped in one given population. The average for Dent × Dent populations (all CFD except CFD01) was 13,247 markers mapped and for Flint × Flint populations (all CFF except CFF01 and CFF02) the average was 9,874. For population CFF05, which comprised only 34 DHs, no stable map could be obtained. The total length of genetic maps ranged from 1,180 centiMorgan (cM) in population CFD05 to 1,893 cM in CFF03 with a mean of 1,508 ± 185 cM (mean ± standard deviation). The average genetic map length in the 10 Dent × Dent populations (CFD02 to CFD12) was 1,353 ± 99 cM, while the 11 genetic maps from Flint × Flint populations (CFF03 to CFF15) were, on average, longer at 1,645 ± 154 cM. The three maps from crosses between Dent and Flint parents (CFD01, CFF01, CFF02) had an intermediate average length of 1,570 ± 85 cM. The largest gap in a genetic map was 53.7 cM on the long arm of chromosome 7 (7L) in population CFF13, where no SNPs were polymorphic between the parents, most likely due to identity-by-descent (IBD). Details about genome-wide diversity and IBD segments between parents of the mapping populations can be found in Additional file 2. Synteny and colinearity of the genetic maps was compared with the B73 AGPv2 genome assembly. In general, a high agreement was found, demonstrating the high consistency of marker order across populations, with few exceptions as described in Additional file 2.

We observed distorted segregation in all populations. Deviations from the expected allele frequencies of 0.5 are displayed in Additional file 5. All 10 chromosomes carried regions with distorted segregation. Populations and chromosomes differed in their patterns of distortion. Only in some rare cases shared features were visible - for example, in the Flint (CFF) populations, chromosome 2 tends to be distorted towards the common parent allele in the centromeric regions and towards the founder line alleles in the distal regions of the long arm. In none of the regions where known gametophytic (*ga*) genes are located (chromosome 3L, *ga7*; chromosome 4S, *ga1*; chromosome 5L, *ga2*; chromosome 9S, *ga8*; data from [30]) was a consistent segregation towards either central or founder line allele across the full-sib families observed.

### Intraspecific variation for genome-wide and chromosome-specific recombination rates in maize

**Figure 1:**
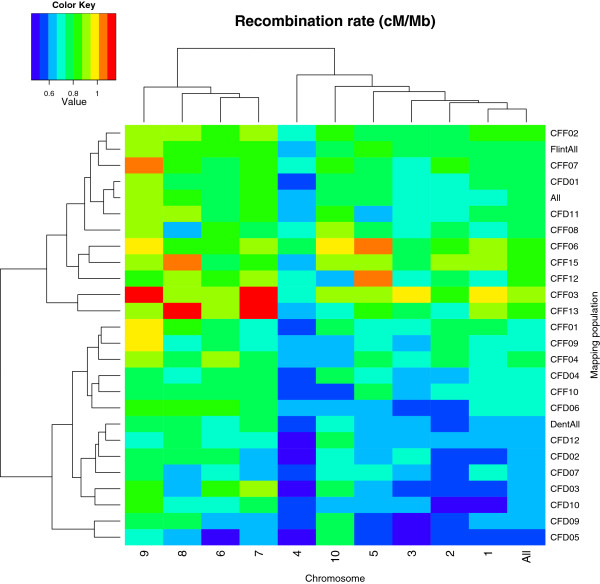
**Diversity of recombination rates.** Heat map of the chromosome-wide recombination rates measured for each chromosome in the 23 genetic maps. On the x-axis, 'All' corresponds to a pooled analysis of all chromosomes. On the y-axis, 'DentAll', 'FlintAll', and 'All' correspond to pooled analyses with all Dent × Dent populations, all Flint × Flint populations, and all 23 populations together, respectively. Warmer colors indicate higher recombination rates. Dendrograms indicate hierarchical clustering of -log_10_(*P* value) based on Euclidian distances, and were used to order the populations and chromosomes.

The average GWRR can be expressed as the ratio of total genetic map length in centiMorgans divided by the genome size in megabase pairs. The genome size of maize is approximately 2.5 Gb, of which 2.1 Gb are sequenced and assembled in the B73 AGPv2 reference sequence. We used the 2.1 Gb B73 AGPv2 assembly as the basis of our calculations. Similarly, the average recombination rate of one chromosome is the ratio between the genetic map length in centiMorgans and the physical length in megabase pairs of this chromosome. The physical lengths of the 10 maize chromosomes in the B73 AGPv2 assembly are between 301 Mbp for chromosome 1 and 150 Mbp for chromosome 10. Genetic map lengths were measured for each population based on the same physical positions of extremities, correcting for particular regions (for example, IBD segments) where mapping information was not available or not reliable. The average GWRR over all populations was 0.74 ± 0.09 cM/Mbp (mean ± standard deviation; Table 1). Figure 1 (details in Additional file 3) shows that the GWRR and chromosome-wide recombination rate varied among chromosomes and among populations. In particular, chromosome 9 recombined the most and chromosome 4 the least. Statistical tests for pairwise comparisons of average recombination rates between chromosomes and between populations confirm these observations (Additional file 6).

**Figure 2:**
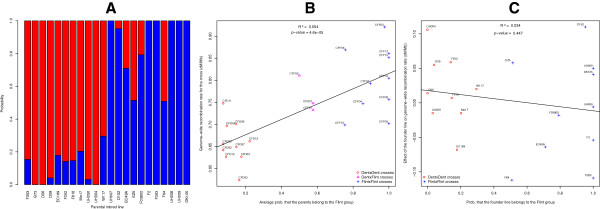
**Structure analysis of parental lines and correlation of structure with genome-wide recombination rate. (A)** Probability of each parental inbred line to belong to the Dent (red) and the Flint (blue) groups. **(B)** For each of the 23 populations, correlation between the average probability of the two parents of the population to belong to the Flint group, and genome-wide recombination rate (GWRR) in the population. **(C)** For each of the 19 founder lines excluding B73, correlation between the probability to belong to the Flint group, and the contribution to GWRR for that parental line, using an additive model whereby the GWRR for a cross is the average GWRR of the two parents of the cross. The effect of the central line was corrected for based on the two populations CFD02 and CFF02, which involve B73 crossed to the two central lines F353 and UH007.

The differences in GWRR between Flint × Flint populations and Dent × Dent populations were much larger than the intrinsic statistical uncertainties, leading us to consider that there is a genetic source of the variability observed for GWRR. To investigate to what extent some alleles more frequent in one pool than in the other may explain the diversity of GWRR, we first estimated for each parent its degree of 'flintness', based on admixture analysis performed on a large panel of lines including Dent and Flint accessions [37]. We then analyzed the correlation between the average 'flintness' of the two parents of a cross and the GWRR of the resulting population. The results are shown in Figure 2, with the probability of belonging to the Dent and the Flint groups for each parental line (Figure 2A) and the highly significant correlation (*P* value = 4.6 × 10^-5^) observed between the GWRR in a population and the average 'flintness' of its parents (Figure 2B). This population structure effect explained 55% of the variance of the GWRR.

Nevertheless, we observed large differences in the GWRR of the two crosses of both central lines with B73 (CFD02 and CFF02, respectively). The GWRR in CFD02 was 0.642 cM/Mbp, whereas in CFF02 the GWRR was 0.811 cM/Mbp. We thus asked whether the significantly higher GWRR in the pooled Flint × Flint populations might follow from differences in the central parents F353 and UH007. To test this, we took an additive model whereby the genetic length of a genetic map is the average of effects contributed by each of its parental lines. These parent-specific effects were determined using the fact that the line B73 was crossed to both central lines (F353 in CFD02, and UH007 in CFF02), thus connecting the two half-sib panels. Then we tested for a correlation between founder line effects on GWRR and their 'flintness'. As shown in Figure 2C, no significant correlation was found (*P* value 0.45), indicating that in the founder lines studied here, no general difference in GWRR can be observed between Dent and Flint inbred lines. We conclude that the broad range of variation observed in Figure 2B originates from (1) a strong difference in GWRR between the two central lines F353 and UH007, and (2) additional variation between the different founder lines, as observed along the y-axis of Figure 2C.

### Intraspecific differences in recombination landscapes

**Figure 3:**
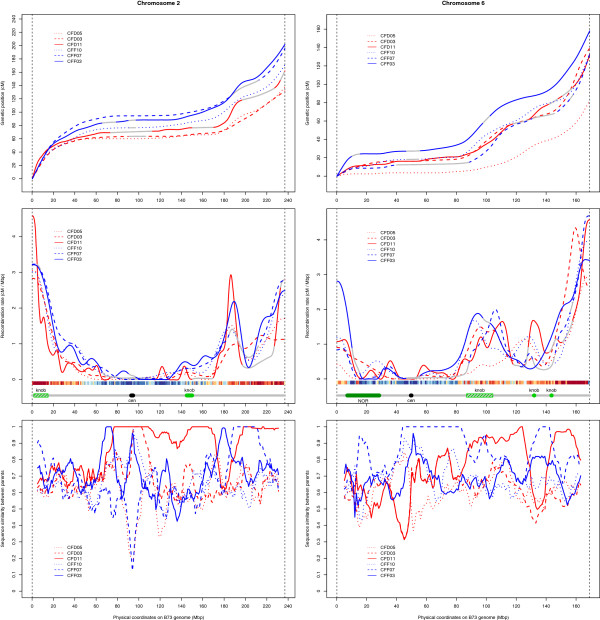
**Recombination rates along chromosomes.** The x-axis indicates the physical position (Mbp) along chromosomes 2 (left panels) and 6 (right panels). The y-axis indicates the genetic position (cM; top panels), recombination rate (cM/Mbp; middle panels), and pairwise parental similarity (frequency of identical SNP alleles in 10 Mbp sliding windows with a step size of 2 Mbp; bottom panels) for six of the 23 populations, after smoothing and imputation. Blue lines: Flint × Flint crosses. Red lines: Dent × Dent crosses. In both groups, the solid, dashed and dotted lines correspond to the population with the highest, median and lowest genome-wide recombination rate within its group, respectively. Gray parts of the lines correspond to regions where the information was missing or not reliable (IBD segments, non-colinearity with B73), and was thus imputed from the other maps. Heat maps below the curves of recombination rates indicate gene density (low for cold colors and high for hot colors). Gray horizontal line below the heat-map: sketch of the chromosome organization showing centromeres (cen), knobs, and nucleolar organizer region (NOR). Centromere, knob and NOR positions are from [30]. Color filling of chromosome features is solid when the estimated boundaries of the region are known, and hatched when the box indicates only the extremities of the bin containing the region.

In addition to GWRR, we analyzed the patterns of recombination rate along the chromosomes, hereafter referred to as recombination landscapes, and compared them for each pairwise combination of linkage maps and between Dent and Flint pools. We wanted to test whether there were intraspecific differences for recombination landscapes given the large intraspecific diversity in maize and the fact that we observe a clear differentiation between lines in genetic diversity analyses (see Additional file 2 for details) and GWRR. To compare recombination landscapes, genetic positions of the markers were plotted against their physical positions to obtain Marey maps. Most such maps showed a rather smooth and monotonic pattern (for example, CFD01 chromosome 1 in Additional file 7). However, in some maps, there were large regions void of polymorphic markers (for example, CFF07 chromosome 1 in Additional file 7), or the map yielded two parallel horizontal lines of markers (for example, CFF01 chromosome 3 in Additional file 7). Such parallel lines could be explained by duplicated chromosomal segments, possible errors in the B73 assembly, and/or non-copy-specific SNP markers that would detect paralogous sequences. In such cases, the missing information was imputed from all other maps. After smoothing and forcing monotonicity (Additional file 8), the derivative was calculated to indicate the local value of recombination rate. The shape of the recombination landscape varied from chromosome to chromosome, and for a given chromosome this shape varied among the populations. This is illustrated in Figure 3 for six populations representing the extremes and the median GWRR in each pool for chromosomes 2 and 6. All chromosomes are shown in Additional file 9. On all chromosomes, recombination was close to zero around the centromeres (compare the almost horizontal curves in the Marey maps and minima in the curves of recombination rates) and increased towards the telomeres. Other structural elements in the maize genome also lead to locally reduced recombination rates, such as the nucleolus organizer region (NOR) on chromosome 6 (Figure 3; Additional file 9). Up to 34 heterochromatic knobs have been described in different maize accessions; however, for only a few of those is the physical position available and knob size can vary across lines [38]. Although no cytological data about knob positions and size variation in our lines are available, some variation can be expected. In several cases recombination rates were reduced in one or more populations around the known knob positions - for example, on chromosomes 1L, 2L, 3L, 6L, 7L and 9L (Figure 3; Additional file 9) - but in some other cases, knobs were in highly recombining regions.

To compare the shapes of the recombination landscapes independently of the chromosome-wide rate of recombination, we normalized the local rates by the chromosome-wide rate. Using these normalized data, statistical tests based on 10 bins of equal genetic length per chromosome revealed significant differences in the shape of the recombination landscapes. In a population-wise comparison considering all chromosomes of a population together (Additional file 10), four populations, CFF03, CFF07, CFF12, and CFF13, clearly showed recombination landscapes significantly different from those of most other populations. Furthermore, when looking at individual chromosomes (Additional file 10), some populations, such as CFD11 for chromosome 1, CFD04 for chromosome 4, or CFF08 for chromosome 9, showed significantly different patterns in their recombination landscapes. In addition, COs were pooled across all Flint × Flint and across all Dent × Dent populations to compare the recombination landscapes between pooled Flint and Dent populations. For chromosomes 2, 4, 5, and 6, there were highly significant differences between recombination landscapes in the Dent and in the Flint pools (Bonferroni-corrected *P* value < 0.01). Significant local differences in the shape of the curves between the pooled Dent and the pooled Flint populations were found, for example, in the centromeric bin 5 and in bin 8 on chromosome 2 or in bin 6 on chromosome 6 (Additional file 11).

Finally, to reveal a possible correlation between local recombination rates and the pairwise genetic similarity of parental genomes, we used a sliding window of 10 Mbp within which we calculated a similarity index as the fraction of shared SNP alleles between the two parents. Overall, no consistent relationship was observed between local parental genome similarity and recombination rate. Peaks of recombination rates were associated with regions of high and low genetic similarity (Figure 3, Additional file 9). In all centromeric regions, recombination rates were very low, but values for pairwise parental similarity were highly variable.

### Crossover interference analysis across populations

Crossover interference leads to a more regular spacing between adjacent COs than in the absence of interference, where COs are positioned randomly. Interference also affects the distribution of CO numbers per gamete by reducing its variance. In our data, the observed distributions for CO numbers and inter-CO distances departed from the ones expected without interference, allowing us to reject the hypothesis that there is no interference. We also characterized interference quantitatively by estimating the two parameters of the Gamma-sprinkling model [11]: (1) the intensity *nu* of interference in the interfering pathway, hereafter referred to as P1, and (2) the proportion *p* of COs formed through the non-interfering pathway, hereafter referred to as P2. The parameters *nu* and *p* were estimated for each chromosome and each population. These parameters greatly varied among chromosomes and among populations, with values of *nu* ranging from 2 to 50, and *p* ranging from 0 to 0.4. Since the population sizes are modest, part of this variation is expected to be due to statistical noise. We thus focused on comparing pooled data, either over chromosomes or over populations.

**Figure 4:**
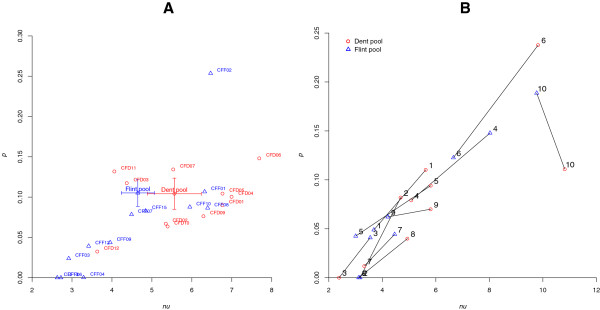
**Diversity of interference characteristics.** Parameter *nu* of the Gamma model measuring interference intensity in pathway P1 (x-axis), versus fraction *p* of crossovers formed via the non-interfering pathway P2 (y-axis). **(A)** Parameters estimated for each population and the 10 chromosomes pooled together. Red circles: Dent × Dent populations. Blue triangles: Flint × Flint populations. Corresponding population names are indicated beside each point. Pooled data for the two pools Dent and Flint are indicated with their 95% confidence intervals (error bars). **(B)** Parameters for the pooled data of all Dent × Dent populations (red circles) and all Flint × Flint populations (blue triangles) estimated for each individual chromosome. Corresponding chromosome numbers are indicated beside each point.

Figure 4A shows the values estimated for *nu* and *p* when pooling all chromosomes together for each population and for the pooled Dent × Dent and Flint × Flint populations. Confidence intervals for estimates of *nu* and *p* from individual populations were large as expected given the population sizes (data not shown). Statistical tests showed that differences between individual populations were not significant at the 5% threshold level - hardly any pairs of individual populations had significantly different values for *nu* (Additional file 12). For the pairwise comparisons of populations pooling all chromosomes, only CFF06 and CFF13 had significantly lower values for *nu* than four or six other populations, respectively. For *p*, however (Additional file 13), its value was found to be significantly higher in CFF02 than in almost all other populations.

Figure 4B presents the values for *nu* and *p* for each chromosome estimated from the Dent × Dent and Flint × Flint pools. Dent × Dent populations tended to have both higher *nu* and higher *p* than Flint × Flint populations for most chromosomes. On the other hand, chromosomes 3, 4, and 7 showed a reversed pattern, while for chromosome 10 the Dent × Dent pool had higher *nu* but lower *p*. Pooling all chromosomes together, the interference strength *nu* observed across our populations ranged approximately between 2 and 8. To provide a qualitative understanding of the meaning behind these values, one can ask how much such interference levels reduce the probability of having a second CO near a first one in the same meiosis for COs formed within the interfering pathway P1. We found that the probability of having a second P1 CO at 40 cM from a first one is reduced by a factor between 1.8 (for *nu* = 2) and 20 (for *nu* = 8). At 10 cM from the first CO, this reduction factor ranges between 5 (for *nu* = 2) and 46,000 (for *nu* = 8). For *nu* > 3 there is almost no chance to find two P1 COs separated by less than 10 cM. Interference intensity in P1 (*nu*) was significantly negatively correlated with GWRR for chromosome 7. For other chromosomes, this correlation was not significant, but it was when pooling all chromosomes together (Additional file 14). On the other hand, no correlation was found between GWRR and the proportion *p* of non-interfering P2 COs, irrespective of the chromosome and pooled over all chromosomes (*P* values always > 0.18).

## Discussion

Recombination and reassortment of parental genomes lead to an increase of genetic variation. Understanding the mechanisms regulating recombination rate during meiosis would allow their manipulation to increase or decrease recombination rates according to specific requirements described in [39]. Although many genes controlling the basic process of CO formation have been identified, little is known about factors influencing CO numbers and distribution, and GWRR. To our knowledge the present work is the most comprehensive study comparing meiotic recombination rates and CO interference within one species and between gene pools of the same species. We used two panels of maize half-sib families comprising 23 full-sib populations with a total of 2,233 DH lines to analyze intraspecific variation of recombination rates and recombination landscapes. The parents of the two panels for Dent and Flint maize were chosen to represent the diversity present in European maize germplasm. We analyzed DH lines produced by *in vivo* haploid induction, which reflect a single female meiosis. As expected, the average number of crossovers per DH line in our study (15.1) was about half the number observed in maize RILs (28.9) [32]. This drawback of DH lines is counterbalanced by the faster development of the DH populations compared to RILs and by the complete homozygosity of DH lines, which are an immortal resource. Moreover, working on DH populations offers the unique possibility to analyze CO interference, while this is impossible in RILs because the successive independent meioses superpose the COs arising during each meiosis. The design of our connected half-sib panels comprising a large number of populations allowed for comparisons of GWRR, recombination landscape, and interference (1) across parents within each panel and (2) across panels via crosses of the central lines with line B73.

As a prerequisite for our approach, we constructed high-density genetic maps for a large number of populations. Genetic map lengths of the 23 populations varied from 1,180 cM to 1,893 cM with a mean of 1,508 cM, which is in the range observed in other high-density genetic maps of maize [32,33]. Due to IBD regions in some of our populations, gaps were observed in the genetic maps. However, since most of these gaps were in pericentromeric regions, where recombination rates are low, most of them were not larger than 15 cM, the most extreme gap (53.7 cM) being on chromosome 7 in CFF13. For 76 markers, we found chromosome assignments different from their annotation in the B73 AGPv2. We provided genetic coordinates for 118 markers where no annotation was available. These results may help to improve future B73 genome assemblies. In addition, genetic maps for several chromosomal regions were found non-colinear with the B73 sequence, as was previously detected from two other maize populations [33]. These discrepancies may be the consequence of mis-assemblies in the B73 genome, or due to lack of locus specificity for some SNP markers that would fall into duplicated genomic regions. The hypothesis of structural rearrangements between some of the parental lines and B73 cannot be excluded. However, given the design of the experiment, the possibility that both parents of a cross within one of the half-sib panels share a structural variation absent from both parents of another cross in the same panel is very limited.

### Intraspecific variation of recombination rate

The average GWRR in our populations was 0.73 cM/Mbp, which is in the same range as can be calculated from other maize maps [30]. This value is about 5-fold lower than in *A. thaliana* (3.6 cM/Mb) [40] which has a 20-fold smaller genome than maize, reflecting the well-known negative correlation of recombination rate with genome size among species [41]. We found clear differences between chromsome-wide recombination rates, with chromosome 4 having the lowest average value (0.60 cM/Mbp) and chromosome 9 the highest (0.88 cM/Mb). We also observed a negative correlation between recombination rate and the physical length of the chromosomes (*r* = 0.66, *P* value 0.003), similar to what arises in human, mouse and rat [19]. Such correlations suggest that the mechanisms regulating CO formation in these organisms tend to enforce some level of homeostasis in the number of COs per meiosis and per chromosome. For very short chromosomes, the obligatory CO ensures that there is at least one CO for each bivalent. We observed clear intraspecific variation for GWRR in this study. Similar levels of variation for GWRR were observed in both pools once the effect of the central line was removed.

Calculating GWRR assumes constant genome size in maize. Genome size differences were reported in maize, with temperate maize lines having up to 10% smaller genomes relative to B73 and these differences were correlated with the number of knob repeats [42]. Genome sizes for the lines in our study are unknown; however, the up to 60% difference in map lengths (1,180 to 1,893 cM) in our study is much larger than the genome size differences recently reported [42]. Therefore, it can be assumed that beyond possible genome size variation genetic factors influencing GWRR play a strong role in our plant material. Differences in recombination frequencies between maize inbred lines have been described based on genetic linkage maps and by using cytological methods to detect recombination nodules and to calculate CO rates [3,43]. *Trans*-acting QTL affecting GWRR were identified in *A. thaliana*, maize, mouse, and wheat [44]. In cattle, several QTL were mapped for male GWRR [45]. For two QTL, putative causal variants were detected in the genes *REC8* (a member of the kleisin family of 'structural maintenance of chromosome' proteins), and *RNF212*, a putative homolog of the yeast *ZIP3* gene, which is involved in meiotic recombination. *RNF212* is also known to be associated with genome-wide recombination in humans [46]. Our findings on different GWRR between individual maize lines pave the way for development of specific crosses to identify genetic factors influencing GWRR by QTL mapping [47]. Given the advances in high-throughput genotyping and genome sequencing, such QTL can be a starting point for fine-mapping and subsequent cloning of genes determining GWRR in plants. Characterizing the structural and functional variation of such genes would promote our understanding of the molecular mechanisms regulating meiotic recombination in plants and would be a highly valuable tool for plant breeders and geneticists.

### Recombination landscapes in maize

Not only GWRR but also the landscapes of recombination along chromosomes are highly variable in our populations. We observed characteristic shapes of Marey map curves for each of the 10 maize chromosomes. The overall recombination profiles for each chromosome tend to follow gene density [34] but this does not explain local differences in recombination rate between populations. Structural variation between parental lines may be one mechanism influencing local recombination rates, as shown for a 26 kb retrotransposon cluster that reduced local recombination rate around the *bz1* locus by a factor two [48]. For the same genomic region it was shown that haplotype structure, as defined by the presence of helitrons and retrotransposons, strongly affected the occurrence of recombination events in heterozygous plants [49]. The extensive structural variation in the maize genome can be seen already at the karyotype level, where large-scale variation was reported [50], but even more at the sequence level, where large variation was observed for repetitive element content, presence-absence or copy number variants [42,51,52]. Such structural variations may influence the pairing of homologs and recombination [53], although inverted regions may also pair normally in pachytene [54]. Apart from the low recombination rates in the heterochromatic pericentromeric and NOR regions and a general increase of recombination rates towards the telomeres, we observed kinks in our Marey map curves in regions where heterochromatic knobs have been mapped in maize. This is the case, for example, on chromosome 4L in all populations, but only in some populations on chromosome 1L, suggesting that variation in knob regions may exist in our lines. Although 34 distinct knob regions were described in maize and its wild progenitor teosinte, most maize lines contain fewer than 12 such knobs, for which in addition polymorphisms are observed between lines [38,55]. Due to a lack of data on knob positions in our parental lines, we could not examine the influence of knob polymorphism on the shape of the recombination landscapes for individual chromosomes more closely. Since knobs are often located in gene-dense regions and suppress local recombination [38] it is likely that some differences in the shapes of the recombination landscape are caused by knob polymorphism. Also outside putative knob regions, we observed many significant local differences in the pairwise comparison of recombination profiles between populations and for chromosomes 2, 4, 5, and 6 between the pooled Dent and Flint populations. We found no significant correlation between parental genetic similarity as determined by SNP markers and recombination rate, a result corroborated by a recent study in *A. thaliana*[40]. Thus, factors influencing local recombination rates other than gene density and similarity at the DNA level must exist. It must be stated though, that for characterizing the influence of genomic features such as nucleotide diversity on local recombination rate, the 10 Mbp scale may be too coarse, so much higher resolution at the kilobase scale might be required, as recently shown in the model plant *Medicago truncatula*[56]. In addition, the parental diversity as assessed by the mainly genic SNPs of the MaizeSNP50 array may not well reflect the structural differences that can have a major impact on local recombination rates [49]. Our study has identified genomic regions with large differences in local recombination rates between inbred lines. This is an important basis for future studies to identify recombination hotspots and to study the influence of structural variation and genome diversity in defined crosses and genomic regions in maize.

### Crossover interference

Interference was previously shown to occur in maize [17], based on numbers and positions of late recombination nodules. That work found two pathways to be operating in maize, one interfering (P1) and the other (P2) contributing a proportion *p* of non-interfering crossovers. In the present study, we also found in most cases values of *p* significantly different from zero, with values averaging 0.1. This conclusion is compatible with the previous estimations that reported an average value of 0.15 [17]. The populations where we found *p* = 0 (CFF04, CFF06, CFF13) may mostly reflect a low power due to limited population sizes: here, individual populations have between 50 and 134 DH lines, whereas in [17], the data set had more than 200 pachytene synaptonemal complexes (SCs), each of them giving about four times more power to the analysis than one DH line. It should be noticed, however, that our statistical tests were very conservative due to Bonferroni correction. Still, we found that CFF02 had a proportion *p* of non-interfering (P2) crossovers significantly higher than almost all other populations when considering all chromosomes pooled. To our knowledge, differences in interference features between different genotypes of the same species have not been shown so far. Values of *p* between 0 and 0.2 for different chromosomes were reported in *A. thaliana*[11], and between 0 and 0.21 in humans [57]. Based on comparisons between MLH1 foci and late recombination nodules, *p* values around 0.3 were found in tomato [14]. Considering the intensity *nu* of interference in the interfering pathway P1, our results indicated values ranging between 2.5 and 8 for all chromosomes pooled, which is similar to the range 4 to 10 found previously in maize [17]. In *Arabidopsis*, *nu* was in the 10 to 21 range [11], whereas *nu* was estimated to be in the 6.9 to 7.9 range in tomato [14]. Finally, based on the 23 full-sib populations, we found a significant negative correlation between the chromosome-pooled *nu* and the genome-wide recombination rate. This result is consistent with the hypothesis that interference may be one of the mechanisms at work to regulate the level of meiotic recombination, biasing the repair of DSBs towards non-COs rather than COs. Compared with the highly significant differences found for GWRR in our study, the variation for CO interference is detected here with much less statistical power. In future works with higher population sizes, providing smaller confidence intervals, but using the same half-sib design it should be possible to estimate the effects of the founder parents alone on interference parameters by removing the effects of the central lines, as we did for GWRR. Similarly as for GWRR, this should also enable the identification and localization of genetic factors influencing interference parameters.

### Impact of variation in recombination rates on genetic studies and applied breeding programs

Covering the whole genome using dense genetic maps increases the chance to detect marker-trait associations, both in linkage analyses and association mapping. The construction of high-density genetic maps is now feasible for many crop species in a very cost-efficient way, either by SNP genotyping arrays or through genotyping-by-sequencing approaches [33,58]. For precise estimation of QTL effects in genome-wide association studies and high accuracy genome-wide prediction of breeding values, it is a prerequisite that markers tag either the causal alleles or they must be in high linkage disequilibrium with the QTL of interest [24,59]. The linkage phase between marker and favorable QTL alleles is crucial when predicting breeding values across breeds or gene pools [60]. Recombination events may invert linkage phases of marker and QTL alleles between unrelated pools, and thus lead to reduced accuracies in prediction of breeding values and marker effects. In the context of genomic prediction or genome-wide association studies, understanding the landscape of recombination within a species is of particular interest, since regions with high recombination rates require higher marker densities. In addition, a detailed genome-wide and local picture of recombination rates permits adequate dimensioning of map-based cloning projects, marker-assisted selection strategies for specific traits, and crossing programs in cases where unfavorable linkage between traits needs to be broken. Recombination is a key factor determining the success with introgression of new variability from distantly related plant genetic resources or poorly adapted material, since introgression of new alleles or traits such as resistance genes in recurrent selection is often accompanied by undesired linkage drag. These effects of linkage drag may be drastic if the regions of interest are located in (peri)centromeric, recombination-poor regions. Choosing elite lines with high GWRR as recurrent backcross parents may help to speed up the introgression process. Finally, identifying genotypes carrying alleles for higher recombination rate may guide the choice of adequate parents for optimizing the number of generations required in breeding schemes. Variability in interference strength may also be of interest in breeding programs because interference is believed to mechanistically affect recombination rates. Just as for the selection of lines with higher or lower recombination rates [61], it should be feasible to develop maize lines with different levels of CO interference or proportion of P2 COs.

## Conclusions

Meiosis and recombination fundamentally influence genome structure and evolution of species. Although key components of the meiotic pathway are known in plants, further research is needed to better understand the interplay of genetic factors controlling recombination, the role and mechanism of CO interference and the influence of chromatin features such as methylation and structural variation on recombination in plants. Recent advances in high-throughput genotyping and next-generation sequencing offer new opportunities to analyze at a large scale the genome-wide distribution of COs and to characterize sequence motifs around CO hotspots. The present study revealed large intraspecific variation for GWRR and recombination landscapes in maize. Our findings pave the way for the selection of proper crossing parents to analyze genetic factors influencing GWRR and local recombination rates. Finally, using appropriate experimental designs, QTL or association studies will enable the identification of QTL for GWRR.

## Materials and methods

### Plant material

Two half-sib panels of 11 and 13 half-sib families were established in the Plant-KBBE project CornFed, one for European Dent and one for European Flint maize. The lines used in this study, their origins, and assignment to Dent or Flint pools are listed in Additional file 1. Each of the two panels consists of a central line (or common parent) that was crossed to founder lines that represent important and diverse breeding lines of the European maize germplasm. The central line (F353) of the Dent panel was crossed with 10 Dent founder lines. For the Dent panel the prefix for populations is CFD. In the Flint panel, the central line UH007 was crossed with 11 Flint founder lines and the prefix for populations of this panel is CFF. In addition, each of the founder lines was crossed with B73 and also the reciprocal populations F353 × UH007 and UH007 × F353 were generated. These additional populations were made to connect the two panels with each other and with the US NAM population [32] via the parental line B73. The crossing scheme of the two half-sib panels and their connection is shown in Additional file 15. All progenies were homozygous DH lines obtained from F_1_ plants. The resulting 24 DH populations consisted of 35 to 129 lines (Table 1). In total, 2,267 DH lines were used for analysis in this study.

Crosses between central and founder lines were made by hand-pollinations using F353 or UH007 as female lines and the founder lines as males. F_1_ plants were pollinated with an inducer line for *in vivo* haploid production followed by chromosome doubling and selfing of D_0_ plants, and subsequent multiplication to obtain D_1_ plants [36]. Atypical lines within a cross or atypical plants within rows of DH lines were eliminated based on phenotypic observations.

### SNP genotyping

Bulk samples of dried leaves or kernels from up to eight D_1_ plants derived from the same D_0_, were used for DNA extraction using the cetyl trimethylammonium bromide (CTAB) procedure. DNA samples were adjusted to 50 to 70 ng/μl and 200 ng per sample were used for genotyping. DH line purity and integrity was first checked using a custom 96plex VeraCode assay (Illumina^®^, San Diego, CA, USA) with genome-wide SNP markers to ensure that the lines carried only one of the parental alleles at each SNP, that they did not carry alleles of the inducer line and that they were derived from true F_1_ plants. For a subset of DH lines, 13 proprietary SNP markers assayed with the KASP™ technology (LGC Genomics, Berlin, Germany) were used for testing line purity and integrity. True DH lines were then used for genotyping with the Illumina^®^ MaizeSNP50 BeadChip [33] on an Illumina^®^ iScan platform. Array hybridization and raw data processing were performed according to manufacturer’s instructions (Illumina^®^). Raw data were analyzed in Illumina^®^’s Genome Studio software version v2011 (Illumina^®^) using an improved version of the public cluster file (MaizeSNP50_B.egt, [62]). SNP data were filtered based on the GTscore using a threshold of 0.7. Heterozygous SNPs were set to missing values (NA) and only markers with a minor allele frequency >0.1 per population were used for mapping. For each population, the allele of the central line was coded as the 'A' allele, and the allele of the founder line was coded as 'B' allele (Additional file 4). Raw genotyping data of parents and DH lines are available at NCBI Gene Expression Omnibus as dataset GSE50558 [63].

### Analysis of parental genetic diversity

Genetic diversity between parental lines was assessed with genome-wide SNP markers by principal coordinate analysis, cluster analysis, and by a pairwise genome scan for polymorphism between the parents of each population. For details, see Additional file 8.

### Genetic map construction

Genetic maps were constructed for each individual population as described earlier [33] using CarthaGene [64] called from custom R scripts. In the first step, statistically robust scaffold maps were constructed with marker distances of at least 10 cM. In a second step, marker density was increased to produce framework maps containing as many markers as possible, while keeping a LOD score >3.0 for the robustness of marker orders. Finally, the complete maps were obtained by placement of additional markers using bin-mapping [65]. CentiMorgan (cM) distances were calculated using Haldane’s mapping function [66]. Individual genetic maps and genotypic data used for construction of the maps (Additional file 4) were deposited at MaizeGDB under the project acronym CORNFED [67].

### Physical map coordinates of SNPs

Chromosome and position assignments of SNPs of the MaizeSNP50 BeadChip supplied by the manufacturer (Illumina^®^, San Diego, CA, USA), are based on the B73 AGPv1 assembly with many markers lacking a chromosome and/or position information. We therefore performed a new mapping of the SNPs on the B73 AGPv2 assembly [68] using BWA [69]. The new assignments were used for all analyses involving the physical mapping information. Assignments are available in Additional file 4.

### Construction of bare and masked Marey maps

Given a chromosome and the associated genetic map of an individual population, we determined the marker positions on the B73 assembly. From these physical and genetic positions, we constructed a first Marey map [70] containing all syntenic markers. This Marey map was smoothed using cubic spline interpolations [71], producing a 'bare' Marey map that was forced to be monotonic. Then regions where mapping information was lacking (for example, segments IBD in the parents) were masked, producing 'masked' Marey maps (Additional file 9). The detailed procedure is explained in Additional file 8.

### Recombination landscapes

Once a bare Marey map was constructed, we defined the recombination landscape function as its derivative. Since the bare map was monotonic by construction, the recombination rates were positive as they should be. In effect, this landscape function provided the local recombination rate (in cM per Mbp) for any physical position of the B73 assembly. Note that this procedure did not distinguish the regions where these recombination rates were estimated reliably from those where they were not (unmasked versus masked regions). For comparison tests, it was thus necessary to resort to imputation, that is, to infer missing data from other maps in a conservative way.

### Imputed Marey maps for comparison tests

To compare the genetic lengths and the recombination landscapes between two different populations or pools of populations, we used 'imputed' Marey maps where the information missing in the masked Marey map of either population was replaced by the information available in the other population. If a region had masked data in both populations, its content was imputed using the averaged data of all other populations. In all cases, the imputation procedure was designed so that missing or unreliable data in either population to be compared never induced artificial differences. The detailed procedure used for imputation is explained in Additional file 8.

### Comparing genetic map lengths

For any given population, mapping data led to an estimate of the genetic length for a given chromosome. We examined pairwise differences in genetic length between populations as well as differences between pools of populations and tested them for their level of statistical significance. Such comparisons were performed from the imputed Marey maps with a significance threshold of 5%, using the welch.test() function in the R software and the conservative Bonferroni correction for multiple testing. The origin of the information (original or imputed data along the chromosomes) was taken into account when comparing populations or pools of populations. For details see Additional file 8.

### Effect of population structure on recombination rate

Genetic map lengths tended to be longer in populations involving Flint parents, suggesting that some alleles of factors controlling recombination rate may be differentially fixed in the two pools. To use a solid and objective measure of degree of 'flintness', we estimated the probability of the 22 parental lines to belong to one of the two main groups (Dent or Flint). To do this, we estimated admixture in a combined analysis of the Dent and Flint diversity panels described earlier [37], which included also the lines of our study. This analysis was done with the Admixture software (version 1.22) [72], using 25,237 PANZEA SNPs and 559 maize lines. We chose k = 2 for the number of groups assuming the two pools Dent and Flint, and used the probability of each of the 22 parental lines to belong to the Flint pool for a correlation analysis with recombination rates. More precisely, we analyzed the correlation between the GWRRs of the 23 populations and the average of the 'flintness' of the two parents of each population using the function lm() of the R software. The associated R^2^ specifies what fraction of the variance in the GWRR is explained by the group structure of the parental lines. The function lm() also provides the *P* value for testing the absence of correlation.

### Individual additive effects for recombination rate

The 23 genetic map lengths we estimated showed a clear positive correlation with the average 'flintness' of the parents in the crosses. However, the two central lines could be driving this correlation. To remove effects coming from the two central lines, we considered an additive model whereby the GWRR of a population produced by a cross is given by the average of two effects, one from each parent in the cross. For each founder line except for B73, there is a single cross in which it is involved. We took the GWRR of that cross and subtracted the GWRR of the cross involving the same central line and B73. This difference gives the individual additive effect of the founder line minus that of B73 up to a constant. This constant does not affect a putative correlation between individual-specific 'flintness' and GWRR. We performed the statistical test for significance of this correlation using the same procedures as in the previous section, central lines and B73 omitted.

### Comparing recombination landscapes

Just as the genetic length of chromosome maps may differ, two recombination landscapes can have different features (different shapes of the Marey maps). To test whether these differences were statistically significant, we normalized the genetic lengths of the two maps or pools of maps to be compared, by rescaling both of them to their mean value. Then, to compare the shape of both normalized Marey maps, our approach was based on binning the landscapes, representing each as a histogram and then applying a chi-squared test with a conservative Bonferroni-corrected significance threshold of 5% (Additional file 11). The detailed procedure is explained in Additional file 8.

### Interference analyses

CO interference was modeled in the framework of the Gamma model [73], including a second pathway using the sprinkling procedure [11] whereby non-interfering pathway P2 COs are simply added to those of P1. So the features of CO distributions along chromosomes were modeled using two parameters: the intensity *nu* of interference in the interfering pathway P1, and the proportion *p* of COs formed through the non-interfering pathway P2. The detailed implementation of the maximum-likelihood method used to estimate the values of the two parameters *nu* and *p* of the model was described earlier [17].

To analyze interference in a pool of maps instead of an individual map, we used a similar maximum-likelihood approach, but the likelihood to be maximized was the product of likelihoods calculated for the individual maps. To test for differences between values of *nu* or *p*, we applied the Welch test (function welch.test() in R) with significance thresholds of 5%, using variances estimated from the Fisher information matrix obtained at the optimal values of the parameters.

## Abbreviations

cM: CentiMorgan; CO: Crossover; DH: Doubled haploid; DSB: Double-strand break; GWRR: Genome-wide recombination rate; IBD: Identity-by-descent; NAM: Nested association mapping; NOR: Nucleolus organizer region; QTL: Quantitative trait loci; SNP: Single nucleotide polymorphism.

## Competing interests

The authors declare that they have no competing interests.

## Authors’ contributions

TA, AC, PF, AEM, MM, JMG, MO, PR, and CCS designed the project. TA, CB, CC, LC, AC, MF, PF, JMG, OCM, AEM, MM, MO, NR, PR, and WS developed the populations or contributed reagents or software tools. EB, CB, CC, PF, NM, NR, WS, and HW performed the experiments. EB, MF, OCM, and CCS designed the data analysis. EB, MF, OCM, RR, and HW analyzed the data. EB, MF, OCM, and CCS wrote the paper. All authors read and approved the manuscript.

## Supplementary Material

Additional file 1: Table S1 Maize lines used in this study, assignment to gene pools and origin of the lines. Click here for file

Additional file 2: Text S1 Supplementary results. Click here for file

Additional file 3: Table S2 Chromosome-wide genetic lengths and recombination rates for all genetic maps. Click here for file

Additional file 4: Table S3 Details of all genetic maps, including raw segregation data. Click here for file

Additional file 5: Figure S1 Allele frequency of the central parent allele for all polymorphic markers **(A)** in all Dent populations and **(B)** in all Flint populations. The 10 chromosomes are represented along the same horizontal axis. The expected frequency of 0.5 is indicated by a dotted line, and surrounded by solid lines representing its 99% confidence intervals. The x-axis indicates physical coordinates in megabase pairs along the B73 genome. In the bottom of the figure, the heat map represents gene density (low for cold colors and high for hot colors). Gray horizontal line below the heat-map: sketch of the chromosome organization showing centromeres (cen), knobs, nucleolar organizer region (NOR), and known gametophytic factors (ga_x_) (from [30]). Color filling of chromosome features is solid when the estimated boundaries of the region are known, and hatched when the box indicates only the extremities of the bin containing the region. Click here for file

Additional file 6: Figure S2 Statistical comparison of chromsome-wide recombination rates between the 23 populations of the experiment. Chromosome 'All' (first page) corresponds to the genome-wide analysis. 'DentAll', 'FlintAll', and 'All' correspond, respectively, to pooled analyses of all Dent × Dent populations, all Flint × Flint populations, and all 23 populations together. Dark blue, light blue, green, yellow, and red correspond respectively to *P* ≥ 5.10^-2^, 10^-3^ ≤ *P* < 10^-2^, 10^-4^ ≤ *P* < 10^-3^, 10^-5^ ≤ *P* < 10^-4^, *P* < 10^-5^ where *P* is the *P* value of the pairwise comparison test, corrected for multiple testing (Bonferroni). Arrows pointing to the right (respectively to the bottom) indicate that the cross listed in the vertical axis (respectively the horizontal axis) has a higher recombination rate than the cross listed in the horizontal axis (respectively the vertical axis). Dendrograms indicate hierarchical clustering of -log_10_(*P* value) based on Euclidian distances, and were used to order the populations. Click here for file

Additional file 7: Figure S3 Marey maps and recombination landscapes along the chromosomes for three examples illustrating the imputation of regions with missing or unreliable data: CFD01 chromosome 1, CFF07 chromosome 1, and CFF01 chromosome 3. The x-axis indicates physical position of the SNPs on the B73 physical map in megabase pairs. The left y-axis indicates genetic map position in centiMorgans. The right y-axis indicates recombination rate in cM/Mbp. Each black empty circle corresponds to a SNP. Red dots indicate the outlier markers removed from the smoothing analysis. Dark blue dotted line: smoothed Marey map. Red dotted line: first derivative of the smoothed Marey map. Hatched rectangles: regions masked when going from bare to masked Marey maps (see Materials and methods). Light blue solid line: imputed smoothed Marey map obtained after imputation in the excluded regions, using data from all maps pooled. Pink solid curve: recombination rate computed as the first derivative of the imputed smoothed Marey map. Click here for file

Additional file 8: Text S2 Detailed methods. Click here for file

Additional file 9: Figure S4 Recombination rates along maize chromosomes 1 to 10. The x-axis indicates physical position (Mbp) along chromosomes. The y-axis indicates genetic position (cM; top panel), recombination rate (cM/Mbp; middle panel), and pairwise parental similarity (frequency of identical SNP alleles in 10 Mbp sliding windows with a step size of 2 Mbp; bottom panel) for 6 of the 23 populations, after smoothing and imputation (see Materials and methods). Blue lines: Flint × Flint crosses. Red lines: Dent × Dent crosses. In both groups, the solid, dashed and dotted lines correspond to the population with the highest, median or lowest genome-wide recombination rate within its group, respectively. Gray parts of the lines correspond to regions where the information was missing or not reliable (IBD segments, non-colinearity with B73), and thus imputed from the other maps (see Materials and methods). Heat-maps below the curves of recombination rates indicate gene density (low for cold colors and high for hot colors). Gray horizontal line below the heat-map: sketch of the chromosome organization (from [30]) showing centromeres (cen), knobs, and nucleolar organizer region (NOR). Color filling of chromosome features is solid when the estimated boundaries of the region are known, and hatched when the box indicates only the extremities of the bin containing the region. Click here for file

Additional file 10: Figure S5 Statistical comparison of genome-wide recombination landscapes between the 23 populations for all chromosomes pooled as well as for each chromosome. For each pairwise comparison test, both genetic map lengths were normalized to their average value, so the test is not affected by differences in the global recombination rate but only by differences in the shape of the recombination landscape. 'DentAll', 'FlintAll', and 'All' correspond, respectively, to pooled analyses of all Dent × Dent populations, all Flint × Flint populations, and all 23 populations together. Dark blue, light blue, green, yellow, and red correspond respectively to *P* ≥ 5.10^-2^, 10^-3^ ≤ *P* < 10^-2^, 10^-4^ ≤ *P* < 10^-3^, 10^-5^ ≤ *P* < 10^-4^, *P* < 10^-5^ where *P* is the *P* value of the pairwise comparison test, corrected for multiple testing (Bonferroni). Dendrograms indicate hierarchical clustering of -log_10_(*P* value) based on Euclidian distances, and were used to order the populations. Click here for file

Additional file 11: Figure S6 Illustration of the statistical test used to compare recombination landscapes between the pooled data of all Dent × Dent (red) and Flint × Flint (blue) populations. Within regions excluded from the analysis for one population, the data were imputed from the other pool for conservativeness of the test (Additional file 8). Solid curves: Marey maps normalized to the average genetic length, so the comparison focuses on differences in the shape of the recombination landscapes and is not affected by differences in the values of chromosome genetic lengths. Dotted curves: first derivative of the normalized Marey maps, indicating the recombination landscape along the chromosome. The black rectangles show the 10 bins used for the analysis (from left to right: bins 1 to 10). Bin boundaries were chosen so each bin contained regions of the same genetic length. On top of each bar, a black vertical arrow indicates the difference between both populations in average recombination rates over the bin considered, and the error bars indicate the 95% confidence intervals of these average recombination rates. Click here for file

Additional file 12: Figure S7 Statistical comparisons of interference intensity in pathway P1 (*nu*) between individual populations, for all chromosomes pooled together. 'DentAll', 'FlintAll', and 'All' correspond, respectively, to pooled analyses of all Dent × Dent populations, all Flint × Flint populations, and all 23 populations together. Dark blue, light blue, green, yellow, and red correspond respectively to *P* ≥ 5.10^-2^, 10^-3^ ≤ *P* < 10^-2^, 10^-4^ ≤ *P* < 10^-3^, 10^-5^ ≤ *P* < 10^-4^, *P* < 10^-5^ where *P* is the *P* value of the pairwise comparison test, corrected for multiple testing (Bonferroni). Arrows pointing to the right (respectively to the bottom) indicate that the cross listed in the vertical axis (respectively the horizontal axis) has a higher value of *nu* than the cross listed in the horizontal axis (respectively the vertical axis). Dendrograms indicate hierarchical clustering of -log_10_(*P* value) based on Euclidian distances, and were used to order the populations. Click here for file

Additional file 13: Figure S8 Statistical comparisons between individual populations, of the fraction (*p*) of crossovers formed via the non-interfering pathway P2, for all chromosomes pooled together. 'DentAll', 'FlintAll', and 'All' correspond, respectively, to pooled analyses of all Dent × Dent populations, all Flint × Flint populations, and all 23 populations together. Dark blue, light blue, green, yellow, and red correspond respectively to *P* ≥ 5.10^-2^, 10^-3^ ≤ *P* < 10^-2^, 10^-4^ ≤ *P* < 10^-3^, 10^-5^ ≤ *P* < 10^-4^, *P* < 10^-5^ where *P* is the *P* value of the pairwise comparison test, corrected for multiple testing (Bonferroni). Arrows pointing to the right (respectively to the bottom) indicate that the cross listed in the vertical axis (respectively the horizontal axis) has a higher value of *p* than the cross listed in the horizontal axis (respectively the vertical axis). Dendrograms indicate hierarchical clustering of -log_10_(*P* value) based on Euclidian distances, and were used to order the populations. Click here for file

Additional file 14: Figure S9 Correlation between interference intensity in pathway P1 (*nu*) and genome-wide recombination rate for the 10 chromosomes pooled together over all populations. Click here for file

Additional file 15: Figure S10 Crossing scheme of the two Dent and Flint half-sib panels. In each panel, a central line was crossed to diverse founder lines and DH lines were developed from the resulting F_1_ plants using the *in vivo* haploid induction method. Dent lines are shown in red, Flint lines in blue. Red lines: Dent × Dent crosses. Blue lines: Flint × Flint crosses. Gray lines: Dent × Flint/Flint × Dent crosses. Two reciprocal crosses (CFD01, CFF01) connect the panels via the central lines F353 and UH007. Both panels are also connected by crossing the central parent to B73 (CFD02, CFF02). The panels consist of 11 CFD and 13 CFF full-sib families, respectively. Click here for file
