# Supplementary material for: Intraspecific variation of recombination rate in maize
Source: Genome Biol. 2013 Sep 19;14(9):R103. doi: 10.1186/gb-2013-14-9-r103 (PMC4053771; doi:10.1186/gb-2013-14-9-r103)
Supplement: Additional file 2: Text S1 — Supplementary results. [file gb-2013-14-9-r103-S2.pdf]

Additional file 2 (Text S1): Supplementary results

Diversity in the parental lines of this study

We examined how well the parents of our two panels of half-sib families reflect the diversity of European maize germplasm in the Dent and Flint pools. This can be shown by PCoA of around 300 Dent and 300 Flint lines, respectively, which were described earlier [1]. The lines were chosen to cover the diversity of European and American temperate maize. In the Dent panel the eleven lines are well distributed over the four main groups which were identified by Admixture analysis (Figure S11 A). Each of the four groups Stiff Stalk, Lancaster, Iodent and the Hohenheim Dent is represented by at least two lines. Similarly, in Flint the four main groups Northern Flint, Hohenheim Flint, Lacaune and the early Flint introductions are represented by the twelve Flint lines of the EU NAM panel (Figure S11 B).

For further characterizing the genetic diversity between the parental lines of the two panels, pairwise distances were calculated for all parental combinations using 23,188 SNP markers. The average pairwise Rogers' distance for all comparisons was 0.75. The average distances between the eleven Dent lines (0.73) and between the twelve Flint lines (0.72) were not significantly different. In the dendrogram, two major clades appeared which clearly separate Flint and Dent lines (Figure S12 A). Within Flint, the French and German lines including the central line UH007 formed one more closely related subgroup of Northern Flint and Hohenheim Flint lines. This subgroup also included the Spanish line EC49A, although this line is less related to the other members of this subgroup. A separate subgroup contained the two Spanish lines EZ5 and EP44, and line F64. In Dent, the three Hohenheim Dent lines D06, D09, and UH250 were most closely related and formed one subgroup. Next to it was the subgroup containing the central line F353 and line UH304, both Iodents, together with the French early dent line F252. The Stiff Stalk-related lines B73, EC169, and F618 also formed

a separate cluster. Two lines from the Lancaster and Minnesota 13 pools, Mo17 and W117, were grouped together. Overall, the grouping obtained in this analysis agrees well with results shown above from PCoA and with information on the origin or pedigree of the lines. If diversity was examined chromosome-wise, the clear differentiation between Flint and Dent was only retained on chromosomes 1 and 8 (Figure S12 B). On chromosome 4, the only exception to the expected clustering was Dent line W117 which grouped among the Flint lines, while the only exception on chromosome 5 was Flint line EC49A which moved to the Dent group. On all other chromosomes new patterns were found with admixed Flint-Dent groups. Some line pairs showed high similarity in the chromosome-wise analysis, e.g. D06 and D09 on chromosome 1, B73 and EC169 on chromosome 2, D06 and UH250 on chromosome 3 or the triple D06, D09 and UH250 on chromosome 8, which is an indication for common ancestry of the lines.

To visualize relatedness between central lines and founder lines of the Flint and Dent panels along the genome and to determine regions with low diversity, we performed a genome-scan for polymorphism in sliding windows of 10 Mbp length along all 10 chromosomes (Figure S13). Several regions were observed where founder lines lack polymorphism compared to their corresponding central lines, probably due to common ancestry (identity by descent, IBD). This was especially true in Flint where most of the German and French lines are closely related to UH007. In Dent on average there was more diversity of the founder lines compared to the central line F353 but IBD blocks also existed. Extended regions of low diversity usually correspond to the regions where gaps in the genetic maps were observed.

Comparing genetic maps with the B73 genome assembly v2

Synteny is conserved between two maps if markers assigned to the same chromosome in one map are also on the same chromosome in the other map. In the 23 genetic maps produced here, out of 39,439 mapped SNPs, only 76 distinct markers were found non-syntenic between one of the genetic maps and the B73 genome assembly (Table S4). The physical positions of all non-syntenic markers are indicated in Figure S14. For all 76 positions where a marker was found non-syntenic with B73 in at least one of the genetic maps, all other genetic maps containing this marker were also in disagreement with B73, and all genetic maps confirmed the same chromosome assignment of the marker (Figure S14), indicating the quality and consistency of the genetic maps. Elsewhere, 118 distinct markers had no chromosomal assignment in the B73 genome assembly, but were mapped in at least one of the genetic maps (Table S4). This information may help to further improve the assembly of B73.

Colinearity is conserved between a genetic map and the B73 physical map if chromosomal assignment *and* marker order are consistent between maps. In the 23 genetic maps studied here, a total of 362 regions were found non-colinear between one of the genetic maps and the B73 genome assembly. The physical positions of these regions are shown in Figure S15. In most cases, within such regions, the order of markers in the B73 genome was in disagreement with several genetic maps, whereas these orders were compatible between the genetic maps. All non-colinearities between genetic maps and the B73 genome are listed in Table S5, again pointing to good consistency amongst our genetic maps. The mean and median genetic lengths of segments where inconsistencies were observed were 13.0 and 3.1 cM, respectively, showing that the majority of inconsistencies affect rather short genetic map regions. We observed translocations in 223 cases and more complex rearrangements in 139 cases. Many of the rearrangements occur in several maps, as for example on chromosome 1S, 2L, 4L, 5L, 6L, 7L and 9L. Most of these regions were also identified earlier in independent mapping populations [2]. One region with small translocations on 1L occurs only

in maps involving Dent lines, it is not observed in Flint x Flint crosses. One cannot exclude that a small number of the non-colinearities are due to limited power in determining marker order in populations with small progeny size, although stringent order robustness threshold criteria were applied when constructing the genetic maps. However, most of the regions are confirmed in several maps and thus likely correspond to regions with either local errors in the B73 assembly or small structural variations in one or both parents of the mapping populations compared to B73.

References

1. Rincent R, Laloë D, Nicolas S, Altmann T, Brunel D, Revilla P, Rodriguez VM, Moreno-Gonzales J, Melchinger AE, Bauer E, Schön C-C, Meyer N, Giauffret C, Bauland C, Jamin P, Laborde J, Monod H, Flament P, Charcosset A, Moreau L: **Maximizing the reliability of genomic selection by optimizing the calibration set of reference individuals: comparison of methods in two diverse groups of maize inbreds (*Zea mays* L.).** *Genetics* 2012, **192:**715-728.
2. Ganal MW, Durstewitz G, Polley A, Berard A, Buckler ES, Charcosset A, Clarke JD, Graner EM, Hansen M, Joets J, Le Paslier MC, McMullen MD, Montalent P, Rose M, Schön CC, Sun Q, Walter H, Martin OC, Falque M: **A large maize (*Zea mays* L.) SNP genotyping array: development and germplasm genotyping, and genetic mapping to compare with the B73 reference genome.** *PLoS ONE* 2011, **6:**e28334.

List of additional figures:

Figure S11. Principal coordinate analysis of the distance matrix from two European diversity panels for Dent (A) and Flint (B). In both panels, the parental lines of the half-sib families are highlighted. The four major germplasm groups as identified by Admixture analysis are colour-coded in each panel.

Figure S12. (A) Dendrogram of 23 maize lines based on Rogers' distance using 23,186 informative SNPs with MAF > 0.05. (B) Dendrograms of 23 maize lines based on Rogers' distance calculated for each chromosome separately. The number of SNPs for each chromosome is indicated in brackets.

Figure S13. Pattern of genome-wide similarity in the Dent (A) and Flint (B) panel between the central parents F353 (A) and UH007 (B), respectively and the founder lines. Founder lines are sorted from top to bottom according to decreasing similarity with F353 or UH007, respectively. Similarity is visualized as proportion of identical SNP alleles within windows of 10 Mbp with a step size of 2 Mbp, displayed as heat maps along the chromosomes of each founder line. Positions of SNPs are based on the B73 genome sequence.

Figure S14. Position of markers which have non-syntenic chromosome assignments between the genetic maps and the B73 genome. Light grey bars represent the B73 genome assembly. Colored dots indicate positions of markers which are assigned to a different chromosome by the genetic map. Empty grey circles represent all markers which are monomorphic in a given population, but have non-syntenic chromosome assignments between the B73 genome and one of the genetic maps from other populations. Individual maps from left to right are: CFD01, 02, 03, 04, 05, 06, 07, 09, 10, 11, 12, CFF01, 02, 03, 04, 06, 07, 08, 09, 10, 12, 13, 15.

Figure S15. Physical location on the B73 genome, of the chromosomal regions in which the linkage maps are not colinear with the B73 genome. All individual maps are from left to right: CFD01, 02, 03, 04, 05, 06, 07, 09, 10, 11, 12, CFF01, 02, 03, 04, 06, 07, 08, 09, 10, 12, 13, 15. Blue lines represent intrachromosomal translocations. The thick blue segment indicates the region of the B73 genome assembly which is translocated to the position of the arrow head in the genetic map. Brown thick lines indicate complex non-colinearities composed of several nested translocations and/or inversions.

List of additional tables:

Table S4. List of all markers assigned to a different chromosome in the B73 genome assembly and in the genetic maps of this experiment.

Table S5. List of regions with non-colinear marker orders between the genetic maps and the B73 genome assembly.

Figure S11

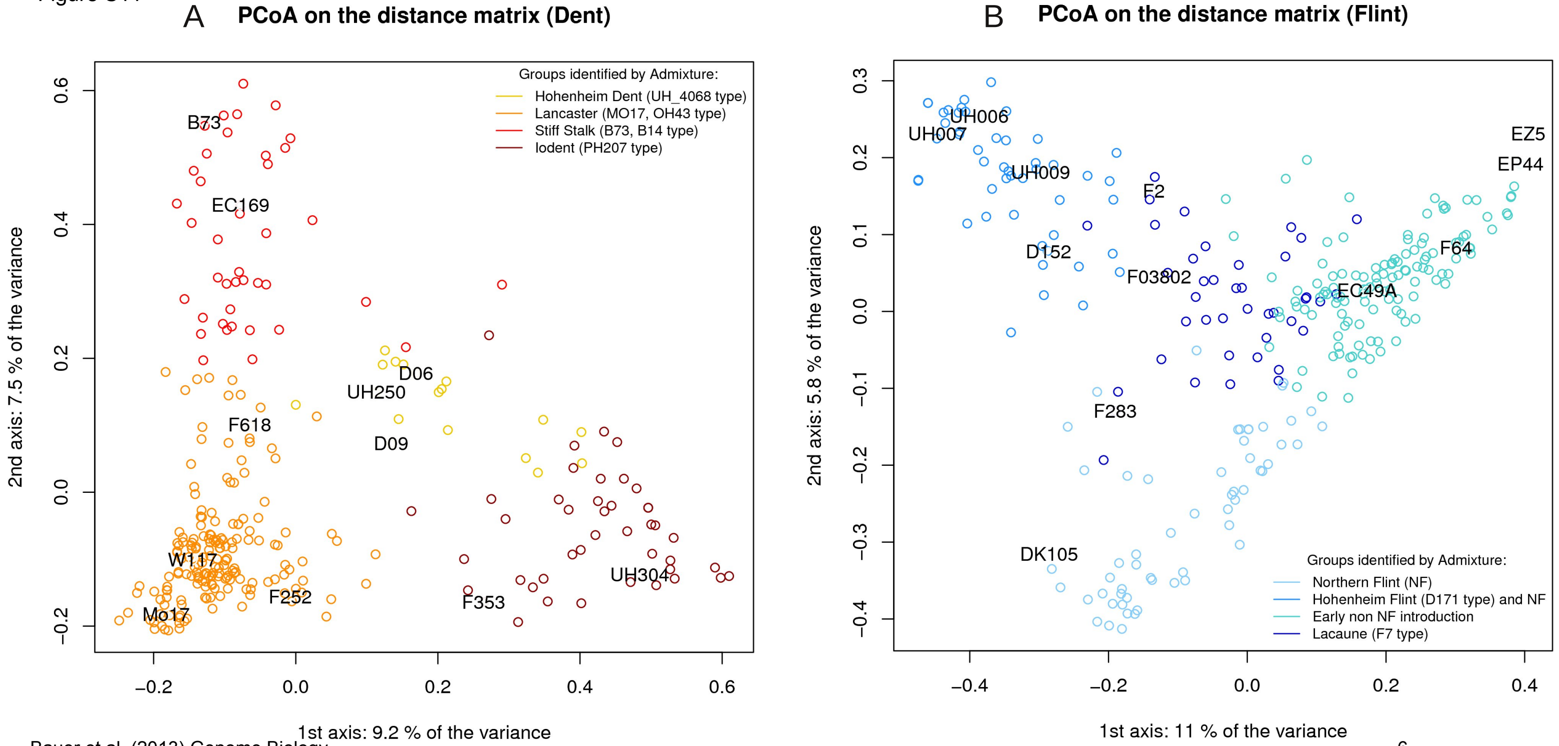

Bauer et al. (2013) Genome Biology

Figure S12

A

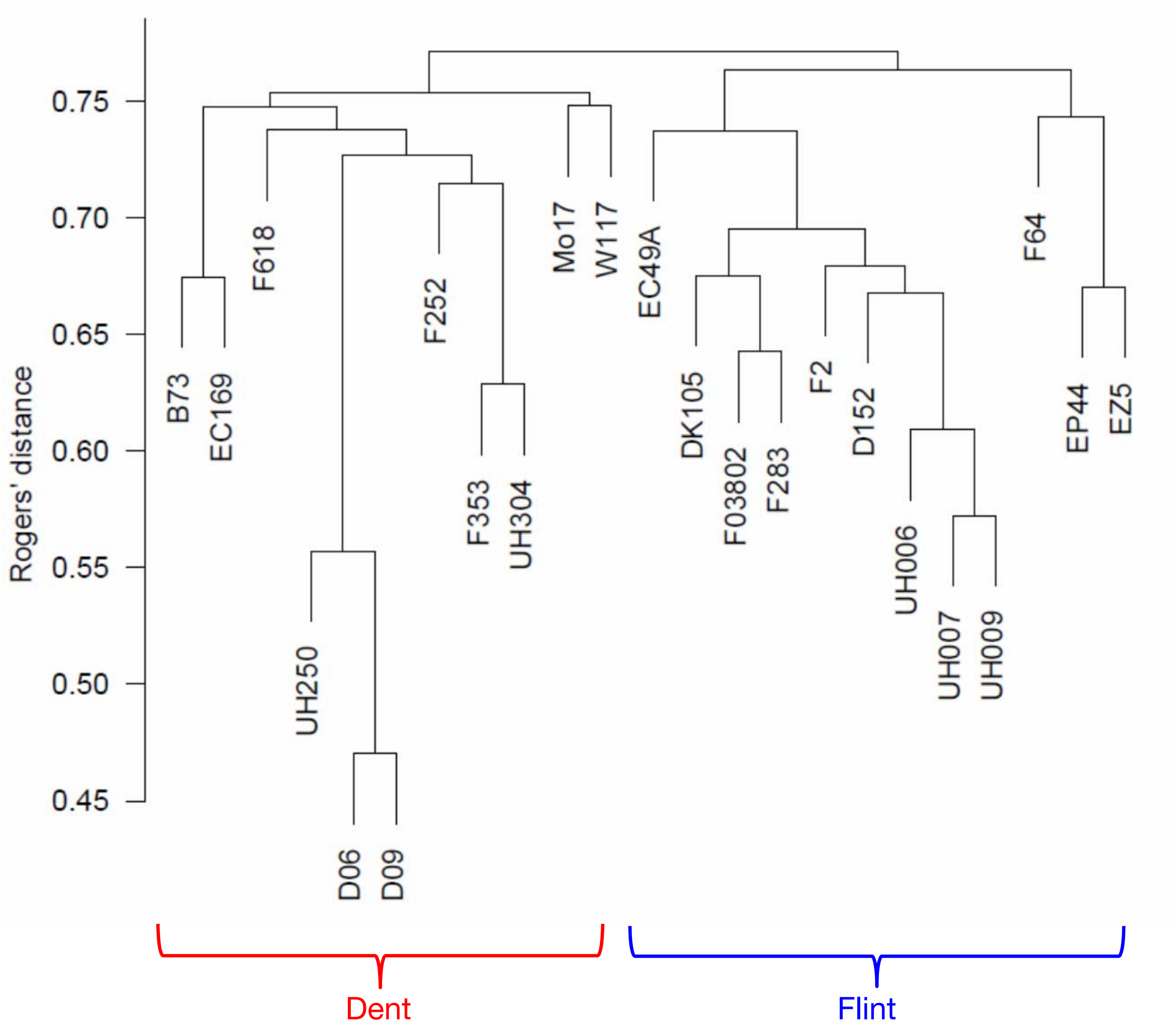

Rogers' distance
0.75
0.70
0.65
0.60
0.55
0.50
0.45
B73
EC169
F618
UH250
D06
D09
F252
F353
UH304
Mo17
W117
EC49A
DK105
F03802
F283
F2
D152
UH006
UH007
UH009
F64
EP44
EZ5
Dent
Flint

Figure S12

B

Chromosome 1 (3,501 SNPs)

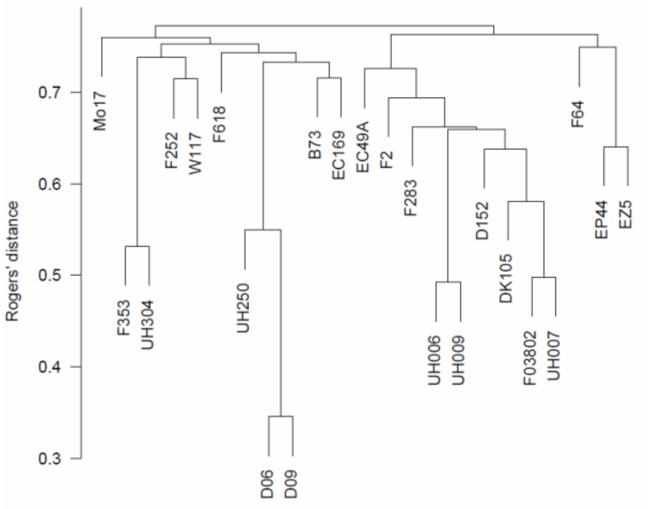

Chromosome 2 (2,576 SNPs)

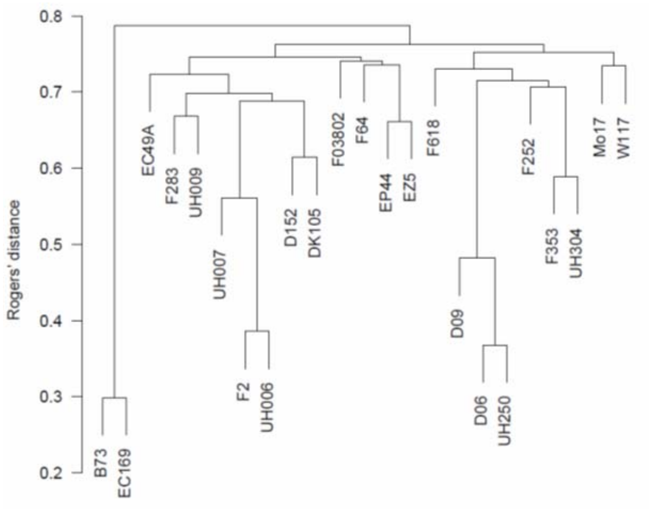

Chromosome 3 (2,621 SNPs)

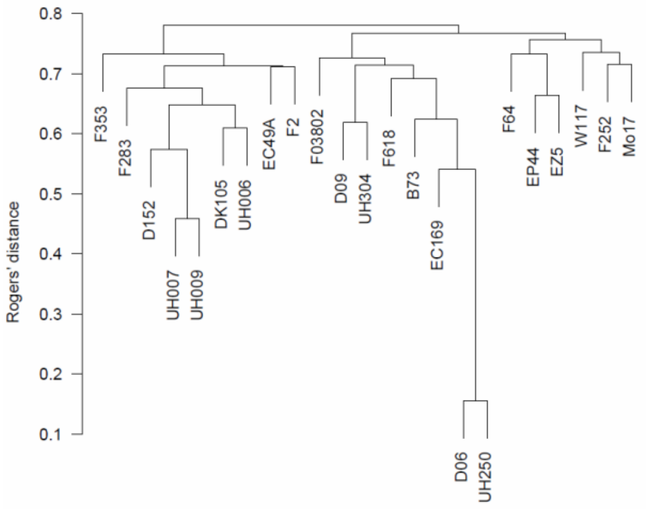

Chromosome 4 (2,611 SNPs)

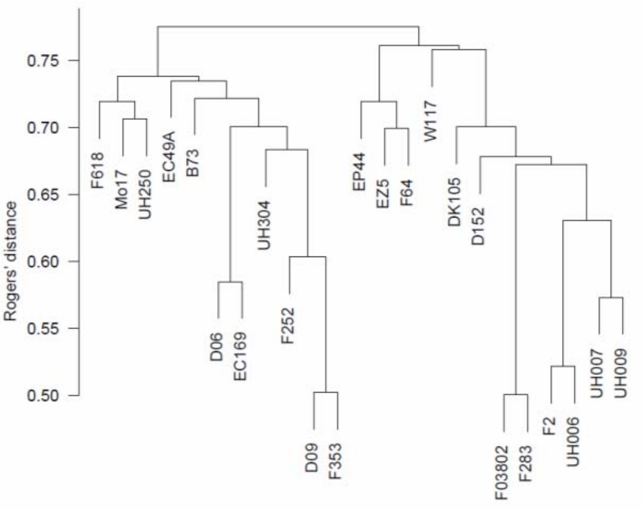

Chromosome 5 (2,534 SNPs)

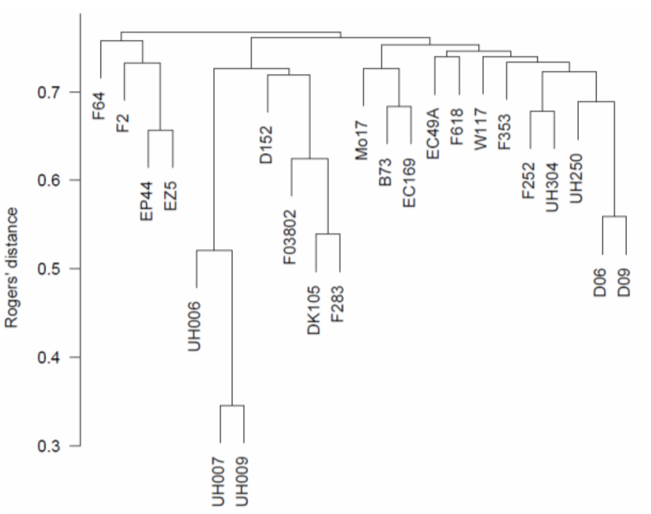

Chromosome 6 (1,769 SNPs)

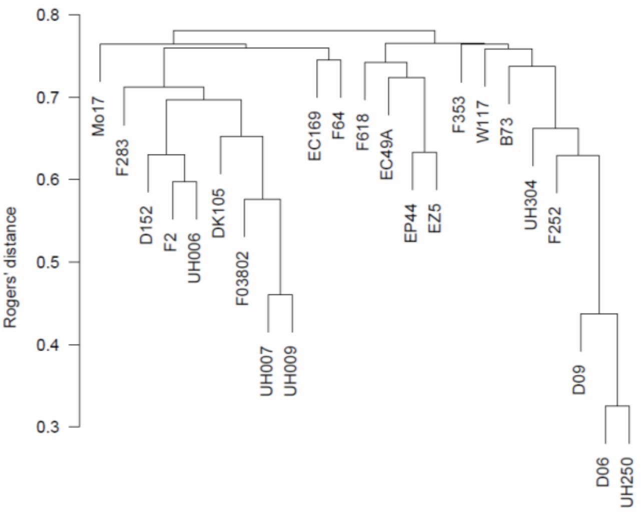

Figure S12

Chromosome 7 (1,905 SNPs)

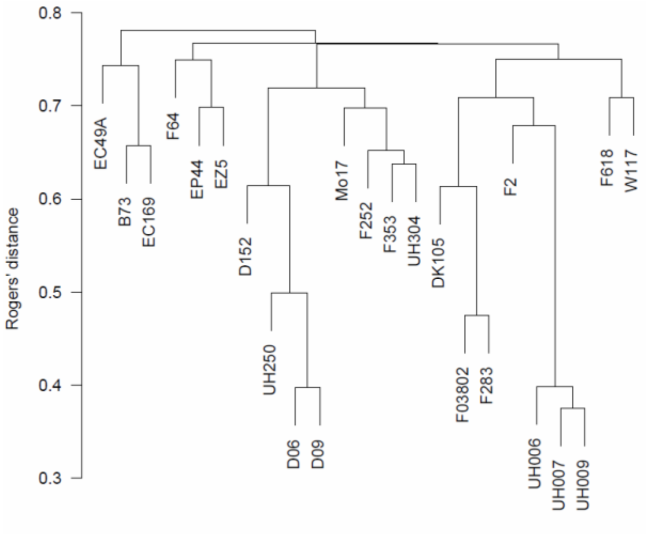

Chromosome 8 (2,116 SNPs)

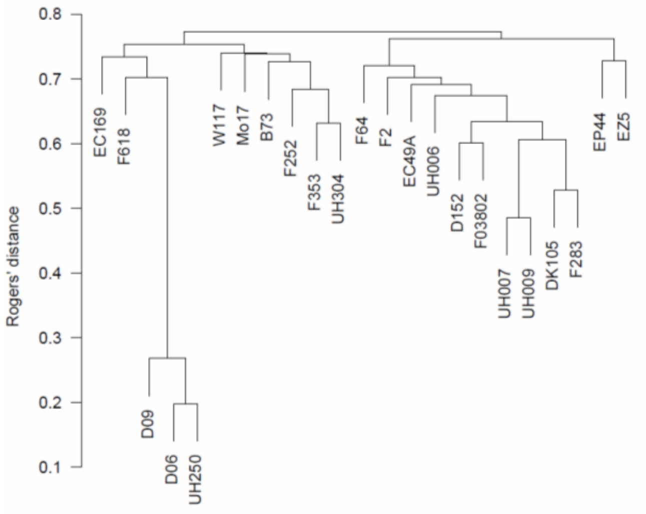

Chromosome 9 (1,779 SNPs)

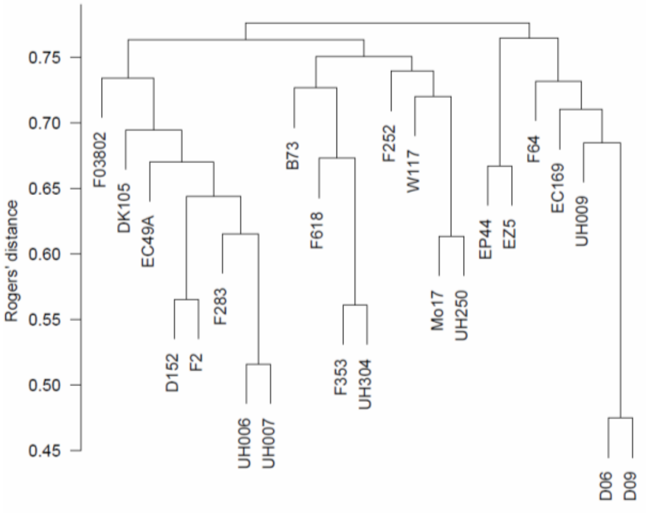

Chromosome 10 (1,679 SNPs)

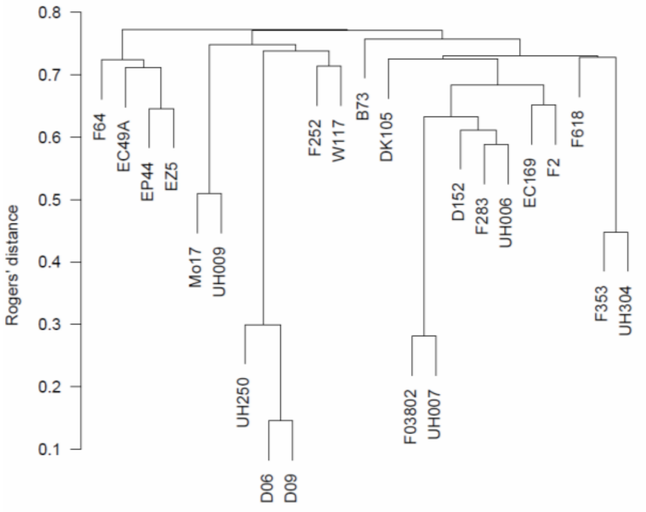

Figure S13

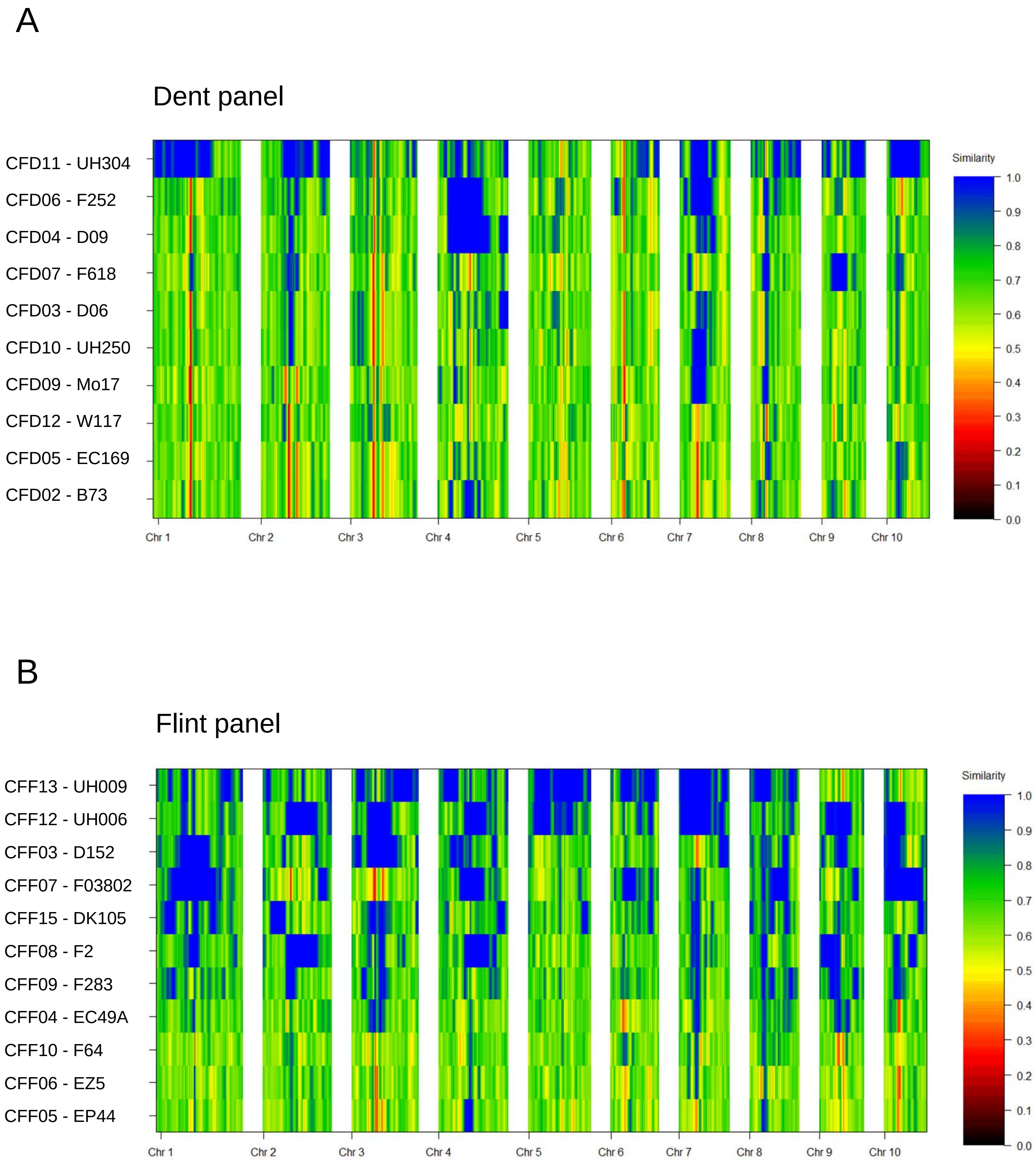
A
Dent panel
CFD11 - UH304
CFD06 - F252
CFD04 - D09
CFD07 - F618
CFD03 - D06
CFD10 - UH250
CFD09 - Mo17
CFD12 - W117
CFD05 - EC169
CFD02 - B73
Chr 1
Chr 2
Chr 3
Chr 4
Chr 5
Chr 6
Chr 7
Chr 8
Chr 9
Chr 10
Similarity
1.0
0.9
0.8
0.7
0.6
0.5
0.4
0.3
0.2
0.1
0.0
B
Flint panel
CFF13 - UH009
CFF12 - UH006
CFF03 - D152
CFF07 - F03802
CFF15 - DK105
CFF08 - F2
CFF09 - F283
CFF04 - EC49A
CFF10 - F64
CFF06 - EZ5
CFF05 - EP44
Chr 1
Chr 2
Chr 3
Chr 4
Chr 5
Chr 6
Chr 7
Chr 8
Chr 9
Chr 10
Similarity
1.0
0.9
0.8
0.7
0.6
0.5
0.4
0.3
0.2
0.1
0.0

Figure S14

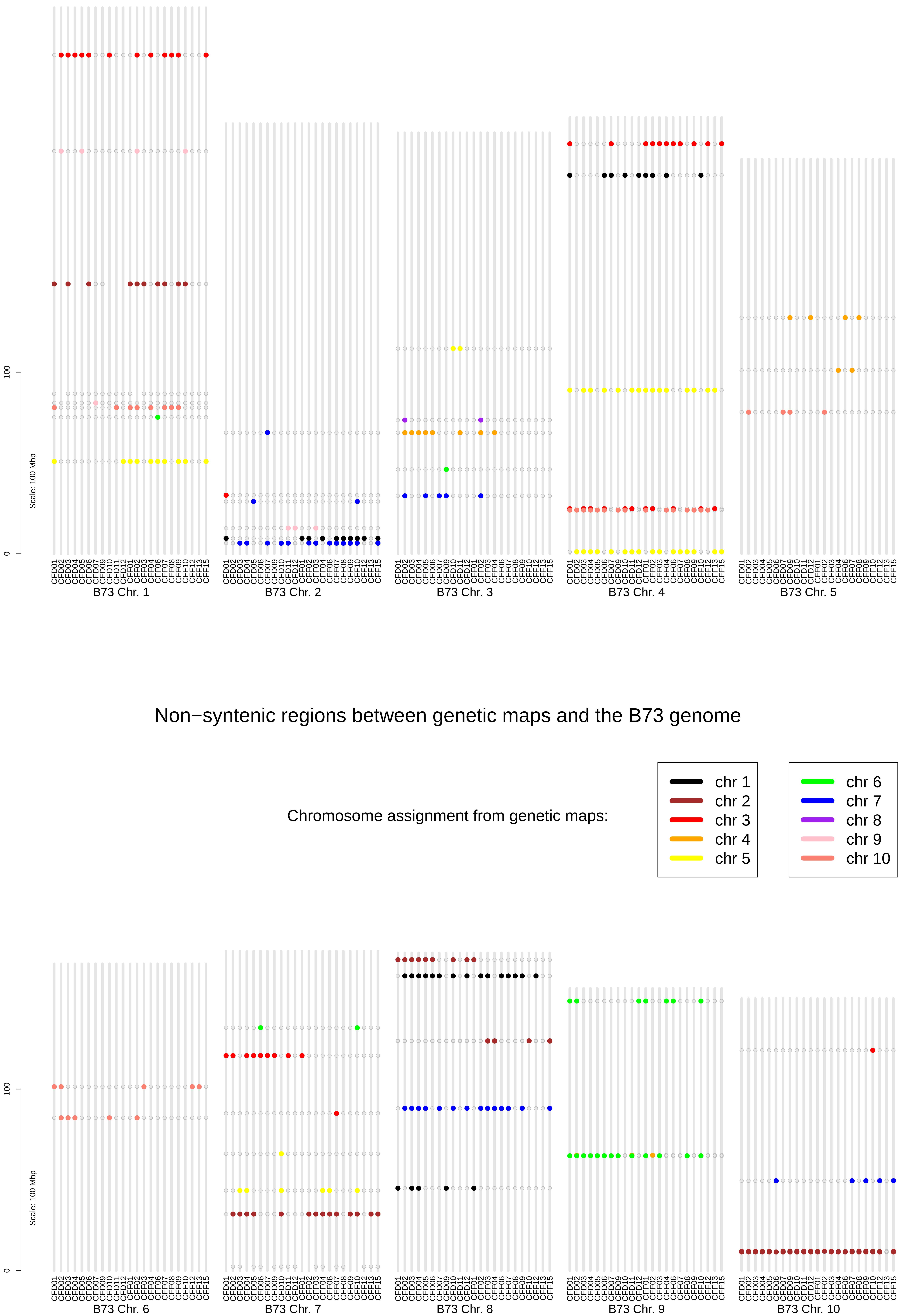
100
0
Scale: 100 Mbp
CFD01 CFD02 CFD03 CFD04 CFD05 CFD06 CFD07 CFD09 CFD10 CFD11 CFD12 CFF01 CFF02 CFF03 CFF04 CFF06 CFF07 CFF08 CFF09 CFF10 CFF12 CFF13 CFF15
B73 Chr. 1
B73 Chr. 2
B73 Chr. 3
B73 Chr. 4
B73 Chr. 5
Non−syntenic regions between genetic maps and the B73 genome
Chromosome assignment from genetic maps:
chr 1
chr 2
chr 3
chr 4
chr 5
chr 6
chr 7
chr 8
chr 9
chr 10
B73 Chr. 6
B73 Chr. 7
B73 Chr. 8
B73 Chr. 9
B73 Chr. 10

Figure S15

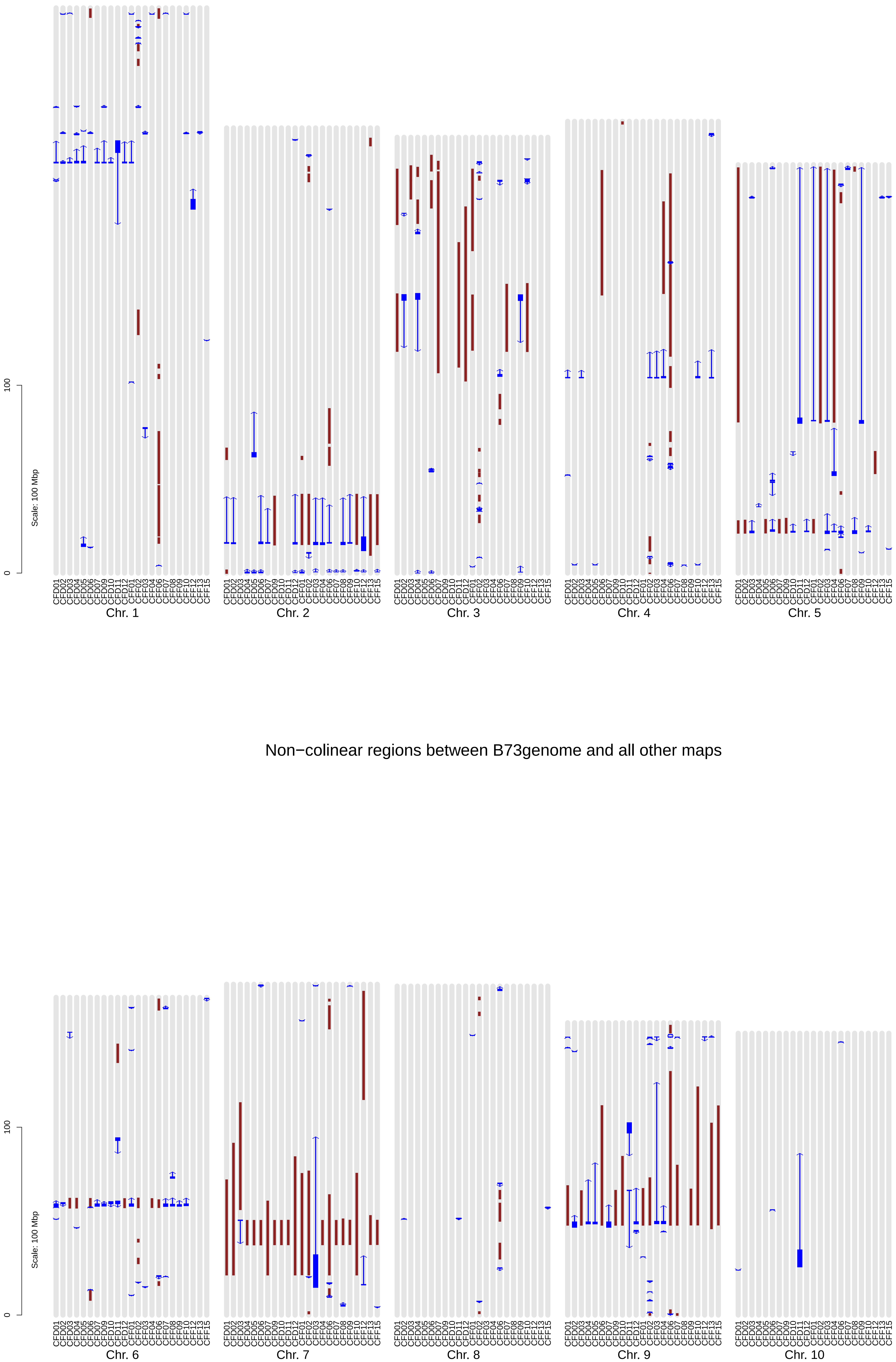
Non−colinear regions between B73genome and all other maps
Scale: 100 Mbp
0
100
CFD01
CFD02
CFD03
CFD04
CFD05
CFD06
CFD07
CFD09
CFD10
CFD11
CFD12
CFF01
CFF02
CFF03
CFF04
CFF06
CFF07
CFF08
CFF09
CFF10
CFF12
CFF13
CFF15
Chr. 1
Chr. 2
Chr. 3
Chr. 4
Chr. 5
Chr. 6
Chr. 7
Chr. 8
Chr. 9
Chr. 10

Table S4

Mapped markers that were non-syntenic with B73.
chr_gen: chromosomal assignment in the genetic map
chr_phy: chromosomal assignment in the B73 assembly
gen_pos: position of marker in the map in cM

map	chr_gen	chr_phy	SNP_name	gen_pos	coordinate
CFF15	1	2	PZE-102184787	9.6	228578509
CFF04	1	2	PZE-102184787	15.8	228578509
CFF02	1	2	PZE-102184787	19.7	228578509
CFF01	1	2	PZE-102184787	19.9	228578509
CFF08	1	2	PZE-102184787	25.9	228578509
CFF07	1	2	PZE-102184787	26.4	228578509
CFF10	1	2	PZE-102184787	26.4	228578509
CFD01	1	2	PZE-102184787	26.6	228578509
CFF12	1	2	PZE-102184787	31.4	228578509
CFD05	1	8	PZE-101046118	35.9	12726804
CFF09	1	2	PZE-102184787	37.4	228578509
CFD03	1	8	PZE-101046118	38	12726804
CFD03	1	8	PZE-108074351	43	129693845
CFD10	1	8	PZE-101046118	43.3	12726804
CFD02	1	8	PZE-101046118	43.7	12726804
CFD12	1	8	PZE-101046118	45.4	12726804
CFD07	1	8	PZE-101046118	47.4	12726804
CFF08	1	8	PZE-101046118	47.4	12726804
CFD09	1	8	PZE-108074351	50	129693845
CFD04	1	8	PZE-101046118	55.8	12726804
CFF12	1	8	PZE-101046118	56.6	12726804
CFD06	1	8	PZE-101046118	59.5	12726804
CFD01	1	8	PZE-108074351	60.4	129693845
CFF09	1	8	PZE-101046118	60.7	12726804
CFF07	1	8	PZE-101046118	60.8	12726804
CFF01	1	8	PZE-108074351	63.1	129693845
CFF02	1	8	PZE-101046118	63.2	12726804
CFD04	1	8	PZE-108074351	65.6	129693845
CFF03	1	8	PZE-101046118	68.6	12726804
CFD10	1	4	PZE-101146702	72.3	31998398
CFF06	1	8	PZE-101046118	84.1	12726804
CFD07	1	4	PZE-101146702	103.4	31998398
CFD12	1	4	PZE-101146702	106	31998398
CFD06	1	4	PZE-101146702	107.8	31998398
CFF04	1	4	PZE-101146702	113.5	31998398
CFD01	1	4	PZE-101146702	115.6	31998398
CFF10	1	4	PZE-101146702	116.4	31998398
CFF01	1	4	PZE-101146702	126.4	31998398
CFF02	1	4	PZE-101146702	130.4	31998398
CFF13	2	7	SYN29313	32.2	144926689
CFD05	2	8	SYN34975	35.8	3769699
CFD04	2	10	PZE-110089707	37.3	139321667
CFD04	2	10	PZE-110090126	37.3	139740144

CFD04	2	10	PZE-110090193	37.3	139745681
CFD04	2	8	SYN34975	37.3	3769699
CFD04	2	1	SYN9944	37.3	152261893
CFD04	2	1	SYN9946	37.3	152262536
CFD04	2	1	SYN9949	37.3	152261832
CFD04	2	1	SYN9950	37.3	152262332
CFD05	2	10	PZE-110089707	37.4	139321667
CFD05	2	10	PZE-110090126	37.4	139740144
CFD05	2	10	PZE-110090193	37.4	139745681
CFD09	2	10	PZA03501.1	38.4	139316531
CFD09	2	10	PZE-110089707	38.4	139321667
CFD09	2	10	PZE-110090075	38.4	139735577
CFD09	2	10	PZE-110090076	38.4	139735606
CFD09	2	10	PZE-110090126	38.4	139740144
CFD09	2	10	PZE-110090205	38.4	139746408
CFD12	2	10	PZA03501.1	38.7	139316531
CFD12	2	10	PZE-110089655	38.7	139319733
CFD12	2	10	PZE-110090052	38.7	139734801
CFD12	2	10	PZE-110090075	38.7	139735577
CFD12	2	10	PZE-110090076	38.7	139735606
CFD12	2	10	PZE-110090126	38.7	139740144
CFD12	2	10	PZE-110090205	38.7	139746408
CFD12	2	8	SYN34975	38.7	3769699
CFD03	2	8	SYN34975	39	3769699
CFD12	2	1	SYN9944	39.7	152261893
CFD12	2	1	SYN9946	39.7	152262536
CFD12	2	1	SYN9949	39.7	152261832
CFD12	2	1	SYN9950	39.7	152262332
CFD03	2	10	PZA03501.1	40	139316531
CFD03	2	10	PZE-110089645	40	139318533
CFD03	2	10	PZE-110089655	40	139319733
CFD03	2	10	PZE-110090052	40	139734801
CFD03	2	10	PZE-110090075	40	139735577
CFD03	2	10	PZE-110090076	40	139735606
CFD03	2	10	PZE-110090126	40	139740144
CFD03	2	10	PZE-110090205	40	139746408
CFF02	2	10	PZA03501.1	40	139316531
CFF02	2	10	PZE-110089537	40	139312924
CFF02	2	10	PZE-110089655	40	139319733
CFF02	2	10	PZE-110089707	40	139321667
CFF02	2	10	PZE-110089645	40.4	139318533
CFF02	2	10	PZE-110090052	40.4	139734801
CFF02	2	10	PZE-110090075	40.4	139735577
CFF02	2	10	PZE-110090076	40.4	139735606
CFD07	2	10	PZE-110090126	40.5	139740144
CFD07	2	10	PZE-110090205	40.5	139746408
CFD06	2	8	SYN34975	40.8	3769699
CFD10	2	8	SYN34975	41.2	3769699
CFD07	2	10	PZE-110090052	41.5	139734801
CFD07	2	10	PZE-110090075	41.5	139735577
CFD07	2	10	PZE-110090076	41.5	139735606

CFF04	2	10	PZE-110089816	41.6	139502648
CFF02	2	1	SYN9946	41.7	152262536
CFF04	2	10	PZE-110090052	41.7	139734801
CFF04	2	10	PZE-110090075	41.7	139735577
CFF04	2	10	PZE-110090076	41.7	139735606
CFF04	2	10	PZE-110090193	41.7	139745681
CFD03	2	1	SYN9944	42	152261893
CFD03	2	1	SYN9949	42	152261832
CFD03	2	1	SYN9950	42	152262332
CFD10	2	10	PZE-110089707	42.2	139321667
CFD10	2	10	PZE-110090126	42.2	139740144
CFD10	2	10	PZE-110090193	42.2	139745681
CFD10	2	1	SYN9944	42.2	152261893
CFD10	2	1	SYN9946	42.2	152262536
CFD10	2	1	SYN9949	42.2	152261832
CFD10	2	1	SYN9950	42.2	152262332
CFD05	2	1	SYN9944	42.3	152261893
CFD05	2	1	SYN9946	42.3	152262536
CFD05	2	1	SYN9949	42.3	152261832
CFD05	2	1	SYN9950	42.3	152262332
CFD07	2	10	PZA03501.1	42.4	139316531
CFD07	2	10	PZE-110089645	42.4	139318533
CFD07	2	10	PZE-110089655	42.4	139319733
CFD06	2	10	PZE-110090052	42.7	139734801
CFD06	2	10	PZE-110090075	42.7	139735577
CFD06	2	10	PZE-110090076	42.7	139735606
CFD06	2	10	PZE-110090126	42.7	139740144
CFD06	2	10	PZE-110090205	42.7	139746408
CFD11	2	10	PZA03501.1	42.7	139316531
CFD11	2	10	PZE-110089655	42.7	139319733
CFD11	2	10	PZE-110090052	42.7	139734801
CFD11	2	10	PZE-110090075	42.7	139735577
CFD11	2	10	PZE-110090076	42.7	139735606
CFD11	2	10	PZE-110090126	42.7	139740144
CFD11	2	10	PZE-110090205	42.7	139746408
CFD11	2	1	SYN9944	42.7	152261893
CFD11	2	1	SYN9946	42.7	152262536
CFD11	2	1	SYN9949	42.7	152261832
CFD11	2	1	SYN9950	42.7	152262332
CFD02	2	10	PZE-110089707	42.8	139321667
CFD02	2	10	PZE-110090126	42.8	139740144
CFD02	2	10	PZE-110090193	42.8	139745681
CFD02	2	8	SYN34975	42.8	3769699
CFD02	2	1	SYN9944	42.8	152261893
CFD02	2	1	SYN9946	42.8	152262536
CFD02	2	1	SYN9949	42.8	152261832
CFD02	2	1	SYN9950	42.8	152262332
CFF01	2	8	SYN34975	43.6	3769699
CFD06	2	1	SYN9944	43.7	152261893
CFD06	2	1	SYN9949	43.7	152261832
CFD06	2	1	SYN9950	43.7	152262332

CFF03	2	10	PZE-110090193	44	139745681
CFF03	2	10	PZE-110090205	44	139746408
CFD01	2	10	PZA03501.1	44.2	139316531
CFD01	2	10	PZE-110089645	44.2	139318533
CFD01	2	10	PZE-110089655	44.2	139319733
CFD01	2	10	PZE-110090052	44.2	139734801
CFD01	2	10	PZE-110090076	44.2	139735606
CFD01	2	10	PZE-110090126	44.2	139740144
CFD01	2	10	PZE-110090193	44.2	139745681
CFD01	2	8	SYN34975	44.2	3769699
CFD01	2	1	SYN9944	44.2	152261893
CFD01	2	1	SYN9949	44.2	152261832
CFD01	2	1	SYN9950	44.2	152262332
CFF01	2	10	PZE-110090052	44.6	139734801
CFF01	2	10	PZE-110090076	44.6	139735606
CFF01	2	10	PZE-110090126	44.6	139740144
CFF01	2	10	PZE-110090193	44.6	139745681
CFF03	2	10	PZE-110089752	44.9	139325492
CFF03	2	10	PZE-110090075	44.9	139735577
CFF03	2	1	SYN9946	44.9	152262536
CFF12	2	10	PZE-110090052	45.3	139734801
CFF12	2	10	PZE-110090075	45.3	139735577
CFF12	2	10	PZE-110090076	45.3	139735606
CFF12	2	10	PZE-110090126	45.3	139740144
CFF12	2	10	PZE-110090193	45.3	139745681
CFF01	2	10	PZA03501.1	45.7	139316531
CFF01	2	10	PZE-110089645	45.7	139318533
CFF01	2	10	PZE-110089655	45.7	139319733
CFF01	2	1	SYN9944	45.7	152261893
CFF01	2	1	SYN9949	45.7	152261832
CFF01	2	1	SYN9950	45.7	152262332
CFF12	2	10	PZE-110089816	46.6	139502648
CFF10	2	10	PZE-110089752	48.5	139325492
CFF10	2	10	PZE-110090075	48.5	139735577
CFF10	2	10	PZE-110090193	48.5	139745681
CFF10	2	10	PZE-110090205	48.5	139746408
CFF10	2	1	SYN9950	48.5	152262332
CFF06	2	10	PZE-110090052	48.9	139734801
CFF06	2	10	PZE-110090075	48.9	139735577
CFF06	2	10	PZE-110090076	48.9	139735606
CFF06	2	10	PZE-110090193	48.9	139745681
CFF07	2	10	PZE-110090075	49.1	139735577
CFF07	2	10	PZE-110090193	49.1	139745681
CFF07	2	10	PZE-110090205	49.1	139746408
CFF06	2	10	PZA03501.1	51.2	139316531
CFF06	2	10	PZE-110089645	51.2	139318533
CFF06	2	10	PZE-110089816	51.2	139502648
CFF06	2	1	SYN9946	51.2	152262536
CFF07	2	10	PZE-110089752	51.4	139325492
CFF08	2	10	PZE-110089645	52.8	139318533
CFF08	2	10	PZE-110089752	52.8	139325492

CFF08	2	10	PZE-110090052	52.8	139734801
CFF08	2	10	PZE-110090075	52.8	139735577
CFF08	2	10	PZE-110090076	52.8	139735606
CFF08	2	10	PZE-110090193	52.8	139745681
CFF07	2	1	SYN9950	53.9	152262332
CFF09	2	10	PZE-110089752	54.3	139325492
CFF09	2	10	PZE-110090075	54.3	139735577
CFF09	2	10	PZE-110090193	54.3	139745681
CFF09	2	10	PZE-110090205	54.3	139746408
CFF09	2	1	SYN9946	54.3	152262536
CFF15	2	10	PZA03501.1	55.4	139316531
CFF15	2	10	PZE-110089645	55.4	139318533
CFF15	2	10	PZE-110089752	55.4	139325492
CFF15	2	10	PZE-110090052	55.4	139734801
CFF15	2	10	PZE-110090075	55.4	139735577
CFF15	2	10	PZE-110090076	55.4	139735606
CFF15	2	10	PZE-110090193	55.4	139745681
CFD05	2	7	SYN29313	58	144926689
CFD10	2	7	SYN29313	60.3	144926689
CFD03	2	7	SYN29313	64	144926689
CFD02	2	7	SYN29313	69.7	144926689
CFF02	2	7	SYN29313	76.2	144926689
CFD04	2	7	SYN29313	76.6	144926689
CFF09	2	7	SYN29313	81.5	144926689
CFF04	2	8	PZE-108045064	81.6	48490428
CFF04	2	8	PZE-108045073	81.6	48507136
CFF04	2	8	PZE-108045079	81.6	48508272
CFF04	2	8	PZE-108045086	81.6	48509098
CFF04	2	8	PZE-108045120	81.6	48631594
CFF04	2	8	SYN10111	81.6	48508449
CFF04	2	7	SYN29313	81.6	144926689
CFF10	2	8	PZE-108045064	81.8	48490428
CFF10	2	8	PZE-108045073	81.8	48507136
CFF10	2	8	PZE-108045079	81.8	48508272
CFF10	2	8	PZE-108045086	81.8	48509098
CFF10	2	8	SYN10111	81.8	48508449
CFF10	2	7	SYN29313	83	144926689
CFF03	2	8	PZE-108045064	85.1	48490428
CFF03	2	8	PZE-108045073	85.1	48507136
CFF03	2	8	PZE-108045079	85.1	48508272
CFF03	2	8	PZE-108045086	85.1	48509098
CFF03	2	8	PZE-108045102	85.1	48555129
CFF03	2	8	PZE-108045120	85.1	48631594
CFF03	2	8	SYN10111	85.1	48508449
CFF03	2	7	SYN29313	85.1	144926689
CFF15	2	8	PZE-108045064	93.7	48490428
CFF15	2	8	PZE-108045073	93.7	48507136
CFF15	2	8	PZE-108045079	93.7	48508272
CFF15	2	8	PZE-108045086	93.7	48509098
CFF15	2	8	PZE-108045120	93.7	48631594
CFF15	2	8	SYN10111	93.7	48508449

CFF15	2	7	SYN29313	94.5	144926689
CFF07	2	7	SYN29313	95.6	144926689
CFF06	2	7	SYN29313	95.7	144926689
CFD04	3	7	PZB01944.3	20.7	57627393
CFD04	3	4	PZE-104132531	24.7	215686126
CFD05	3	7	PZB01944.3	26	57627393
CFD11	3	7	PZB01944.3	30.4	57627393
CFF01	3	7	PZB01944.3	31.2	57627393
CFD07	3	7	PZB01944.3	31.8	57627393
CFD09	3	7	PZB01944.3	33.5	57627393
CFD06	3	7	PZB01944.3	35.5	57627393
CFF13	3	4	PZE-104132531	38.7	215686126
CFD02	3	7	PZB01944.3	38.9	57627393
CFF01	3	4	PZE-104132531	39	215686126
CFF07	3	7	PZE-107044619	40.3	89425882
CFF06	3	4	PZE-104132531	40.8	215686126
CFF02	3	4	PZE-104132531	40.9	215686126
CFD01	3	7	PZB01944.3	41.7	57627393
CFD06	3	4	PZE-104132531	42.5	215686126
CFD03	3	4	PZE-104132531	47.1	215686126
CFD11	3	4	PZE-104132531	47.2	215686126
CFD01	3	4	PZE-104132531	50.4	215686126
CFF10	3	4	PZE-104132531	51.1	215686126
CFD10	3	4	PZE-104132531	51.3	215686126
CFD01	3	2	SYN15096	59.9	204787657
CFD05	3	1	PZB02510.5	68.8	26129082
CFF12	3	4	PZE-103109317	74.3	14611372
CFF04	3	4	PZE-103109317	77.6	14611372
CFF03	3	4	PZE-103109317	77.8	14611372
CFD07	3	4	PZE-103109317	80.5	14611372
CFF01	3	4	PZE-103109317	81.3	14611372
CFF02	3	4	PZE-103109317	83.6	14611372
CFD04	3	1	PZB02510.5	84.3	26129082
CFF09	3	4	PZE-103109317	84.4	14611372
CFF04	3	1	PZB02510.5	85.6	26129082
CFF07	3	4	PZE-103109317	86.7	14611372
CFD06	3	1	PZB02510.5	89.6	26129082
CFD01	3	4	PZE-103109317	91.5	14611372
CFF06	3	4	PZE-103109317	92.1	14611372
CFF15	3	4	PZE-103109317	92.7	14611372
CFD03	3	1	PZB02510.5	95.2	26129082
CFD10	3	1	PZB02510.5	96.9	26129082
CFF08	3	1	PZB02510.5	100.8	26129082
CFD02	3	1	PZB02510.5	102.2	26129082
CFF09	3	1	PZB02510.5	103.8	26129082
CFF07	3	1	PZB02510.5	111.5	26129082
CFF02	3	1	PZB02510.5	112.5	26129082
CFF10	3	10	PZA03171.3	117.8	28603338
CFF15	3	1	PZB02510.5	119	26129082
CFD02	4	9	PZA00726.8	36	91996108
CFD11	4	9	PZA00726.8	50	91996108

CFF02	4	9	PZA00726.8	60.8	91996108
CFD02	4	3	PZE-104112743	65.7	165323001
CFD05	4	3	PZE-104112743	83.2	165323001
CFD03	4	3	PZE-104112743	88.3	165323001
CFD11	4	3	PZE-104112743	98.1	165323001
CFD04	4	3	PZE-104112743	98.6	165323001
CFD06	4	3	PZE-104112743	107.4	165323001
CFF04	4	3	PZE-104112743	116.8	165323001
CFD12	4	5	PUT-163a-149095258-897	121.4	87311971
CFD09	4	5	PUT-163a-149095258-897	122.4	87311971
CFF02	4	3	PZE-104112743	123.8	165323001
CFF04	4	5	SYNGENTA12599	126.6	116347844
CFF07	4	5	SYNGENTA12599	130.9	116347844
CFF08	4	5	PUT-163a-149095258-897	143	87311971
CFF06	4	5	PUT-163a-149095258-897	154.2	87311971
CFF04	5	1	PUT-163a-148942702-461	40.7	250067064
CFD12	5	1	PUT-163a-148942702-461	41.8	250067064
CFF01	5	1	PUT-163a-148942702-461	44.8	250067064
CFF10	5	1	PUT-163a-148942702-461	47.2	250067064
CFF15	5	1	PUT-163a-148942702-461	47.9	250067064
CFF02	5	1	PUT-163a-148942702-461	51.8	250067064
CFD01	5	1	PUT-163a-148942702-461	51.9	250067064
CFD05	5	4	PZA02862.10	55.8	239413639
CFD12	5	4	PZA02862.10	55.8	239413639
CFD11	5	4	PZA02862.10	57.7	239413639
CFD03	5	4	PZA02862.10	58.3	239413639
CFD03	5	7	PZE-105088747	58.3	131964472
CFF09	5	1	PUT-163a-148942702-461	59.5	250067064
CFD07	5	4	PZA02862.10	60	239413639
CFF07	5	1	PUT-163a-148942702-461	62.1	250067064
CFF06	5	1	PUT-163a-148942702-461	62.6	250067064
CFD10	5	7	PZE-107058114	63.4	111705493
CFD10	5	4	PZA02862.10	64.4	239413639
CFD10	5	7	PZE-105088747	64.4	131964472
CFF08	5	4	PZA02862.10	65.5	239413639
CFF10	5	7	PZE-105088747	66.9	131964472
CFF02	5	4	PZA02862.10	69	239413639
CFD04	5	4	PZA02862.10	69.7	239413639
CFD02	5	4	PZA02862.10	70.5	239413639
CFD04	5	7	PZE-105088747	70.7	131964472
CFF13	5	4	PZA02862.10	71.3	239413639
CFD10	5	3	PZB01689.2	73.5	118940226
CFF04	5	7	PZE-105088747	74.1	131964472
CFF15	5	4	PZA02862.10	74.1	239413639
CFF09	5	4	PZA02862.10	75.3	239413639
CFD09	5	4	PZA03317.1	76	150406740
CFD03	5	4	PZA03317.1	76.3	150406740
CFD11	5	3	PZB01689.2	82.9	118940226
CFF07	5	4	PZA02862.10	83.7	239413639
CFF06	5	4	PZA02862.10	84	239413639
CFD12	5	4	PZA03317.1	90	150406740

CFD11	5	4	PZA03317.1	90.1	150406740
CFF03	5	4	PZA02862.10	96.4	239413639
CFF06	5	7	PZE-105088747	96.8	131964472
CFD04	5	4	PZA03317.1	98.1	150406740
CFD06	5	4	PZA03317.1	98.4	150406740
CFD01	5	4	PZA03317.1	103.5	150406740
CFF02	5	4	PZA03317.1	107.1	150406740
CFF01	5	4	PZA03317.1	108.4	150406740
CFF04	5	4	PZA03317.1	108.7	150406740
CFF08	5	4	PZA03317.1	109.5	150406740
CFF09	5	4	PZA03317.1	109.7	150406740
CFF13	5	4	PZA03317.1	118.5	150406740
CFF03	5	4	PZA03317.1	139.1	150406740
CFF12	5	4	PZA03317.1	140.3	150406740
CFD12	6	9	SYN10183	12	7051221
CFD01	6	9	SYN10183	12.1	7051221
CFD05	6	9	PUT-163a-4730575-2151	12.1	92346815
CFF01	6	9	SYN10183	12.7	7051221
CFF06	6	9	SYN10183	13	7051221
CFD02	6	9	SYN10183	13.7	7051221
CFF04	6	9	SYN10183	14.2	7051221
CFD06	6	9	PUT-163a-4730575-2151	19.6	92346815
CFF10	6	9	SYN10183	20.6	7051221
CFD09	6	9	PUT-163a-4730575-2151	21.8	92346815
CFF01	6	9	PUT-163a-4730575-2151	28.9	92346815
CFD04	6	9	PUT-163a-4730575-2151	31.4	92346815
CFD11	6	9	PUT-163a-4730575-2151	33.9	92346815
CFD01	6	9	PUT-163a-4730575-2151	34	92346815
CFD07	6	9	PUT-163a-4730575-2151	34.7	92346815
CFD02	6	9	PUT-163a-4730575-2151	38	92346815
CFD03	6	9	PUT-163a-4730575-2151	38.1	92346815
CFF08	6	9	PUT-163a-4730575-2151	39.2	92346815
CFF09	6	7	PZE-106073612	45.9	173931817
CFF09	6	7	PZE-106073613	45.9	173931722
CFF10	6	9	PUT-163a-4730575-2151	46.3	92346815
CFD01	6	7	PZE-106073612	54.5	173931817
CFD01	6	7	PZE-106073613	54.5	173931722
CFF01	6	7	PZE-106073612	56.7	173931817
CFF01	6	7	PZE-106073613	56.7	173931722
CFD04	6	7	PZE-106073612	57.2	173931817
CFD04	6	7	PZE-106073613	57.2	173931722
CFD09	6	3	PZE-103128711	58.1	185556616
CFD07	6	7	PZE-106073612	58.5	173931817
CFD07	6	7	PZE-106073613	58.5	173931722
CFF02	6	7	PZE-106073612	58.8	173931817
CFF02	6	7	PZE-106073613	58.8	173931722
CFF03	6	9	PUT-163a-4730575-2151	63.8	92346815
CFF10	6	7	PZE-106073612	68.3	173931817
CFF10	6	7	PZE-106073613	68.3	173931722
CFF06	6	7	PZE-106073612	69.5	173931817
CFF06	6	7	PZE-106073613	69.5	173931722

CFF06	6	1	PZE-101181366	71.8	225714207
CFF10	6	7	PZE-107032178	123.6	42291768
CFD06	6	7	PZE-107032178	137.9	42291768
CFD11	7	2	PZE-102187151	29.4	231152724
CFD02	7	8	SYN28351	31.9	85670381
CFD05	7	8	SYN28351	32.7	85670381
CFD07	7	2	PZA00153.6	40.2	170296830
CFD07	7	2	PZE-102187151	40.2	231152724
CFD07	7	8	SYN28351	40.2	85670381
CFD03	7	8	SYN28351	41.4	85670381
CFF02	7	8	SYN28351	41.4	85670381
CFF02	7	2	PZE-102187116	42.2	231117697
CFF02	7	2	SYN5581	42.2	231206946
CFF02	7	2	SYN5585	42.2	231206883
CFD03	7	2	PZE-102187151	43.4	231152724
CFD12	7	8	SYN28351	43.8	85670381
CFF15	7	8	SYN28351	44	85670381
CFF15	7	2	PZE-102187119	44.9	231115198
CFF15	7	2	SYN5581	44.9	231206946
CFF09	7	2	PZE-102187119	45.6	231115198
CFF09	7	8	SYN28351	45.6	85670381
CFF09	7	2	SYN5581	45.6	231206946
CFF08	7	2	SYN5581	46.4	231206946
CFD04	7	8	SYN28351	49.8	85670381
CFF04	7	8	SYN28351	49.8	85670381
CFF04	7	2	SYN5581	49.8	231206946
CFF04	7	2	SYN5585	49.8	231206883
CFD09	7	2	SYN5581	50.2	231206946
CFD09	7	2	SYN5585	50.2	231206883
CFF01	7	2	SYN5581	50.7	231206946
CFF01	7	2	SYN5585	50.7	231206883
CFF10	7	2	SYN5581	50.8	231206946
CFF07	7	2	PZE-102187119	51.7	231115198
CFF07	7	8	SYN28351	51.7	85670381
CFD04	7	2	PZE-102187151	51.8	231152724
CFD10	7	8	SYN28351	51.8	85670381
CFF06	7	2	PZE-102187151	51.9	231152724
CFF06	7	2	SYN5585	51.9	231206883
CFF07	7	2	SYN5581	52.5	231206946
CFD10	7	2	SYN5585	53.9	231206883
CFD01	7	2	SYN5581	55.2	231206946
CFD01	7	2	SYN5585	55.2	231206883
CFF12	7	10	PZE-110053442	56.3	100492412
CFF06	7	2	PZE-102187116	56.6	231117697
CFF06	7	8	SYN28351	56.6	85670381
CFF06	7	2	SYN5581	56.6	231206946
CFF03	7	2	PZE-102187119	62.3	231115198
CFF03	7	8	SYN28351	62.3	85670381
CFF03	7	2	SYN5581	62.3	231206946
CFD06	7	10	PZE-110053442	68.5	100492412
CFD05	7	2	PHM424.13	75.5	208227068

CFF09	7	10	PZE-110053442	89.6	100492412
CFD05	7	3	PZA00043.1	96.1	200125220
CFF15	7	10	PZE-110053442	101.2	100492412
CFF10	7	2	PHM424.13	104.4	208227068
CFD02	7	3	PZA00043.1	108.4	200125220
CFD07	7	3	PZA00043.1	115.5	200125220
CFD09	7	3	PZA00043.1	116.3	200125220
CFF07	7	10	PZE-110053442	124.5	100492412
CFF02	7	3	PZA00043.1	146.9	200125220
CFD02	8	1	SYN3668	56.4	212728227
CFD02	8	1	SYN3669	56.4	212728147
CFD02	8	1	SYN3672	56.4	212728669
CFF02	8	1	SYN3668	71.1	212728227
CFF02	8	1	SYN3669	71.1	212728147
CFF02	8	1	SYN3672	71.1	212728669
CFD02	8	3	PUT-163a-60346954-2563	81.6	158354188
CFF02	8	3	PUT-163a-60346954-2563	105	158354188
CFD07	9	1	PZE-101174118	48	217831613
CFD05	9	1	PZE-109086414	48.2	79095469
CFD02	9	1	PZE-109086414	60.2	79095469
CFF10	9	1	PZE-109086414	67	79095469
CFF02	9	1	PZE-109086414	79	79095469
CFD11	9	2	PUT-163a-14245519-343	84.7	222879720
CFD12	9	2	PUT-163a-14245519-343	85.6	222879720
CFF03	9	2	PUT-163a-14245519-343	128	222879720
CFF12	10	6	PZE-110042307	13.5	67773609
CFF03	10	6	PZE-110042307	27.1	67773609
CFF12	10	4	PUT-163a-149005002-744	30.7	216454725
CFD02	10	5	PZE-110041462	35.6	139406437
CFD02	10	6	PZE-106037049	37	84901278
CFD02	10	6	PZE-110042307	37	67773609
CFD03	10	6	PZE-106037049	38.9	84901278
CFD05	10	4	PUT-163a-149005002-744	39.9	216454725
CFD10	10	6	PZE-106037049	41.4	84901278
CFD07	10	5	PZE-110041462	42	139406437
CFD02	10	4	PUT-163a-149005002-744	42.6	216454725
CFD06	10	4	PUT-163a-149005002-744	43.8	216454725
CFF02	10	5	PZE-110041462	46	139406437
CFD04	10	6	PZE-106037049	46.8	84901278
CFD03	10	4	PUT-163a-149005002-744	46.9	216454725
CFD10	10	4	PUT-163a-149005002-744	47.4	216454725
CFF02	10	6	PZE-106037049	48.5	84901278
CFD09	10	5	PZE-110041462	50.6	139406437
CFF07	10	1	PZE-101175968	55.6	220376839
CFD04	10	4	PUT-163a-149005002-744	57.6	216454725
CFF09	10	4	PUT-163a-149005002-744	58.5	216454725
CFD01	10	6	PZE-110042307	58.8	67773609
CFF04	10	4	PUT-163a-149005002-744	60.6	216454725
CFF13	10	6	PZE-110042307	62.4	67773609
CFF10	10	4	PUT-163a-149005002-744	63.1	216454725
CFF01	10	4	PUT-163a-149005002-744	63.5	216454725

CFD09	10	4	PUT-163a-149005002-744	63.9	216454725
CFD01	10	4	PUT-163a-149005002-744	69	216454725
CFF06	10	4	PUT-163a-149005002-744	69.4	216454725
CFF08	10	4	PUT-163a-149005002-744	72.7	216454725
CFF04	10	1	PZE-101175968	106.7	220376839
CFF09	10	1	PZE-101175968	112	220376839
CFD11	10	1	PZE-101175968	112.2	220376839
CFF01	10	1	PZE-101175968	116.5	220376839
CFD01	10	1	PZE-101175968	119.5	220376839
CFF02	10	1	PZE-101175968	123.6	220376839
CFF08	10	1	PZE-101175968	125.3	220376839
CFD03	1	0	PUT-163a-94473548-4861	47	0
CFD03	1	0	PUT-163a-94473548-4862	47	0
CFD05	1	0	PUT-163a-94473548-4861	50.7	0
CFD05	1	0	PUT-163a-94473548-4862	50.7	0
CFD11	1	0	PZA01039.1	54	0
CFD10	1	0	PUT-163a-94473548-4861	55.3	0
CFD10	1	0	PUT-163a-94473548-4862	55.3	0
CFD09	1	0	PUT-163a-94473548-4861	55.9	0
CFD09	1	0	PUT-163a-94473548-4862	55.9	0
CFD12	1	0	PUT-163a-94473548-4861	60	0
CFD12	1	0	PUT-163a-94473548-4862	60	0
CFD02	1	0	PUT-163a-94473548-4861	60.5	0
CFD02	1	0	PUT-163a-94473548-4862	60.5	0
CFD07	1	0	PUT-163a-94473548-4861	62.2	0
CFD07	1	0	PUT-163a-94473548-4862	62.2	0
CFF08	1	0	PUT-163a-94473548-4861	63.4	0
CFF08	1	0	PUT-163a-94473548-4862	63.4	0
CFD11	1	0	PZA00560.2	66	0
CFD01	1	0	PUT-163a-94473548-4861	67.6	0
CFD01	1	0	PUT-163a-94473548-4862	67.6	0
CFD04	1	0	PUT-163a-94473548-4861	69.6	0
CFD04	1	0	PUT-163a-94473548-4862	69.6	0
CFF12	1	0	PUT-163a-94473548-4861	72.8	0
CFF12	1	0	PUT-163a-94473548-4862	72.8	0
CFF01	1	0	PUT-163a-94473548-4861	73.4	0
CFF01	1	0	PUT-163a-94473548-4862	73.4	0
CFD06	1	0	PUT-163a-94473548-4861	78.3	0
CFD06	1	0	PUT-163a-94473548-4862	78.3	0
CFD09	1	0	PHM14519.8	83.4	0
CFD05	1	0	PZA01039.1	89.1	0
CFF03	1	0	PUT-163a-94473548-4861	89.4	0
CFF03	1	0	PUT-163a-94473548-4862	89.4	0
CFF08	1	0	PZE-101130736	89.8	0
CFD10	1	0	PZA01039.1	90.6	0
CFD03	1	0	PZA01039.1	93	0
CFD02	1	0	PUT-163a-60356868-2816	93.3	0
CFD12	1	0	PZE-101130736	95	0
CFD12	1	0	PHM14519.8	102	0
CFF09	1	0	PZE-101130736	105.2	0
CFD09	1	0	PZA01039.1	106.6	0

CFD09	1	0	PUT-163a-148943685-476	110	0
CFF12	1	0	PZE-101130736	111.1	0
CFF10	1	0	PHM14519.8	114.6	0
CFF12	1	0	PHM14519.8	115.6	0
CFF15	1	0	PZE-101130736	119.9	0
CFF13	1	0	PZE-101130736	121	0
CFD04	1	0	PZA01039.1	128.5	0
CFD01	1	0	PZA01039.1	131.2	0
CFF13	1	0	PHM14519.8	131.8	0
CFD07	1	0	PZA00560.2	133.4	0
CFD06	1	0	PZA01039.1	134	0
CFD01	1	0	PUT-163a-148943685-476	136	0
CFF02	1	0	PUT-163a-60356868-2816	145.1	0
CFF04	1	0	PUT-163a-148943685-476	145.6	0
CFF09	1	0	PUT-163a-148943685-476	148.8	0
CFF12	1	0	PUT-163a-148943685-476	149.5	0
CFF01	1	0	PZA01039.1	154.4	0
CFF02	1	0	PZA01039.1	159.3	0
CFF01	1	0	PUT-163a-148943685-476	161.6	0
CFF02	1	0	PUT-163a-148943685-476	163.3	0
CFF13	1	0	PUT-163a-148943685-476	166.6	0
CFF06	1	0	PUT-163a-60356868-2816	176.9	0
CFF06	1	0	PUT-163a-148943685-476	188	0
CFD06	1	0	PZE-101235343	189.1	0
CFF03	1	0	PUT-163a-148943685-476	191.8	0
CFF08	1	0	ZM013672-0446	195.1	0
CFF09	1	0	ZM013672-0446	199.1	0
CFF04	1	0	ZM013672-0446	200.9	0
CFF10	1	0	ZM013672-0446	202.8	0
CFF07	1	0	ZM013672-0446	206.9	0
CFF12	1	0	ZM013672-0446	210.7	0
CFD01	1	0	ZM013672-0446	211	0
CFF01	1	0	ZM013672-0446	219.6	0
CFF02	1	0	ZM013672-0446	234.7	0
CFF06	1	0	PZE-101235343	236.8	0
CFF15	1	0	ZM013672-0446	248.6	0
CFF03	1	0	PZE-101235343	252.7	0
CFF06	1	0	ZM013672-0446	259.6	0
CFF03	1	0	ZM013672-0446	276.9	0
CFD03	2	0	ZM012095-0708	0	0
CFD04	2	0	ZM012095-0708	0	0
CFD09	2	0	ZM012095-0708	0	0
CFD11	2	0	ZM012095-0708	0	0
CFF02	2	0	ZM013608-0315	13.3	0
CFD04	2	0	ZM013608-0315	14.7	0
CFF04	2	0	ZM013608-0315	16	0
CFD09	2	0	ZM013608-0315	16.8	0
CFD03	2	0	ZM013608-0315	17.1	0
CFF03	2	0	ZM013608-0315	18.4	0
CFD05	2	0	ZM013608-0315	19.1	0
CFF06	2	0	ZM013608-0315	19.2	0

CFD02	2	0	ZM013608-0315	21.5	0
CFD10	2	0	ZM013608-0315	22.7	0
CFF09	2	0	ZM013608-0315	24.4	0
CFD06	2	0	ZM013608-0315	29	0
CFD10	2	0	PUT-163a-148957860-553	48.2	0
CFD05	2	0	PUT-163a-148957860-553	50.2	0
CFD03	2	0	PUT-163a-148957860-553	52	0
CFD05	2	0	PZA01034.1	56.7	0
CFF12	2	0	PUT-163a-148957860-553	56.9	0
CFD06	2	0	PUT-163a-148957860-553	57.4	0
CFD04	2	0	PUT-163a-148957860-553	57.6	0
CFD12	2	0	PUT-163a-148957860-553	57.7	0
CFF01	2	0	PUT-163a-148957860-553	57.7	0
CFD02	2	0	PUT-163a-148957860-553	58.4	0
CFF10	2	0	PUT-163a-148957860-553	58.6	0
CFF03	2	0	PUT-163a-148957860-553	59.6	0
CFD09	2	0	PUT-163a-148957860-553	63.6	0
CFD01	2	0	PUT-163a-148957860-553	66.1	0
CFF09	2	0	PUT-163a-148957860-553	66.7	0
CFD02	2	0	PZA01034.1	66.8	0
CFF08	2	0	PUT-163a-148957860-553	70.5	0
CFD07	2	0	PZA00755.2	70.7	0
CFF02	2	0	PZA01034.1	76.2	0
CFD09	2	0	PZA00755.2	80.2	0
CFD06	2	0	PZA00755.2	85	0
CFF12	2	0	PZA00755.2	107.1	0
CFF08	2	0	PZA00755.2	111.4	0
CFF04	2	0	PZA00755.2	113.7	0
CFF10	2	0	PZA00755.2	115.6	0
CFF03	2	0	PZA00755.2	116.7	0
CFD10	2	0	PUT-163a-93013486-4809	118.3	0
CFD03	2	0	PUT-163a-93013486-4809	130.5	0
CFD02	2	0	PUT-163a-93013486-4809	141.9	0
CFD04	2	0	PUT-163a-93013486-4809	148.4	0
CFD12	2	0	PUT-163a-93013486-4809	154.5	0
CFD01	2	0	PUT-163a-93013486-4809	166.7	0
CFF12	2	0	PUT-163a-93013486-4809	173.4	0
CFF01	2	0	PUT-163a-93013486-4809	174.5	0
CFF08	2	0	PUT-163a-93013486-4809	177.5	0
CFF07	2	0	PUT-163a-93013486-4809	194.4	0
CFF03	2	0	PUT-163a-93013486-4809	195.9	0
CFD07	3	0	PUT-163a-74241344-3659	36.5	0
CFD09	3	0	PUT-163a-74241344-3659	40.1	0
CFD05	3	0	PUT-163a-101392844-22	40.7	0
CFD05	3	0	PHM4955.12	42	0
CFD05	3	0	PZA02474.1	42	0
CFD05	3	0	PZA02906.7	42	0
CFD06	3	0	PUT-163a-74241344-3659	42.5	0
CFD09	3	0	PZA02296.1	46.7	0
CFD09	3	0	PZA02906.7	46.7	0
CFD04	3	0	PUT-163a-101392844-22	46.8	0

CFF06	3	0	PUT-163a-17930775-1188	49.3	0
CFD06	3	0	PZA02296.1	49.5	0
CFD11	3	0	SYNGENTA17315	49.6	0
CFD06	3	0	PZA02906.12	50.5	0
CFD06	3	0	PUT-163a-17930775-1188	51	0
CFD06	3	0	PHM4955.12	51.5	0
CFD07	3	0	PUT-163a-17930775-1188	53.2	0
CFF07	3	0	PUT-163a-101392844-22	53.9	0
CFF07	3	0	PUT-163a-17930775-1188	53.9	0
CFD03	3	0	PUT-163a-101392844-22	55.1	0
CFD11	3	0	PUT-163a-17930775-1188	56.9	0
CFD12	3	0	PZA02296.1	58.3	0
CFD10	3	0	PUT-163a-101392844-22	59.4	0
CFD04	3	0	PHM4955.12	59.6	0
CFD04	3	0	PZA02906.7	59.6	0
CFD05	3	0	PZA01726.1	59.6	0
CFF13	3	0	PUT-163a-17930775-1188	59.6	0
CFD03	3	0	PZA02474.1	60.1	0
CFF06	3	0	PZA02474.1	60.1	0
CFF02	3	0	PUT-163a-101392844-22	60.6	0
CFF02	3	0	PUT-163a-17930775-1188	60.6	0
CFD03	3	0	PZA02906.7	61.1	0
CFD11	3	0	PZA02906.7	61.7	0
CFF15	3	0	PUT-163a-17930775-1188	61.9	0
CFD11	3	0	PHM4955.12	62.9	0
CFD07	3	0	PUT-163a-101392844-22	63.8	0
CFF10	3	0	PUT-163a-17930775-1188	64.1	0
CFF10	3	0	PZA02296.1	64.1	0
CFF01	3	0	PZA02906.7	64.9	0
CFD03	3	0	PHM4955.12	65.1	0
CFF07	3	0	PZA02474.1	65.1	0
CFD10	3	0	PZA02474.1	65.4	0
CFF02	3	0	PHM4955.12	65.5	0
CFF02	3	0	PZA02474.1	65.5	0
CFF01	3	0	PHM4955.12	65.9	0
CFD10	3	0	PHM4955.12	66.4	0
CFD10	3	0	PZA02906.7	66.4	0
CFF07	3	0	PZA02906.12	66.7	0
CFF07	3	0	PZA02906.7	66.7	0
CFD02	3	0	PUT-163a-101392844-22	67.2	0
CFF15	3	0	PZA02906.7	67.3	0
CFD07	3	0	PZA02474.1	67.4	0
CFD07	3	0	PHM4955.12	69.2	0
CFD07	3	0	PZA02906.7	69.2	0
CFD01	3	0	PZA02906.7	69.9	0
CFD01	3	0	PHM4955.12	71.1	0
CFD04	3	0	PZA01726.1	71.6	0
CFD12	3	0	PUT-163a-17930775-1188	71.7	0
CFD02	3	0	PZA02474.1	72.1	0
CFF07	3	0	PHM4955.12	72.3	0
CFD02	3	0	PZA02906.7	74.2	0

CFF10	3	0	PZA02474.1	76.8	0
CFF10	3	0	PZA02906.7	77.8	0
CFF04	3	0	PZA01726.1	79.6	0
CFD03	3	0	PZA01726.1	80.2	0
CFF12	3	0	PZA01726.1	80.6	0
CFD10	3	0	PZA01726.1	81.5	0
CFD12	3	0	PZA02474.1	87.4	0
CFF01	3	0	PZA01726.1	87.5	0
CFF09	3	0	PZA01726.1	87.5	0
CFD12	3	0	PZA02906.12	88.4	0
CFF03	3	0	PZA01726.1	88.8	0
CFD04	3	0	ZM007732-0520	90.1	0
CFD12	3	0	PHM4955.12	90.4	0
CFF01	3	0	PUT-163a-78109387-4288	90.5	0
CFD02	3	0	PZA01726.1	92.4	0
CFF04	3	0	ZM007732-0520	93.5	0
CFD03	3	0	ZM007732-0520	96.2	0
CFF02	3	0	PUT-163a-78109387-4288	96.8	0
CFF07	3	0	PZA01726.1	97.1	0
CFF06	3	0	PZA01726.1	98.5	0
CFD04	3	0	PUT-163a-149112808-975	99.1	0
CFD01	3	0	PZA01726.1	99.9	0
CFF12	3	0	ZM007732-0520	100.6	0
CFD03	3	0	PUT-163a-149112808-975	101.2	0
CFD01	3	0	PUT-163a-78109387-4288	102.3	0
CFD10	3	0	ZM007732-0520	102.9	0
CFD12	3	0	PZA01726.1	104.4	0
CFD06	3	0	PZE-103152091	105.4	0
CFF08	3	0	ZM007732-0520	106	0
CFF03	3	0	ZM007732-0520	106.3	0
CFF09	3	0	ZM007732-0520	109.9	0
CFF10	3	0	ZM007732-0520	110.5	0
CFD10	3	0	PUT-163a-149112808-975	111	0
CFF01	3	0	ZM007732-0520	111.2	0
CFF06	3	0	ZM007732-0520	113.6	0
CFF07	3	0	ZM007732-0520	114.7	0
CFF01	3	0	PZE-103152091	115	0
CFF10	3	0	PUT-163a-149112808-975	116	0
CFF09	3	0	PUT-163a-149112808-975	116.1	0
CFD01	3	0	ZM007732-0520	116.9	0
CFD11	3	0	PUT-163a-149112808-975	118.6	0
CFF07	3	0	PUT-163a-149112808-975	119.5	0
CFF02	3	0	ZM007732-0520	120.2	0
CFD02	3	0	PUT-163a-149112808-975	121.8	0
CFD09	3	0	ZM008460-0475	123.5	0
CFF02	3	0	PUT-163a-149112808-975	125.1	0
CFF15	3	0	ZM007732-0520	127.1	0
CFF02	3	0	PZE-103152091	128.8	0
CFD01	3	0	PZE-103152091	136.3	0
CFD06	3	0	ZM008460-0475	146.9	0
CFF12	3	0	ZM008460-0475	159.2	0

CFF08	3	0	ZM008460-0475	161.3	0
CFF04	3	0	ZM008460-0475	161.4	0
CFF10	3	0	ZM008460-0475	162.9	0
CFF01	3	0	ZM008460-0475	165.7	0
CFD02	3	0	ZM008460-0475	170.8	0
CFF07	3	0	ZM008460-0475	170.9	0
CFF06	3	0	ZM008460-0475	171.3	0
CFD01	3	0	ZM008460-0475	175.3	0
CFD02	4	0	PUT-163a-50330221-2211	17.6	0
CFD02	4	0	PZE-104013979	20.4	0
CFD05	4	0	PUT-163a-50330221-2211	24.4	0
CFD10	4	0	PUT-163a-50330221-2211	29.4	0
CFD05	4	0	PZE-104013979	29.9	0
CFD03	4	0	PUT-163a-50330221-2211	30.1	0
CFD07	4	0	PUT-163a-50330221-2211	31.8	0
CFD03	4	0	PZE-104013979	32.1	0
CFD04	4	0	PZE-104013979	32.3	0
CFD10	4	0	PZE-104013979	34.6	0
CFF06	4	0	PZE-104013979	34.6	0
CFD12	4	0	PUT-163a-50330221-2211	34.9	0
CFD07	4	0	PZE-104013979	35.7	0
CFD01	4	0	PZE-104013979	37.3	0
CFD02	4	0	PZA00311.4	37.4	0
CFF01	4	0	PZE-104013979	37.7	0
CFF07	4	0	PZE-104013979	38.6	0
CFF10	4	0	PZE-104013979	38.6	0
CFD02	4	0	PUT-163a-89763353-4714	38.8	0
CFD12	4	0	PZE-104013979	39.4	0
CFD06	4	0	PUT-163a-50330221-2211	42.8	0
CFF09	4	0	PZE-104013979	43.1	0
CFD06	4	0	PZE-104013979	48.7	0
CFF15	4	0	PZA00311.4	51.7	0
CFD09	4	0	PUT-163a-89763353-4714	52.2	0
CFD01	4	0	PUT-163a-89763353-4712	52.8	0
CFD12	4	0	PUT-163a-89763353-4714	54.1	0
CFD10	4	0	PZA00311.4	54.7	0
CFF12	4	0	PZA00311.4	55.2	0
CFD02	4	0	PZE-100002337	55.7	0
CFD02	4	0	PZE-100002365	55.7	0
CFD02	4	0	PZE-100002384	55.7	0
CFD07	4	0	PUT-163a-89763353-4714	55.7	0
CFD10	4	0	PUT-163a-89763353-4714	56.7	0
CFF07	4	0	PZA00311.4	57	0
CFD02	4	0	PZE0008531494	57.1	0
CFD02	4	0	PZE-100002463	57.1	0
CFD02	4	0	PZE-100002465	57.1	0
CFF08	4	0	PZA00311.4	58.4	0
CFD02	4	0	PZE-100002529	58.5	0
CFF01	4	0	PUT-163a-89763353-4712	59.6	0
CFF09	4	0	PZA00311.4	59.6	0
CFF04	4	0	PUT-163a-89763353-4712	60.2	0

CFF04	4	0	PUT-163a-89763353-4714	60.2	0
CFF10	4	0	PZA00311.4	60.2	0
CFF02	4	0	PZA00311.4	60.8	0
CFF09	4	0	PUT-163a-89763353-4712	61.2	0
CFF09	4	0	PUT-163a-89763353-4714	61.2	0
CFF06	4	0	PUT-163a-89763353-4712	62.3	0
CFF06	4	0	PUT-163a-89763353-4714	62.3	0
CFF02	4	0	PUT-163a-89763353-4712	63.3	0
CFF02	4	0	PUT-163a-89763353-4714	63.3	0
CFD03	4	0	PZE-100002337	76.3	0
CFD03	4	0	PZE-100002384	76.3	0
CFD05	4	0	PZE-100002337	76.5	0
CFD05	4	0	PZE-100002384	76.5	0
CFD05	4	0	PZE-100002463	76.5	0
CFD05	4	0	PZE-100002464	76.5	0
CFD05	4	0	PZE-100002478	78	0
CFD05	4	0	PZE-100002529	78	0
CFD04	4	0	PZE-100002337	79.1	0
CFD04	4	0	PZE-100002384	79.1	0
CFD04	4	0	PZE-100002464	79.1	0
CFD04	4	0	PZE-100002478	79.1	0
CFD04	4	0	PZE-100002529	79.1	0
CFD04	4	0	PZE-100002530	79.1	0
CFD09	4	0	PZE-100002337	85.6	0
CFD10	4	0	PZE0008531494	86.9	0
CFD10	4	0	PZE-100002337	86.9	0
CFD10	4	0	PZE-100002365	86.9	0
CFD10	4	0	PZE-100002384	86.9	0
CFD10	4	0	PZE-100002463	86.9	0
CFD10	4	0	PZE-100002465	86.9	0
CFD10	4	0	PZE-100002529	86.9	0
CFD09	4	0	PZE-100002365	87.4	0
CFD09	4	0	PZE-100002384	87.4	0
CFD09	4	0	PZE-100002416	87.4	0
CFD12	4	0	PZE-100002365	88.9	0
CFD12	4	0	PZE-100002384	88.9	0
CFD12	4	0	PZE-100002464	88.9	0
CFD12	4	0	PZE-100002478	88.9	0
CFD12	4	0	PZE-100002529	88.9	0
CFD12	4	0	PZE-100002530	88.9	0
CFD11	4	0	PZE-100002337	89.7	0
CFD11	4	0	PZE-100002365	90.9	0
CFD11	4	0	PZE-100002384	90.9	0
CFD11	4	0	PZE-100002416	90.9	0
CFF01	4	0	PZE0008531494	94.2	0
CFF01	4	0	PZE-100002337	94.2	0
CFF01	4	0	PZE-100002365	94.2	0
CFF01	4	0	PZE-100002384	94.2	0
CFF01	4	0	PZE-100002465	94.2	0
CFF01	4	0	PZE-100002529	94.2	0
CFD07	4	0	PZE-100002320	94.9	0

CFD06	4	0	PZE-100002337	95.8	0
CFD06	4	0	PZE-100002365	95.8	0
CFD06	4	0	PZE-100002384	95.8	0
CFD06	4	0	PZE-100002530	95.8	0
CFD07	4	0	PZE0008531494	96.3	0
CFD07	4	0	PZE-100002337	96.3	0
CFD07	4	0	PZE-100002384	96.3	0
CFD07	4	0	PZE-100002465	96.3	0
CFD07	4	0	PZE-100002530	96.3	0
CFD02	4	0	PZA00086.8	101.5	0
CFF09	4	0	PZE0008531494	102	0
CFF09	4	0	PZE-100002464	102	0
CFF09	4	0	PZE-100002465	102	0
CFD01	4	0	PZE0008531494	102.7	0
CFD01	4	0	PZE-100002337	102.7	0
CFD01	4	0	PZE-100002365	102.7	0
CFD01	4	0	PZE-100002384	102.7	0
CFD01	4	0	PZE-100002465	102.7	0
CFD01	4	0	PZE-100002529	102.7	0
CFF15	4	0	PZE0008531494	103	0
CFF15	4	0	PZE-100002320	103	0
CFF15	4	0	PZE-100002463	103	0
CFF15	4	0	PZE-100002465	103	0
CFF15	4	0	PZE-100002478	103	0
CFF13	4	0	PZE0008531494	103.1	0
CFF13	4	0	PZE-100002320	103.1	0
CFF13	4	0	PZE-100002365	103.1	0
CFF13	4	0	PZE-100002384	103.1	0
CFF13	4	0	PZE-100002463	103.1	0
CFF13	4	0	PZE-100002464	103.1	0
CFF13	4	0	PZE-100002465	103.1	0
CFF01	4	0	PZB01487.2	104.4	0
CFF07	4	0	PZE0008531494	105.3	0
CFF07	4	0	PZE-100002463	105.3	0
CFF07	4	0	PZE-100002464	105.3	0
CFF07	4	0	PZE-100002465	105.3	0
CFF10	4	0	PZE0008531494	106.7	0
CFF10	4	0	PZE-100002320	106.7	0
CFF10	4	0	PZE-100002365	106.7	0
CFF10	4	0	PZE-100002384	106.7	0
CFF10	4	0	PZE-100002463	106.7	0
CFF10	4	0	PZE-100002464	106.7	0
CFF10	4	0	PZE-100002465	106.7	0
CFF10	4	0	PZE-100002478	106.7	0
CFF10	4	0	PZE-100002530	106.7	0
CFD11	4	0	PZE-100000018	108.1	0
CFD11	4	0	PZE-100000045	108.1	0
CFD12	4	0	PZE-100000018	108.4	0
CFF12	4	0	PZE0008531494	108.4	0
CFF12	4	0	PZE-100002320	108.4	0
CFF12	4	0	PZE-100002365	108.4	0

CFF12	4	0	PZE-100002384	108.4	0
CFF12	4	0	PZE-100002463	108.4	0
CFF12	4	0	PZE-100002464	108.4	0
CFF12	4	0	PZE-100002465	108.4	0
CFD09	4	0	PZE-100000018	110.6	0
CFD09	4	0	PZE-100000045	110.6	0
CFF15	4	0	PZB01487.2	111.1	0
CFF10	4	0	PZB01487.2	112.1	0
CFF02	4	0	PZE0008532123	112.2	0
CFF02	4	0	PZE-100002463	112.2	0
CFF02	4	0	PZE-100002471	112.2	0
CFD01	4	0	PZB01487.2	112.3	0
CFD05	4	0	PZA00086.8	114.4	0
CFF04	4	0	PZE0008531494	114.9	0
CFF04	4	0	PZE-100002463	114.9	0
CFF04	4	0	PZE-100002464	114.9	0
CFF04	4	0	PZE-100002465	114.9	0
CFF04	4	0	PZE-100002478	114.9	0
CFD06	4	0	PZE-100000018	116.2	0
CFD06	4	0	PZE-100000045	116.2	0
CFF10	4	0	PZE-100000018	117.5	0
CFF06	4	0	PZE-100002316	119.8	0
CFF08	4	0	PZE-100000018	122.3	0
CFF08	4	0	PZE-100000045	122.3	0
CFF04	4	0	PZE-100000018	122.7	0
CFF04	4	0	PZE-100000045	122.7	0
CFF04	4	0	PZE-100000066	122.7	0
CFF13	4	0	PZE-100000018	122.7	0
CFF13	4	0	PZE-100000045	122.7	0
CFF06	4	0	PZE0008531494	124.6	0
CFF06	4	0	PZE-100002464	124.6	0
CFF06	4	0	PZE-100002465	124.6	0
CFF03	4	0	PZE-100000018	125.4	0
CFF03	4	0	PZE-100000045	125.4	0
CFF02	4	0	PZB01487.2	125.8	0
CFF12	4	0	PZE-100000018	127.6	0
CFF12	4	0	PZE-100000045	127.6	0
CFF06	4	0	PZE-100002529	128.6	0
CFF06	4	0	PZB01487.2	130	0
CFD11	4	0	PZA00086.8	132.8	0
CFD04	4	0	PZA00086.8	135.8	0
CFF06	4	0	PZE-100000045	137.3	0
CFF06	4	0	PZE-100000066	137.3	0
CFD07	4	0	PZA00086.8	137.4	0
CFF15	4	0	PZA00086.8	149.2	0
CFF02	4	0	PZA00086.8	163.4	0
CFF06	5	0	PUT-163a-29129786-1728	0	0
CFF02	5	0	PUT-163a-29129786-1728	5.1	0
CFD04	5	0	PUT-163a-29129786-1728	6.8	0
CFD12	5	0	PUT-163a-29129786-1728	7.1	0
CFD03	5	0	PUT-163a-29129786-1728	8.1	0

CFD02	5	0	PUT-163a-29129786-1728	10	0
CFD06	5	0	PUT-163a-29129786-1728	22.4	0
CFF03	5	0	SYNGENTA1616	22.9	0
CFF04	5	0	PUT-163a-148942702-460	40.7	0
CFF04	5	0	PUT-163a-148942702-462	40.7	0
CFF04	5	0	PUT-163a-148942702-464	40.7	0
CFF04	5	0	PZA03578.1	40.7	0
CFD12	5	0	PUT-163a-148942702-460	41.8	0
CFD12	5	0	PUT-163a-148942702-462	41.8	0
CFD12	5	0	PUT-163a-148942702-464	41.8	0
CFD12	5	0	PZA03578.1	41.8	0
CFF01	5	0	PUT-163a-148942702-460	44.8	0
CFF01	5	0	PUT-163a-148942702-462	44.8	0
CFF01	5	0	PUT-163a-148942702-464	44.8	0
CFF01	5	0	PZA03578.1	44.8	0
CFF10	5	0	PUT-163a-148942702-460	47.2	0
CFF10	5	0	PUT-163a-148942702-462	47.2	0
CFF10	5	0	PUT-163a-148942702-464	47.2	0
CFF10	5	0	PZA03578.1	47.2	0
CFF15	5	0	PUT-163a-148942702-460	47.9	0
CFF15	5	0	PUT-163a-148942702-462	47.9	0
CFF02	5	0	PUT-163a-148942702-460	51.8	0
CFF02	5	0	PUT-163a-148942702-462	51.8	0
CFF02	5	0	PUT-163a-148942702-464	51.8	0
CFF02	5	0	PZA03578.1	51.8	0
CFD01	5	0	PUT-163a-148942702-460	51.9	0
CFD01	5	0	PUT-163a-148942702-462	51.9	0
CFD01	5	0	PUT-163a-148942702-464	51.9	0
CFD01	5	0	PZA03578.1	51.9	0
CFD07	5	0	PUT-163a-13515860-232	53.6	0
CFF10	5	0	PUT-163a-13515860-234	57.1	0
CFF10	5	0	PUT-163a-13515860-235	57.1	0
CFF09	5	0	PUT-163a-148942702-460	59.5	0
CFF09	5	0	PUT-163a-148942702-462	59.5	0
CFF09	5	0	PZA03578.1	59.5	0
CFF02	5	0	PUT-163a-13515860-232	59.9	0
CFF02	5	0	PUT-163a-13515860-234	59.9	0
CFF02	5	0	PUT-163a-13515860-235	59.9	0
CFD01	5	0	PUT-163a-13515860-232	60.3	0
CFD01	5	0	PUT-163a-13515860-234	60.3	0
CFD01	5	0	PUT-163a-13515860-235	60.3	0
CFF01	5	0	PUT-163a-13515860-232	60.8	0
CFF01	5	0	PUT-163a-13515860-234	60.8	0
CFF01	5	0	PUT-163a-13515860-235	60.8	0
CFF07	5	0	PUT-163a-148942702-460	62.1	0
CFF07	5	0	PUT-163a-148942702-462	62.1	0
CFF07	5	0	PUT-163a-148942702-464	62.1	0
CFF07	5	0	PZA03578.1	62.1	0
CFF06	5	0	PUT-163a-148942702-460	62.6	0
CFF06	5	0	PUT-163a-148942702-462	62.6	0
CFF06	5	0	PUT-163a-148942702-464	62.6	0

CFF06	5	0	PZA03578.1	62.6	0
CFD10	5	0	PUT-163a-13515860-232	63.4	0
CFD10	5	0	PUT-163a-13515860-234	63.4	0
CFD10	5	0	PUT-163a-13515860-235	63.4	0
CFF06	5	0	PUT-163a-13515860-234	73.3	0
CFF06	5	0	PUT-163a-13515860-235	73.3	0
CFD10	5	0	PZB01689.1	73.5	0
CFF07	5	0	PUT-163a-13515860-232	74.1	0
CFF07	5	0	PUT-163a-13515860-234	74.1	0
CFF07	5	0	PUT-163a-13515860-235	74.1	0
CFF03	5	0	PUT-163a-13515860-232	81	0
CFF03	5	0	PUT-163a-13515860-234	81	0
CFF03	5	0	PUT-163a-13515860-235	81	0
CFD11	5	0	PZB01689.1	82.9	0
CFD12	5	0	PZB01689.1	83.9	0
CFD11	5	0	PUT-163a-29544284-1772	84.1	0
CFD03	5	0	SYNGENTA16704	86.3	0
CFD10	5	0	SYNGENTA16704	89.7	0
CFD03	5	0	PUT-163a-101389210-16	90.3	0
CFD06	5	0	PZB01689.1	92.3	0
CFD05	5	0	SYNGENTA16703	94.4	0
CFD05	5	0	SYNGENTA16704	94.4	0
CFD12	5	0	SYNGENTA16704	95	0
CFD12	5	0	SYNGENTA16705	95	0
CFD07	5	0	SYNGENTA16704	96.8	0
CFD05	5	0	PUT-163a-101389210-16	98.4	0
CFF08	5	0	PUT-163a-18172808-1387	98.6	0
CFD02	5	0	SYNGENTA16703	98.7	0
CFD02	5	0	SYNGENTA16704	98.7	0
CFD12	5	0	PUT-163a-101389210-16	99	0
CFD07	5	0	PUT-163a-101389210-16	99.6	0
CFF08	5	0	PUT-163a-29544284-1772	101.3	0
CFF09	5	0	PUT-163a-18172808-1387	101.9	0
CFF09	5	0	PZB01689.1	102.7	0
CFD06	5	0	SYNGENTA16703	104.4	0
CFD06	5	0	SYNGENTA16704	104.4	0
CFD04	5	0	SYNGENTA16704	106.9	0
CFD11	5	0	SYNGENTA16704	109.3	0
CFF07	5	0	PZB01689.1	109.4	0
CFF10	5	0	SYNGENTA16705	113.4	0
CFD01	5	0	SYNGENTA16704	115.5	0
CFD01	5	0	SYNGENTA16705	115.5	0
CFF09	5	0	SYNGENTA16704	117	0
CFF09	5	0	SYNGENTA16705	117	0
CFF02	5	0	SYNGENTA16703	117.9	0
CFF02	5	0	SYNGENTA16705	117.9	0
CFF01	5	0	SYNGENTA16704	118.7	0
CFF01	5	0	SYNGENTA16705	118.7	0
CFD01	5	0	PUT-163a-101389210-16	119.8	0
CFD09	5	0	PHM3630.8	123	0
CFF01	5	0	PUT-163a-101389210-16	123.8	0

CFD03	5	0	PHM3630.8	124.5	0
CFF02	5	0	PUT-163a-101389210-16	125.5	0
CFF07	5	0	SYNGENTA16704	127.8	0
CFF07	5	0	SYNGENTA16705	127.8	0
CFF03	5	0	PUT-163a-29544284-1772	128.1	0
CFF03	5	0	PZB01689.1	128.1	0
CFD12	5	0	PHM3630.8	131.1	0
CFF15	5	0	SYNGENTA16704	137.6	0
CFF15	5	0	SYNGENTA16705	137.6	0
CFD02	5	0	PHM3630.8	140.6	0
CFD07	5	0	PHM3630.8	143.7	0
CFF15	5	0	PUT-163a-101389210-16	148.3	0
CFF03	5	0	SYNGENTA16705	153.5	0
CFF12	5	0	PUT-163a-101389210-16	161.6	0
CFF01	5	0	PHM3630.8	163.9	0
CFF06	5	0	SYNGENTA16704	164.3	0
CFF06	5	0	SYNGENTA16705	165.3	0
CFD01	5	0	PHM3630.8	174.7	0
CFF06	5	0	PHM3630.8	225.7	0
CFD06	6	0	PZE-100000356	10.7	0
CFD06	6	0	PZE-100000438	10.7	0
CFF06	6	0	PZE-100000365	10.9	0
CFD10	6	0	PZE-100000356	11.1	0
CFD10	6	0	PZE-100000438	11.1	0
CFD04	6	0	PZE-100000356	11.5	0
CFD04	6	0	PZE-100000438	11.5	0
CFD09	6	0	PZE-106035881	13	0
CFD02	6	0	PZE-100000356	13.7	0
CFD02	6	0	PZE-100000438	13.7	0
CFF04	6	0	PZE-100000356	14.2	0
CFF04	6	0	PZE-100000438	14.2	0
CFD11	6	0	PZE-100000356	14.6	0
CFD11	6	0	PZE-100000438	14.6	0
CFD06	6	0	ZM013526-0477	15.6	0
CFD03	6	0	PZE-100000356	16.1	0
CFD03	6	0	PZE-100000438	16.1	0
CFD05	6	0	PUT-163a-60396905-2949	16.1	0
CFD05	6	0	PUT-163a-60396905-2950	16.1	0
CFF02	6	0	PZE-100000356	17.1	0
CFF02	6	0	PZE-100000438	17.1	0
CFD02	6	0	PZE-106035881	17.9	0
CFF09	6	0	ZM013526-0477	17.9	0
CFD07	6	0	PZE-100000365	18.8	0
CFF02	6	0	PZE-106035881	20.5	0
CFF01	6	0	PUT-163a-4730575-2150	28.9	0
CFD04	6	0	PUT-163a-4730575-2150	31.4	0
CFF15	6	0	ZM013526-0477	31.6	0
CFD09	6	0	PUT-163a-18163661-1261	31.7	0
CFD09	6	0	PUT-163a-18163661-1262	31.7	0
CFD06	6	0	PUT-163a-18163661-1261	32.1	0
CFD10	6	0	PUT-163a-18163661-1261	32.2	0

CFD11	6	0	PUT-163a-4730575-2150	33.9	0
CFD01	6	0	PUT-163a-4730575-2150	34	0
CFD07	6	0	PUT-163a-4730575-2150	34.7	0
CFD02	6	0	PUT-163a-4730575-2150	38	0
CFD01	6	0	PUT-163a-18163661-1261	38.1	0
CFD03	6	0	PUT-163a-4730575-2150	38.1	0
CFD12	6	0	PUT-163a-18163661-1261	38.5	0
CFD12	6	0	PUT-163a-18163661-1262	38.5	0
CFF08	6	0	PUT-163a-4730575-2150	39.2	0
CFF08	6	0	PUT-163a-4730575-2153	39.2	0
CFD04	6	0	PUT-163a-18163661-1261	39.4	0
CFF01	6	0	PUT-163a-18163661-1261	39.4	0
CFD10	6	0	PUT-163a-60396905-2949	39.5	0
CFD10	6	0	PUT-163a-60396905-2950	39.5	0
CFF06	6	0	PUT-163a-4730575-2150	39.8	0
CFF04	6	0	PUT-163a-4730575-2150	39.9	0
CFD11	6	0	PUT-163a-18163661-1261	41.1	0
CFD11	6	0	PUT-163a-18163661-1262	41.1	0
CFD07	6	0	PUT-163a-18163661-1261	43	0
CFD12	6	0	PUT-163a-60396905-2949	43.5	0
CFD12	6	0	PUT-163a-60396905-2950	43.5	0
CFD04	6	0	PUT-163a-60396905-2949	44.4	0
CFD04	6	0	PUT-163a-60396905-2950	44.4	0
CFF02	6	0	PUT-163a-18163661-1262	44.7	0
CFD07	6	0	PUT-163a-60396905-2950	45.7	0
CFF08	6	0	PUT-163a-18163661-1261	45.9	0
CFF10	6	0	PUT-163a-4730575-2150	46.3	0
CFD02	6	0	PUT-163a-18163661-1261	46.5	0
CFD02	6	0	PUT-163a-18163661-1262	46.5	0
CFD03	6	0	PUT-163a-18163661-1261	49.2	0
CFD02	6	0	PUT-163a-60396905-2949	50.7	0
CFD02	6	0	PUT-163a-60396905-2950	50.7	0
CFF02	6	0	PUT-163a-60396905-2949	51.4	0
CFF02	6	0	PUT-163a-60396905-2950	51.4	0
CFF06	6	0	PUT-163a-18163661-1262	53.4	0
CFD03	6	0	PUT-163a-60396905-2949	54.2	0
CFD03	6	0	PUT-163a-60396905-2950	54.2	0
CFD05	6	0	PUT-163a-71769572-3542	54.6	0
CFF12	6	0	PUT-163a-60396905-2950	55.6	0
CFF04	6	0	PUT-163a-60396905-2949	56	0
CFF04	6	0	PUT-163a-60396905-2950	56	0
CFF10	6	0	PUT-163a-60396905-2950	56.5	0
CFF06	6	0	PUT-163a-60396905-2949	61	0
CFF06	6	0	PUT-163a-60396905-2950	61	0
CFF03	6	0	PUT-163a-4730575-2150	63.8	0
CFF03	6	0	PUT-163a-4730575-2153	63.8	0
CFF13	6	0	PUT-163a-60396905-2950	64.3	0
CFF09	6	0	PUT-163a-71769572-3542	81.2	0
CFF01	6	0	PUT-163a-71769572-3542	86.3	0
CFD01	6	0	PUT-163a-71769572-3542	88.2	0
CFD03	6	0	PUT-163a-71769572-3542	91.2	0

CFF10	6	0	PUT-163a-71769572-3542	92.2	0
CFF02	6	0	PUT-163a-71769572-3542	92.3	0
CFD06	6	0	PUT-163a-71769572-3542	93.2	0
CFD04	6	0	PUT-163a-71769572-3542	95.5	0
CFF06	6	0	PUT-163a-71769572-3542	96.3	0
CFD11	7	0	PUT-163a-89757491-4674	25.8	0
CFD06	7	0	PUT-163a-89757491-4674	26.2	0
CFD02	7	0	PUT-163a-89757491-4674	29	0
CFD05	7	0	PUT-163a-89757491-4674	31.4	0
CFD03	7	0	PUT-163a-89757491-4674	39.2	0
CFF15	7	0	PUT-163a-89757491-4674	41.3	0
CFF02	7	0	PZA00153.3	43	0
CFF02	7	0	PZA00153.7	43	0
CFD03	7	0	PZA00153.3	44.4	0
CFD03	7	0	PZA00153.7	44.4	0
CFF09	7	0	PUT-163a-89757491-4674	44.8	0
CFF09	7	0	PUT-163a-18180613-1496	45.6	0
CFF09	7	0	PZA00153.3	45.6	0
CFF09	7	0	PZA00153.7	45.6	0
CFD12	7	0	PZA00153.3	45.8	0
CFD12	7	0	PZA00153.7	45.8	0
CFF08	7	0	PUT-163a-89757491-4674	46.4	0
CFF08	7	0	PZA00153.3	46.4	0
CFF08	7	0	PZA00153.7	46.4	0
CFD10	7	0	PUT-163a-89757491-4674	46.7	0
CFF15	7	0	PUT-163a-18180613-1496	47.6	0
CFF01	7	0	PUT-163a-89757491-4674	48.7	0
CFD04	7	0	PUT-163a-89757491-4674	48.8	0
CFF07	7	0	PUT-163a-89757491-4674	49.3	0
CFD12	7	0	PUT-163a-18180613-1496	49.8	0
CFF04	7	0	PZA00153.3	49.8	0
CFF04	7	0	PZA00153.7	49.8	0
CFF10	7	0	PUT-163a-89757491-4674	49.9	0
CFD09	7	0	PUT-163a-89757491-4674	50.2	0
CFD09	7	0	PZA00153.3	50.2	0
CFD09	7	0	PZA00153.7	50.2	0
CFF01	7	0	PZA00153.3	50.7	0
CFF01	7	0	PZA00153.7	50.7	0
CFF10	7	0	PZA00153.3	50.8	0
CFF10	7	0	PZA00153.7	50.8	0
CFD04	7	0	PZA00153.3	51.8	0
CFD04	7	0	PZA00153.7	51.8	0
CFF04	7	0	PUT-163a-18180613-1496	51.8	0
CFF08	7	0	PUT-163a-18180613-1496	52	0
CFF07	7	0	PZA00153.3	52.5	0
CFF07	7	0	PZA00153.7	52.5	0
CFF07	7	0	PUT-163a-18180613-1496	53.3	0
CFD10	7	0	PZA00153.3	53.9	0
CFD10	7	0	PZA00153.7	53.9	0
CFF06	7	0	PUT-163a-89757491-4674	54.5	0
CFD01	7	0	PUT-163a-89757491-4674	55.9	0

CFD01	7	0	PZA00153.3	57.5	0
CFD01	7	0	PZA00153.7	57.5	0
CFD02	7	0	PUT-163a-28982443-1665	60.1	0
CFD02	7	0	PUT-163a-28982443-1666	60.1	0
CFD02	7	0	PUT-163a-28982443-1667	60.1	0
CFD06	7	0	PUT-163a-28982443-1665	60.6	0
CFD06	7	0	PUT-163a-28982443-1666	60.6	0
CFD06	7	0	PUT-163a-28982443-1667	60.6	0
CFD07	7	0	PUT-163a-28982443-1665	69.5	0
CFD07	7	0	PUT-163a-28982443-1666	69.5	0
CFD07	7	0	PUT-163a-28982443-1667	69.5	0
CFD09	7	0	PUT-163a-28982443-1665	69.6	0
CFD09	7	0	PUT-163a-28982443-1666	69.6	0
CFD09	7	0	PUT-163a-28982443-1667	69.6	0
CFD11	7	0	PUT-163a-28982443-1665	81.9	0
CFD11	7	0	PUT-163a-28982443-1666	81.9	0
CFD11	7	0	PUT-163a-28982443-1667	81.9	0
CFD12	7	0	PUT-163a-28982443-1665	82.3	0
CFD12	7	0	PUT-163a-28982443-1666	82.3	0
CFF02	7	0	PUT-163a-28982443-1665	91.8	0
CFF02	7	0	PUT-163a-28982443-1666	91.8	0
CFF02	7	0	PUT-163a-28982443-1667	91.8	0
CFF04	7	0	PUT-163a-28982443-1665	97.6	0
CFF04	7	0	PUT-163a-28982443-1666	97.6	0
CFF04	7	0	PUT-163a-28982443-1667	97.6	0
CFF06	7	0	PUT-163a-28982443-1666	112.1	0
CFD02	8	0	PZA03370.1	0	0
CFD03	8	0	PZA03370.1	0	0
CFD04	8	0	PZA03370.1	0	0
CFD05	8	0	PZA03370.1	0	0
CFF01	8	0	PZA03370.1	0	0
CFD09	8	0	PUT-163a-60352761-2693	16.8	0
CFF04	8	0	PUT-163a-60352761-2693	18.1	0
CFD05	8	0	PUT-163a-60352761-2693	20.3	0
CFF06	8	0	PUT-163a-60352761-2693	22	0
CFD11	8	0	PUT-163a-60352761-2693	25.8	0
CFD12	8	0	PUT-163a-60352761-2693	25.8	0
CFF15	8	0	PZE-108051160	31	0
CFF01	8	0	PUT-163a-60352761-2693	32.2	0
CFF02	8	0	PUT-163a-60352761-2693	34.1	0
CFF07	8	0	PUT-163a-60352761-2693	39	0
CFD05	8	0	PZE-108051160	43.4	0
CFD05	8	0	SYNGENTA17266	44.7	0
CFF08	8	0	PZE-108051160	45	0
CFF09	8	0	PZE-108051160	46.8	0
CFF04	8	0	SYNGENTA17266	50.5	0
CFF06	8	0	PZA00452.4	53.5	0
CFD02	8	0	SYNGENTA17266	53.6	0
CFD05	8	0	ZM013785-0420	54	0
CFD03	8	0	ZM013785-0420	55.4	0
CFD10	8	0	ZM013785-0420	57.7	0

CFF06	8	0	SYNGENTA17266	57.7	0
CFF10	8	0	PZE-108051160	57.7	0
CFD06	8	0	SYNGENTA17266	57.9	0
CFD09	8	0	ZM013785-0420	57.9	0
CFD05	8	0	PZE-108072680	58	0
CFD07	8	0	SYNGENTA17266	58	0
CFF13	8	0	PZE-108051160	60	0
CFF07	8	0	SYNGENTA17266	60.7	0
CFD12	8	0	ZM013785-0420	61.8	0
CFD04	8	0	ZM013785-0420	61.9	0
CFD11	8	0	ZM013785-0420	63.3	0
CFF10	8	0	SYNGENTA17266	64	0
CFD02	8	0	ZM013785-0420	64.8	0
CFD07	8	0	ZM013785-0420	65	0
CFD02	8	0	PZE-108072680	66.2	0
CFF02	8	0	SYNGENTA17266	67.9	0
CFF03	8	0	PZE-108051160	68.7	0
CFD06	8	0	ZM013785-0420	70.8	0
CFD01	8	0	ZM013785-0420	75.6	0
CFF01	8	0	ZM013785-0420	81.8	0
CFF02	8	0	PZE-108072680	81.8	0
CFD03	8	0	ZM014127-0216	88.4	0
CFF09	8	0	ZM014127-0216	91.2	0
CFD04	8	0	ZM014127-0216	97.2	0
CFD10	8	0	ZM014127-0216	97.7	0
CFF10	8	0	ZM014127-0216	111.4	0
CFF04	8	0	ZM014127-0216	120.3	0
CFF01	8	0	ZM014127-0216	122.9	0
CFF15	8	0	ZM014127-0216	123.1	0
CFF02	8	0	ZM014127-0216	124.2	0
CFF06	8	0	ZM014127-0216	124.2	0
CFD01	8	0	ZM014127-0216	125.3	0
CFF12	8	0	ZM014127-0216	126.8	0
CFF04	9	0	PZE-109029454	23.8	0
CFF04	9	0	PZE-109029455	23.8	0
CFF04	9	0	PZE-109029456	23.8	0
CFD05	9	0	PZA03036.23	33.6	0
CFF04	9	0	ZM012517-0430	36.2	0
CFD05	9	0	ZM011231-0277	40.2	0
CFD10	9	0	PZA03036.23	45.2	0
CFF04	9	0	ZM012184-0473	45.3	0
CFF09	9	0	PZE-109029454	45.9	0
CFF09	9	0	PZE-109029455	45.9	0
CFF09	9	0	PZE-109029456	45.9	0
CFD10	9	0	PZE-109029454	46.2	0
CFD10	9	0	PZE-109029456	46.2	0
CFD04	9	0	PZE-109029455	46.7	0
CFD02	9	0	PZA03036.23	49	0
CFD02	9	0	PZE-109029455	49	0
CFF08	9	0	PUT-163a-14245519-344	49.5	0
CFD12	9	0	PZA03036.23	50.2	0

CFD12	9	0	PZE-109029455	50.2	0
CFF01	9	0	PZE-109029454	50.8	0
CFF01	9	0	PZE-109029456	50.8	0
CFF10	9	0	PZE-109029454	51	0
CFD07	9	0	PZE-109029455	52.3	0
CFD02	9	0	ZM011231-0277	53.2	0
CFD12	9	0	ZM012517-0430	53.2	0
CFD09	9	0	PZA03036.23	53.7	0
CFD09	9	0	PZE-109029454	53.7	0
CFD09	9	0	PZE-109029456	53.7	0
CFF15	9	0	PZE-109029454	53.8	0
CFF15	9	0	PZE-109029455	53.8	0
CFF15	9	0	PZE-109029456	53.8	0
CFD12	9	0	ZM011231-0277	54.2	0
CFF09	9	0	ZM012517-0430	54.5	0
CFD02	9	0	PZE0008250961	54.6	0
CFD02	9	0	PZE0008411093	54.6	0
CFD02	9	0	PZE-100002267	54.6	0
CFD02	9	0	PZE-100002298	54.6	0
CFD04	9	0	ZM012517-0430	54.7	0
CFD03	9	0	PZE-109029455	55.1	0
CFD06	9	0	PZE-109029454	56.5	0
CFD06	9	0	PZE-109029456	56.5	0
CFF13	9	0	PZA03036.23	56.9	0
CFF13	9	0	PZE-109029454	56.9	0
CFF10	9	0	ZM011231-0277	58	0
CFF10	9	0	ZM012517-0430	58	0
CFD10	9	0	ZM011231-0277	58.4	0
CFD10	9	0	ZM012517-0430	58.4	0
CFF07	9	0	PZE-109029454	58.6	0
CFF10	9	0	PZE0008411093	59	0
CFF10	9	0	PZE-100002298	59	0
CFD07	9	0	ZM011231-0277	59.3	0
CFD09	9	0	ZM012517-0430	59.6	0
CFF03	9	0	PZE-109029454	60	0
CFF03	9	0	PZE-109029455	60	0
CFF03	9	0	PZE-109029456	60	0
CFD03	9	0	ZM012517-0430	61.1	0
CFD07	9	0	PZE-100002267	61.2	0
CFF02	9	0	PZE-109029454	62.3	0
CFF02	9	0	PZE-109029455	62.5	0
CFF02	9	0	PZE-109029456	62.5	0
CFD04	9	0	PZE-109075522	62.6	0
CFF15	9	0	ZM012517-0430	62.8	0
CFD01	9	0	PZE-109029454	65.2	0
CFD01	9	0	PZE-109029456	65.2	0
CFF01	9	0	ZM012517-0430	67.2	0
CFD06	9	0	ZM011231-0277	67.4	0
CFF07	9	0	ZM012517-0430	69.8	0
CFD05	9	0	SYNGENTA16911	71.2	0
CFF01	9	0	ZM011231-0277	71.4	0

CFF02	9	0	ZM012517-0430	71.8	0
CFF15	9	0	PZE0008411093	72.8	0
CFF15	9	0	PZE-100002298	72.8	0
CFF13	9	0	ZM012517-0430	73.2	0
CFF15	9	0	PZE-109075522	73.7	0
CFF02	9	0	PZE0008250961	74.8	0
CFF02	9	0	PZE-100002267	74.8	0
CFF01	9	0	PZE0008411093	75.4	0
CFF01	9	0	PZE-100002298	75.4	0
CFD01	9	0	ZM011231-0277	76.7	0
CFD01	9	0	ZM012517-0430	76.7	0
CFD01	9	0	PZE0008411093	80.3	0
CFD01	9	0	PZE-100002298	80.3	0
CFF06	9	0	ZM012517-0430	81.5	0
CFF06	9	0	ZM011231-0277	83.9	0
CFF06	9	0	PZE0008250961	86.4	0
CFF06	9	0	PZE-100002267	86.4	0
CFD10	9	0	SYNGENTA16911	89.5	0
CFF10	9	0	PUT-163a-14245519-344	92.9	0
CFD07	9	0	PUT-163a-14245519-344	93.2	0
CFD03	9	0	SYNGENTA16911	94.2	0
CFF07	9	0	PUT-163a-14245519-344	95.1	0
CFD06	9	0	PUT-163a-14245519-344	104.1	0
CFF13	9	0	PUT-163a-14245519-344	114.1	0
CFF03	9	0	PUT-163a-14245519-344	127.8	0
CFF10	10	0	ZM013298-0288	0	0
CFD12	10	0	ZM013298-0288	1	0
CFD02	10	0	PUT-163a-28930802-1645	42.6	0
CFF02	10	0	PUT-163a-28930802-1645	61	0
CFF06	10	0	PUT-163a-28930802-1645	67.3	0
CFD06	10	0	SYNGENTA16568	81.5	0
CFF09	10	0	SYNGENTA16568	87.3	0
CFF08	10	0	PZA02167.2	104.8	0
CFF08	10	0	SYNGENTA16568	104.8	0
CFD12	10	0	PZA02167.2	109.8	0
CFD12	10	0	SYNGENTA16568	109.8	0
CFF06	10	0	PZA02167.2	118.3	0

Table S5

type "T" means translocated segment between the genetic map and the B73 genome assembly
type "C" means complex rearrangement of marker orders between the genetic map and the B73 genome assembly
start: B73 genome coordinate of the beginning of the non-colinear region
stop: B73 genome coordinate of the end of the non-colinear region
mrkStart: first marker of the non-colinear region
mrkStop: last marker of the non-colinear region

map	chr	type	start	stop	mrkStart	mrkStop
CFD01	1	T	52.302	52.966	PZE-101069206	SYN122
CFD01	1	T	70.907	82.556	PZE-101083422	PZE-101090535
CFD01	1	T	91.494	91.575	PZE-101099594	PZE-101099581
CFD01	1	T	92.12	92.558	PZE-101094236	SYN13723
CFD01	2	C	170.194	175.921	PZE-102123185	SYN9557
CFD01	2	T	196.262	221.162	PZE-102149548	ZM012288-0143
CFD01	2	C	235.08	236.971	PZE-102192857	SYN6910
CFD01	3	C	16.661	45.806	PZE-103024784	PZE-103044948
CFD01	3	C	83.13	113.323	PZE-103069222	PZE-103059383
CFD01	4	T	132.264	136.594	PZE-104067023	PUT-163a-60397057-2952
CFD01	4	T	187.973	188.405	PZE-104112784	SYN3580
CFD01	5	C	1.434	136.374	PZE-105001734	PHM662.27
CFD01	5	C	189.256	195.492	PZE-105132863	PZE-105134565
CFD01	6	T	108.157	111.244	PZE-106059425	PUT-163a-18168669-1321
CFD01	6	T	111.244	111.818	PZE-106057592	PZE-106057740
CFD01	6	T	118.109	118.427	SYN29431	PZE-106066077
CFD01	7	C	103.918	154.097	PZE-107053089	PZE-107040665
CFD01	9	T	7.512	7.681	SYN32602	SYN32614
CFD01	9	T	12.85	13.394	PZE-109012469	PZE-109012923
CFD01	9	C	86.521	107.1	PZE-109049841	PZE-109063960
CFD01	10	T	126.081	126.317	SYN18413	PZE-110069267
CFD02	1	T	3.32	3.559	PZE-101003115	SYN9365
CFD02	1	T	65.823	66.876	SYN36408	SYN6003
CFD02	1	T	81.234	82.845	PZE-101089901	PZE-101090535
CFD02	2	T	196.673	221.367	PZE-102151093	PZE-102178400
CFD02	3	T	40.385	42.008	PZE-103042413	PZE-103042714
CFD02	3	T	83.687	112.009	PZE-103068739	PZE-103060290
CFD02	4	T	236.142	236.294	SYN23995	SYN23997
CFD02	5	C	189.014	195.492	PZE-105132778	PZE-105134565
CFD02	6	T	109.176	111.338	PZE-106060557	PUT-163a-31909945-2001
CFD02	7	C	84.44	154.097	PZE-107042960	PZE-107040665
CFD02	8	T	123.481	124.1	PZE-108070686	PZE-108070885
CFD02	9	T	15.336	15.505	SYN10998	PZE-109015397
CFD02	9	T	102.461	108.938	PZE-109060940	PZE-109063960
CFD03	1	T	2.525	2.846	SYN35329	SYN38926
CFD03	1	T	79.586	82.556	SYN23488	PZE-101090535
CFD03	3	C	14.862	22.536	PZE-103024864	SYN18361
CFD03	3	C	22.536	32.237	PZE-103031314	PZE-103037256
CFD03	4	T	132.509	136.594	PZE-104067296	PUT-163a-60397057-2952
CFD03	5	T	16.594	17.844	PZE-105031680	PZE-105031848
CFD03	5	T	189.3	195.964	PZE-105133024	PZE-105129902
CFD03	6	T	18.532	21.806	PZE-106021260	PZE-106007446
CFD03	6	C	106.677	111.496	PZE-106057989	PUT-163a-18168669-1321
CFD03	7	C	62.8	119.254	PZE-107048079	SYN18271

CFD03	7	T	125.492	138.126	PZE-107083381	PZE-107082619
CFD03	9	C	89.193	107.103	PZE-109051750	PZE-109063960
CFD04	1	T	52.326	52.966	PZE-101069307	SYN122
CFD04	1	T	66.289	67.59	PZE-101081952	SYN24908
CFD04	1	T	75.093	82.556	PZE-101086195	PZE-101090535
CFD04	2	T	234.926	236.971	PZE-102192857	SYN6910
CFD04	3	C	15.714	20.226	PZE-103024864	PZE-103026083
CFD04	3	C	33.06	45.139	SYN9954	PZE-103044762
CFD04	3	T	48.831	51.409	PZE-103047230	SYN937
CFD04	3	T	83.13	114.001	PZE-103069222	PZE-103059383
CFD04	3	T	230.392	232.011	SYN3326	PUT-163a-18170300-1354
CFD04	5	T	180.304	182.134	PZE-105124575	SYN9153
CFD04	6	C	106.677	111.496	PZE-106057989	PUT-163a-18168669-1321
CFD04	6	T	122.639	123.103	SYN33891	SYN33888
CFD04	7	C	125.492	138.126	PZE-107083381	PZE-107082619
CFD04	9	T	83.488	107.103	PZE-109048473	PZE-109063960
CFD05	1	T	65.551	65.843	SYN3989	PZE-101080722
CFD05	1	T	73.326	82.556	PZE-101085136	PZE-101090535
CFD05	1	T	281.479	286.758	SYN14771	PZE-101239020
CFD05	2	T	151.216	175.235	ZM013834-0560	PZE-102125779
CFD05	2	T	235.228	236.971	PUT-163a-110194326-51	SYN6910
CFD05	4	T	236.142	236.294	SYN23995	SYN23997
CFD05	5	C	188.647	195.492	SYN35847	PZE-105134565
CFD05	7	C	125.492	138.126	PZE-107083381	PZE-107082619
CFD05	9	T	74.49	107.104	PZE-109044159	PZE-109063960
CFD06	1	C	0.327	4.38	PZE-101000169	SYN5936
CFD06	1	T	65.823	66.876	SYN36408	SYN6003
CFD06	1	T	287.141	287.72	PZB02513.3	SYN37684
CFD06	2	T	195.629	221.367	PZE-102149235	PZE-102178400
CFD06	2	T	235.08	236.894	PZE-102192857	SYN6910
CFD06	3	C	9.328	17.342	PZE-103016732	PZE-103024784
CFD06	3	C	22.8	36.966	PZE-103031314	SYN20171
CFD06	3	T	176.058	176.792	PZE-103117542	SYN17589
CFD06	3	T	176.792	178.168	PZE-103118331	PZE-103119393
CFD06	3	T	230.572	232.041	SYN3326	PUT-163a-18170300-1353
CFD06	4	C	25.934	91.86	PZE-104023433	PZE-104026198
CFD06	5	T	0.883	2.162	SYN33185	SYN35352
CFD06	5	T	164.05	168.398	PZE-105107790	PZE-105110883
CFD06	5	T	168.871	176.165	SYN34468	PZE-105119280
CFD06	5	T	188.716	195.216	PZE-105132637	PZE-105129902
CFD06	6	C	106.754	111.244	PZE-106058148	PUT-163a-18168669-1321
CFD06	6	T	111.244	111.818	PZE-106057592	PZE-106057733
CFD06	6	T	155.319	155.828	PZE-106104150	SYN23881
CFD06	6	C	155.828	160.657	SYNGENTA17033	ZM011303-0272
CFD06	7	T	0.303	1.647	SYN23678	PUT-163a-21481304-1570
CFD06	7	C	125.492	138.126	PZE-107083381	PZE-107082619
CFD06	9	C	43.889	107.1	PZA03097.4	PZE-109063960
CFD06	10	T	93.808	93.972	PZE-110049981	PZE-110049820
CFD07	1	T	74.601	82.556	PZE-101085526	PZE-101090535
CFD07	2	T	202.51	221.162	SYN20465	ZM012288-0143
CFD07	3	C	12.545	16.357	PZE-103020378	SYN26605
CFD07	3	C	18.054	124.606	PZE-103025888	PZE-103074862
CFD07	5	C	188.647	195.49	SYN35847	PZE-105134617

CFD07	6	T	107.552	111.244	SYN35096	PUT-163a-18168669-1321
CFD07	7	C	115.185	154.097	PZE-107059758	PZE-107040665
CFD07	9	T	96.868	108.938	PZE-109056240	PZE-109063960
CFD09	1	T	51.836	52.966	PZE-101069206	SYN122
CFD09	1	T	70.649	82.556	PZE-101083051	PZE-101090535
CFD09	2	C	195.814	221.367	PZE-102149810	PZE-102178400
CFD09	5	C	187.991	195.49	PZE-105132637	PZE-105134617
CFD09	6	T	108.61	111.133	PZE-106059969	PUT-163a-31909945-2001
CFD09	7	C	125.492	137.947	PZE-107083381	PZE-107082619
CFD09	9	C	88.988	107.1	PZE-109055561	PZE-109063960
CFD10	1	T	79.586	82.556	SYN23488	PZE-101090535
CFD10	4	C	0.029	0.805	PZE-104000008	PZE-104000566
CFD10	5	T	152.793	154.91	PZE-105101822	PZE-105101867
CFD10	5	T	191.237	195.742	PZE-105135639	PZE-105134617
CFD10	6	T	108.731	111.496	PZE-106060570	PUT-163a-18168669-1321
CFD10	7	C	125.492	137.99	PZE-107083381	PZE-107082619
CFD10	9	C	70.888	107.104	PZE-109043894	PZE-109063960
CFD11	1	T	70.653	115.309	PZE-101083105	PZE-101090535
CFD11	3	C	55.778	121.666	PZE-103051529	PZE-103073425
CFD11	5	T	1.389	137.69	SYN2372	PHM662.27
CFD11	6	C	24.608	34.032	PZE-106008617	SYN11180
CFD11	6	T	74.652	83.094	PZE-106032478	PZA03069.4
CFD11	6	T	108.376	111.686	PZE-106060557	PUT-163a-18168669-1321
CFD11	7	C	125.389	137.947	PZE-107083381	PZE-107082619
CFD11	8	T	123.481	124.46	PZE-108070686	PZE-108070885
CFD11	9	T	53.154	70.831	PZE-109038367	PZE-109042232
CFD11	9	T	89.128	119.759	PZE-109055660	SYN36627
CFD11	10	T	63.925	124.429	SYN18547	PZE-110067890
CFD12	1	T	71.166	82.556	SYN14652	PZE-101090535
CFD12	2	T	6.248	6.846	PZE-102015152	SYNGENTA1701
CFD12	2	T	194.973	221.604	PZE-102148280	ZM012288-0143
CFD12	2	T	235.383	236.816	PUT-163a-110194326-51	SYN6910
CFD12	3	C	36.763	129.069	SYN20171	PZE-103077287
CFD12	5	T	188.647	195.401	SYN35847	PZE-105129902
CFD12	6	C	106.895	111.29	SYN23925	PUT-163a-18168669-1321
CFD12	7	C	91.583	154.097	SYN29320	PZE-107040665
CFD12	9	T	87.969	107.104	PZE-109051042	PZE-109063960
CFD12	9	T	110.467	112.295	PZE0003716573	PZE0003747944
CFF01	1	T	3.32	3.537	PZE-101003115	SYN9365
CFF01	1	T	70.739	82.556	PZE-101083165	PZE-101090535
CFF01	1	T	198.962	199.208	PZE-101156159	PZE-101156396
CFF01	2	C	174.577	175.921	PUT-163a-74236165-3621	SYN9557
CFF01	2	C	194.765	221.162	PZE-102147990	ZM012288-0143
CFF01	2	T	235.08	236.971	PZE-102192857	SYN6910
CFF01	3	C	16.661	59.709	PZE-103024784	PZE-103052411
CFF01	3	C	83.687	112.735	PZE-103068739	PZE-103059709
CFF01	3	T	228.679	228.903	PZE-103183938	PUT-163a-94473855-4870
CFF01	5	T	0.984	136.374	SYN9892	PHM662.27
CFF01	5	C	188.647	195.492	SYN35847	PZE-105134565
CFF01	6	T	5.299	6.038	PZE-106004214	PZE-106004598
CFF01	6	T	28.137	28.471	SYN36834	PZE-106010241
CFF01	6	T	106.677	111.244	PZE-106057989	PUT-163a-18168669-1321
CFF01	6	T	158.564	158.957	SYN4188	SYN4190

CFF01	7	T	19.404	19.705	SYN3758	SYN3763
CFF01	7	C	100.55	154.097	PZE-107051275	PZE-107040665
CFF01	8	T	26.222	26.491	PZE-108028580	PZE-108028672
CFF01	9	C	87.969	107.1	PZE-109051042	PZE-109063960
CFF01	9	T	124.368	124.64	PZE-109076932	PZE-109076881
CFF02	1	T	6.391	6.85	PZE-101011048	PZE-101011197
CFF02	1	C	8.455	9.056	PZA-000175002	PZA00447.5
CFF02	1	T	9.879	10.786	SYN2431	PZE-101018400
CFF02	1	T	15.282	16.181	SYN22238	PZE-101026621
CFF02	1	T	18.415	18.92	PZE-101030508	PZE-101030616
CFF02	1	C	19.172	22.153	PZE-101031542	PZE-101033801
CFF02	1	C	27.103	30.001	PZE-101040594	PZE-101043682
CFF02	1	T	51.836	53.1	PZE-101069206	SYN122
CFF02	1	C	160.619	173.32	PZE-101127171	SYN2709
CFF02	2	T	14.157	15.557	SYN9584	PZE-102032348
CFF02	2	C	20.27	22.39	SYN9109	PZE-102043418
CFF02	2	C	24.259	27.968	PZE-102047235	PZE-102049498
CFF02	2	C	194.83	221.162	PZE-102148280	ZM012288-0143
CFF02	2	T	226.032	229.209	SYN8350	PZE-102185359
CFF02	3	T	12.915	14.842	PZE-103021174	SYN12453
CFF02	3	T	18.122	19.041	PZE-103025846	PZE-103026023
CFF02	3	C	20.359	22.123	PZE-103028959	SYN18361
CFF02	3	T	32.887	33.311	PZE-103038295	SYN9954
CFF02	3	C	165.374	166.378	PUT-163a-16925568-1102	PZA01396.1
CFF02	3	C	176.545	177.517	PZE-103117610	SYN18160
CFF02	3	C	178.483	179.986	SYN37579	PZE-103120875
CFF02	3	T	183.754	184.218	SYN28564	PZE-103126857
CFF02	3	C	190.254	192.993	SYNGENTA15970	PZE-103136534
CFF02	3	T	196.704	198.373	PZE-103141647	PZE-103142436
CFF02	3	T	198.373	199.133	SYN38174	PZE-103143491
CFF02	3	C	200.865	204.416	PZE-103146956	PZE-103150095
CFF02	3	T	223.322	223.74	PZE-103177674	ZM014594-0272
CFF02	4	T	122.814	136.594	PZE-104062998	PUT-163a-60397057-2952
CFF02	4	C	171.245	171.942	PZE-104094429	SYNGENTA3399
CFF02	4	T	177.846	178.582	PZE-104101436	SYNGENTA12917
CFF02	4	T	179.696	180.948	PZE-104103509	PZE-104104502
CFF02	4	C	220.944	228.192	PZE-104136244	PZE-104139840
CFF02	4	T	231.606	233.074	PZE-104143133	SYN11077
CFF02	4	C	233.074	234.885	PZE-104144389	PZE-104146572
CFF02	4	C	240.291	240.786	SYN8790	PZE-104158009
CFF02	5	C	0.975	136.727	SYN9892	PHM662.27
CFF02	6	C	106.553	111.244	PZE-106057989	PUT-163a-18168669-1321
CFF02	6	C	128.525	129.604	PUT-163a-18167596-1292	PZE-106074105
CFF02	6	C	138.559	141.075	SYN4850	SYN25251
CFF02	6	T	151.609	152.269	SYN35959	SYN16937
CFF02	7	C	99.231	154.132	PZE-107050490	PZE-107040666
CFF02	7	T	155.723	156.376	SYN3467	PZE-107102059
CFF02	7	C	174.108	174.837	SYN34644	PZE-107137132
CFF02	8	C	5.626	6.587	PZE-108005661	SYN10428
CFF02	8	C	13.627	15.025	PZE-108013775	PZA02955.3
CFF02	8	T	167.727	168.563	SYN36533	PZE-108119984
CFF02	8	C	173.101	173.748	PUT-163a-4647150-2111	PZE-108133242
CFF02	9	T	7.507	7.696	SYN32602	PZE-109006730

CFF02	9	T	7.904	8.136	SYN39070	SYN39071
CFF02	9	T	10.839	11.606	PUT-163a-18163412-1251	sh1.1
CFF02	9	C	82.3	107.018	PZE-109047977	PZE-109063841
CFF02	9	T	137.419	138.355	SYNGENTA15232	PZE-109090188
CFF02	9	T	142.972	143.19	SYN30946	PZE-109098198
CFF02	9	T	147.204	148.13	SYN26903	PZE-109106215
CFF02	9	T	153.76	154.203	SYN13929	PZE-109119712
CFF02	9	C	154.505	156.415	PUT-163a-78122776-4410	PZE-109122151
CFF03	1	T	65.499	67.221	SYN36408	SYN6003
CFF03	1	T	223.359	229.13	SYN22253	PZE-101184213
CFF03	2	T	196.867	221.887	SYN6136	ZM012288-0143
CFF03	2	T	234.56	236.431	PZE-102192857	SYN29561
CFF03	4	T	122.161	136.728	SYN26788	PUT-163a-60397057-2952
CFF03	5	T	1.855	136.727	SYN541	PHM662.27
CFF03	5	T	185.657	196.417	PZE-105130338	PZE-105129902
CFF03	5	T	204.586	204.865	SYN1942	PZE-105163109
CFF03	6	T	153.981	154.672	PZE-106102131	SYN24465
CFF03	7	T	0.707	1.067	PZE-107000462	PZE-107000845
CFF03	7	T	81.178	161.367	PZE-107041962	PZE-107040665
CFF03	9	T	7.507	9.579	SYN32602	SYN32614
CFF03	9	T	31.66	107.018	PZE-109029355	PZE-109063841
CFF04	1	T	3.314	3.517	PZE-101003115	SYN9377
CFF04	2	T	196.923	221.887	PZE-102151194	ZM012288-0143
CFF04	4	C	42.637	91.051	PZE-104034056	PZE-104055161
CFF04	4	T	121.276	136.607	PZE-104061691	PUT-163a-60397057-2952
CFF04	5	C	2.61	136.374	SYN22678	PHM662.27
CFF04	5	T	140.183	165.472	PZE-105095939	PZE-105107819
CFF04	5	T	191.079	195.49	PZE-105135342	PZE-105134612
CFF04	6	C	106.895	111.244	SYN23928	PUT-163a-18168669-1321
CFF04	7	C	125.473	137.947	PZE-107083403	PZE-107082619
CFF04	9	T	97.269	107.018	SYN37647	PZE-109063841
CFF04	9	T	110.67	111.301	PZE0003716573	PZE0003747944
CFF06	1	C	0.206	4.895	PZE-101000111	PZE-101006745
CFF06	1	C	189.5	191.138	PZE-101146521	PUT-163a-78106858-4272
CFF06	1	C	194.897	196.733	PZE-101151332	SYN28736
CFF06	1	C	225.348	230.764	PZE-101181333	PZE-101185989
CFF06	1	C	230.764	252.557	PZE-101186097	SYN18315
CFF06	1	C	254.131	265.154	PZE-101205734	PZE-101214376
CFF06	1	C	265.955	280.424	PZE-101215580	PUT-163a-71305340-3082
CFF06	1	C	281.851	284.325	SYN14776	SYN8671
CFF06	1	T	296.507	296.89	SYN26859	PUT-163a-60346998-2564
CFF06	2	T	43.2	44.049	PUT-163a-74246330-3711	PZE-102065854
CFF06	2	C	149.176	167.213	PUT-163a-4226318-2039	PZE-102120732
CFF06	2	C	169.846	179.088	PZE-102122951	PZE-102129011
CFF06	2	T	200.584	221.112	SYNGENTA5309	PZE-102178400
CFF06	2	T	234.925	236.371	PZE-102192857	SYN29561
CFF06	3	T	22.8	25.529	PZE-103031314	PZE-103032119
CFF06	3	T	123.524	127.276	PZE-103074862	PZE-103076395
CFF06	3	C	136.559	143.845	PZE-103082567	SYN31570
CFF06	3	C	149.867	152.127	PZE-103091108	PZE-103091693
CFF06	4	C	27.742	74.717	PZE-104024382	PZE-104048439
CFF06	4	T	74.717	76.125	PZE-104048874	PZE-104049074
CFF06	4	C	76.125	124.437	PZE-104049616	PZE-104062770

CFF06	4	C	130.346	141.084	PZE-104066088	PZE-104070126
CFF06	4	C	164.865	169.762	SYN2280	PZE-104093194
CFF06	4	C	173.652	177.263	SYN2452	SYN2317
CFF06	4	T	182.035	183.663	PZE-104106033	SYN15646
CFF06	4	T	183.663	185.451	PZE-104107169	PZE-104108322
CFF06	4	T	234.688	235.035	PZE-104146988	SYN923
CFF06	4	T	235.206	237.175	SYN13870	SYN14093
CFF06	5	T	10.163	11.806	PZE-105021734	SYN903
CFF06	5	C	14.614	19.609	SYN16500	PUT-163a-76290498-3947
CFF06	5	C	173.775	174.808	SYN34979	SYN37191
CFF06	5	T	192.233	196.053	SYN1851	PZE-105134612
CFF06	5	T	196.053	198.498	PZE-105142621	PZE-105144436
CFF06	5	C	215.249	217.62	SYN32971	PZE-105182657
CFF06	6	C	0.599	6.079	PZE-106000286	PZE-106004598
CFF06	6	C	107.548	111.244	PZE-106058841	PUT-163a-18168669-1321
CFF06	6	T	148.085	149.909	SYN37017	PZE-106093877
CFF06	6	C	151.059	152.631	SYN22585	SYN16940
CFF06	7	C	7.74	8.309	PZE-107011423	SYN24995
CFF06	7	C	11.174	23.122	PZE-107015084	PZE-107022494
CFF06	7	C	111.818	154.097	PZE-107059010	PZE-107040666
CFF06	7	T	158.95	159.859	PZE-107107154	SYN3345
CFF06	7	C	161.921	165.264	PZE-107113180	SYN23691
CFF06	7	T	165.264	166.716	SYN3958	SYN3206
CFF06	8	T	0.167	2.424	PZA02388.1	SYN15862
CFF06	8	T	104.821	106.71	PZE-108058793	PZE-108059570
CFF06	8	C	108.493	112.603	PZE-108060864	PZE-108062231
CFF06	8	C	115.32	124.471	PUT-163a-149026703-794	PZE-108071041
CFF06	8	C	136.622	144.594	PZE-108080946	PZE-108086816
CFF06	8	T	149.943	151.488	PZE-108093081	PZE-108094801
CFF06	9	C	1.138	4.789	PZE-109000616	PZE-109003647
CFF06	9	T	6.065	6.269	PZE-109005548	SYN9428
CFF06	9	T	7.507	7.871	SYN32602	PZE-109006938
CFF06	9	T	12.526	13.672	PZE-109012061	PZE-109012939
CFF06	9	C	25.669	107.104	PZE-109025557	PZE-109063960
CFF06	9	C	152.565	154.018	SYN6090	PZE-109119196
CFF06	9	T	155.039	155.776	PZE-109121297	PZE-109121439
CFF06	10	T	4.297	4.685	SYN14833	SYN4471
CFF07	1	T	2.53	2.94	SYN35333	PUT-163a-16926058-1127
CFF07	2	T	235.08	236.371	PZE-102192857	SYN29561
CFF07	3	C	77.992	113.323	PZE-103069222	PZE-103059383
CFF07	5	T	0.565	2.62	SYN5719	SYN35370
CFF07	6	T	4.517	6.079	PZE-106004307	PZE-106004598
CFF07	6	T	107.038	111.332	SYN35087	PZE-106056816
CFF07	6	T	148.223	148.713	ZM013757-0256	PZE-106092530
CFF07	7	C	125.473	137.947	PZE-107083403	PZE-107082619
CFF07	9	T	7.507	7.681	SYN32602	SYN32614
CFF07	9	C	75.6	107.1	PZE-109044890	PZE-109063960
CFF07	9	C	154.584	156.415	SYN23701	PZE-109122151
CFF08	2	T	196.867	221.887	SYN6136	ZM012288-0143
CFF08	2	T	235.08	236.431	PZE-102192857	SYN29561
CFF08	4	T	236.144	236.215	ZM013866-0286	SYN24018
CFF08	5	C	0.86	2.771	SYN12357	SYN35352
CFF08	5	T	187.716	196.417	SYN16483	PZE-105129902

CFF08	6	T	92.906	96.262	PZE-106044414	PZE-106046532
CFF08	6	T	106.677	111.086	PZE-106057989	PUT-163a-31909945-2001
CFF08	7	C	124.673	137.947	PZE-107083403	PZE-107082619
CFF08	7	T	169.444	171.373	PZE-107128144	PZE-107128846
CFF09	2	T	194.976	221.162	PZE-102149548	ZM012288-0143
CFF09	3	T	83.75	109.29	SYN38653	PZE-103062153
CFF09	3	T	228.217	231.471	PZE-103185177	PUT-163a-18170300-1353
CFF09	5	T	1.383	137.67	PZE-105001734	PHM662.27
CFF09	5	T	206.616	206.778	PZE-105158980	PZE-105159062
CFF09	6	T	108.132	111.244	PZE-106059789	PUT-163a-18168669-1321
CFF09	7	T	0.518	1.047	PZE-107000462	PZE-107000687
CFF09	7	C	125.443	137.947	PZE-107083446	PZE-107082619
CFF09	9	C	88.308	107.018	PZE-109055561	PZE-109063841
CFF10	1	T	3.32	3.517	PZE-101003115	SYN9377
CFF10	1	T	65.958	67.023	PZE-101120107	SYN6003
CFF10	2	C	194.83	221.162	PZE-102148280	ZM012288-0143
CFF10	2	T	234.926	235.987	PZE-102192857	PUT-163a-110194326-51
CFF10	3	T	11.444	12.278	PZE-103019236	PZE-103019668
CFF10	3	T	22.123	22.8	PZE-103029873	PZE-103029988
CFF10	3	T	22.8	25.036	PZE-103031323	PZE-103032315
CFF10	3	C	77.611	113.388	PZE-103069222	PZE-103059383
CFF10	4	T	127.522	136.607	PZE-104064730	PUT-163a-60397057-2952
CFF10	4	T	236.142	236.312	SYN23995	SYN23997
CFF10	5	T	191.968	195.492	PZE-105137112	PZE-105134565
CFF10	6	T	106.782	110.942	PZE-106058198	PZE-106056649
CFF10	7	C	100.433	154.127	PZE-107051275	PZE-107040665
CFF10	9	C	33.946	107.018	PZE-109030320	PZE-109063841
CFF12	1	T	96.368	107.236	PZE-101100465	PZE-101105211
CFF12	2	T	196.233	225.101	PZE-102149655	ZM012288-0143
CFF12	2	T	235.08	236.431	PZE-102192857	SYN29561
CFF12	5	C	152.367	163.798	PZE-105101687	PZE-105106627
CFF12	7	C	3.477	60.688	PZB01083.4	SYN39339
CFF12	7	T	144.488	160.11	PZE-107101793	SYN3345
CFF12	9	T	7.399	9.72	SYN32602	SYN32614
CFF13	1	T	65.842	67.322	SYN36408	SYN6003
CFF13	2	C	5.181	8.913	SYN9295	PUT-163a-4630656-2097
CFF13	2	C	194.976	226.866	PZE-102149548	ZM012288-0143
CFF13	4	T	6.358	8.22	SYN26462	SYN19801
CFF13	4	T	121.516	136.728	SYN26788	PUT-163a-60397057-2952
CFF13	5	T	16.514	17.845	PZE-105031680	PZE-105031901
CFF13	7	C	122.899	137.947	PZE-107083446	PZE-107082619
CFF13	9	T	6.813	7.681	SYN32602	SYN32614
CFF13	9	C	53.154	108.954	PZE-109038367	PZE-109063960
CFF15	1	T	176.989	177.389	ZM001656-0297	SYN9634
CFF15	2	C	194.976	221.162	PZE-102149548	ZM012288-0143
CFF15	2	T	234.925	236.371	PZE-102192857	SYN29561
CFF15	5	T	16.774	17.845	PZE-105031680	PZE-105031901
CFF15	5	T	204.607	204.865	SYN1942	PZE-105163109
CFF15	6	T	0.36	1.983	PZE-106000108	PZE-106000349
CFF15	7	C	125.443	137.947	PZE-107083446	PZE-107082619
CFF15	7	T	171.803	172.378	PZE-107130734	PZE-107130802
CFF15	8	T	117.562	118.768	PZE-108066177	PZA00567.10
CFF15	9	C	44.009	107.018	PZE-109034784	PZE-109063841
