# Supplementary figures and images for: Intraspecific variation of recombination rate in maize
Source: Genome Biol. 2013 Sep 19;14(9):R103. doi: 10.1186/gb-2013-14-9-r103 (PMC4053771; doi:10.1186/gb-2013-14-9-r103)

A

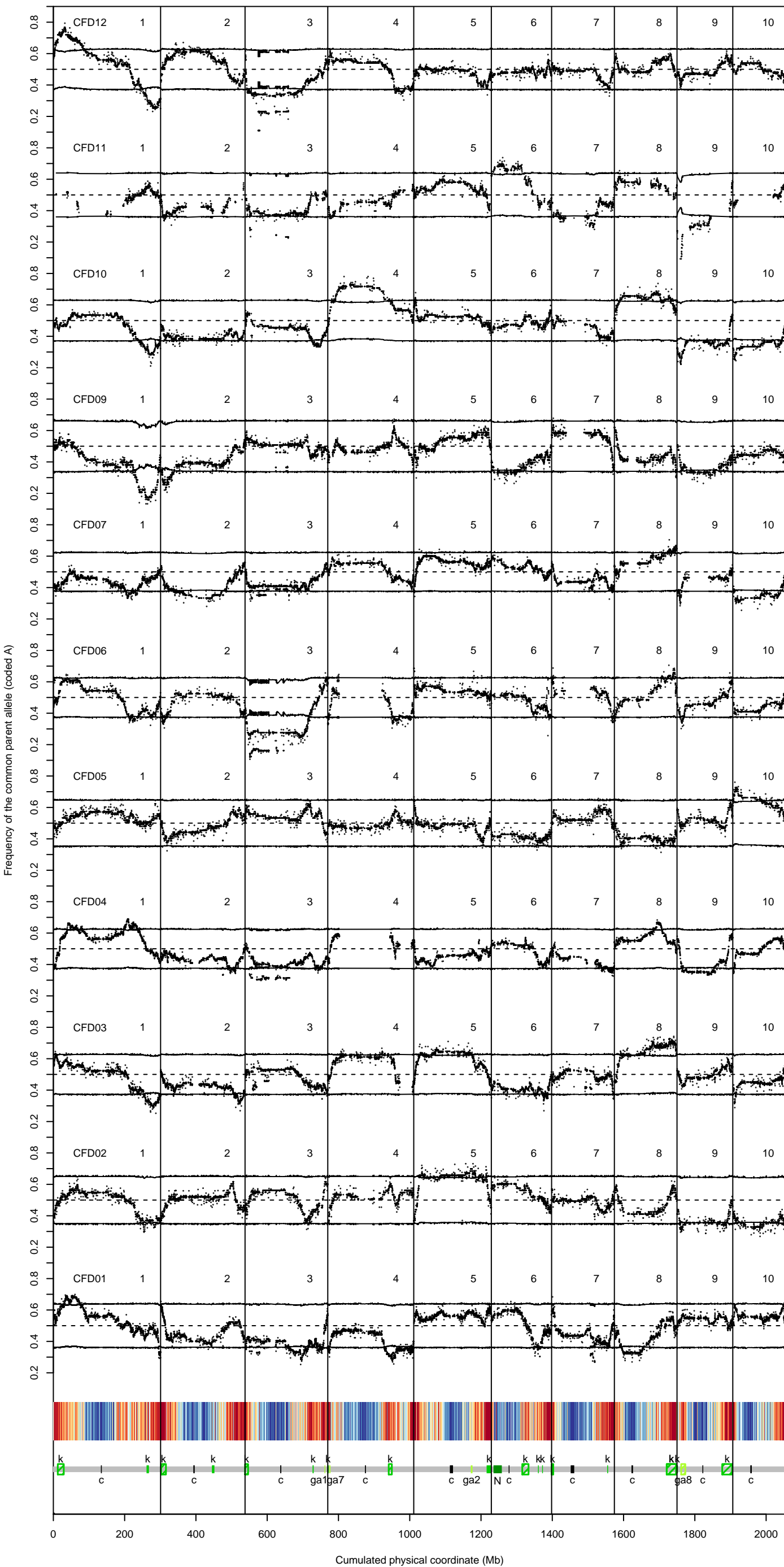

B

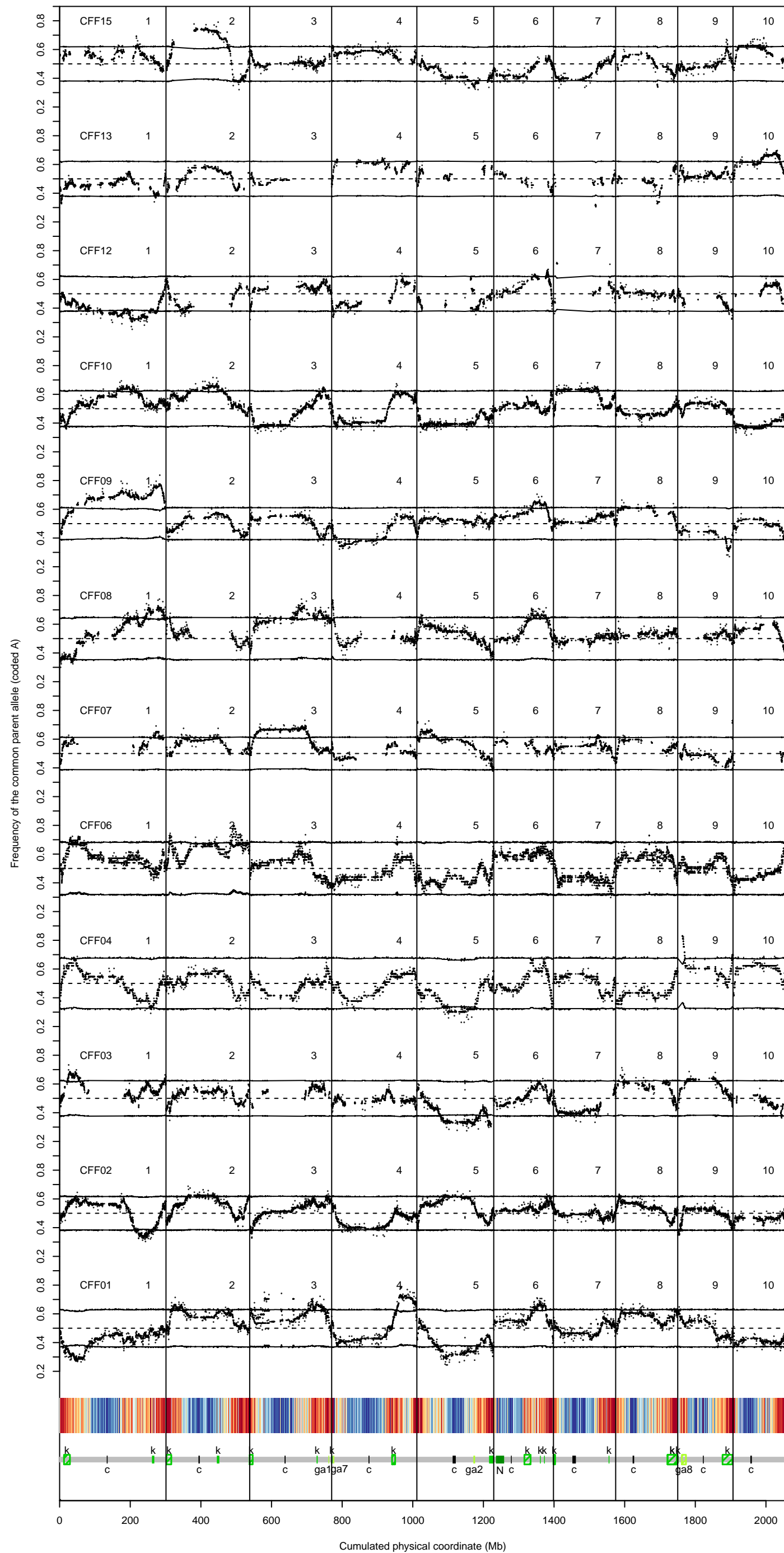

Supplement: Additional file 5: Figure S1 — Allele frequency of the central parent allele for all polymorphic markers (A) in all Dent populations and (B) in all Flint populations. The 10 chromosomes are represented along the same horizontal axis. The expected frequency of 0.5 is indicated by a dotted line, and surrounded by solid lines representing its 99% confidence intervals. The x-axis indicates physical coordinates in megabase pairs along the B73 genome. In the bottom of the figure, the heat map represents gene density (low for cold colors and high for hot colors). Gray horizontal line below the heat-map: sketch of the chromosome organization showing centromeres (cen), knobs, nucleolar organizer region (NOR), and known gametophytic factors (gax) (from [30]). Color filling of chromosome features is solid when the estimated boundaries of the region are known, and hatched when the box indicates only the extremities of the bin containing the region. [file gb-2013-14-9-r103-S5.pdf]

# CFD01 chromosome 1

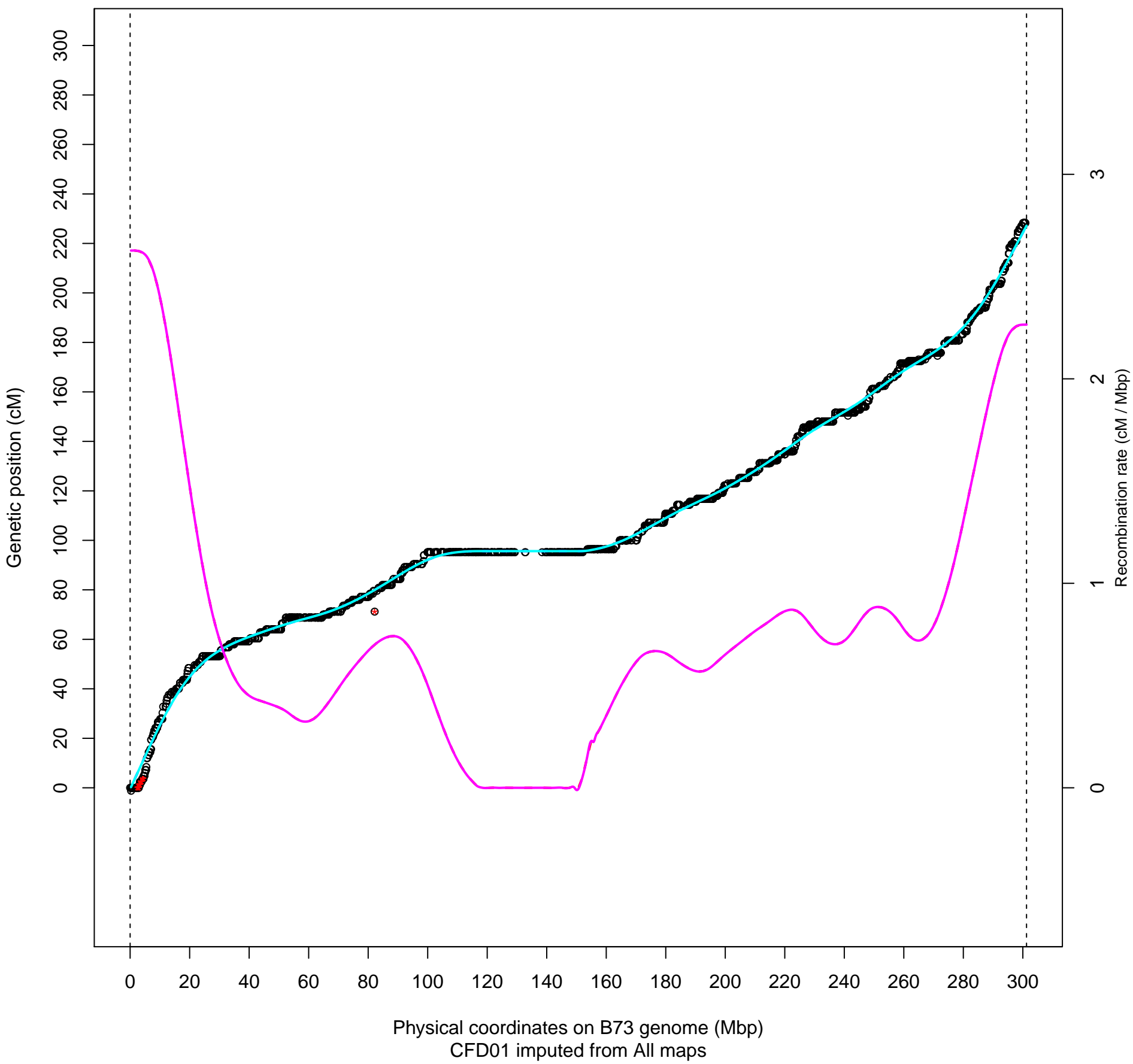

# CFF07 chromosome 1

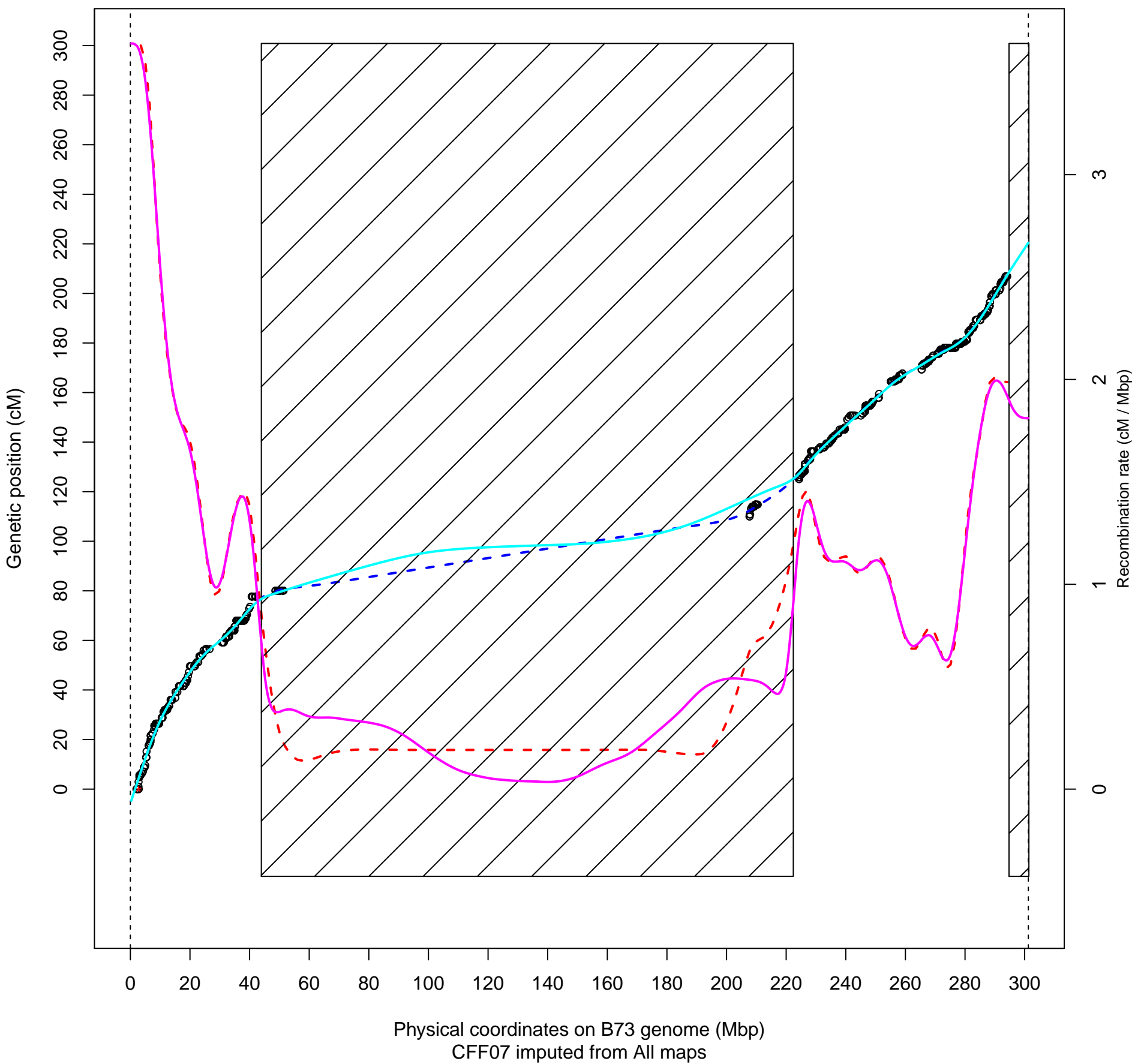

# CFF01 chromosome 3

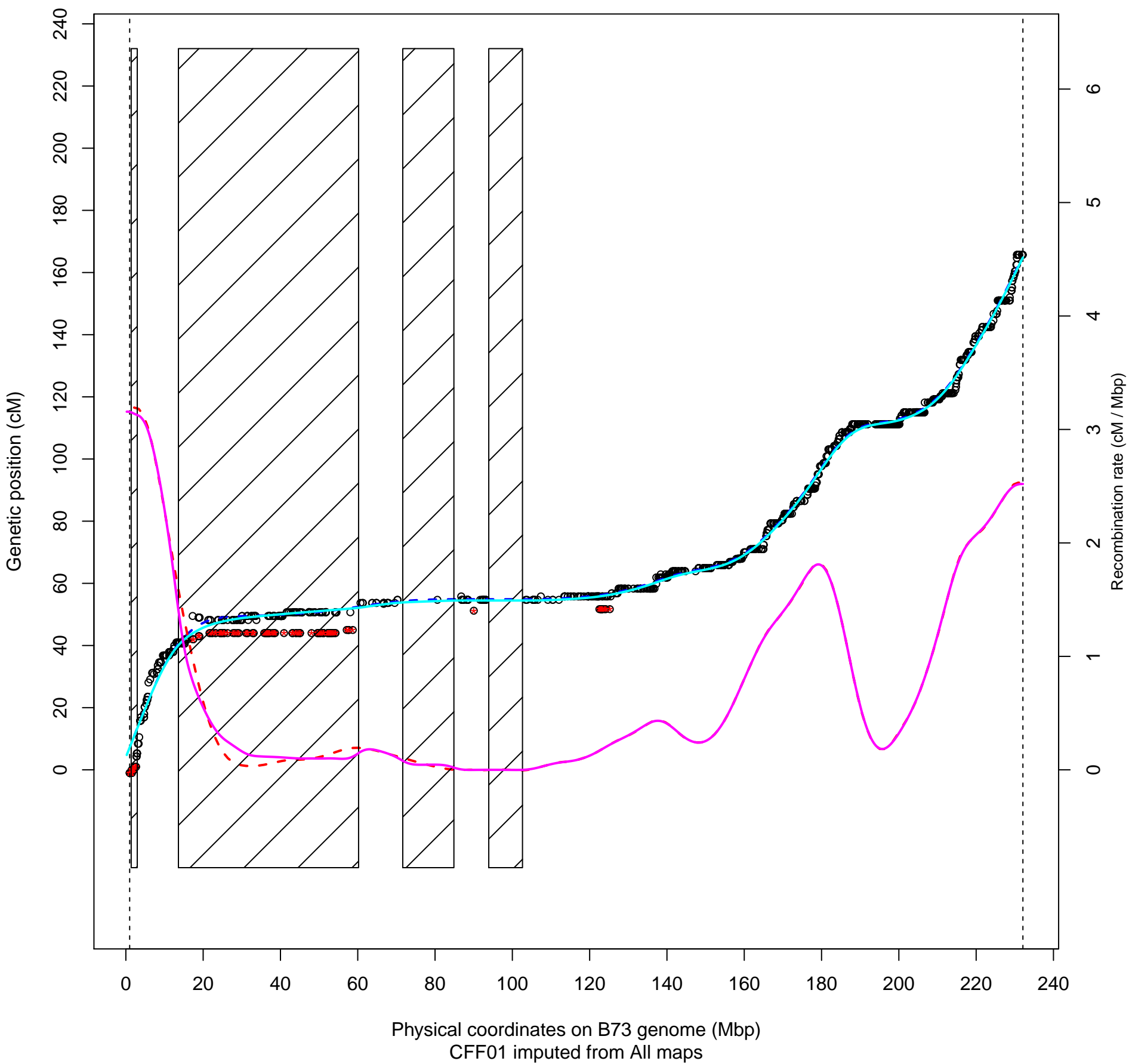

Supplement: Additional file 7: Figure S3 — Marey maps and recombination landscapes along the chromosomes for three examples illustrating the imputation of regions with missing or unreliable data: CFD01 chromosome 1, CFF07 chromosome 1, and CFF01 chromosome 3. The x-axis indicates physical position of the SNPs on the B73 physical map in megabase pairs. The left y-axis indicates genetic map position in centiMorgans. The right y-axis indicates recombination rate in cM/Mbp. Each black empty circle corresponds to a SNP. Red dots indicate the outlier markers removed from the smoothing analysis. Dark blue dotted line: smoothed Marey map. Red dotted line: first derivative of the smoothed Marey map. Hatched rectangles: regions masked when going from bare to masked Marey maps (see Materials and methods). Light blue solid line: imputed smoothed Marey map obtained after imputation in the excluded regions, using data from all maps pooled. Pink solid curve: recombination rate computed as the first derivative of the imputed smoothed Marey map. [file gb-2013-14-9-r103-S7.pdf]

# Interference intensity $nu$ in P1

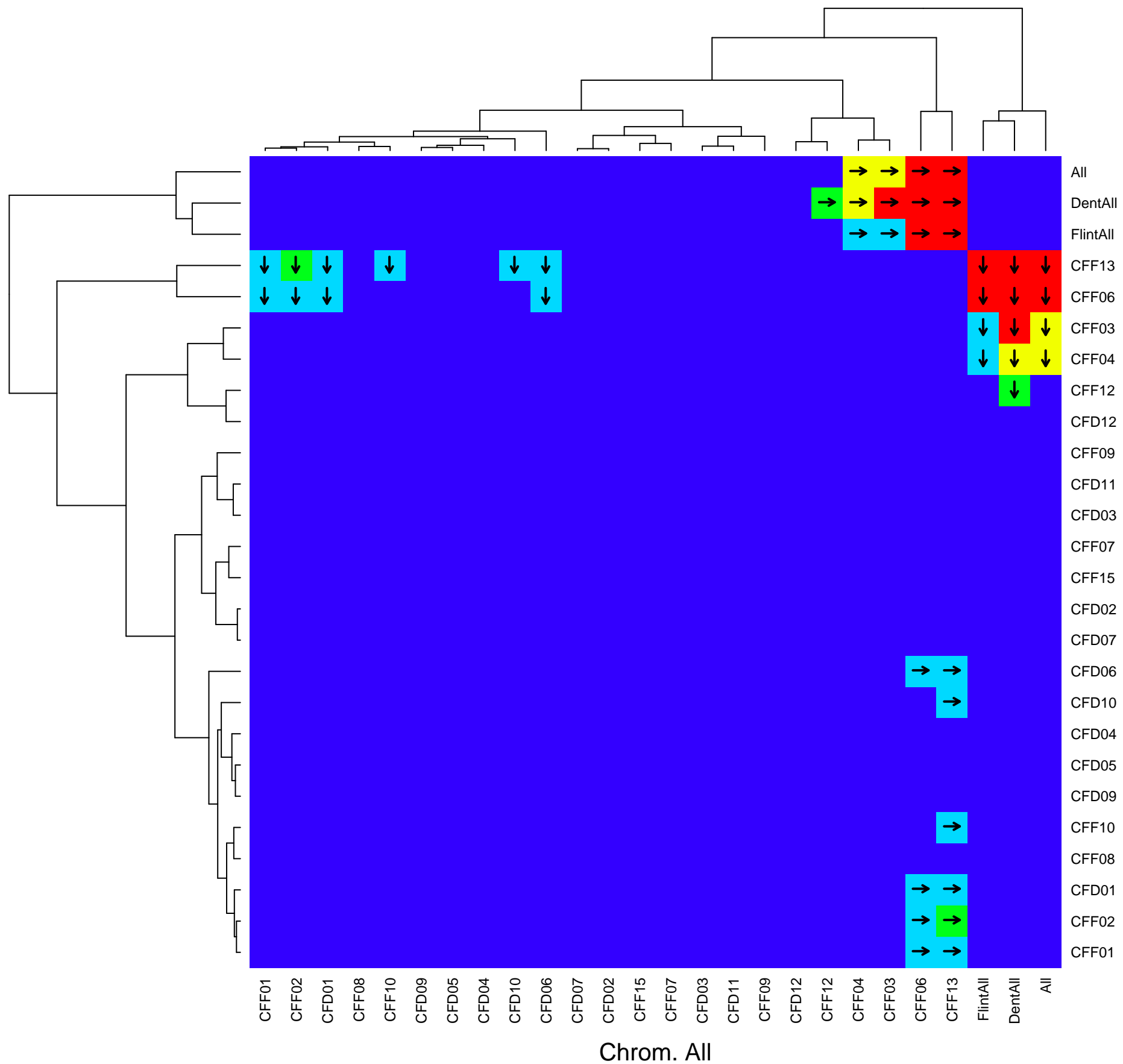

Supplement: Additional file 12: Figure S7 — Statistical comparisons of interference intensity in pathway P1 (nu) between individual populations, for all chromosomes pooled together. 'DentAll', 'FlintAll', and 'All' correspond, respectively, to pooled analyses of all Dent × Dent populations, all Flint × Flint populations, and all 23 populations together. Dark blue, light blue, green, yellow, and red correspond respectively to P ≥ 5.10-2, 10-3 ≤ P < 10-2, 10-4 ≤ P < 10-3, 10-5 ≤ P < 10-4, P < 10-5 where P is the P value of the pairwise comparison test, corrected for multiple testing (Bonferroni). Arrows pointing to the right (respectively to the bottom) indicate that the cross listed in the vertical axis (respectively the horizontal axis) has a higher value of nu than the cross listed in the horizontal axis (respectively the vertical axis). Dendrograms indicate hierarchical clustering of -log10(P value) based on Euclidian distances, and were used to order the populations. [file gb-2013-14-9-r103-S12.pdf]

Proportion  $p$  of crossover formed *via* P2

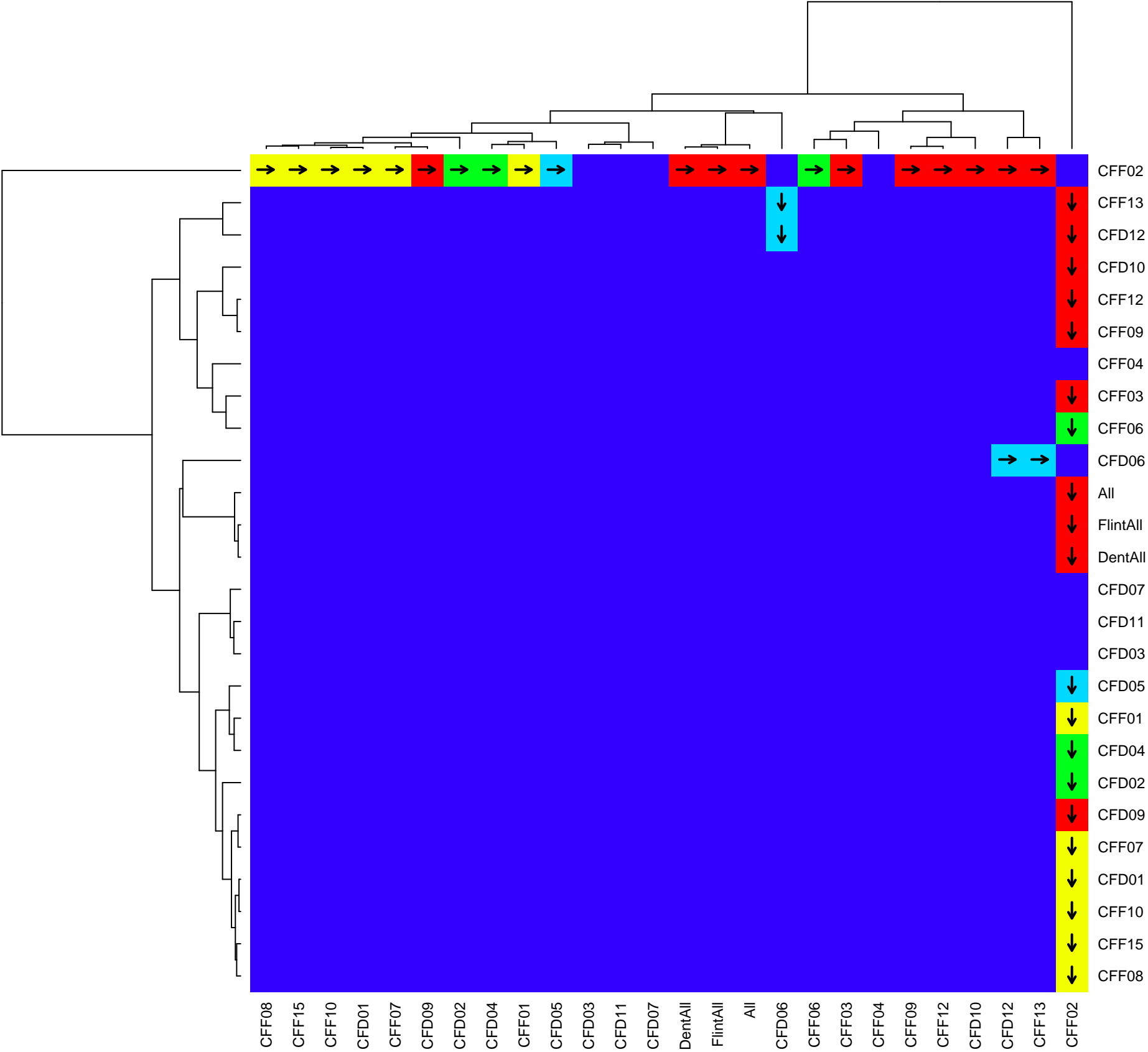

Chrom. All

Supplement: Additional file 13: Figure S8 — Statistical comparisons between individual populations, of the fraction (p) of crossovers formed via the non-interfering pathway P2, for all chromosomes pooled together. 'DentAll', 'FlintAll', and 'All' correspond, respectively, to pooled analyses of all Dent × Dent populations, all Flint × Flint populations, and all 23 populations together. Dark blue, light blue, green, yellow, and red correspond respectively to P ≥ 5.10-2, 10-3 ≤ P < 10-2, 10-4 ≤ P < 10-3, 10-5 ≤ P < 10-4, P < 10-5 where P is the P value of the pairwise comparison test, corrected for multiple testing (Bonferroni). Arrows pointing to the right (respectively to the bottom) indicate that the cross listed in the vertical axis (respectively the horizontal axis) has a higher value of p than the cross listed in the horizontal axis (respectively the vertical axis). Dendrograms indicate hierarchical clustering of -log10(P value) based on Euclidian distances, and were used to order the populations. [file gb-2013-14-9-r103-S13.pdf]

## Chromosome All

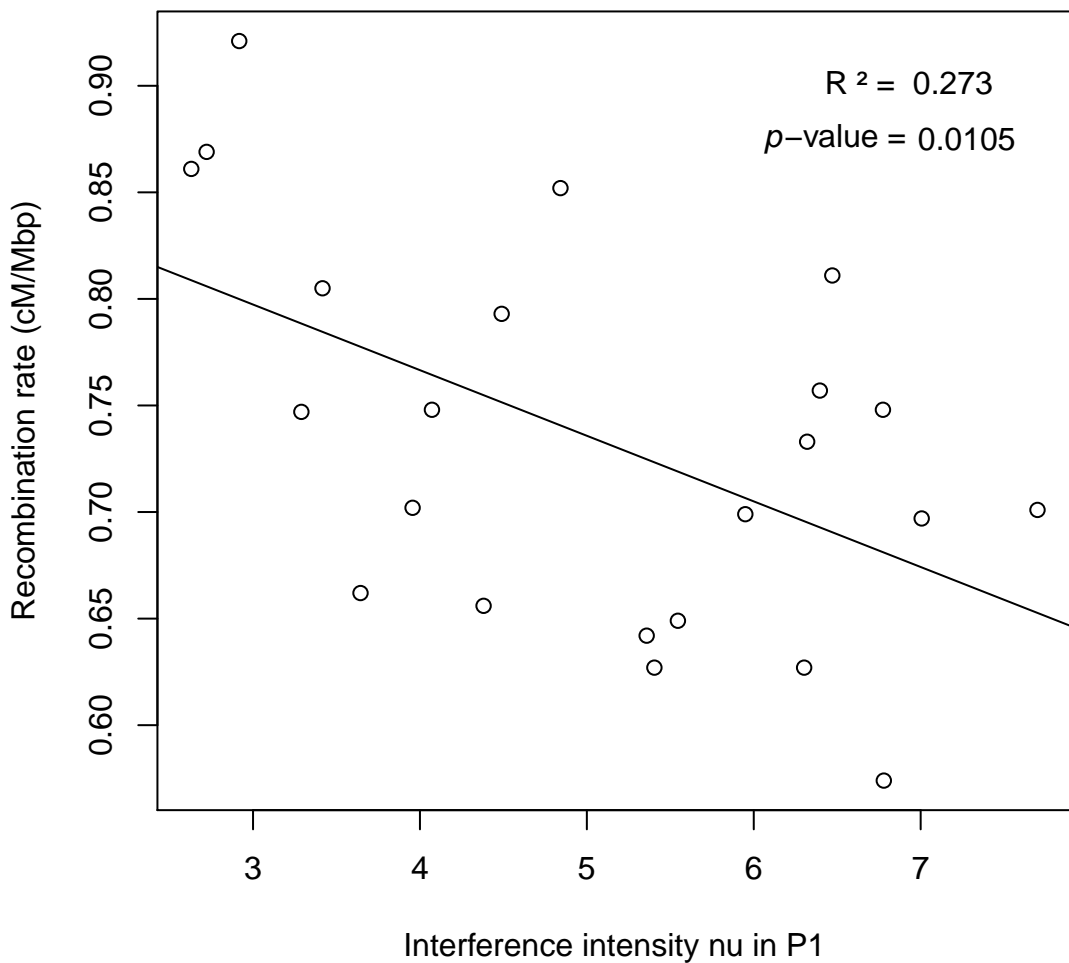

Supplement: Additional file 14: Figure S9 — Correlation between interference intensity in pathway P1 (nu) and genome-wide recombination rate for the 10 chromosomes pooled together over all populations. [file gb-2013-14-9-r103-S14.pdf]

## Half-sib panel Dent

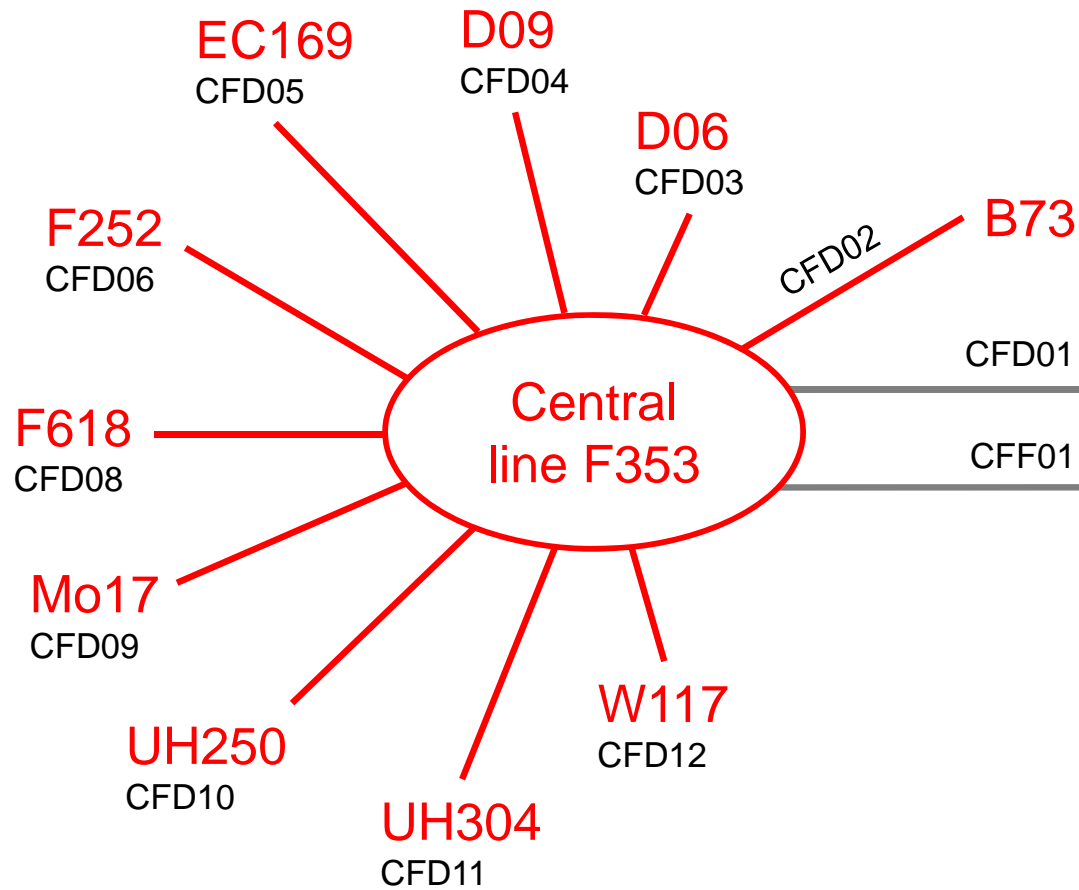

## Half-sib panel Flint

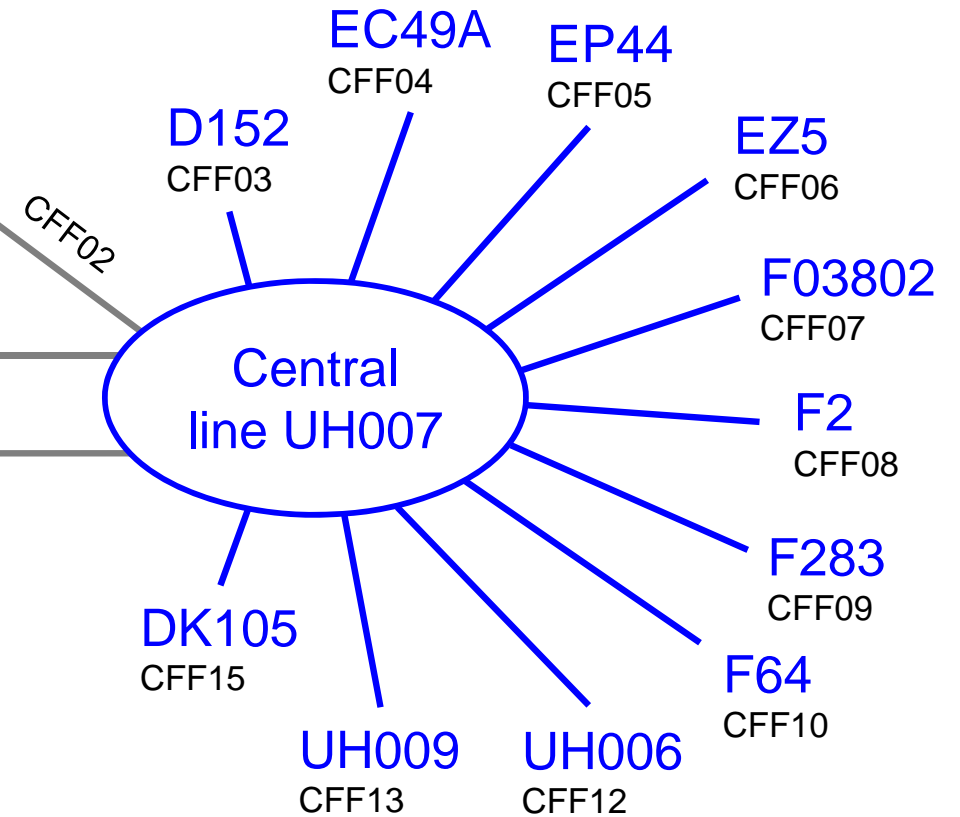

Supplement: Additional file 15: Figure S10 — Crossing scheme of the two Dent and Flint half-sib panels. In each panel, a central line was crossed to diverse founder lines and DH lines were developed from the resulting F1 plants using the in vivo haploid induction method. Dent lines are shown in red, Flint lines in blue. Red lines: Dent × Dent crosses. Blue lines: Flint × Flint crosses. Gray lines: Dent × Flint/Flint × Dent crosses. Two reciprocal crosses (CFD01, CFF01) connect the panels via the central lines F353 and UH007. Both panels are also connected by crossing the central parent to B73 (CFD02, CFF02). The panels consist of 11 CFD and 13 CFF full-sib families, respectively. [file gb-2013-14-9-r103-S15.pdf]
