# Supplementary material for: Intraspecific variation of recombination rate in maize
Source: Genome Biol. 2013 Sep 19;14(9):R103. doi: 10.1186/gb-2013-14-9-r103 (PMC4053771; doi:10.1186/gb-2013-14-9-r103)
Supplement: Additional file 6: Figure S2 — Statistical comparison of chromsome-wide recombination rates between the 23 populations of the experiment. Chromosome 'All' (first page) corresponds to the genome-wide analysis. 'DentAll', 'FlintAll', and 'All' correspond, respectively, to pooled analyses of all Dent × Dent populations, all Flint × Flint populations, and all 23 populations together. Dark blue, light blue, green, yellow, and red correspond respectively to P ≥ 5.10-2, 10-3 ≤ P < 10-2, 10-4 ≤ P < 10-3, 10-5 ≤ P < 10-4, P < 10-5 where P is the P value of the pairwise comparison test, corrected for multiple testing (Bonferroni). Arrows pointing to the right (respectively to the bottom) indicate that the cross listed in the vertical axis (respectively the horizontal axis) has a higher recombination rate than the cross listed in the horizontal axis (respectively the vertical axis). Dendrograms indicate hierarchical clustering of -log10(P value) based on Euclidian distances, and were used to order the populations. [file gb-2013-14-9-r103-S6.pdf]

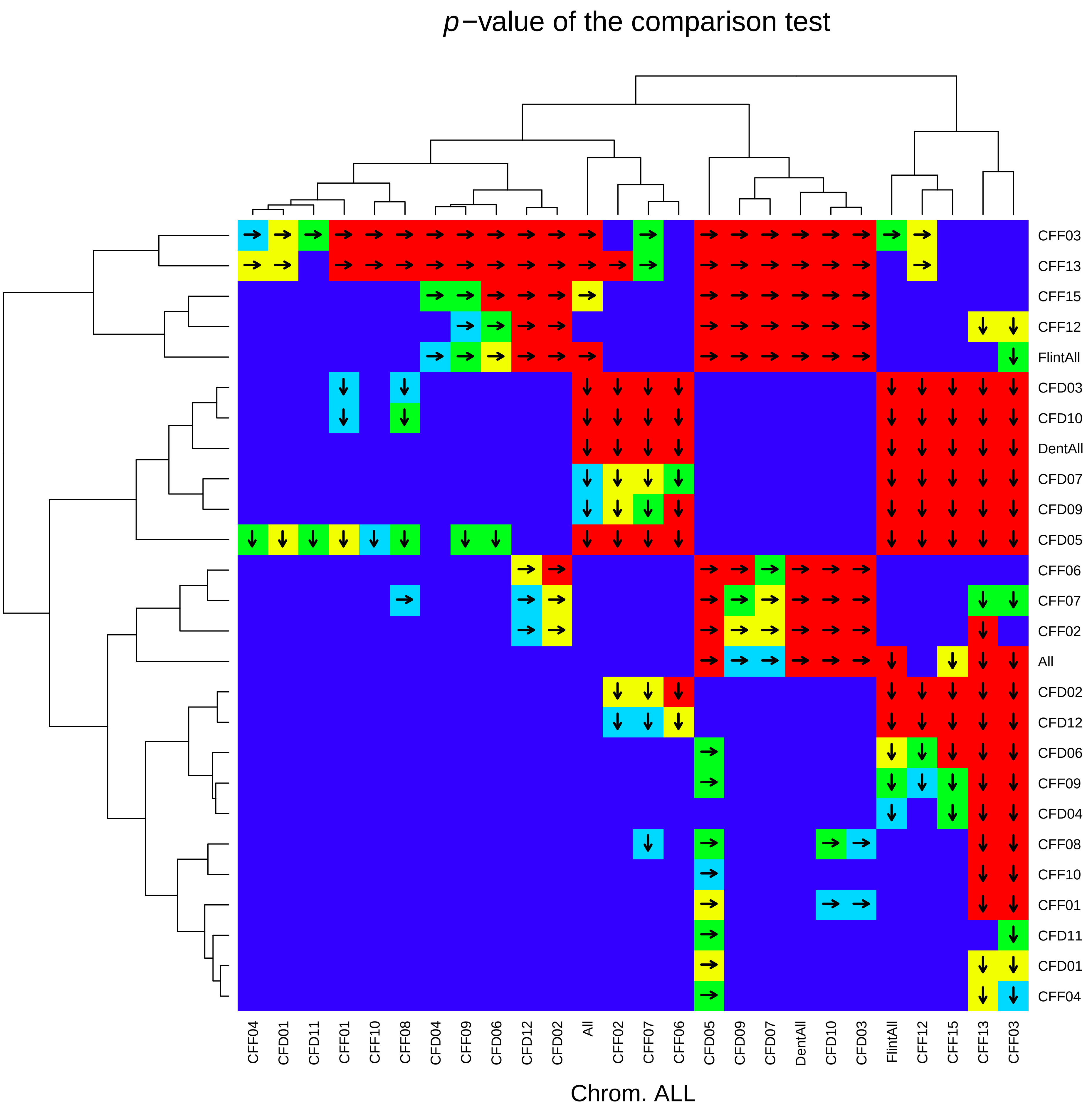

p−value of the comparison test
CFF03
CFF13
CFF15
CFF12
FlintAll
CFD03
CFD10
DentAll
CFD07
CFD09
CFD05
CFF06
CFF07
CFF02
All
CFD02
CFD12
CFD06
CFF09
CFD04
CFF08
CFF10
CFF01
CFD11
CFD01
CFF04
CFF04
CFD01
CFD11
CFF01
CFF10
CFF08
CFD04
CFF09
CFD06
CFD12
CFD02
All
CFF02
CFF07
CFF06
CFD05
CFD09
CFD07
DentAll
CFD10
CFD03
FlintAll
CFF12
CFF15
CFF13
CFF03
Chrom. ALL

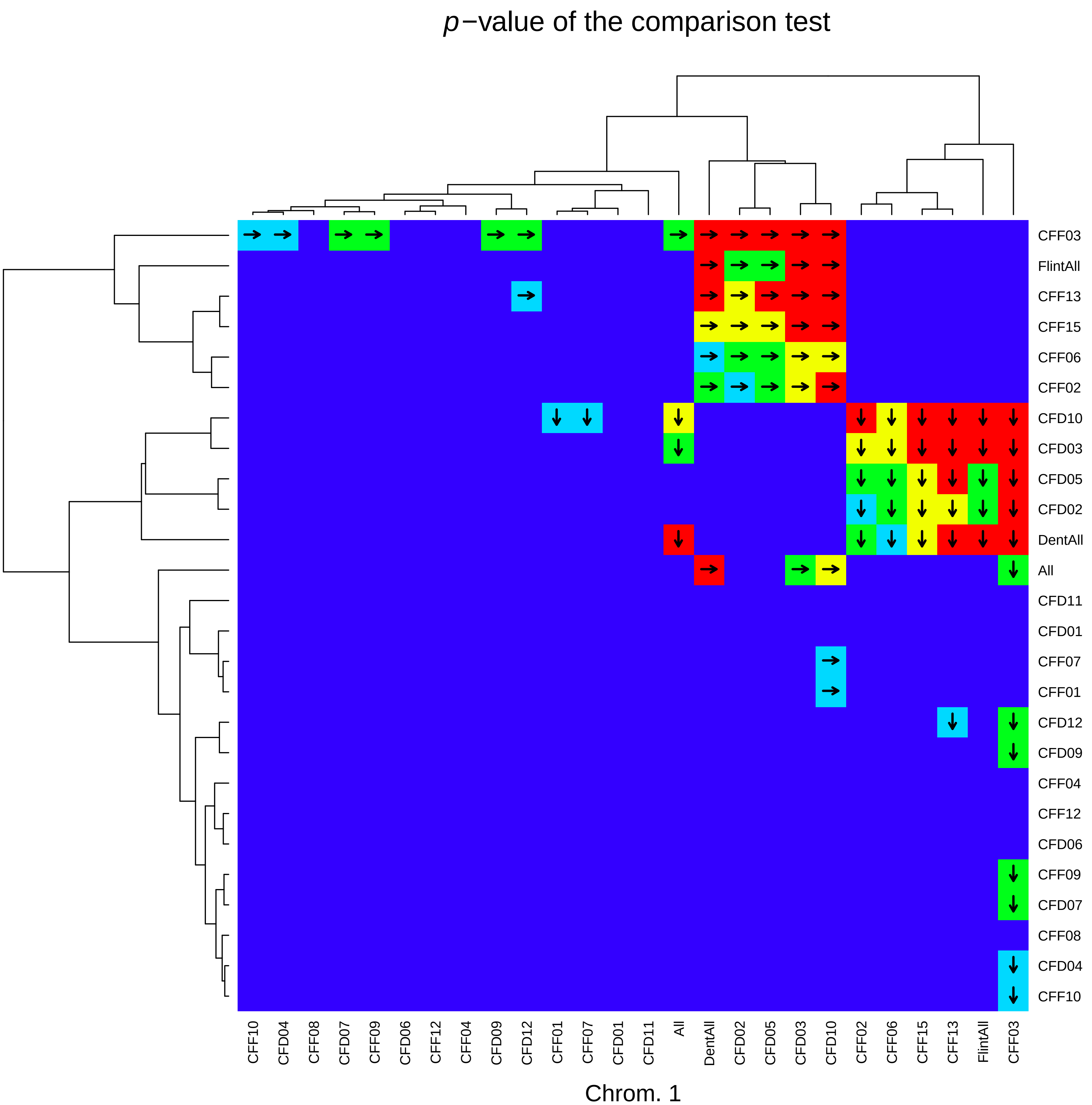

p−value of the comparison test
CFF10
CFD04
CFF08
CFD07
CFF09
CFD06
CFF12
CFF04
CFD09
CFD12
CFF01
CFF07
CFD01
CFD11
All
DentAll
CFD02
CFD05
CFD03
CFD10
CFF02
CFF06
CFF15
CFF13
FlintAll
CFF03
CFF03
FlintAll
CFF13
CFF15
CFF06
CFF02
CFD10
CFD03
CFD05
CFD02
DentAll
All
CFD11
CFD01
CFF07
CFF01
CFD12
CFD09
CFF04
CFF12
CFD06
CFF09
CFD07
CFF08
CFD04
CFF10
Chrom. 1

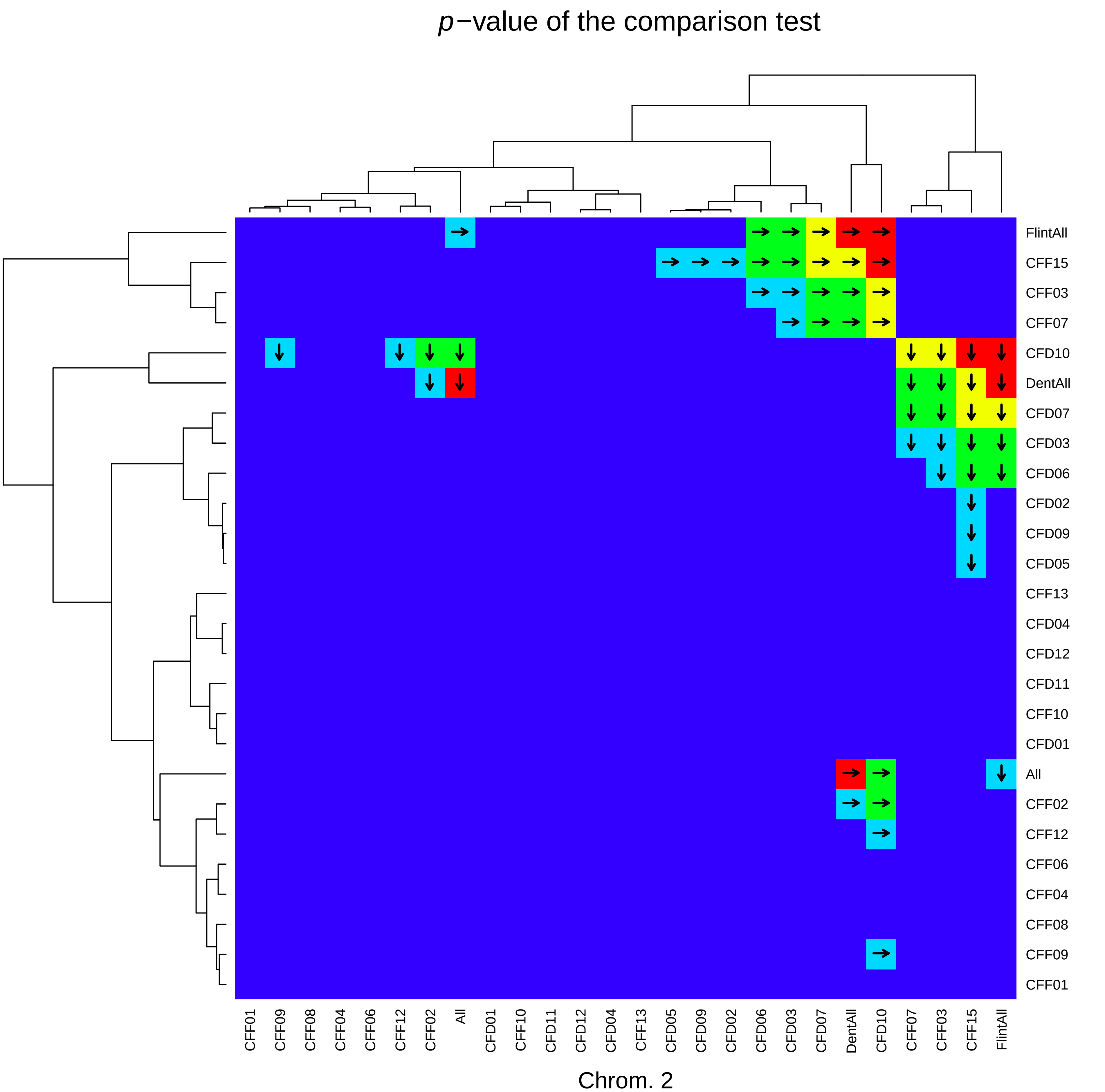
p−value of the comparison test
FlintAll
CFF15
CFF03
CFF07
CFD10
DentAll
CFD07
CFD03
CFD06
CFD02
CFD09
CFD05
CFF13
CFD04
CFD12
CFD11
CFF10
CFD01
All
CFF02
CFF12
CFF06
CFF04
CFF08
CFF09
CFF01
CFF01
CFF09
CFF08
CFF04
CFF06
CFF12
CFF02
All
CFD01
CFF10
CFD11
CFD12
CFD04
CFF13
CFD05
CFD09
CFD02
CFD06
CFD03
CFD07
DentAll
CFD10
CFF07
CFF03
CFF15
FlintAll
Chrom. 2

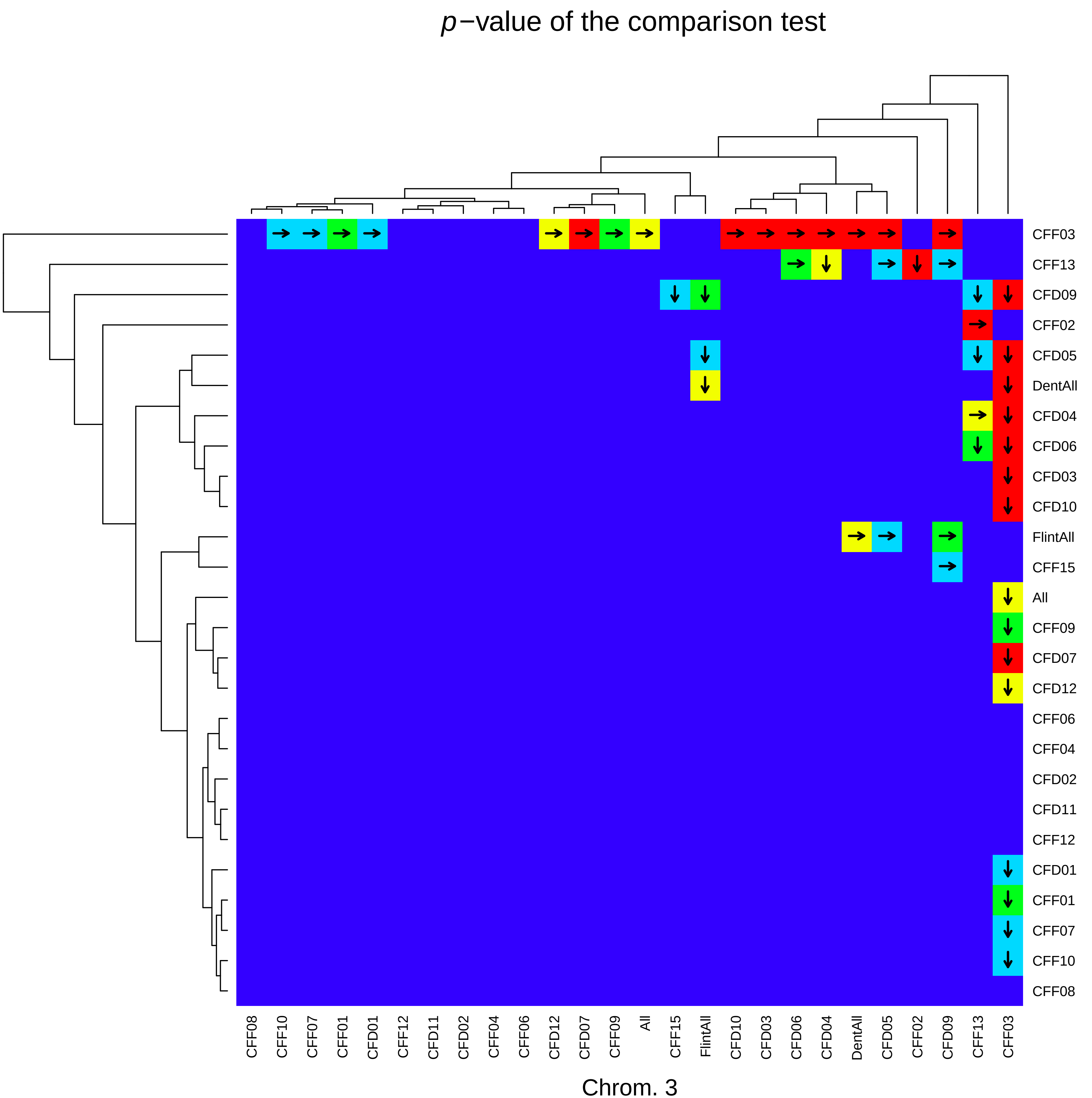

p−value of the comparison test
CFF08 CFF10 CFF07 CFF01 CFD01 CFF12 CFD11 CFD02 CFF04 CFF06 CFD12 CFD07 CFF09 All CFF15 FlintAll CFD10 CFD03 CFD06 CFD04 DentAll CFD05 CFF02 CFD09 CFF13 CFF03
CFF03 CFF13 CFD09 CFF02 CFD05 DentAll CFD04 CFD06 CFD03 CFD10 FlintAll CFF15 All CFF09 CFD07 CFD12 CFF06 CFF04 CFD02 CFD11 CFF12 CFD01 CFF01 CFF07 CFF10 CFF08
Chrom. 3

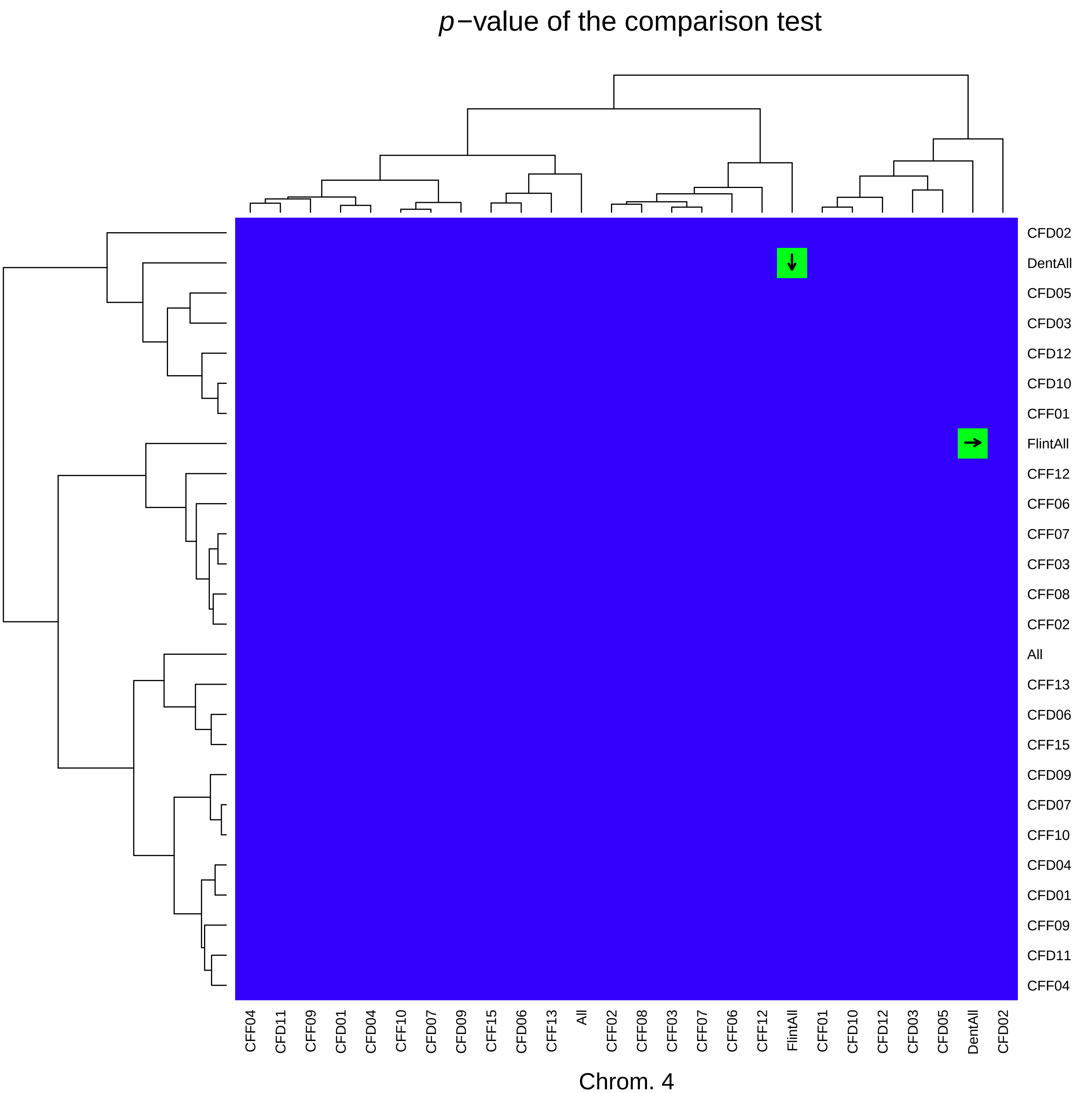
p−value of the comparison test
CFD02
DentAll
CFD05
CFD03
CFD12
CFD10
CFF01
FlintAll
CFF12
CFF06
CFF07
CFF03
CFF08
CFF02
All
CFF13
CFD06
CFF15
CFD09
CFD07
CFF10
CFD04
CFD01
CFF09
CFD11
CFF04
CFF04
CFD11
CFF09
CFD01
CFD04
CFF10
CFD07
CFD09
CFF15
CFD06
CFF13
All
CFF02
CFF08
CFF03
CFF07
CFF06
CFF12
FlintAll
CFF01
CFD10
CFD12
CFD03
CFD05
DentAll
CFD02
Chrom. 4

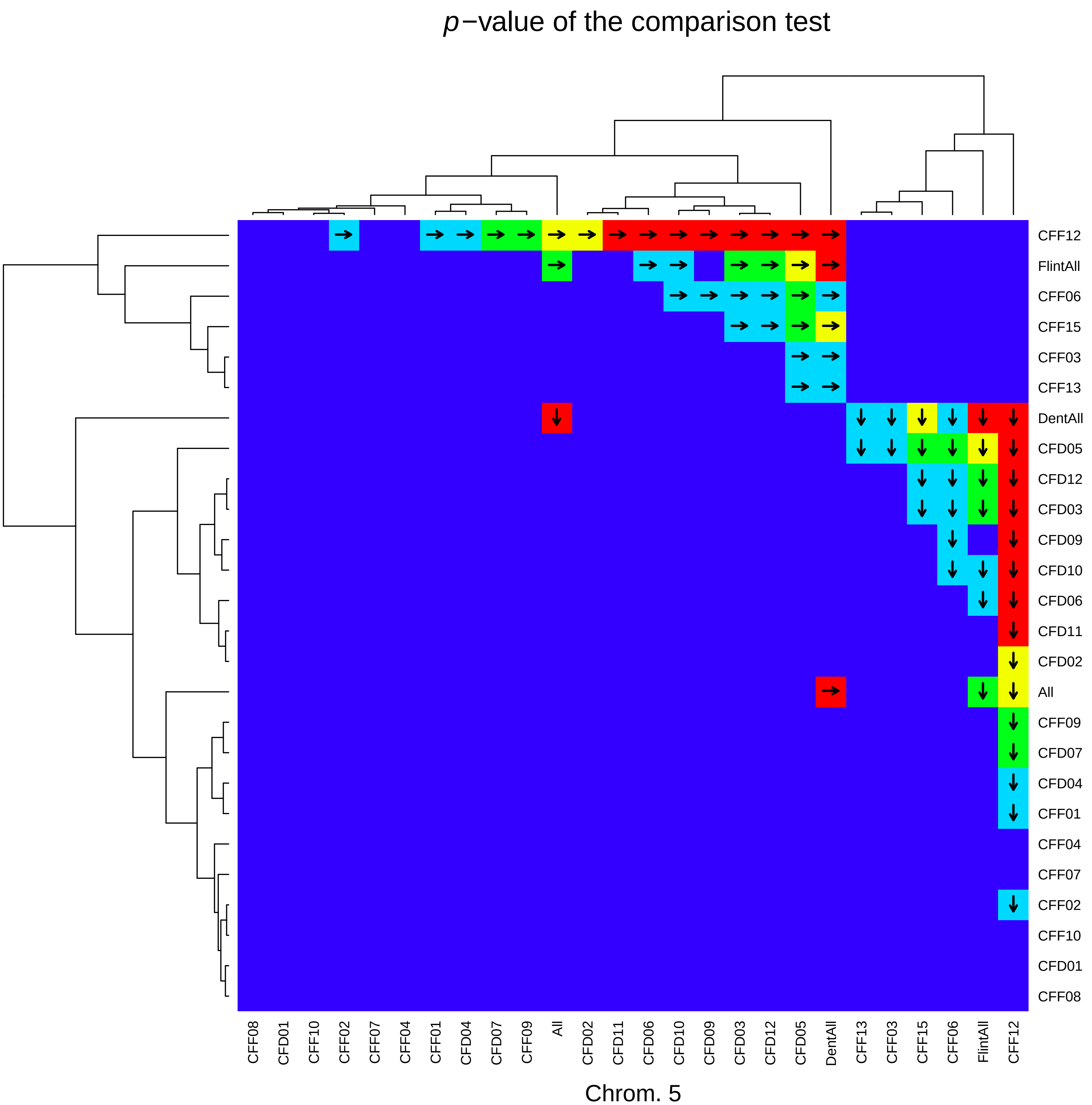
p−value of the comparison test
CFF12
FlintAll
CFF06
CFF15
CFF03
CFF13
DentAll
CFD05
CFD12
CFD03
CFD09
CFD10
CFD06
CFD11
CFD02
All
CFF09
CFD07
CFD04
CFF01
CFF04
CFF07
CFF02
CFF10
CFD01
CFF08
CFF08
CFD01
CFF10
CFF02
CFF07
CFF04
CFF01
CFD04
CFD07
CFF09
All
CFD02
CFD11
CFD06
CFD10
CFD09
CFD03
CFD12
CFD05
DentAll
CFF13
CFF03
CFF15
CFF06
FlintAll
CFF12
Chrom. 5

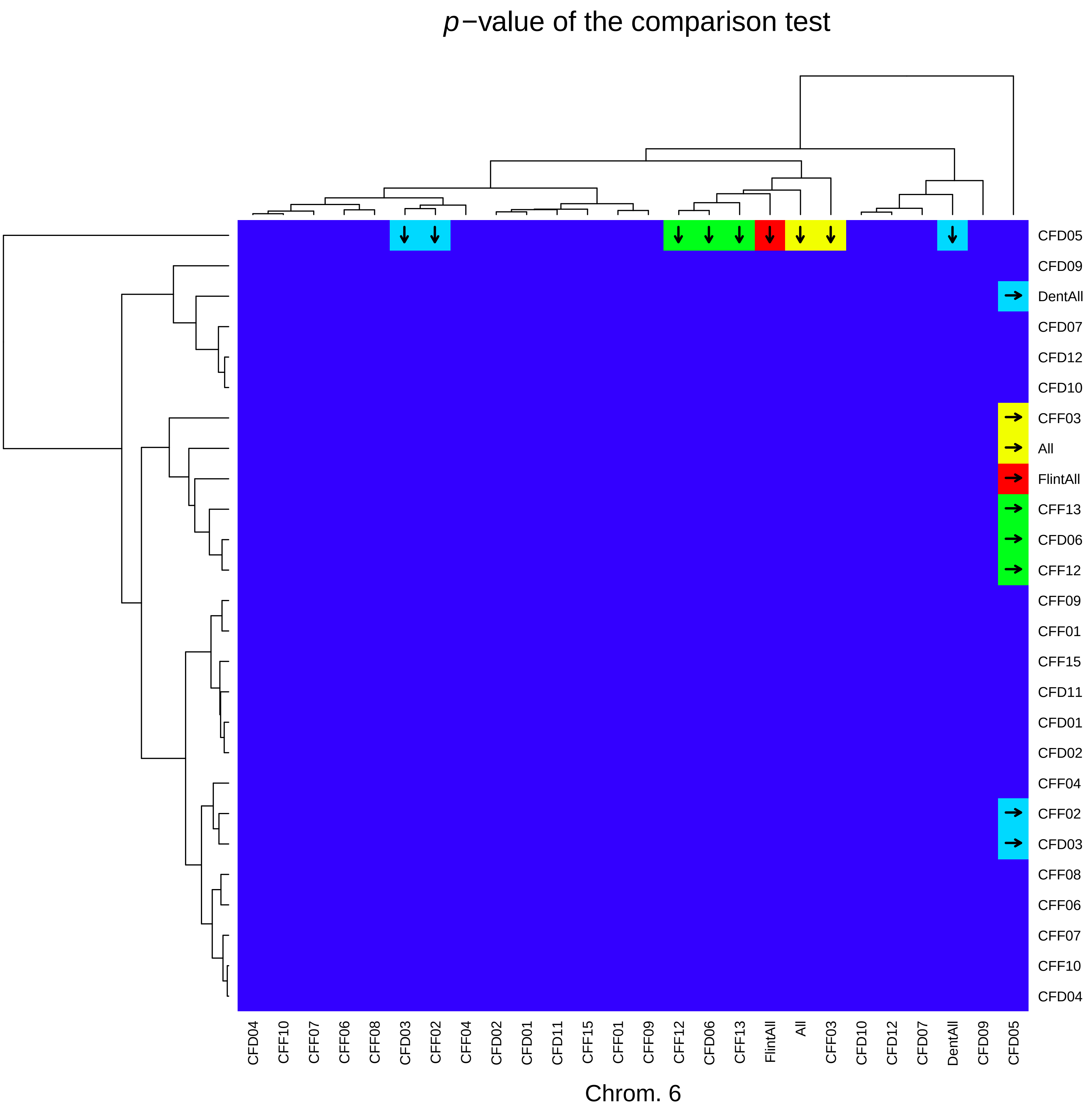
p−value of the comparison test
CFD05
CFD09
DentAll
CFD07
CFD12
CFD10
CFF03
All
FlintAll
CFF13
CFD06
CFF12
CFF09
CFF01
CFF15
CFD11
CFD01
CFD02
CFF04
CFF02
CFD03
CFF08
CFF06
CFF07
CFF10
CFD04
CFD04
CFF10
CFF07
CFF06
CFF08
CFD03
CFF02
CFF04
CFD02
CFD01
CFD11
CFF15
CFF01
CFF09
CFF12
CFD06
CFF13
FlintAll
All
CFF03
CFD10
CFD12
CFD07
DentAll
CFD09
CFD05
Chrom. 6

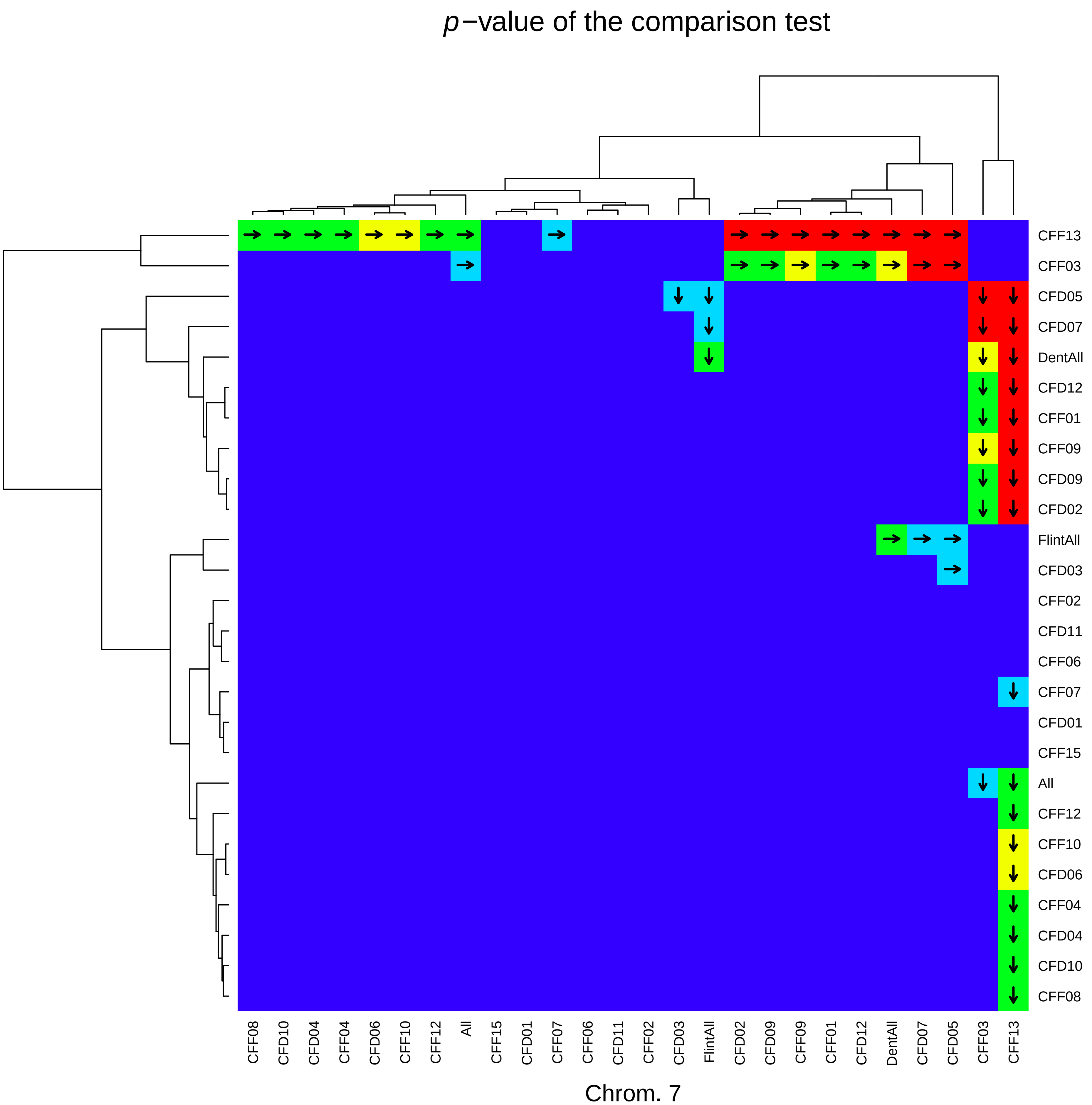
p−value of the comparison test
CFF08
CFD10
CFD04
CFF04
CFD06
CFF10
CFF12
All
CFF15
CFD01
CFF07
CFF06
CFD11
CFF02
CFD03
FlintAll
CFD02
CFD09
CFF09
CFF01
CFD12
DentAll
CFD07
CFD05
CFF03
CFF13
CFF13
CFF03
CFD05
CFD07
DentAll
CFD12
CFF01
CFF09
CFD09
CFD02
FlintAll
CFD03
CFF02
CFD11
CFF06
CFF07
CFD01
CFF15
All
CFF12
CFF10
CFD06
CFF04
CFD04
CFD10
CFF08
Chrom. 7

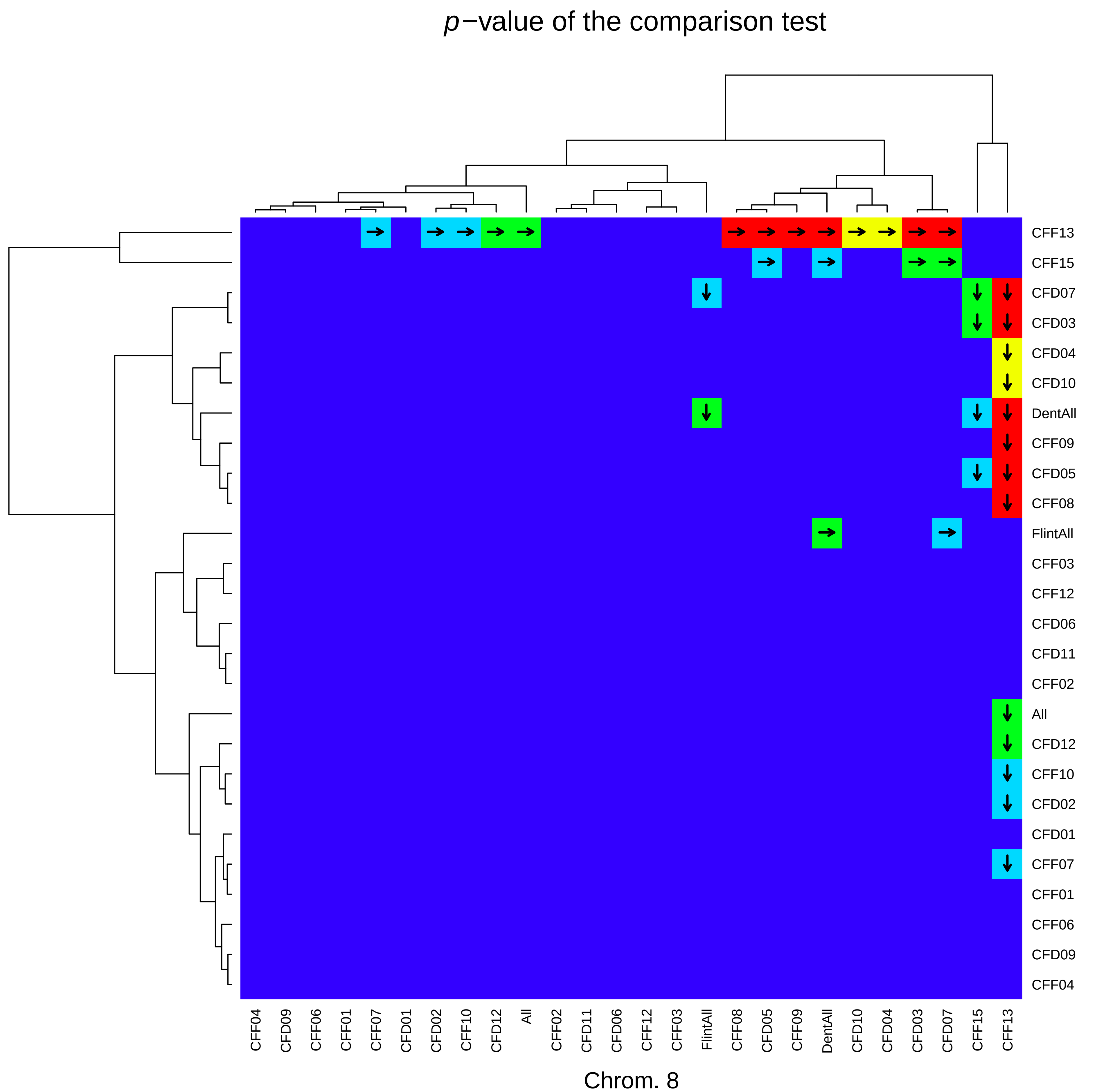
p−value of the comparison test
CFF04
CFD09
CFF06
CFF01
CFF07
CFD01
CFD02
CFF10
CFD12
All
CFF02
CFD11
CFD06
CFF12
CFF03
FlintAll
CFF08
CFD05
CFF09
DentAll
CFD10
CFD04
CFD03
CFD07
CFF15
CFF13
CFF13
CFF15
CFD07
CFD03
CFD04
CFD10
DentAll
CFF09
CFD05
CFF08
FlintAll
CFF03
CFF12
CFD06
CFD11
CFF02
All
CFD12
CFF10
CFD02
CFD01
CFF07
CFF01
CFF06
CFD09
CFF04
Chrom. 8

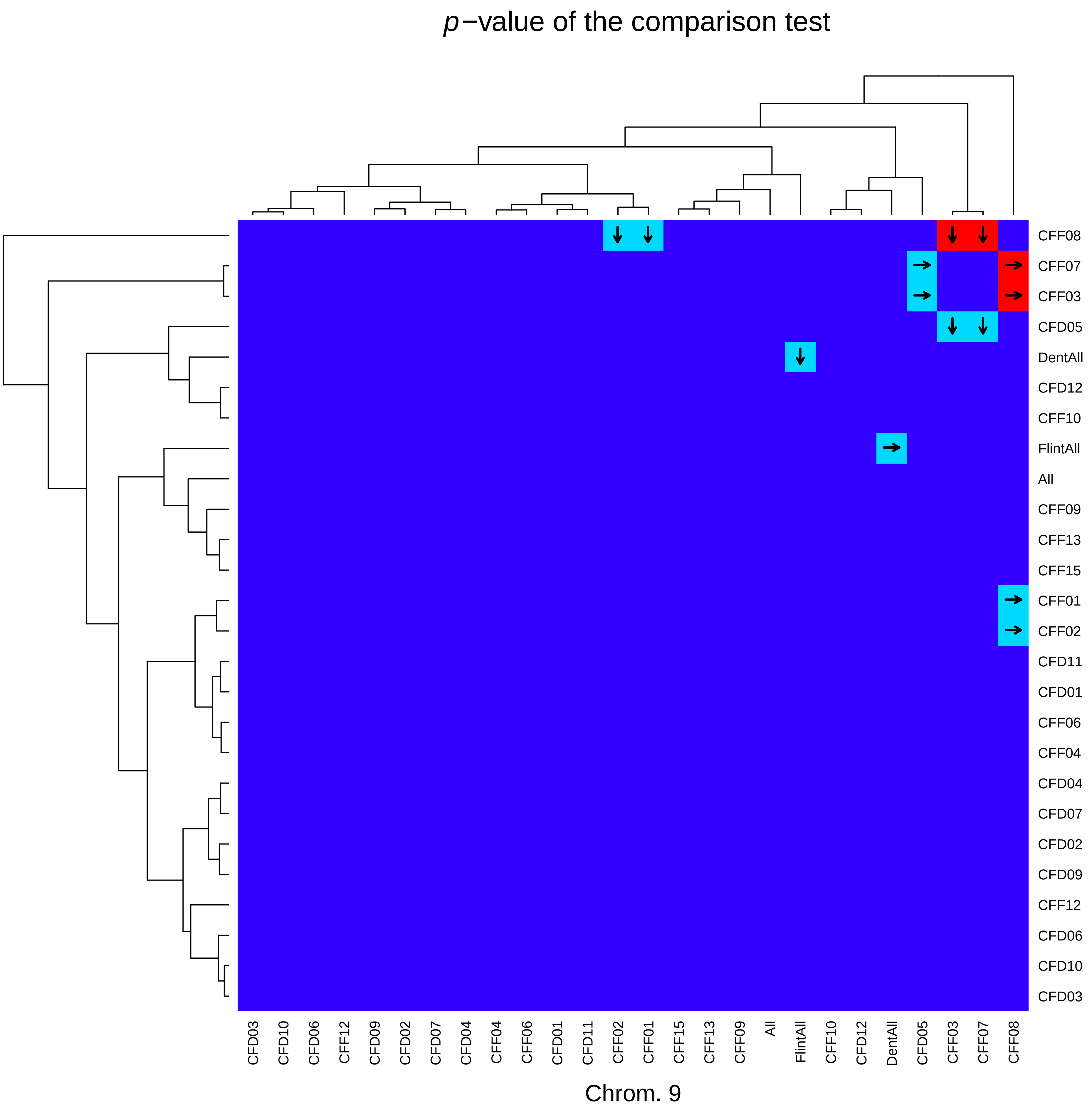

p−value of the comparison test
CFF08
CFF07
CFF03
CFD05
DentAll
CFD12
CFF10
FlintAll
All
CFF09
CFF13
CFF15
CFF01
CFF02
CFD11
CFD01
CFF06
CFF04
CFD04
CFD07
CFD02
CFD09
CFF12
CFD06
CFD10
CFD03
Chrom. 9

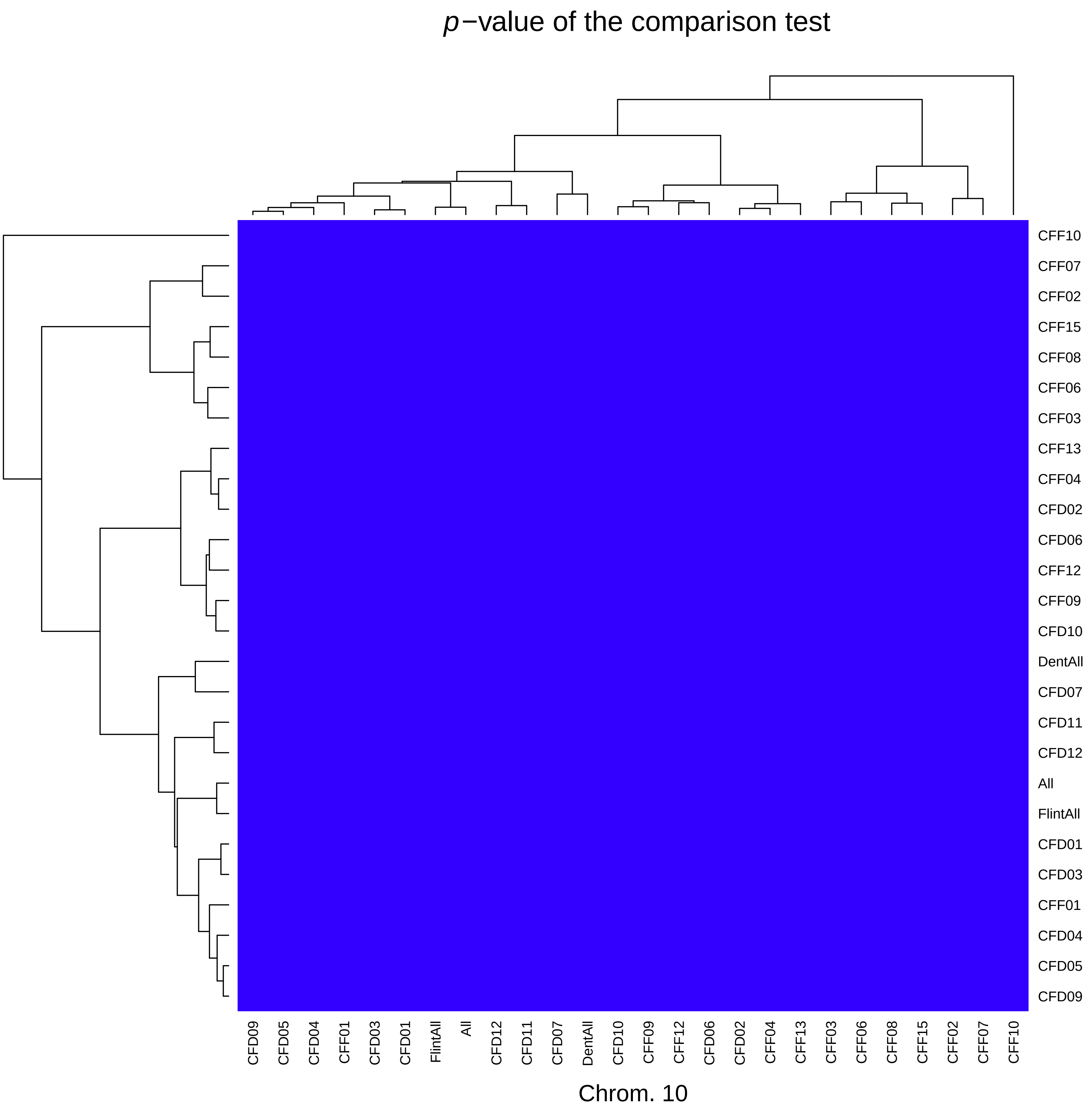

p−value of the comparison test
CFF10
CFF07
CFF02
CFF15
CFF08
CFF06
CFF03
CFF13
CFF04
CFD02
CFD06
CFF12
CFF09
CFD10
DentAll
CFD07
CFD11
CFD12
All
FlintAll
CFD01
CFD03
CFF01
CFD04
CFD05
CFD09
CFD09
CFD05
CFD04
CFF01
CFD03
CFD01
FlintAll
All
CFD12
CFD11
CFD07
DentAll
CFD10
CFF09
CFF12
CFD06
CFD02
CFF04
CFF13
CFF03
CFF06
CFF08
CFF15
CFF02
CFF07
CFF10
Chrom. 10
