# Supplementary material for: Intraspecific variation of recombination rate in maize
Source: Genome Biol. 2013 Sep 19;14(9):R103. doi: 10.1186/gb-2013-14-9-r103 (PMC4053771; doi:10.1186/gb-2013-14-9-r103)
Supplement: Additional file 8: Text S2 — Detailed methods. [file gb-2013-14-9-r103-S8.pdf]

Additional file 8 (Text S2): Detailed Methods

Analysis of parental genetic diversity

Principal coordinate analysis: To determine how well the central and founder lines in our study represent the European Flint and Dent maize germplasm pool, we reanalysed the parental lines within the diversity panels described earlier [1]. These two diversity panels for European Flint and Dent were composed each of around 300 lines each, including all parents of the two half-sib panels of the present study, except UH009 (Flint). We added data for this line and performed a principal coordinate analysis (PCoA) based on the distance matrices obtained from PANZEA SNPs as described earlier [1]. For the Dent panel, 29,013 SNPs were used and for the Flint panel 26,660. The main germplasm groups in each panel were identified by structure analysis using the Admixture software (version 1.22; [2]). In both Dent and Flint, four main groups were identified and lines were colour-coded accordingly in Additional file 2 (Figure S11).

Cluster analysis: To describe the genetic diversity among the 23 central and founder lines of the two half-sib panels, 23,186 SNP markers were used (MAF > 0.05, max. 10% missing data). Markers with the prefix SYNGENTA or SYN were omitted to avoid bias introduced by this set of markers in diversity studies [3]. Missing data were imputed using the Synbreed R package [4] using the *codeGeno* function and the imputing type *random*. Genetic distances between parents were calculated based on 23,186 SNPs with the R function *hclust* of the R stats package. We calculated Rogers' distances and used the *average* method for clustering. Calculations were performed genome-wide as well as chromosome-wise based on chromosome assignment in the maize B73 AGPv2 physical map (Additional file 2, Figure S12).

Genome-scan for genetic similarity between parents of each cross: For a visual display of similarity between each of the central lines F353 and UH007 and their corresponding Dent and Flint crossing partners we performed a genome-scan for SNP polymorphisms along all chromosomes using physical map coordinates of 32,501 SNPs, excluding SYNGENTA or SYN SNPs. In sliding windows with a window size of 10 Mbp and a step size of 2 Mbp, pairwise distances were calculated as per cent identical SNP alleles within each window. The results were displayed as heat maps in a pairwise comparison between the central lines F353 and UH007 and the corresponding founder lines in Dent and Flint, respectively (Additional file 2, Figure S13).

Construction of bare and masked Marey maps

Given the genetic map of an individual population for a chromosome, the positions of the markers on the B73 AGPv2 assembly were obtained. From these physical and genetic positions, we constructed a first Marey map containing all syntenic markers. Due to small inaccuracies in the genetic positions or potential structural rearrangements, this Marey map showed regions where the genetic coordinate did not increase monotonically with physical position. Since it is necessary to obtain a smooth and monotonic Marey map, we iteratively performed interpolations and filtered outlying markers. In detail, a first smoothed map was generated using a cubic spline interpolation from which we identified outlier markers (residuals greater than 10 cM) that were removed. We then scanned the remaining markers from left to right, removing those which produced non-monotonicities beyond a tolerance of 2cM when comparing adjacent markers. In case there were large gaps, we added points to interpolate the Marey map linearly. We then smoothed the map encoded by these markers and points via a cubic spline again. Then we took a total of 1000 positions evenly spaced on the physical B73 chromosome. Scanning these 1000 positions from left to right, the value of the map function was taken to be that given by the previous spline if monotonicity was maintained; otherwise it was set to the value at the position immediately to the left. A last

spline of this discretized monotonic function then defined what we call the "*bare Marey map*", specifying a genetic position for any physical position on B73.

Superimposing the (continuous and monotonic) bare Marey map with the scatter plot of physical and genetic positions of the markers in the original genetic map, one sometimes finds regions of discrepancies or regions with no data from the genetic map. The first kind of region could arise for instance from structural differences with B73 while the second kind could be expected from lack of polymorphism between the parents because pieces of the chromosome are identical by descent (IBD). Regions of the Marey maps having such discrepancies require interpretation outside of a B73 framework while those having no data were not reliable since they were based on simple interpolation to fill gaps. Thus whenever Marey maps were used for further analyses such as for comparing recombination landscapes, we replaced them by "masked Marey maps" as follows. First we defined the regions that must be masked because the Marey map in this region was either not interpretable or not determined reliably. The larger regions with no marker data were easily delimited (e.g. CFF07 chromosome 1 in Additional file 7), while regions suggesting possible structural problems had to be detected manually. Although in general this second type of masking involved a subjective appreciation, for the data at hand the regions were quite unambiguous (e.g. CFF01 chromosome 3 in Additional file 7): they either had a doubling of the map so that the markers fell on two curves separated by an offset in genetic position, or they contained markers clearly suggesting an inversion compared to B73. In all cases, we delimited the misbehaved regions by masking them. The (continuous and monotonic) map in the remaining regions then defined the "masked Marey maps" (see Additional file 9).

Imputed Marey maps for comparison tests

To compare the genetic lengths and the recombination landscapes between the different crosses, only masked Marey maps should be used because the shape of the inferred recombination landscape within masked regions is unreliable. Comparing masked maps was

a challenge because the masked regions varied from one map to another. We overcame this difficulty by replacing each masked Marey map by an “imputed” Marey map. The idea was that for each region of a map where the recombination landscape was missing, we replaced it by an imputed landscape. Call *M* the masked Marey map to be imputed, and focus on one of its masked regions (for typical sizes of these masked regions, see Additional file 9). We divided that region into many small intervals. For each interval, we calculated its average recombination rate from all other masked Marey maps for which that same interval was *not* masked. This average was weighted by the population sizes used to produce those maps. The *shape* of the imputed landscape was thus inferred from the other maps while the genetic length of this region was unchanged. The exception to this rule arose when the masked region was at the end of the chromosome: in that case, since the cross provided no estimate of the region's genetic length, we took the region's genetic length to be the one inferred from the other maps.

Comparing genetic map lengths

For any population, mapping data lead to an estimate of the genetic length for a given chromosome. We wanted to examine pairwise differences between populations as well as differences between pools of populations and test them for their level of statistical significance. Consider first the comparison of two populations. The associated bare Marey maps provided estimates for the respective genetic lengths (L1 and L2). Since these estimates were obtained by averaging over a significant number of independent meioses (given by the number of plants in the population, a number much greater than 10), their distribution can be approximated by a normal distribution. Each associated variance can be obtained by considering that the number of crossovers in a meiosis follows a Poisson distribution with mean and variance given by the estimated genetic length in Morgan. The statistical significance of the difference L1 - L2 was then obtained from the Welch test with a significance threshold of 5% and Bonferroni correction for multiple testing. However, if either

of the ends of the chromosome was masked in the masked Marey map, it was necessary to perform the comparison tests starting with the imputed rather than the bare Marey maps. As explained in the previous section, these imputed maps were computed for each comparison test and then each provided an estimate of the recombination landscape for its associated cross. The regions of any imputed map could be of three imputation “types”: (1) the recombination landscape was determined directly, without imputation; (2) the recombination landscape was imputed but the genetic length of the region was directly determined; (3) both the recombination landscape and the genetic length of the region were imputed (in the extremities of chromosomes). Given two imputed Marey maps, we decomposed the chromosome into maximal subregions where both of the maps had constant (though perhaps different) imputation types (1, 2, or 3). Subregions were delimited by boundaries of the masked regions in at least one of the underlying masked Marey maps. For sub-regions where both maps could be used to estimate the corresponding genetic length (imputation type 1 and type 2), we computed a value of genetic length and an associated variance as described above. The other subregions were not used so when comparing two maps, some chromosomes had less than half of their length contributing to the comparison. Summing genetic length and variance over all usable subregions, we got two estimates of total genetic length along with their variances. The Welch test was then applied to the difference of these two estimates. For this work, we used the implementation in the R software called welch.test(). In effect, we tested for a difference between populations of the length of the regions where each population was informative, not of the entire chromosome's genetic length.

The comparison of genetic lengths between two populations was extended to the two pools of Dent x Dent and Flint x Flint populations. Each individual population had a masked Marey map that had to be imputed using all the other populations as previously explained. Once these imputed Marey maps were generated, we decomposed the chromosome into maximal subregions where each of the imputed Marey maps was of constant imputation type. (This is

a direct generalization of what was done above when comparing just two imputed Marey maps). For each informative subregion (that is a subregion where at least one population in *each group* had an imputed map of type 1 or 2), we computed the estimated genetic lengths and associated variances for the two groups. The sum over all these subregions then provided us with a “total” genetic length and variance for each group. In practice, for the pooling of Dent vs Flint, all subregions were informative so this total genetic length was an estimate of the chromosome's genetic length in each group. The Welch test was then applied to these lengths using their respective variances.

Comparing recombination landscapes

Just as the genetic lengths of two chromosomes or genomes may differ, their respective recombination landscapes can have different features (different *shapes* of the Marey maps) and it was of interest to test whether these differences were statistically significant. To do so, we normalized the genetic lengths of the two maps or pools of maps to be compared by rescaling both of them to the mean of the two values. Then, to compare the shape of both normalized Marey maps, our approach was based on binning the landscapes, representing each as a histogram and then applying a chi-squared test (see Additional file 11). The comparison involved two landscapes or two groups of landscapes and started by defining the bins: we used 10 bins that each corresponded to 1/10th of the chromosome's genetic length (averaged over all the landscapes) so that the statistical power was well balanced amongst the bins. For each bin, we applied the methodology previously used for the whole chromosome, providing an estimate of the bin’s genetic length and associated variance for the two landscapes or two groups of landscapes. We then tested the hypothesis that the two histograms were obtained from the same underlying landscape; the *p*-value for this test was obtained from the chi-squared distribution having as many degrees of freedom as there were bins with a Bonferroni-corrected significance threshold of 5%.

References

1. Rincent R, Laloë D, Nicolas S, Altmann T, Brunel D, Revilla P, Rodriguez VM, Moreno-Gonzales J, Melchinger AE, Bauer E, Schön C-C, Meyer N, Giauffret C, Bauland C, Jamin P, Laborde J, Monod H, Flament P, Charcosset A, Moreau L: **Maximizing the reliability of genomic selection by optimizing the calibration set of reference individuals: comparison of methods in two diverse groups of maize inbreds (*Zea mays* L.).** *Genetics* 2012, **192:**715-728.
2. Alexander DH, Novembre J, Lange K: **Fast model-based estimation of ancestry in unrelated individuals.** *Genome Research* 2009, **19:**1655-1664.
3. Ganal MW, Durstewitz G, Polley A, Berard A, Buckler ES, Charcosset A, Clarke JD, Graner EM, Hansen M, Joets J, Le Paslier MC, McMullen MD, Montalent P, Rose M, Schön CC, Sun Q, Walter H, Martin OC, Falque M: **A large maize (*Zea mays* L.) SNP genotyping array: development and germplasm genotyping, and genetic mapping to compare with the B73 reference genome.** *PLoS ONE* 2011, **6:**e28334.
4. Wimmer V, Albrecht T, Auinger H-J, Schön C-C: **synbreed: a framework for the analysis of genomic prediction data using R.** *Bioinformatics* 2012, **28:**2086-2087.
