# Supplementary material for: Intraspecific variation of recombination rate in maize
Source: Genome Biol. 2013 Sep 19;14(9):R103. doi: 10.1186/gb-2013-14-9-r103 (PMC4053771; doi:10.1186/gb-2013-14-9-r103)
Supplement: Additional file 9: Figure S4 — Recombination rates along maize chromosomes 1 to 10. The x-axis indicates physical position (Mbp) along chromosomes. The y-axis indicates genetic position (cM; top panel), recombination rate (cM/Mbp; middle panel), and pairwise parental similarity (frequency of identical SNP alleles in 10 Mbp sliding windows with a step size of 2 Mbp; bottom panel) for 6 of the 23 populations, after smoothing and imputation (see Materials and methods). Blue lines: Flint × Flint crosses. Red lines: Dent × Dent crosses. In both groups, the solid, dashed and dotted lines correspond to the population with the highest, median or lowest genome-wide recombination rate within its group, respectively. Gray parts of the lines correspond to regions where the information was missing or not reliable (IBD segments, non-colinearity with B73), and thus imputed from the other maps (see Materials and methods). Heat-maps below the curves of recombination rates indicate gene density (low for cold colors and high for hot colors). Gray horizontal line below the heat-map: sketch of the chromosome organization (from [30]) showing centromeres (cen), knobs, and nucleolar organizer region (NOR). Color filling of chromosome features is solid when the estimated boundaries of the region are known, and hatched when the box indicates only the extremities of the bin containing the region. [file gb-2013-14-9-r103-S9.pdf]

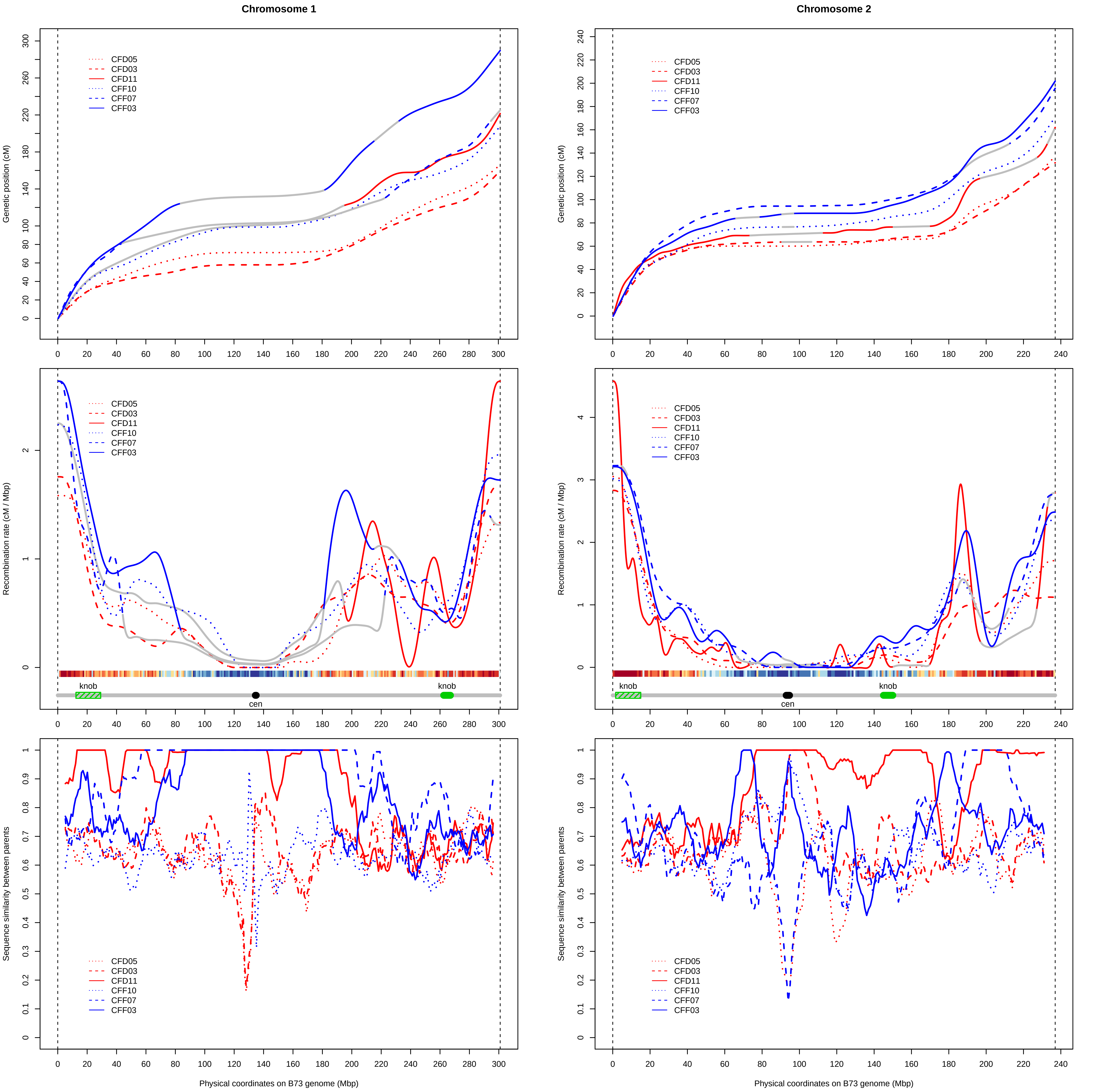

Chromosome 1
Chromosome 2
Genetic position (cM)
Recombination rate (cM / Mbp)
Sequence similarity between parents
CFD05
CFD03
CFD11
CFF10
CFF07
CFF03
knob
cen
Physical coordinates on B73 genome (Mbp)

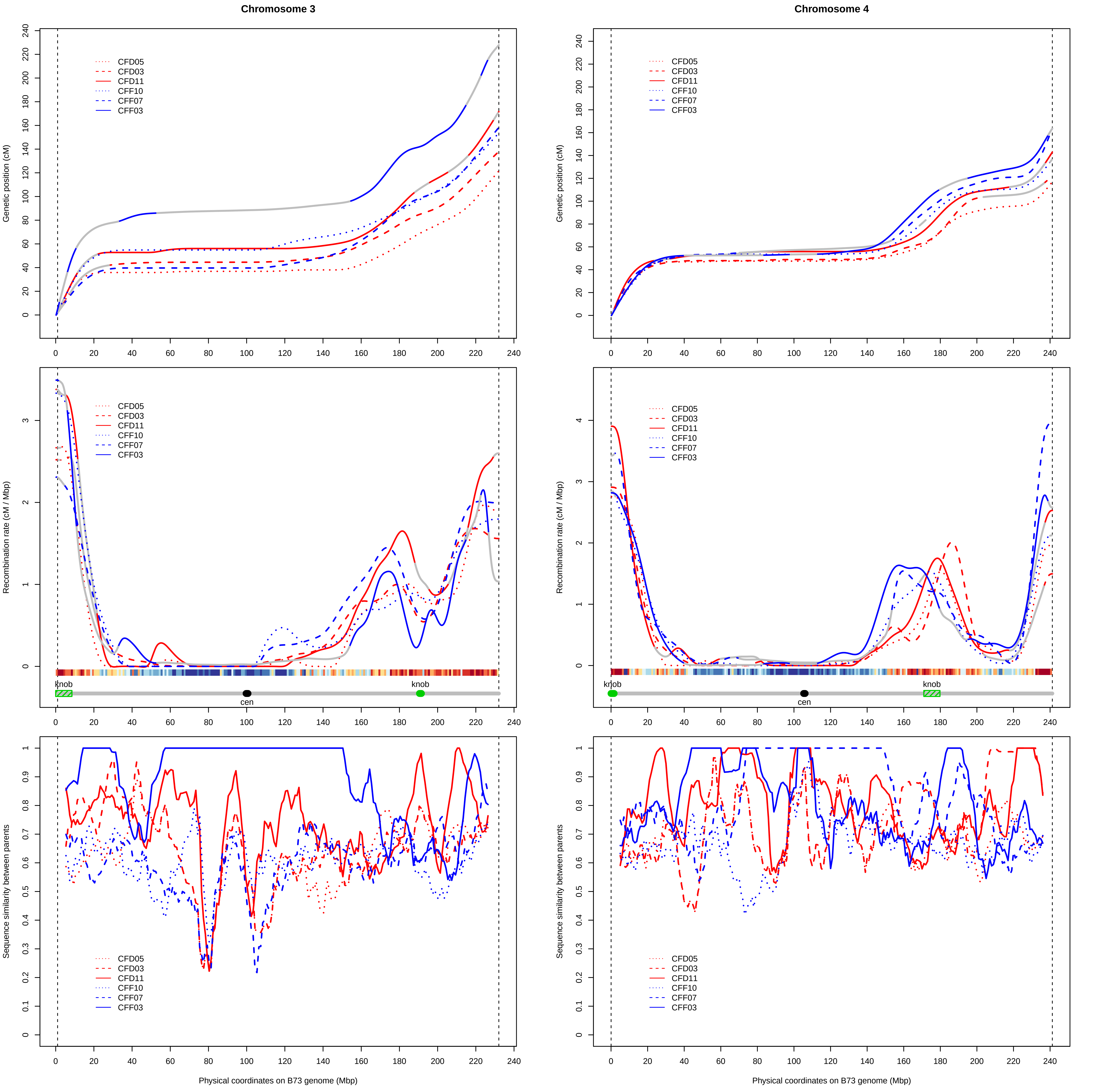
Chromosome 3
Chromosome 4
Genetic position (cM)
Recombination rate (cM / Mbp)
Sequence similarity between parents
Physical coordinates on B73 genome (Mbp)
CFD05
CFD03
CFD11
CFF10
CFF07
CFF03
knob
cen

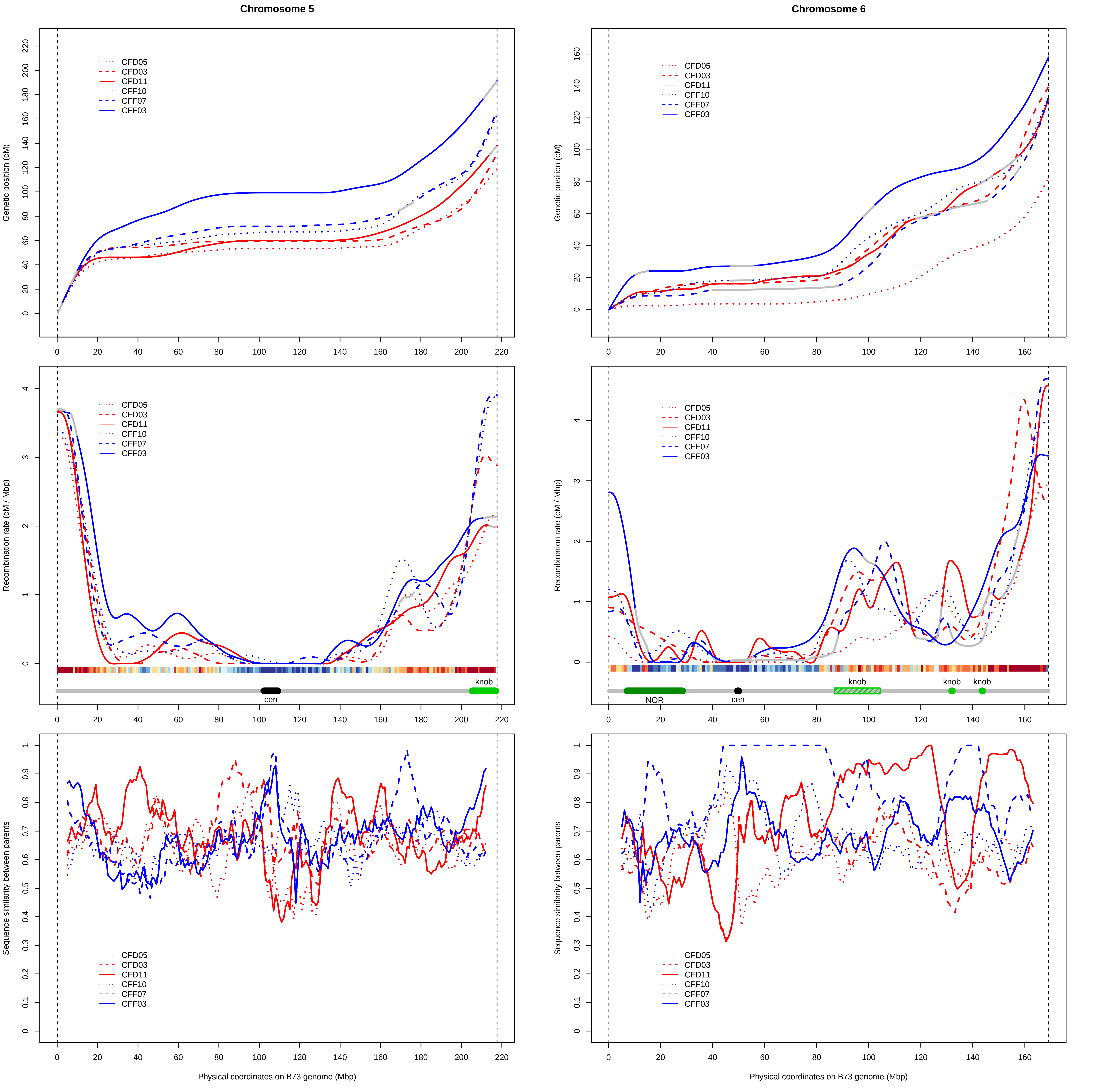
Chromosome 5
Chromosome 6
Genetic position (cM)
Recombination rate (cM / Mbp)
Sequence similarity between parents
Physical coordinates on B73 genome (Mbp)
CFD05
CFD03
CFD11
CFF10
CFF07
CFF03
cen
knob
NOR

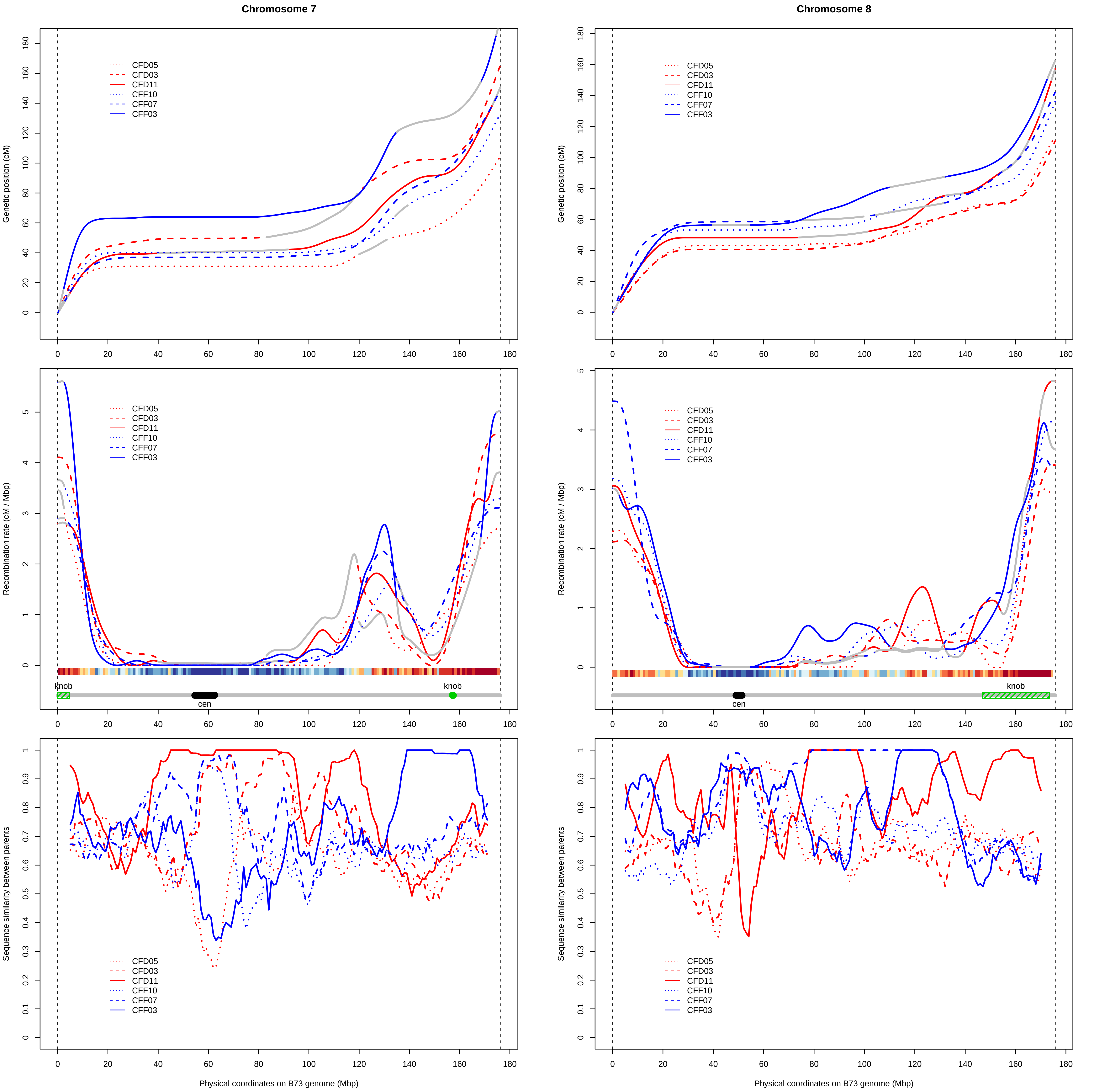

Chromosome 7
Chromosome 8
Genetic position (cM)
Recombination rate (cM / Mbp)
Sequence similarity between parents
Physical coordinates on B73 genome (Mbp)
CFD05
CFD03
CFD11
CFF10
CFF07
CFF03
knob
cen

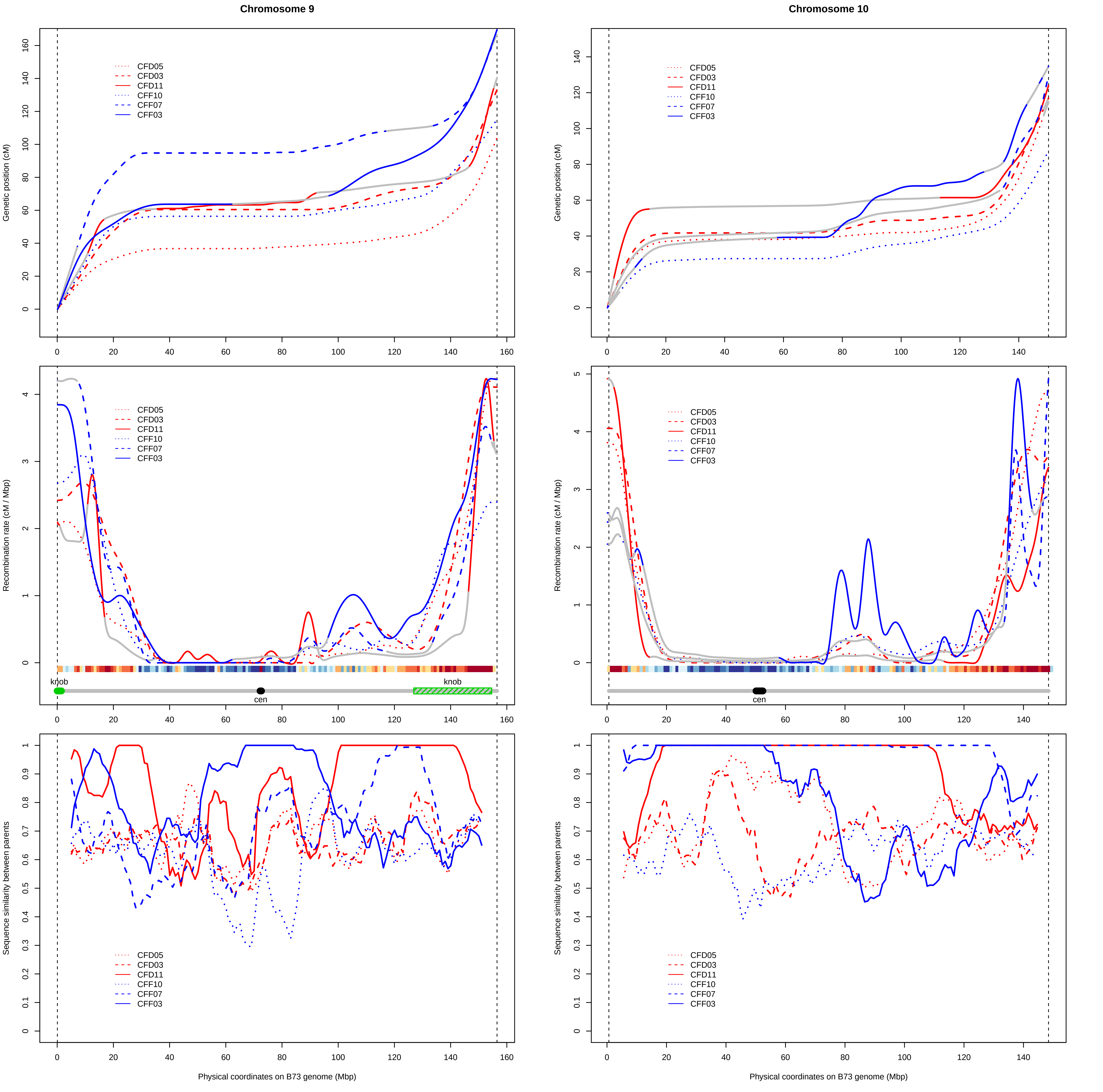

Chromosome 9
Chromosome 10
Genetic position (cM)
Recombination rate (cM / Mbp)
Sequence similarity between parents
Physical coordinates on B73 genome (Mbp)
CFD05
CFD03
CFD11
CFF10
CFF07
CFF03
knob
cen
knob
cen
