# Supplementary material for: Intraspecific variation of recombination rate in maize
Source: Genome Biol. 2013 Sep 19;14(9):R103. doi: 10.1186/gb-2013-14-9-r103 (PMC4053771; doi:10.1186/gb-2013-14-9-r103)
Supplement: Additional file 10: Figure S5 — Statistical comparison of genome-wide recombination landscapes between the 23 populations for all chromosomes pooled as well as for each chromosome. For each pairwise comparison test, both genetic map lengths were normalized to their average value, so the test is not affected by differences in the global recombination rate but only by differences in the shape of the recombination landscape. 'DentAll', 'FlintAll', and 'All' correspond, respectively, to pooled analyses of all Dent × Dent populations, all Flint × Flint populations, and all 23 populations together. Dark blue, light blue, green, yellow, and red correspond respectively to P ≥ 5.10-2, 10-3 ≤ P < 10-2, 10-4 ≤ P < 10-3, 10-5 ≤ P < 10-4, P < 10-5 where P is the P value of the pairwise comparison test, corrected for multiple testing (Bonferroni). Dendrograms indicate hierarchical clustering of -log10(P value) based on Euclidian distances, and were used to order the populations. [file gb-2013-14-9-r103-S10.pdf]

Comparison of recomb. landscapes between crosses

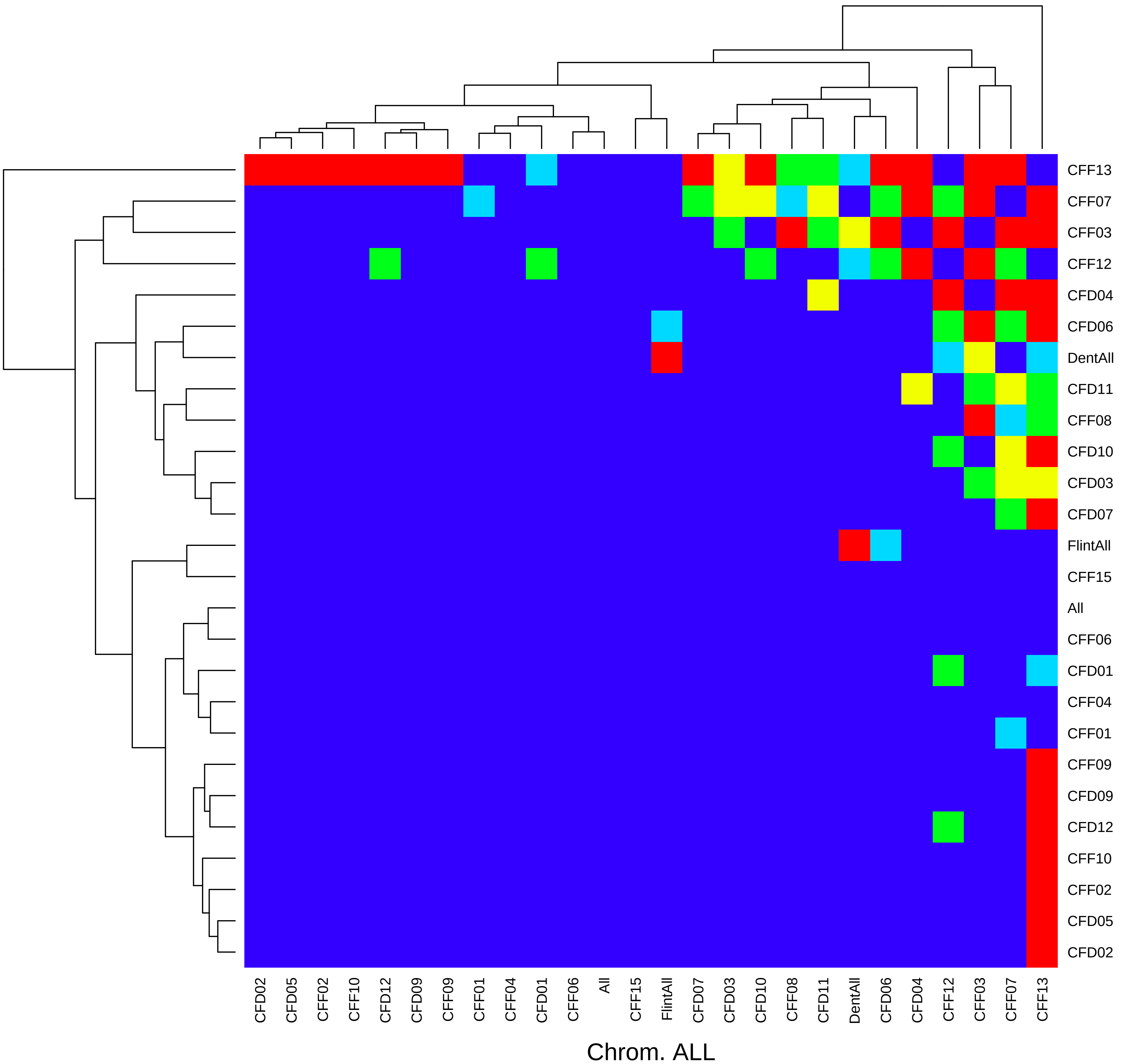

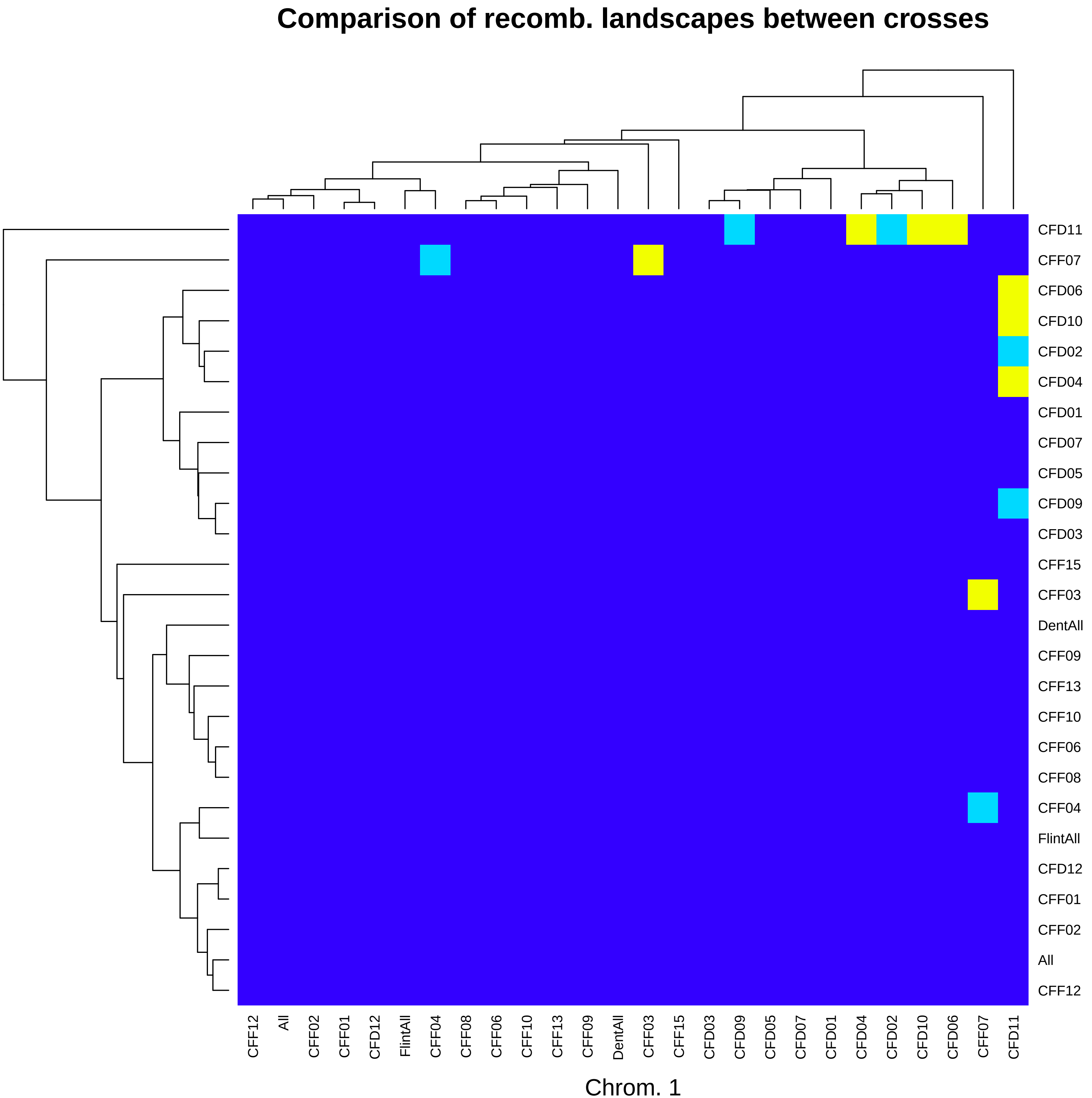
Comparison of recomb. landscapes between crosses
CFD11
CFF07
CFD06
CFD10
CFD02
CFD04
CFD01
CFD07
CFD05
CFD09
CFD03
CFF15
CFF03
DentAll
CFF09
CFF13
CFF10
CFF06
CFF08
CFF04
FlintAll
CFD12
CFF01
CFF02
All
CFF12
CFF12
All
CFF02
CFF01
CFD12
FlintAll
CFF04
CFF08
CFF06
CFF10
CFF13
CFF09
DentAll
CFF03
CFF15
CFD03
CFD09
CFD05
CFD07
CFD01
CFD04
CFD02
CFD10
CFD06
CFF07
CFD11
Chrom. 1

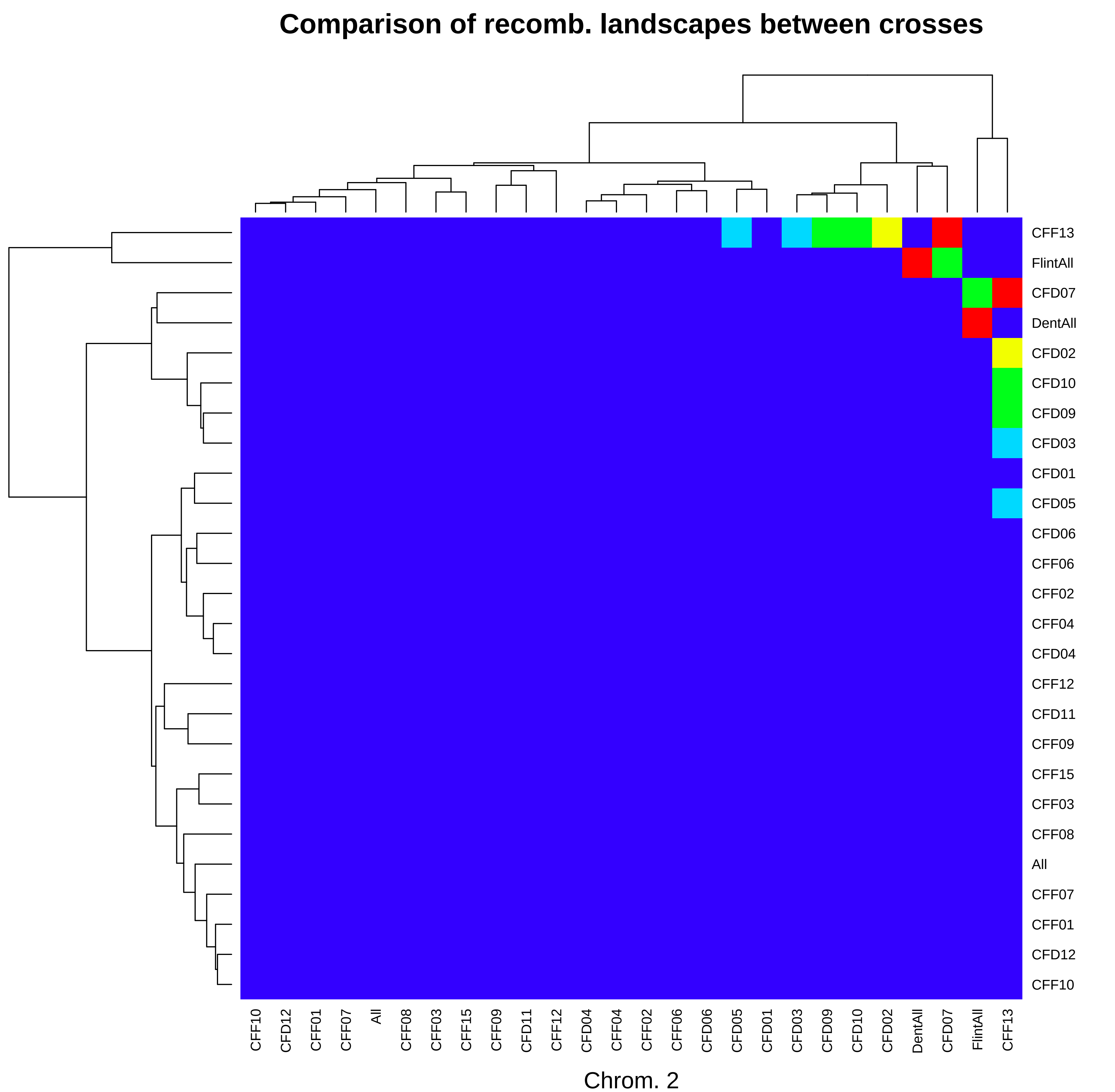
Comparison of recomb. landscapes between crosses
CFF13
FlintAll
CFD07
DentAll
CFD02
CFD10
CFD09
CFD03
CFD01
CFD05
CFD06
CFF06
CFF02
CFF04
CFD04
CFF12
CFD11
CFF09
CFF15
CFF03
CFF08
All
CFF07
CFF01
CFD12
CFF10
CFF10
CFD12
CFF01
CFF07
All
CFF08
CFF03
CFF15
CFF09
CFD11
CFF12
CFD04
CFF04
CFF02
CFF06
CFD06
CFD05
CFD01
CFD03
CFD09
CFD10
CFD02
DentAll
CFD07
FlintAll
CFF13
Chrom. 2

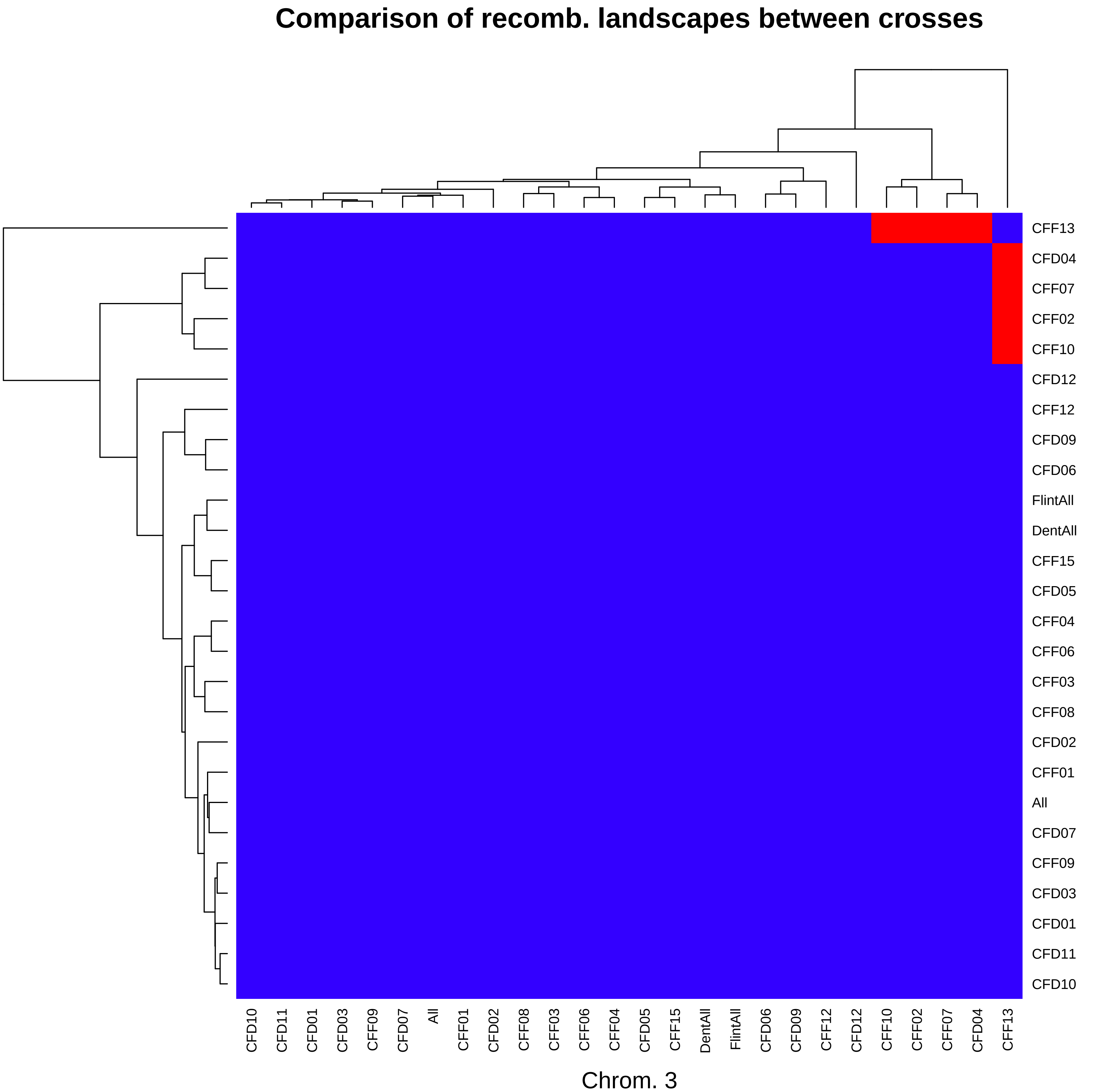
Comparison of recomb. landscapes between crosses
CFF13
CFD04
CFF07
CFF02
CFF10
CFD12
CFF12
CFD09
CFD06
FlintAll
DentAll
CFF15
CFD05
CFF04
CFF06
CFF03
CFF08
CFD02
CFF01
All
CFD07
CFF09
CFD03
CFD01
CFD11
CFD10
Chrom. 3

Comparison of recomb. landscapes between crosses

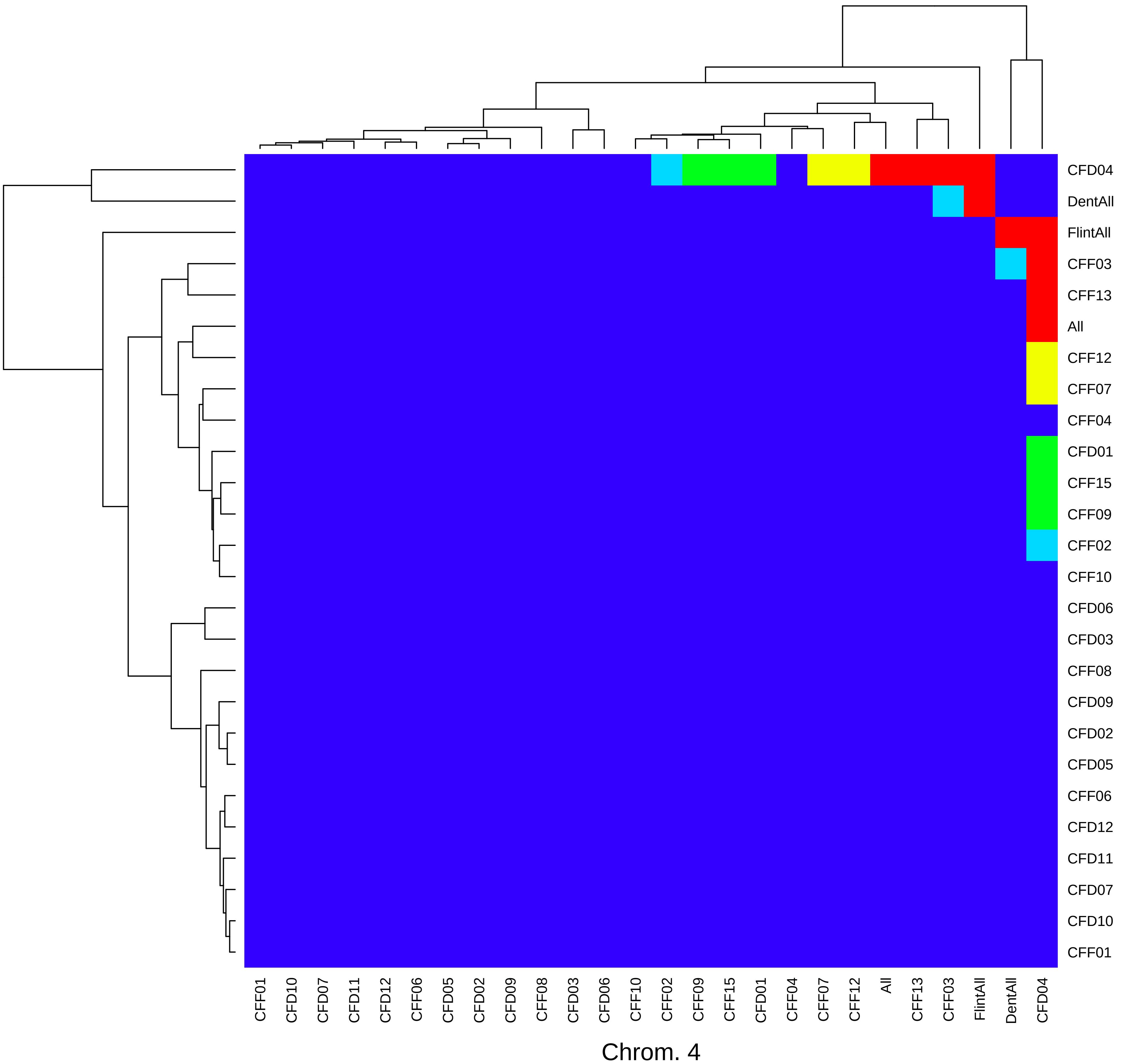

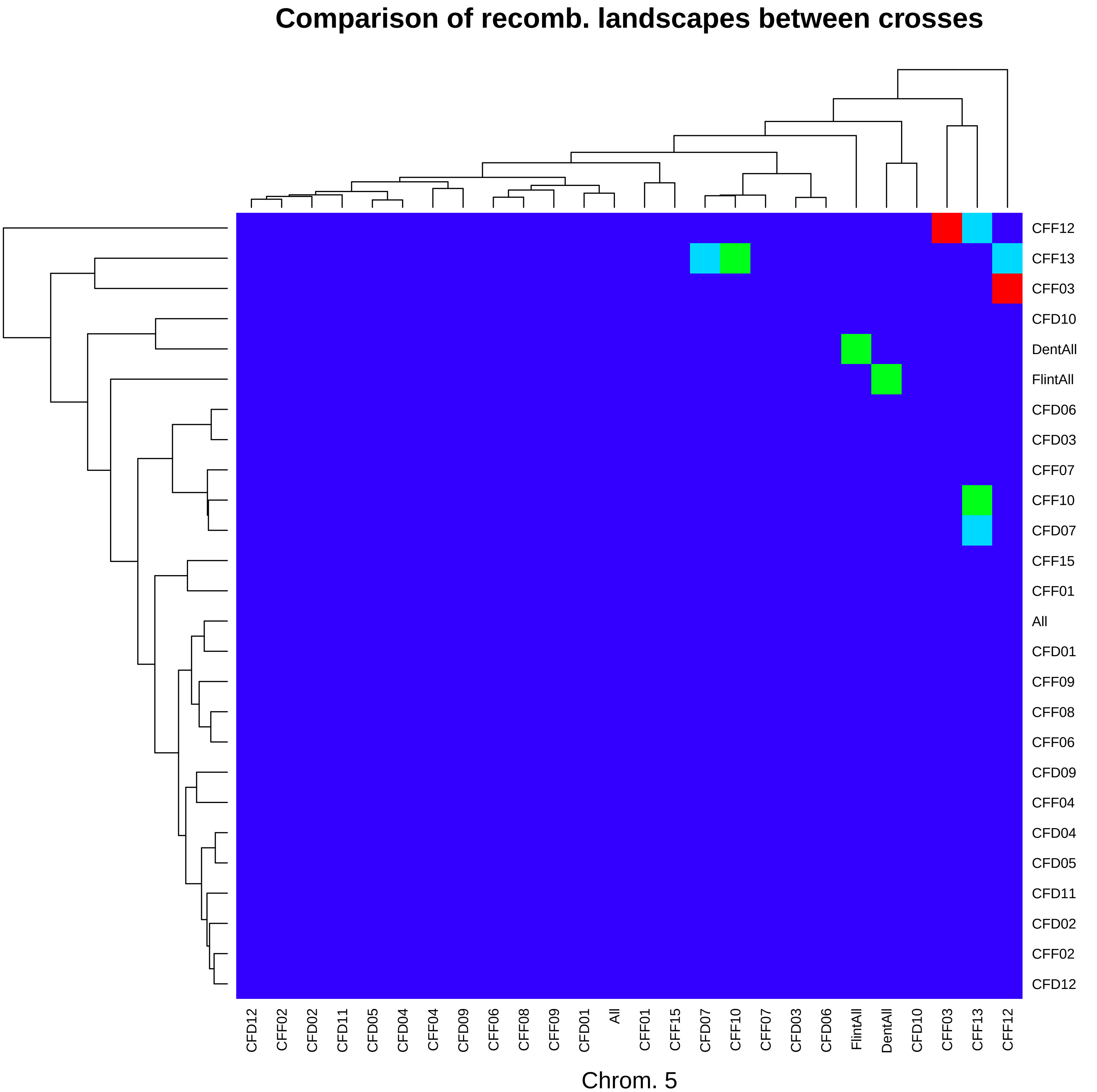
Comparison of recomb. landscapes between crosses
CFF12
CFF13
CFF03
CFD10
DentAll
FlintAll
CFD06
CFD03
CFF07
CFF10
CFD07
CFF15
CFF01
All
CFD01
CFF09
CFF08
CFF06
CFD09
CFF04
CFD04
CFD05
CFD11
CFD02
CFF02
CFD12
Chrom. 5

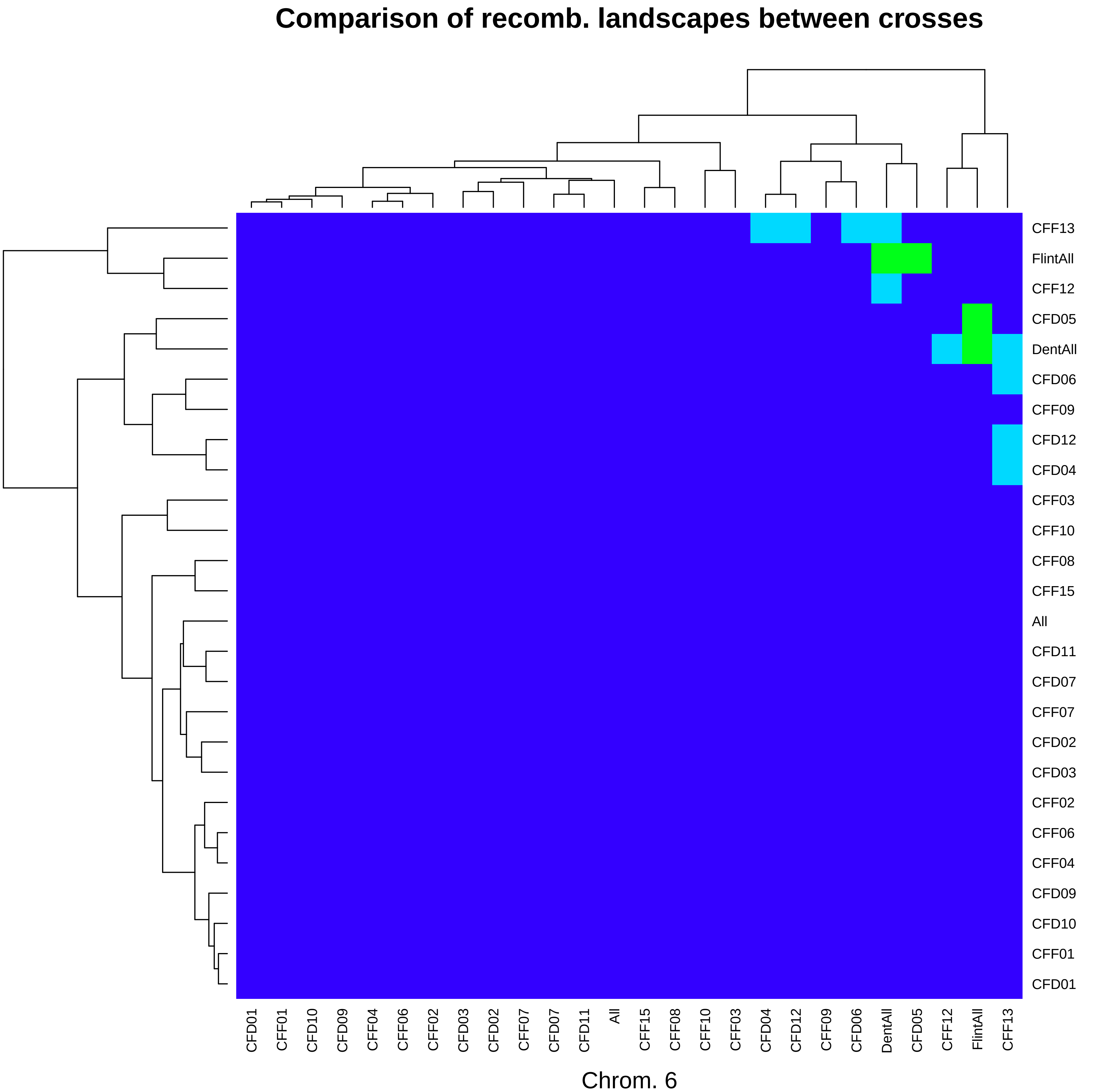
Comparison of recomb. landscapes between crosses
CFF13
FlintAll
CFF12
CFD05
DentAll
CFD06
CFF09
CFD12
CFD04
CFF03
CFF10
CFF08
CFF15
All
CFD11
CFD07
CFF07
CFD02
CFD03
CFF02
CFF06
CFF04
CFD09
CFD10
CFF01
CFD01
CFD01
CFF01
CFD10
CFD09
CFF04
CFF06
CFF02
CFD03
CFD02
CFF07
CFD07
CFD11
All
CFF15
CFF08
CFF10
CFF03
CFD04
CFD12
CFF09
CFD06
DentAll
CFD05
CFF12
FlintAll
CFF13
Chrom. 6

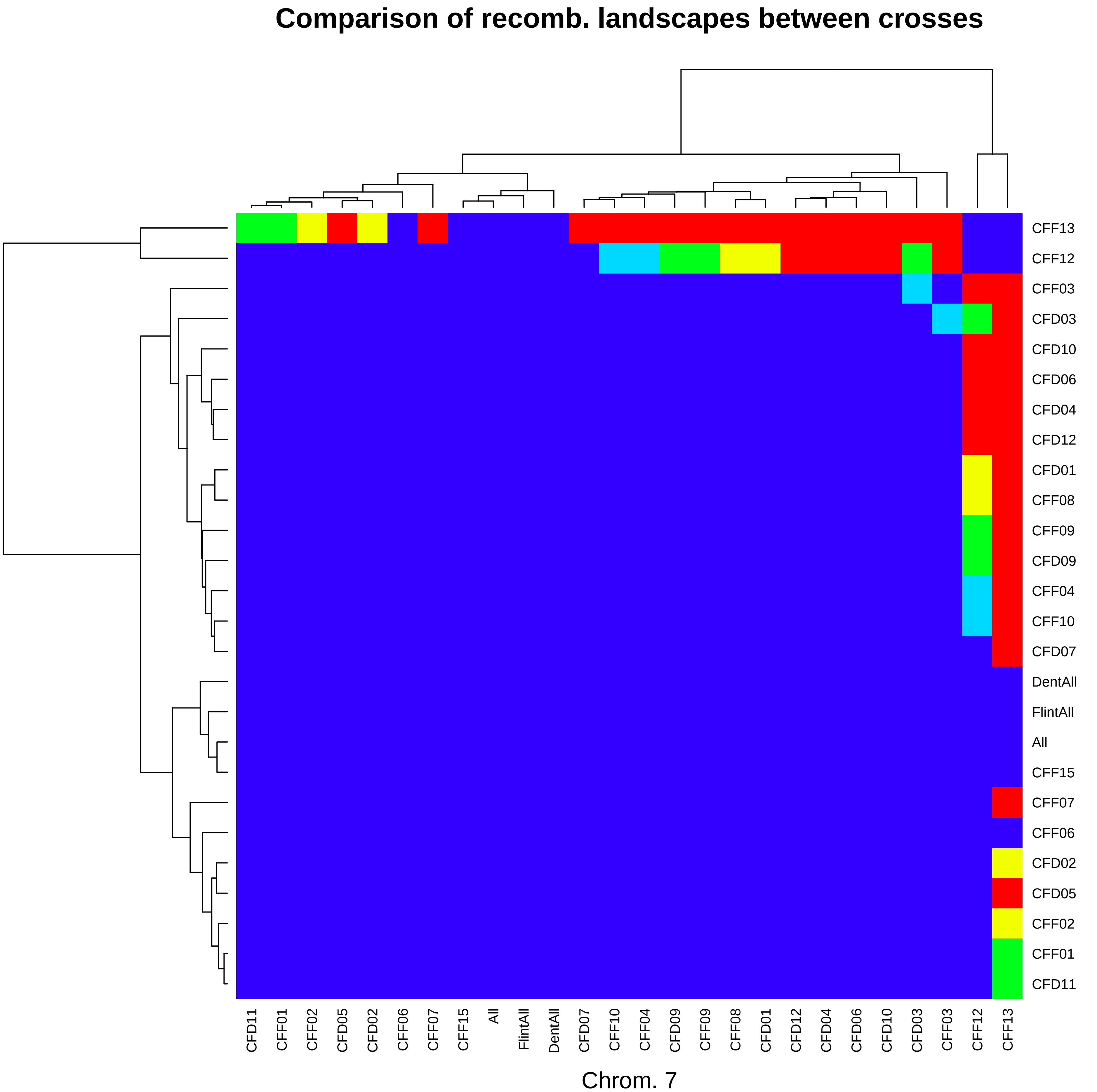
Comparison of recomb. landscapes between crosses
CFD11
CFF01
CFF02
CFD05
CFD02
CFF06
CFF07
CFF15
All
FlintAll
DentAll
CFD07
CFF10
CFF04
CFD09
CFF09
CFF08
CFD01
CFD12
CFD04
CFD06
CFD10
CFD03
CFF03
CFF12
CFF13
CFF13
CFF12
CFF03
CFD03
CFD10
CFD06
CFD04
CFD12
CFD01
CFF08
CFF09
CFD09
CFF04
CFF10
CFD07
DentAll
FlintAll
All
CFF15
CFF07
CFF06
CFD02
CFD05
CFF02
CFF01
CFD11
Chrom. 7

Comparison of recomb. landscapes between crosses

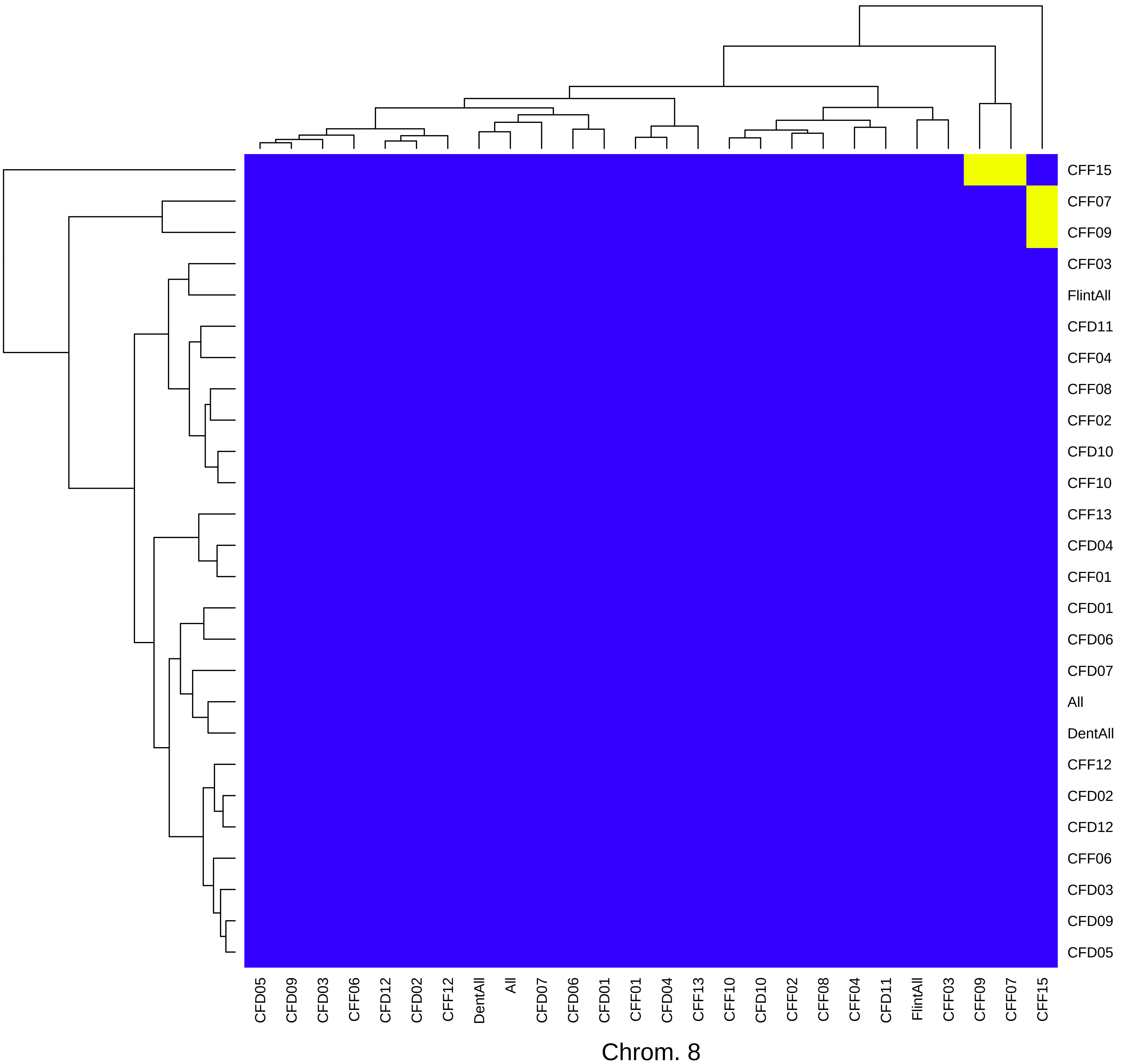

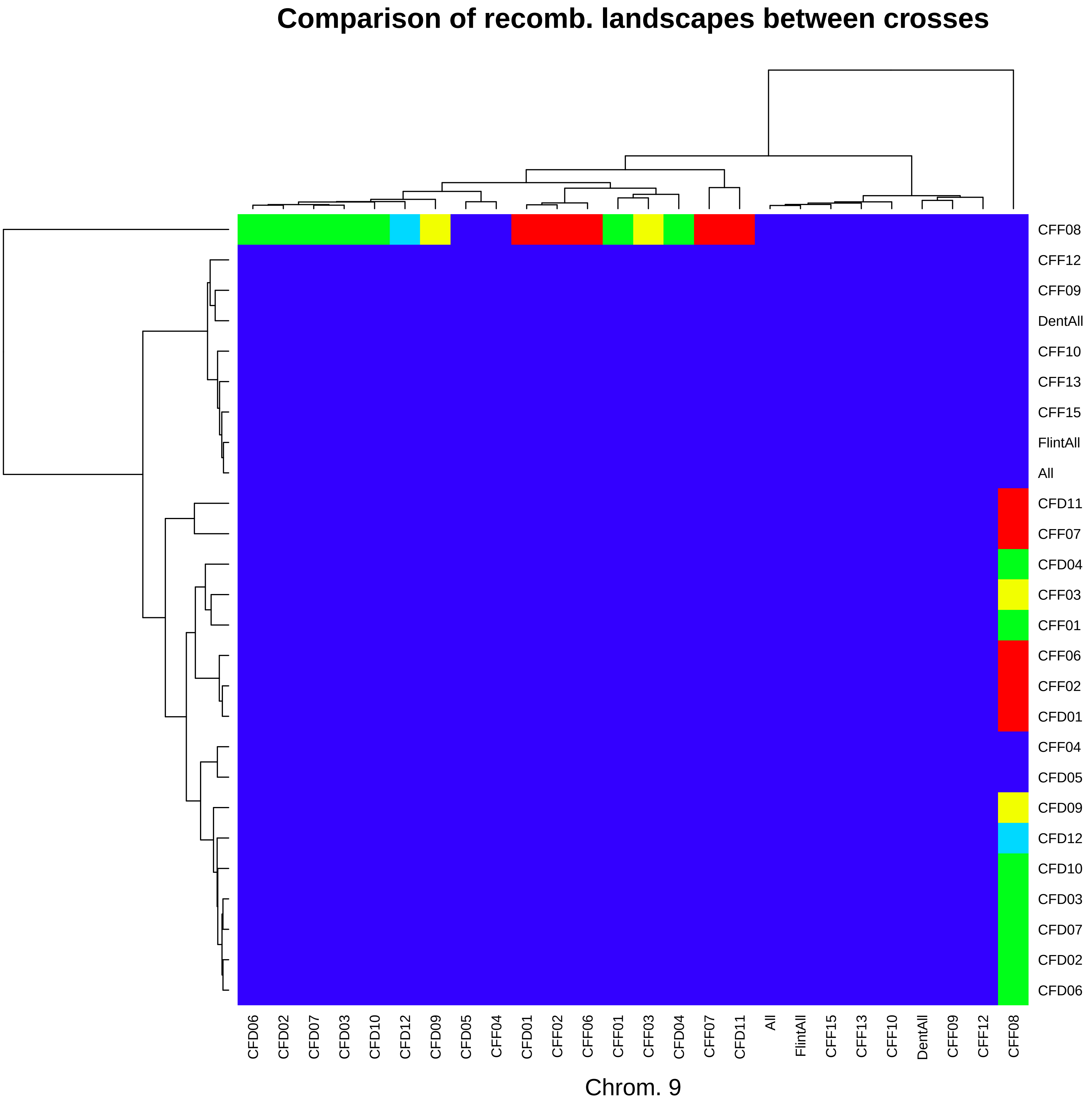
Comparison of recomb. landscapes between crosses
CFF08
CFF12
CFF09
DentAll
CFF10
CFF13
CFF15
FlintAll
All
CFD11
CFF07
CFD04
CFF03
CFF01
CFF06
CFF02
CFD01
CFF04
CFD05
CFD09
CFD12
CFD10
CFD03
CFD07
CFD02
CFD06
CFD06
CFD02
CFD07
CFD03
CFD10
CFD12
CFD09
CFD05
CFF04
CFD01
CFF02
CFF06
CFF01
CFF03
CFD04
CFF07
CFD11
All
FlintAll
CFF15
CFF13
CFF10
DentAll
CFF09
CFF12
CFF08
Chrom. 9

Comparison of recomb. landscapes between crosses

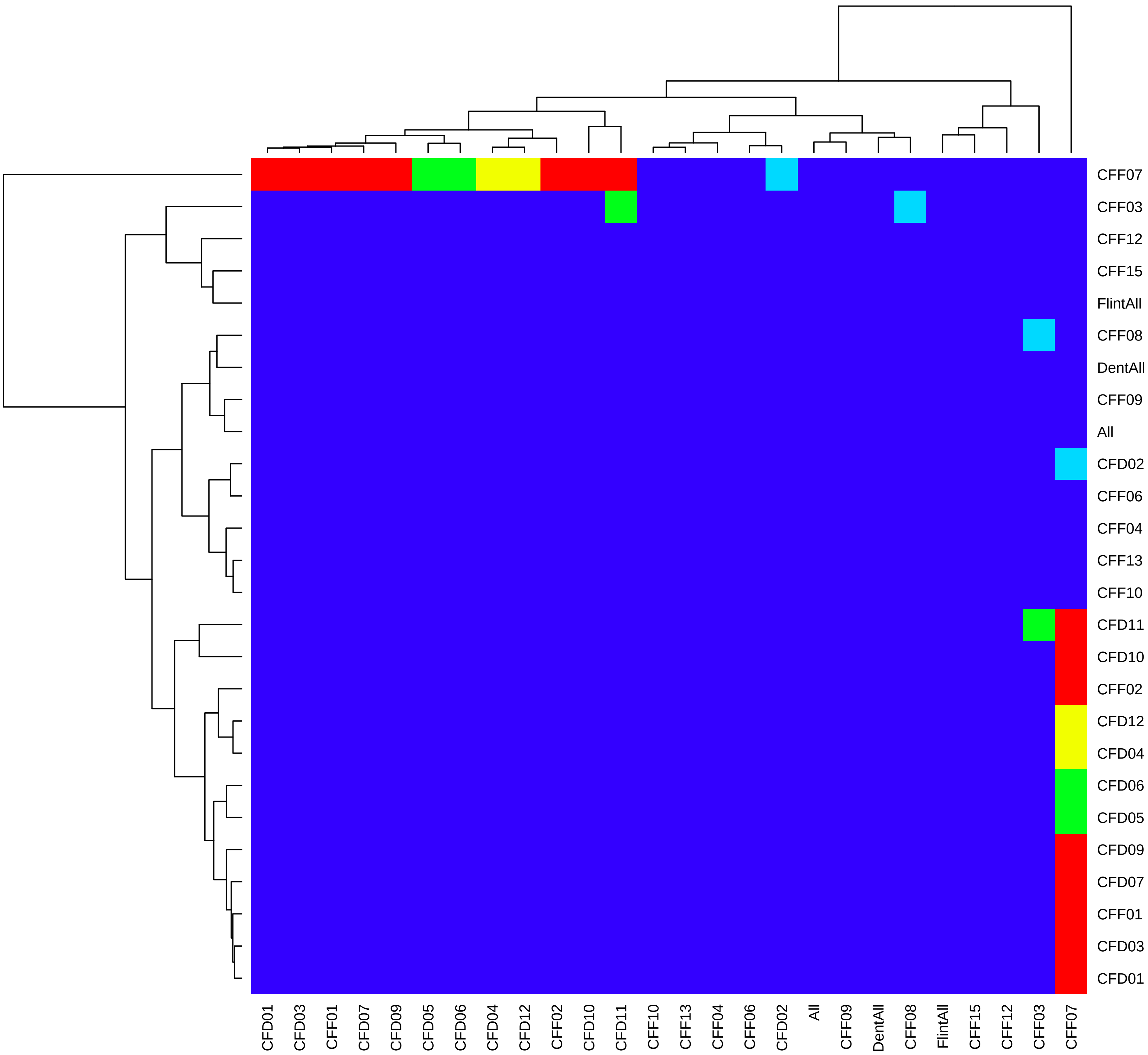

Chrom. 10
