# Supplementary material for: Intraspecific variation of recombination rate in maize
Source: Genome Biol. 2013 Sep 19;14(9):R103. doi: 10.1186/gb-2013-14-9-r103 (PMC4053771; doi:10.1186/gb-2013-14-9-r103)
Supplement: Additional file 11: Figure S6 — Illustration of the statistical test used to compare recombination landscapes between the pooled data of all Dent × Dent (red) and Flint × Flint (blue) populations. Within regions excluded from the analysis for one population, the data were imputed from the other pool for conservativeness of the test (Additional file 8). Solid curves: Marey maps normalized to the average genetic length, so the comparison focuses on differences in the shape of the recombination landscapes and is not affected by differences in the values of chromosome genetic lengths. Dotted curves: first derivative of the normalized Marey maps, indicating the recombination landscape along the chromosome. The black rectangles show the 10 bins used for the analysis (from left to right: bins 1 to 10). Bin boundaries were chosen so each bin contained regions of the same genetic length. On top of each bar, a black vertical arrow indicates the difference between both populations in average recombination rates over the bin considered, and the error bars indicate the 95% confidence intervals of these average recombination rates. [file gb-2013-14-9-r103-S11.pdf]

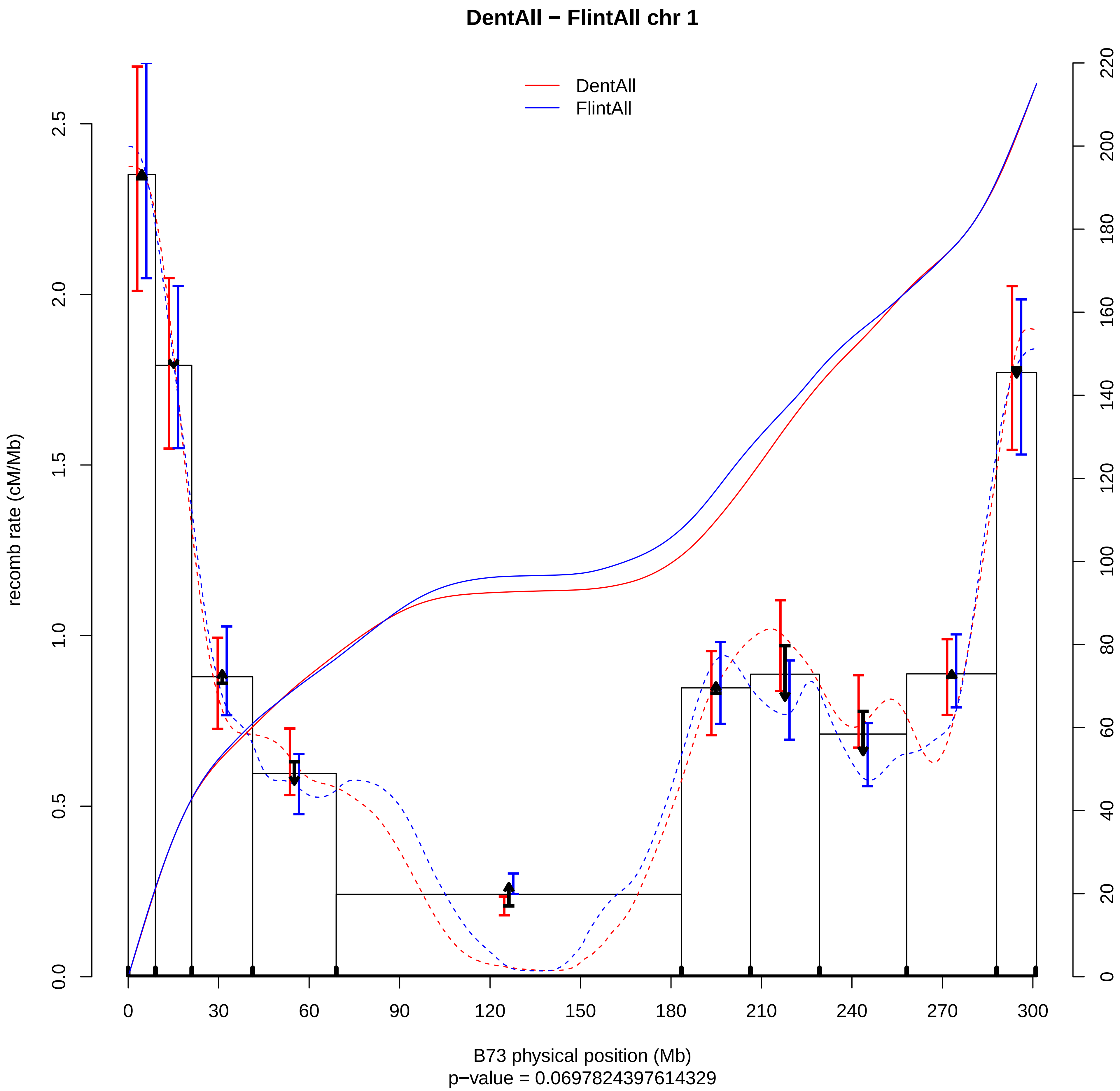

DentAll − FlintAll chr 1
DentAll
FlintAll
recomb rate (cM/Mb)
0.0
0.5
1.0
1.5
2.0
2.5
0
20
40
60
80
100
120
140
160
180
200
220
0
30
60
90
120
150
180
210
240
270
300
B73 physical position (Mb)
p−value = 0.0697824397614329

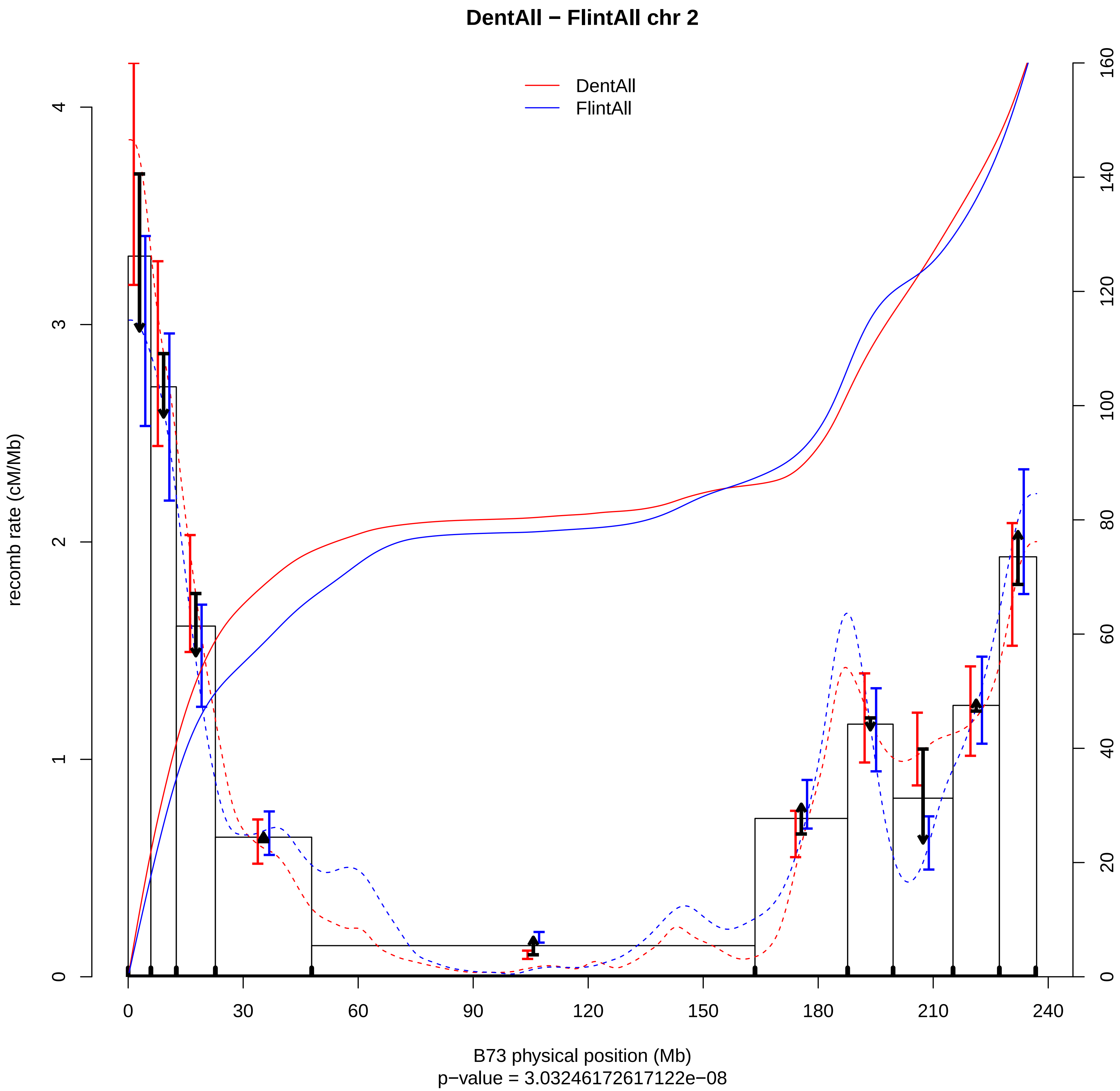

DentAll − FlintAll chr 2
DentAll
FlintAll
recomb rate (cM/Mb)
0
1
2
3
4
0
20
40
60
80
100
120
140
160
0
30
60
90
120
150
180
210
240
B73 physical position (Mb)
p−value = 3.03246172617122e−08

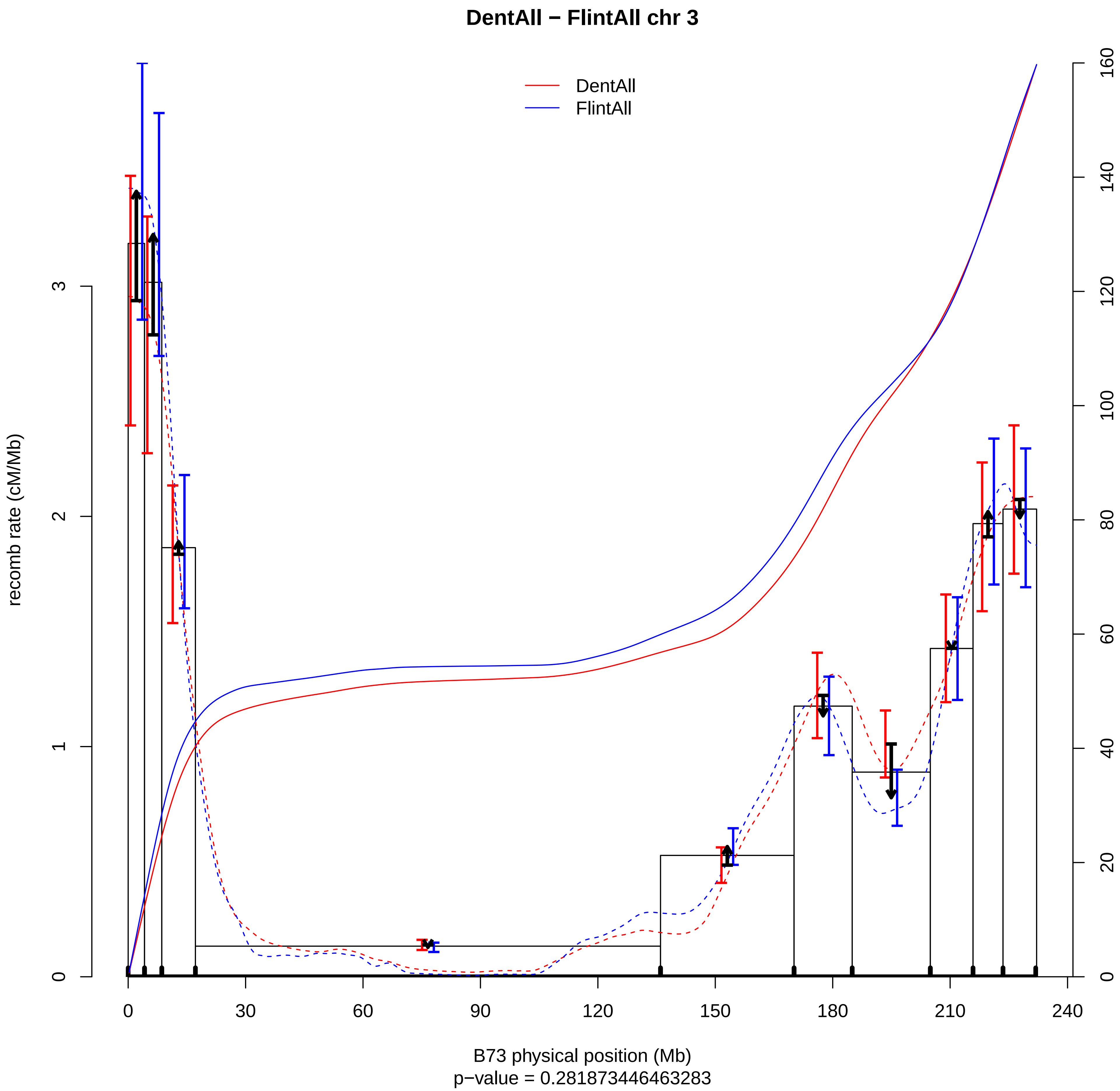

DentAll − FlintAll chr 3
DentAll
FlintAll
recomb rate (cM/Mb)
0
1
2
3
0
20
40
60
80
100
120
140
160
0
30
60
90
120
150
180
210
240
B73 physical position (Mb)
p−value = 0.281873446463283

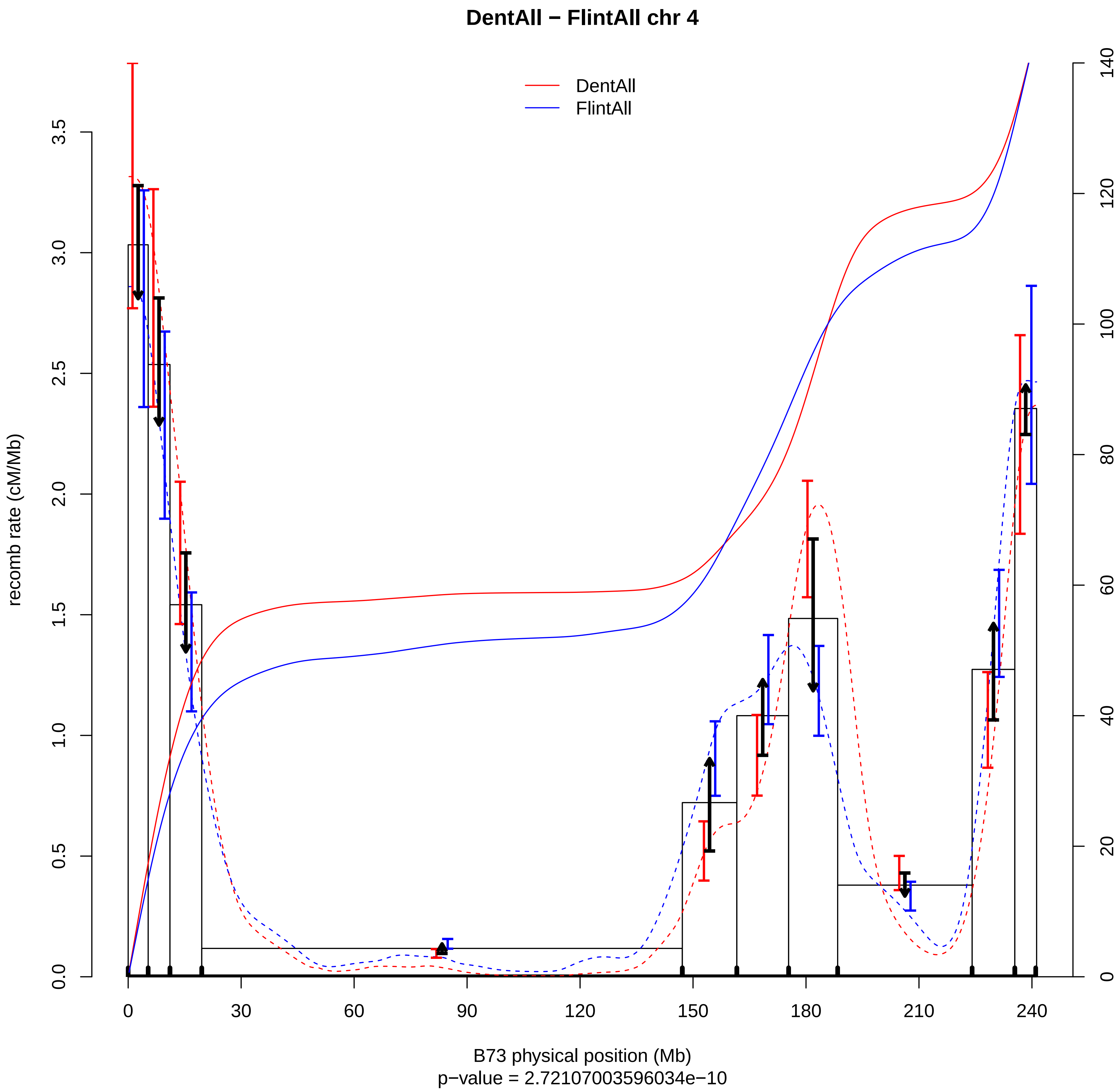
DentAll − FlintAll chr 4
DentAll
FlintAll
recomb rate (cM/Mb)
0.0
0.5
1.0
1.5
2.0
2.5
3.0
3.5
0
20
40
60
80
100
120
140
0
30
60
90
120
150
180
210
240
B73 physical position (Mb)
p−value = 2.72107003596034e−10

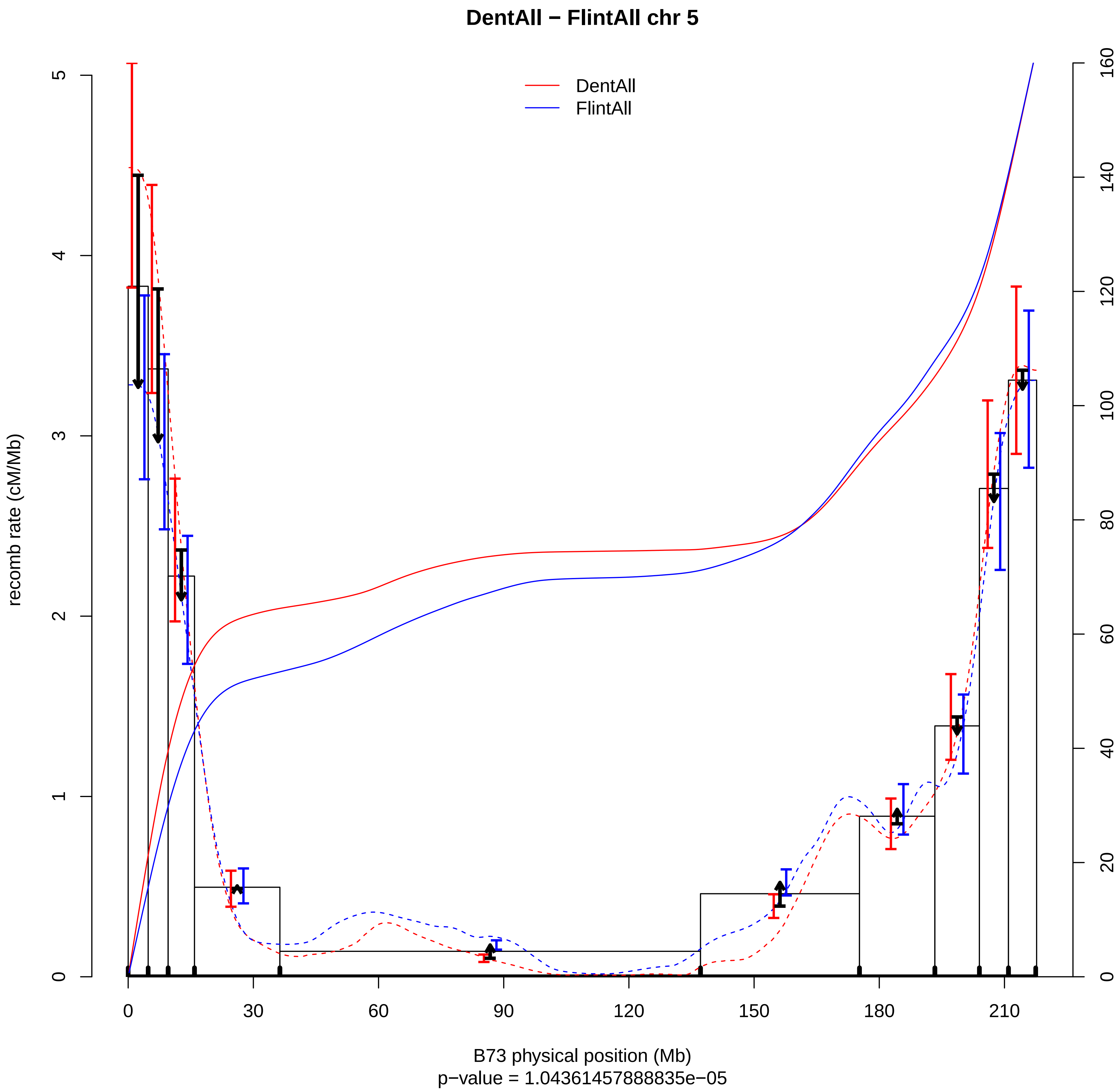

DentAll − FlintAll chr 5
DentAll
FlintAll
recomb rate (cM/Mb)
B73 physical position (Mb)
p−value = 1.04361457888835e−05
0
1
2
3
4
5
0
30
60
90
120
150
180
210
0
20
40
60
80
100
120
140
160

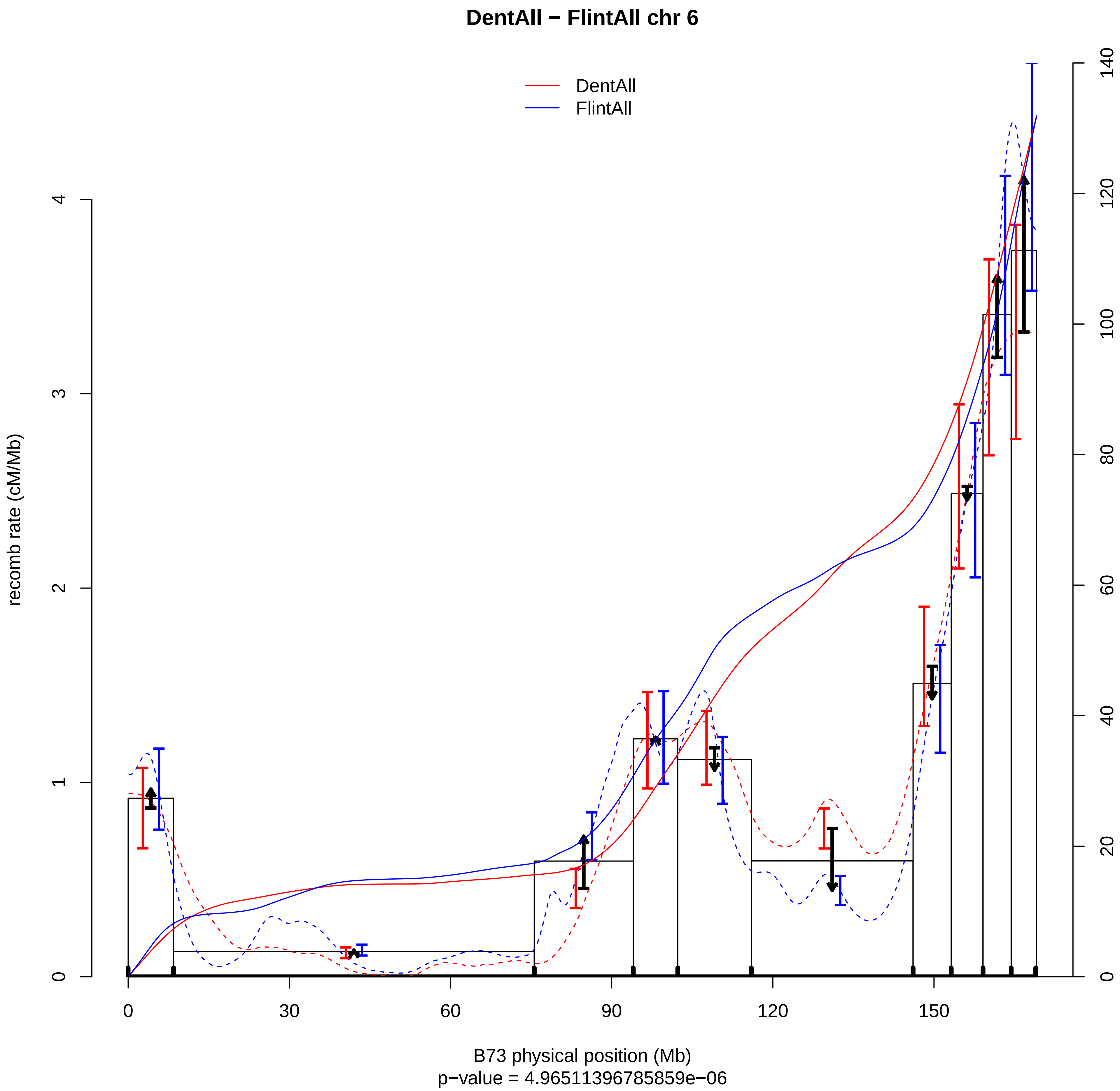
DentAll − FlintAll chr 6
DentAll
FlintAll
recomb rate (cM/Mb)
0
1
2
3
4
0
20
40
60
80
100
120
140
0
30
60
90
120
150
B73 physical position (Mb)
p−value = 4.96511396785859e−06

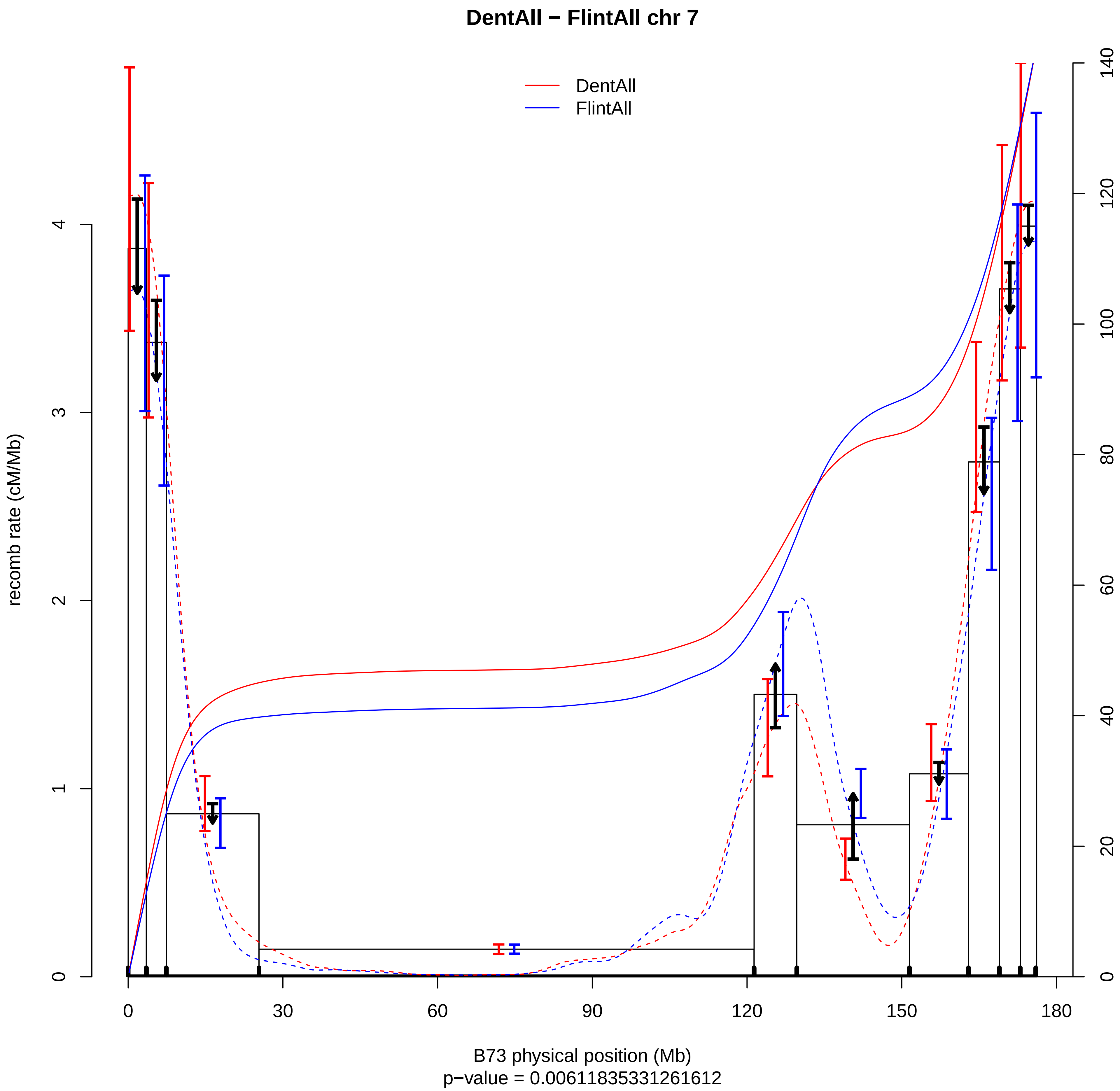
DentAll − FlintAll chr 7
DentAll
FlintAll
recomb rate (cM/Mb)
0
1
2
3
4
0
20
40
60
80
100
120
140
0
30
60
90
120
150
180
B73 physical position (Mb)
p−value = 0.00611835331261612

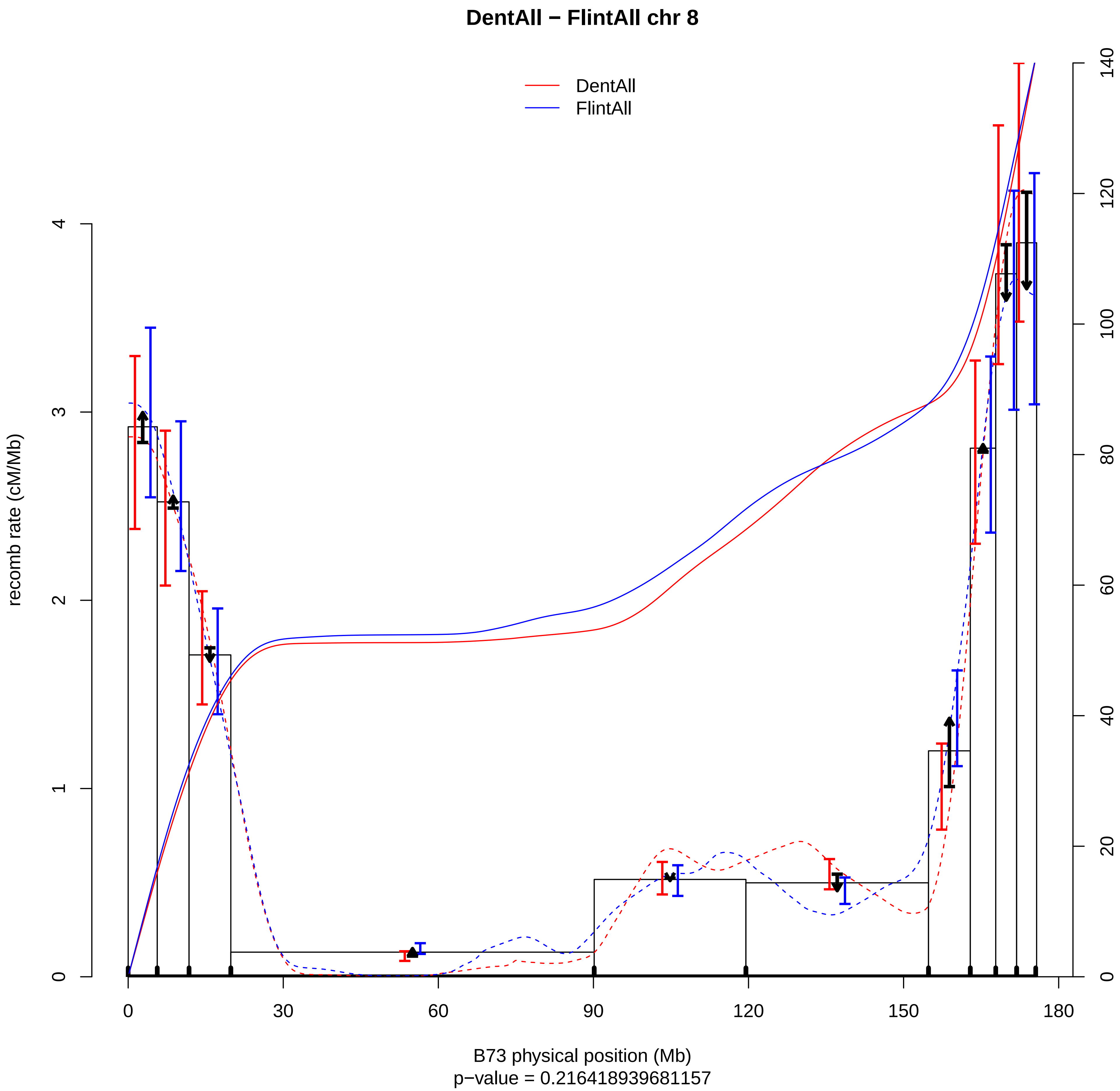

DentAll − FlintAll chr 8
DentAll
FlintAll
recomb rate (cM/Mb)
0
1
2
3
4
0
20
40
60
80
100
120
140
0
30
60
90
120
150
180
B73 physical position (Mb)
p−value = 0.216418939681157

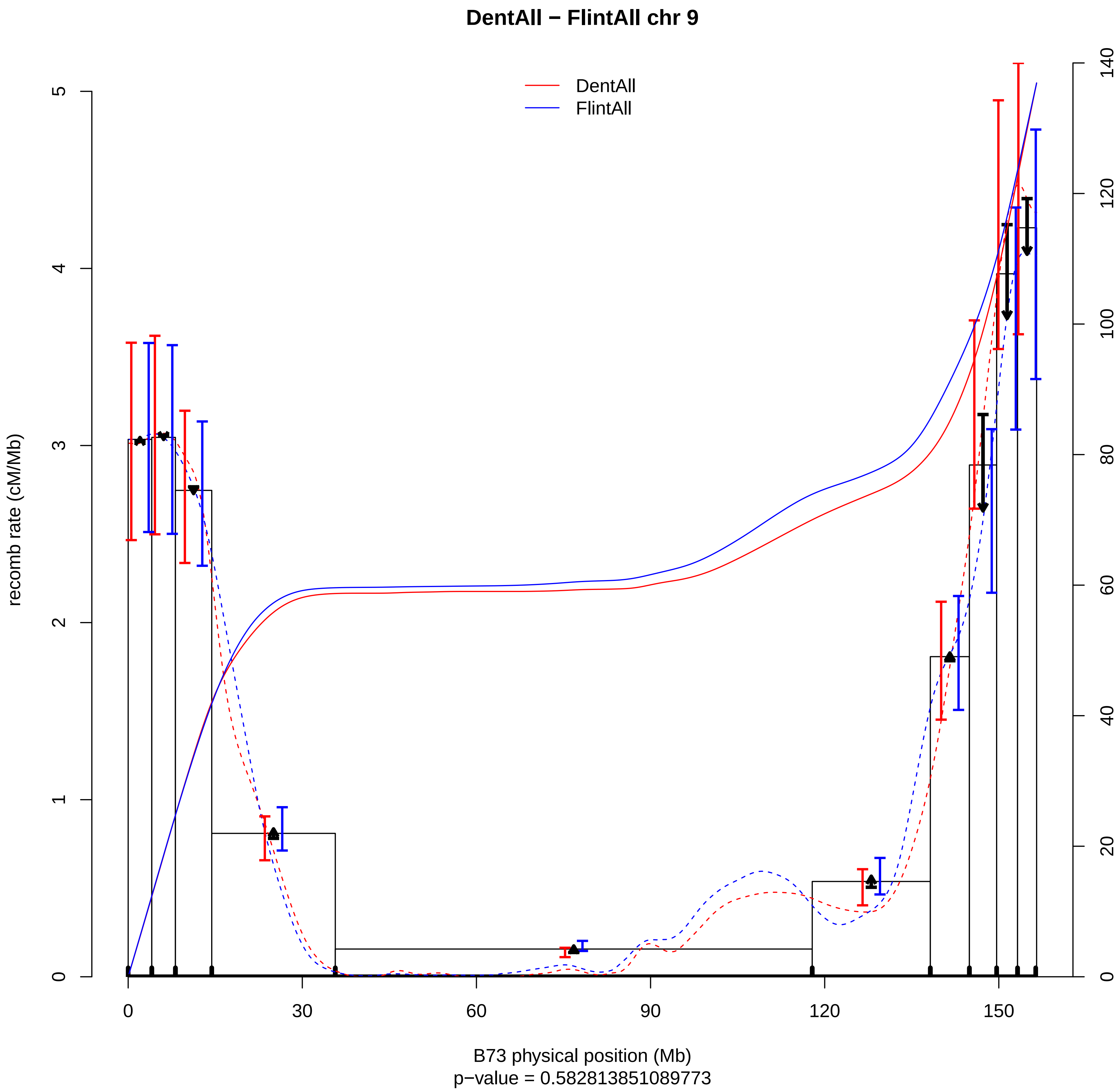
DentAll − FlintAll chr 9
DentAll
FlintAll
recomb rate (cM/Mb)
0
1
2
3
4
5
0
20
40
60
80
100
120
140
0
30
60
90
120
150
B73 physical position (Mb)
p−value = 0.582813851089773

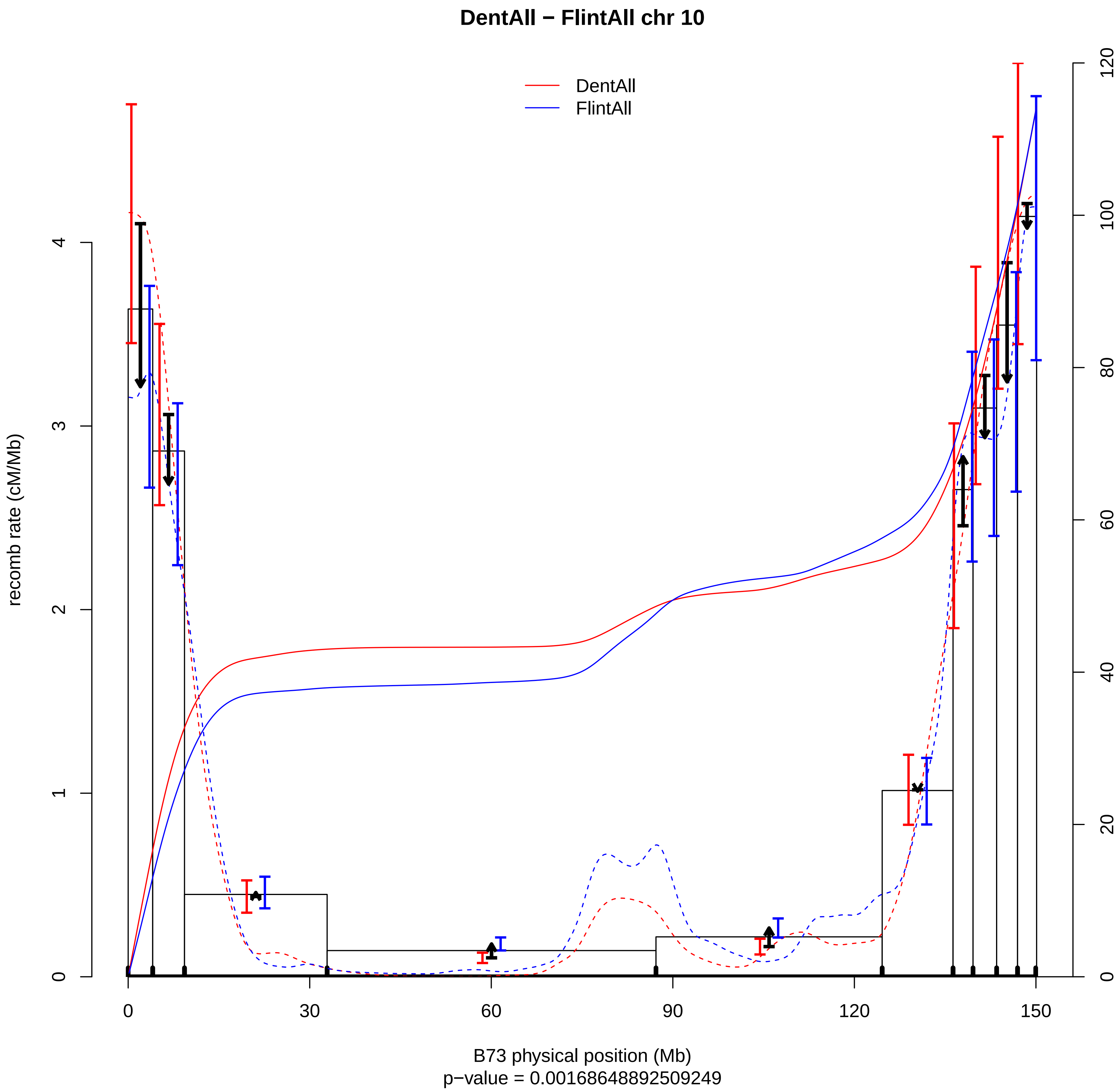

DentAll − FlintAll chr 10
DentAll
FlintAll
recomb rate (cM/Mb)
0
1
2
3
4
0
20
40
60
80
100
120
0
30
60
90
120
150
B73 physical position (Mb)
p−value = 0.00168648892509249
